# Comparison of Multivariable Logistic Regression and Other Machine Learning Algorithms for Prognostic Prediction Studies in Pregnancy Care: Systematic Review and Meta-Analysis

**DOI:** 10.2196/16503

**Published:** 2020-11-17

**Authors:** Herdiantri Sufriyana, Atina Husnayain, Ya-Lin Chen, Chao-Yang Kuo, Onkar Singh, Tso-Yang Yeh, Yu-Wei Wu, Emily Chia-Yu Su

**Affiliations:** 1 Graduate Institute of Biomedical Informatics College of Medical Science and Technology Taipei Medical University Taipei Taiwan; 2 Department of Medical Physiology College of Medicine University of Nahdlatul Ulama Surabaya Surabaya Indonesia; 3 Department of Biostatistics, Epidemiology, and Population Health Faculty of Medicine, Public Health and Nursing Universitas Gadjah Mada Yogyakarta Indonesia; 4 School of Pharmacy College of Pharmacy Taipei Medical University Taipei Taiwan; 5 Bioinformatics Program, Taiwan International Graduate Program Institute of Information Science Academia Sinica Taipei Taiwan; 6 Institute of Biomedical Informatics National Yang-Ming University Taipei Taiwan; 7 School of Dentistry College of Oral Medicine Taipei Medical University Taipei Taiwan; 8 Clinical Big Data Research Center Taipei Medical University Hospital Taipei Taiwan

**Keywords:** machine learning, pregnancy complications, prognosis, clinical prediction rule, meta-analysis, systematic review

## Abstract

**Background:**

Predictions in pregnancy care are complex because of interactions among multiple factors. Hence, pregnancy outcomes are not easily predicted by a single predictor using only one algorithm or modeling method.

**Objective:**

This study aims to review and compare the predictive performances between logistic regression (LR) and other machine learning algorithms for developing or validating a multivariable prognostic prediction model for pregnancy care to inform clinicians’ decision making.

**Methods:**

Research articles from MEDLINE, Scopus, Web of Science, and Google Scholar were reviewed following several guidelines for a prognostic prediction study, including a risk of bias (ROB) assessment. We report the results based on the PRISMA (Preferred Reporting Items for Systematic Reviews and Meta-Analyses) guidelines. Studies were primarily framed as PICOTS (population, index, comparator, outcomes, timing, and setting): Population: men or women in procreative management, pregnant women, and fetuses or newborns; Index: multivariable prognostic prediction models using non-LR algorithms for risk classification to inform clinicians’ decision making; Comparator: the models applying an LR; Outcomes: pregnancy-related outcomes of procreation or pregnancy outcomes for pregnant women and fetuses or newborns; Timing: pre-, inter-, and peripregnancy periods (predictors), at the pregnancy, delivery, and either puerperal or neonatal period (outcome), and either short- or long-term prognoses (time interval); and Setting: primary care or hospital. The results were synthesized by reporting study characteristics and ROBs and by random effects modeling of the difference of the logit area under the receiver operating characteristic curve of each non-LR model compared with the LR model for the same pregnancy outcomes. We also reported between-study heterogeneity by using *τ^2^* and *I^2^*.

**Results:**

Of the 2093 records, we included 142 studies for the systematic review and 62 studies for a meta-analysis. Most prediction models used LR (92/142, 64.8%) and artificial neural networks (20/142, 14.1%) among non-LR algorithms. Only 16.9% (24/142) of studies had a low ROB. A total of 2 non-LR algorithms from low ROB studies significantly outperformed LR. The first algorithm was a random forest for preterm delivery (logit AUROC 2.51, 95% CI 1.49-3.53; *I^2^*=86%; *τ^2^*=0.77) and pre-eclampsia (logit AUROC 1.2, 95% CI 0.72-1.67; *I^2^*=75%; *τ^2^*=0.09). The second algorithm was gradient boosting for cesarean section (logit AUROC 2.26, 95% CI 1.39-3.13; *I^2^*=75%; *τ^2^*=0.43) and gestational diabetes (logit AUROC 1.03, 95% CI 0.69-1.37; *I^2^*=83%; *τ^2^*=0.07).

**Conclusions:**

Prediction models with the best performances across studies were not necessarily those that used LR but also used random forest and gradient boosting that also performed well. We recommend a reanalysis of existing LR models for several pregnancy outcomes by comparing them with those algorithms that apply standard guidelines.

**Trial Registration:**

PROSPERO (International Prospective Register of Systematic Reviews) CRD42019136106; https://www.crd.york.ac.uk/prospero/display_record.php?RecordID=136106

## Introduction

### Background

Pregnancy is a common health condition that requires long-term rigorous care to anticipate adverse outcomes. Most pregnancy outcomes are identified after delivery; however, these are results of interactions among multiple factors occurring for many weeks beforehand. The number of factors and their interactions along with the time intervals make predictions of pregnancy outcomes very complicated. Multiple or multivariable logistic regression (LR) is widely used to deal with similar multifactorial problems in health outcome research [1]. Applied to medicine, statistics, and machine learning (computer science), this algorithm fits multiple parameters in a prediction model by assuming that predictors are linearly and additively related to an outcome [2]. Nevertheless, nonlinear problems commonly occur in human physiology because of complex interactions, such that a linear model might not be capable of adequately predicting outcomes [3]. With the growth of machine learning applications in health care, applying other algorithms may scale up the solution space for accurate predictions of pregnancy outcomes long before giving birth.

Despite improvements in maternal and neonatal mortality, conditions still differ between developing and developed countries or regions [4]. The most common causes of maternal deaths are hemorrhage, hypertension, and sepsis [5], whereas the causes of neonatal deaths are mostly due to prematurity, birth asphyxia, and infections [6]. Postpartum hemorrhage and sepsis are further compounded by multiple causes and risk factors [7,8], and hypertension in pregnancy or prematurity is associated with multiple mechanisms [9,10]. The aforementioned diseases and complications cannot be very easily predicted by a single epidemiological predictor, a single measure by a medical device, or a single biomarker. Furthermore, interactions among multiple predictors also might not be captured by a single machine learning algorithm including LR. Therefore, a prediction study may need to compare multiple machine learning algorithms to develop a prognostic prediction model that uses multiple predictors.

Machine learning algorithms have long been applied for clinical prediction purposes. A support vector machine demonstrated a summary of receiver operating characteristics (ROCs) of >90% for breast cancer prognostic prediction [11]. To predict therapeutic outcomes in depression, the pooled estimated accuracy of machine learning algorithms was 0.82 (95% CI 0.77-0.87) [12]. However, the difference in the logit area under the ROC curve (AUROC) was 0.00 (95% CI −0.18 to 0.18) between LR and machine learning in studies with a low risk of bias (ROB) [13]. A similar conclusion was found for predicting intracerebral hemorrhage (*P*=.49) outlined in a systematic review [14]. These previous results imply that (1) machine learning algorithms may or may not perform better than traditional modeling by LR and (2) applying only a single algorithm may cause an investigator to lose the chance to obtain a model with optimal predictive performance using the same predictors. Meanwhile, a unique interaction should exist between a set of predictors and a pregnancy outcome. A particular predictive algorithm may work best to capture this predictor-outcome interaction. Prediction tasks are even more challenging in pregnancy care because they demand more prognostic instead of diagnostic predictions. Yet, unlike the common nature of other long-term conditions in health care (eg, diabetes mellitus), the onset, time to event, and target population in pregnancy care are rather apparent. However, unpredictable events leading to disabilities and death in a population such as pregnant women or newborns are also not easily accepted as in other populations (eg, patients with cancer and older adults). Thus, clinicians should apply several prediction models with satisfactory predictive performances throughout the pregnancy period. Clinicians and investigators would benefit from knowing whether an LR or other algorithms have a better chance of achieving satisfactory predictive performances for a particular pregnancy outcome. However, no previous systematic review in pregnancy care has reviewed multiple machine learning algorithms and compared their predictive performances, including LR, to predict pregnancy outcomes.

This review will allow investigators and clinicians in pregnancy care to consider the development or application of prediction models throughout the pregnancy period. This review demonstrates which algorithms have shown robust predictive performances for a particular pregnancy outcome using a similar set of predictors. Investigators in pregnancy care may also consider whether a reanalysis by another predictive algorithm is needed by using existing data previously analyzed by an algorithm including LR. Beyond the algorithm issue, the development of machine learning models also requires an adequate methodology and interpretable results [15]. Biased conclusions should be avoided when describing machine learning predictive performances [11,16]. Standard guidelines are important when investigating and reviewing machine learning applications in clinical prediction modeling [15,17].

### Objectives

By applying the standard guidelines, we aim to review machine learning models and compare their predictive performances between LRs and other machine learning algorithms. In this review, we focus on machine learning models either developed or validated for making prognostic predictions in pregnancy care intended to inform clinicians’ decision making.

## Methods

### Protocol and Registration

We reported this study based on PRISMA (Preferred Reporting Items for Systematic Reviews and Meta-Analyses) guidelines [18] and conducted the review based on several guidelines related to prediction studies. The review objective was defined according to a standard of key items [19]. Our eligibility criteria were composed of items elaborated with 2 guidelines for developing and reporting a prediction model and a guideline for assessing the applicability. These included transparent reporting of a multivariable prediction model for individual prognosis or diagnosis (TRIPOD) [20] and another that focuses on machine learning modeling in biomedical research (hereafter referred to as guidelines for developing and reporting machine learning predictive models in biomedical research [MLP-BIOM]) [15]. Applicability was assessed using assessments that were a part of the *prediction model risk of bias assessment tool* (PROBAST) [17,21]. Data were extracted based on the checklist for critical appraisal and data extraction for systematic reviews of prediction modeling studies (CHARMS), which also describes items for the review objective. Our review protocol was registered with PROSPERO (CRD42019136106).

### Eligibility Criteria

Before defining the eligibility criteria, we decided to view the LR as one of many algorithms in the machine learning field with respect to its use in statistics and data science. A prediction model development consisted of several elements: predictor selection, parameter fitting, and hyperparameter optimization [2]. In this review, the term *prediction model* refers to all those elements, whereas the term *prediction algorithm* refers to a parameter-fitting method. Using the same set of predictors, we would expect different predictive performances if the parameters of a model are fitted using different algorithms. A prediction algorithm in machine learning is a way for the computer to learn from data by fitting the parameters with respect to predicting a class measured by hyperparameters from the human user [22]. Several optimization algorithms have been developed to reduce the human role in determining these hyperparameters, such as sequential search, random search, and Bayesian optimization [23]. However, that is beyond the scope of this review.

By focusing on prediction algorithms, we defined eligibility criteria to screen studies by the title, abstract, and full text. We also assessed the applicability by examining the full text. These were the candidates we selected for the qualitative analysis. Key items of population, index, comparator, outcomes, timing, setting (PICOTS) [19] and additional items [15,20] composed the eligibility criteria. The first item of these criteria was a review question framed using PICOTS. The key items consisted of the following:

1. Population: men or women in procreative management, pregnant women, and fetuses or newborns.
2. Index: multivariable prognostic prediction models applying non-LR algorithms for risk classification tasks intended to inform clinicians’ decision making.
3. Comparator: multivariable prognostic predictions applying an LR algorithm, excluding a scoring system in which the parameters determined by humans instead of using LR, for risk classification tasks intended to inform clinicians’ decision making.
4. Outcomes: pregnancy-related outcomes of procreative management or pregnancy outcomes for pregnant women or fetuses or newborns.
5. Timing: with predictors being measured at the pre-, inter-, and peripregnancy periods and outcomes being assessed at the pregnancy, delivery, and either puerperal or neonatal period, short- and long-term prognoses were applied.
6. Setting: primary care or hospital.

Additional items were the availability of several reporting components as required by TRIPOD and MLP-BIOM. These components included (1) data sources, (2) outcomes, (3) evaluation metrics, (4) predictors, (5) descriptive statistics, (6) event sample sizes, (7) modeling methods or algorithms, and (8) model validation.

After briefly screening studies by eligibility criteria, we conducted an applicability assessment by thoroughly examining the full texts. Using PROBAST guidelines, we assessed the applicability according to the review question framed by PICOTS. Low, high, or unclear criteria were determined for applicable, not applicable, or unclear applicability, respectively. The assessment covered 3 domains of participants, predictors, and outcomes. Only those fulfilling *low* criteria were selected for the qualitative analysis.

For the quantitative analysis, studies had to report the AUROC. Studies were selected from those applicable for the qualitative analysis. If there were at least three LR models and a non-LR model from any studies for an outcome, all studies with that outcome were included in the meta-analysis. This was determined based on the requirement of a minimum number of data points to calculate the variance as part of the meta-analytical procedure. If studies did not report the AUROC, we estimated the sensitivity and specificity using the trapezoidal rule (see *Summary Measures* and *Synthesis of Results* sections).

### Information Sources

We searched the MEDLINE, Scopus, Web of Science, and Google Scholar databases up to May 2020. There was no limit on the publication period. However, considering the limitations of the search interface in Google Scholar, we only retrieved results from the last year with keywords in the abstract or the entire period with those keywords in the title. We also limited the publication period to the last 10 years for search results by keywords including “logistic regression multivariable prediction.” This was because we estimated that there would be enormous amounts of studies applying LR because we applied a broad range of outcomes in this study. In contrast, we might lack studies using other machine learning models, although the outcomes were broad.

### Search

The initial search filter was limited to the title, abstract, keywords, or Medical Subject Heading (MeSH; MEDLINE only) using “machine learning” AND pregnancy. We also used “machine learning AND ([pregnancy outcome from initial search] NOT pregnancy).” Keywords for pregnancy outcomes were used based on MeSH to generalize a variety of terms for pregnancy outcomes from selected studies. If the MeSH term contained “pregnancy,” then we used the alternative entry terms in the webpage recorded for this MeSH term. If all entry terms also contained “pregnancy,” then we used the term without negating “pregnancy.” In addition, we also substituted the “machine learning” part with one of the keywords consisting of “decision tree,” “artificial neural network,” “support vector machine,” “random forest,” “artificial intelligence,” “deep learning,” and “logistic regression multivariable prediction.” All keywords are described in Multimedia Appendix 1. These search terms were applied to all databases.

### Study Selection

Duplicate records from multiple databases were removed. We refined the search results in the title or abstract using EndNote X8 (Clarivate Analytics) by “(supervised NOT unsupervised) OR prediction OR classification.” Records were screened by HS and AH, and the results were assessed by HS, AH, YC, CK, OS, TY, and YW. Disagreements were resolved by discussion with the last author (ES). Study selection was conducted in brief and thorough assessments. These brief assessments were intended to select studies by checking eligibility criteria from TRIPOD and MLP-BIOM in the title, abstract, and briefly in the full-text article. A thorough assessment of the applicability from PROBAST was conducted later before the ROB assessment.

### Data Collection Process

We extracted data based on the CHARMS checklist, which includes (1) outcomes, (2) study design, (3) data sources, (4) data source design, (5) setting, (6) type of study, and (7) modeling methods or algorithms, and (8) predictive performance. Outcomes were pooled as distinct MeSH terms. Study and data source designs were classified into prospective, retrospective, nested case-control, case-control, and cross-sectional. We defined the type of study based on the model validation, which might be development, validation, or both. Eligible studies were described as developing prediction models by applying LR, non-LR, or both algorithms. Predictive performances were only taken from studies that were eligible for the meta-analysis (see *Eligibility Criteria* section). If there were multiple models developed within a study using the same algorithm, we retrieved the AUROC from the best performing one among the models. If both LR and non-LR algorithms were applied in a study, we selected the predictive performances of the best models applying either the LR or non-LR algorithm. Model performances derived from external validation were preferred if available.

### ROB Within and Across Studies

We used PROBAST to assess the ROB [17,21]. The ROB in individual studies was assessed as low, high, or unclear in 4 domains of participants, predictors, outcomes, and analyses. In addition, 20 signaling questions were answered for each study in a transparent and accountable form. Across studies, we described the proportion of low, unclear, or high ROBs. ROBs were compared for each domain. We also summarized the answers for each signaling question.

### Summary Measures

We compared AUROCs from studies that reported this metric. Logit transformation was applied to the AUROCs. We computed logit AUROC differences between each non-LR and LR algorithm across studies. Summary measures from any eligible studies with all, low, or high ROB were pooled by random effects modeling, as previously described [24]. Assuming that selected studies were random samples from a larger population, we chose a random effects model that attempted to generalize findings beyond the included studies using that assumption [25]. Despite this, we did not conduct random effects modeling for all selected studies considering the broad range of target populations, outcomes, and algorithms. Meanwhile, we conducted this review within a narrower field compared with a previous systematic review of machine learning in medicine [13]. Therefore, we only applied random effects modeling to the predictive performances of selected studies using a particular pregnancy outcome. These studies consisted of a minimum number of non-LR and 3 LR models from any studies. This minimum number was considered to obtain a minimum number of data points of logit AUROCs to compute the interval estimates in a random effects model. We depicted the AUROCs using forest plots; thus, one can see which prediction algorithm may have a better chance of obtaining optimal predictive performance for a particular pregnancy outcome.

Pooled estimates of pairwise differences in logit AUROCs were described by points and the 95% CI [26]. A positive difference in logit AUROCs means that the non-LR algorithm had a higher logit AUROC than that of the LR algorithm. The difference was significant if 0 was not included within the 95% CI. The number of pairwise comparisons (*k*) for each random effects model was reported. We also reported variance across studies (*τ^2^*) and *I^2^* as absolute and relative values of between-study heterogeneity, respectively.

If a study did not report the AUROC, we estimated this metric based on sensitivity and specificity. As a specificity of 0% means a sensitivity of 100% and *vice versa*, the AUROC could be estimated from the reported sensitivity and specificity using a common rule to calculate the area of the trapezoid (Equation 1). Before we subtracted the AUROC of a non-LR algorithm from that of an LR algorithm, we applied a logit transformation (Equation 2).

AUROC = 0.5 × (1 − specificity) × sensitivity + specificity × sensitivity + 0.5 × (1 − sensitivity) × specificity **(1)**

Logit(AUROC) = log (AUROC / (1 – AUROC)) **(2)**

We used RStudio 1.2 (RStudio) with R 3.6.1 and an additional package, metafor 2.4.0, for random effects modeling. We applied the restricted maximum likelihood estimator method [27]. These are common tools and recommended modeling methods for meta-analyses [28].

### Synthesis of Results

We described the characteristics of the studies consisting of population, study design, timing, and setting. This was described as the number of algorithms used for prediction modeling. The algorithms were categorized into LR, non-LR, or both algorithms. We also show the proportion of each characteristic compared with all characteristics within the same algorithm category.

ROBs within studies were described for the number of low, high, or unclear ROB studies. This was reported for overall assessment results and by domain in studies that used LR, non-LR, or both algorithms. ROBs across studies were described for the proportion of studies in which the answer to each signaling question led to low, high, or unclear ROB studies. We intended to show what makes most studies considered to have high ROBs.

Meta-analytical results were described by a forest plot faceted by outcome. Each facet showed comparisons of differences in logit AUROCs for each random effects model of non-LR versus LR algorithms. This demonstrated which algorithms tended to outperform LR for each pregnancy outcome. Comparisons that included non-LR high ROB studies were color coded. The best predictive performance for each outcome was reported. Between-study heterogeneity for each random effects model was also reported.

We described predictors in the prediction models from studies in the meta-analysis. For each outcome in the meta-analysis, we selected only random effects models in which an algorithm significantly outperformed the other. This was determined by the 95% CI of the difference in logit AUROCs between a non-LR and an LR model for an outcome. If any, we only selected those that included only non-LR low ROB studies. Only predictors in the final model were included. This was intended to elucidate predictor-outcome interactions that characterized an algorithm if it outperformed the others for a particular outcome.

## Results

### Study Selection

**Figure 1:**
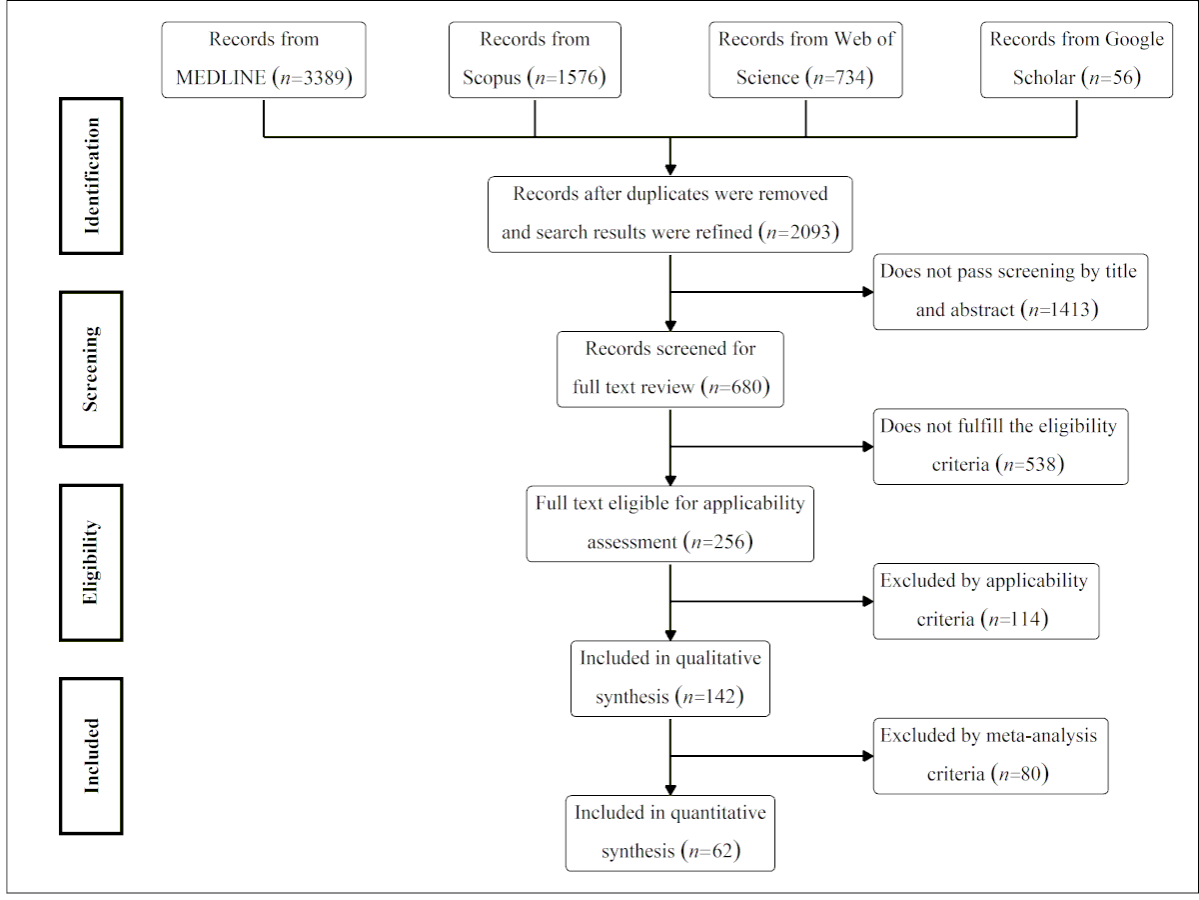
Study selection workflow.

We found 2093 records from 4 literature databases (Figure 1). The search filters consisted of 144 combinations of keywords from 8 machine learning terms and 18 MeSH terms for pregnancy outcomes recursively derived from the keywords “machine learning AND pregnancy” (Multimedia Appendix 1). We refined the search results, identified research articles (not including conference abstracts or theses), and removed duplicates. After screening and eligibility assessment, we included 142 studies for the qualitative analysis, of which 62 were used for the quantitative analysis. A detailed description of the eligible criteria, process of study selection, and list of studies for the full-text review are given in Multimedia Appendix 1.

### Characteristics of the Studies

Briefly, we collected studies that either developed or validated a prediction model applying either LR (77/142, 54.2%) [29-105] or non-LR machine learning algorithms (50/142, 35.2%; Table 1) [106-155]. Overall, 15 studies applied both LR and non-LR algorithms (15/142, 10.6%) [156-170]. The cohort population of the studies in this review consisted of every type of population, study design, timing, and setting that we desired to discuss in this review. More studies discussed fetuses or newborns than pregnant women in non-LR prediction studies (26/50, 52% vs 11/50, 22%). Meanwhile, the opposite occurred in LR studies that focused more on pregnant women than fetuses or newborns (50/77, 65% vs 19/77, 25%). Most used data sets were from retrospective cohorts for LR (53/77, 69%) [29-36,38-42,47,49-54,56-61,64-66,68-71,76-80,83,87-90,92,94,95, 97,100-105], non-LR prediction studies (27/50, 54%) [107,108,111-113,116,117,121,122,127,130-138,140,142,143,148,150,151, 153,154], or both (9/15, 60%) [157-160,163-165,167-170]. A retrospective cohort is one of the recommended study designs for prognostic purposes instead of diagnostic prediction [21]. This corresponds to our review question that warrants prognostic predictions in pregnancy care intended to inform clinicians’ decision making.

Only a few studies had prediction timing up to the puerperal or neonatal period for LR (2/77, 3%) [74,85], non-LR (3/50, 6%) [114,129,149], or both algorithms (2/15, 13%) [162,168]. This is because some predictors were assessed after delivery, whereas our review question demanded those be assessed up to delivery. We also considered studies using data sets from either primary care or hospital settings because the data are applicable for clinicians’ decision making on a daily basis. As applicability was already included in the eligibility assessment before the qualitative analysis, eligible studies were not found to use data sets from either primary care or hospital settings, such as from a house-to-house survey or a screening program. Most used data sets were from hospital settings, whereas only a few of those were from primary care settings in the LR (6/77, 8%) [65,69,73,77,78,87], non-LR (6/50, 12%) [119,122,132,135,148,153], or both algorithms (1/15, 7%) [162]. A detailed description of this is also given in Multimedia Appendix 1.

**Table 1:** Characteristics of eligible studies.

Variable		Number of studies (percentage based on column total)			
		LR^a^ (n=77), n (%)	Non-LR (n=50), n (%)	Both (n=15), n (%)	Total (n=142), n (%)
**Population**					
	Pregnant women	50 (65)	11 (22)	6 (40)	67 (47.2)
	Fetuses or newborns	19 (25)	26 (52)	7 (47)	52 (36.6)
	Men or women in procreative management	8 (10)	13 (26)	2 (13)	23 (16.2)
**Study design**					
	Retrospective	53 (69)	27 (54)	9 (60)	89 (62.7)
	Nested case-control	4 (5)	14 (28)	2 (13)	20 (14.1)
	Prospective	13 (17)	4 (8)	0 (0)	17 (12)
	Cross-sectional	3 (4)	3 (6)	3 (20)	9 (6.3)
	Case-control	4 (5)	2 (4)	1 (7)	7 (4.9)
**Timing**					
	At delivery	28 (36)	26 (52)	7 (46.7)	61 (42.9)
	At pregnancy	34 (44)	21 (42)	5 (33.3)	60 (42.3)
	Mixed timing	13 (17)	0 (0)	1 (6.7)	14 (9.9)
	Puerperal or neonatal period	2 (3)	3 (6)	2 (13.3)	7 (4.9)
**Setting**					
	Hospital	61 (79)	43 (86)	9 (60)	113 (79.6)
	Both	10 (13)	1 (2)	5 (33)	16 (11.3)
	Primary care	6 (8)	6 (12)	1 (7)	13 (9.2)

^a^LR: logistic regression.

### LR and Other Machine Learning Algorithms

Most studies applied an LR (92/142, 64.8%) to develop a prediction model (Table 2). Meanwhile, an artificial neural network was mostly applied by non-LR studies (20/142, 14.1%). Studies that applied LR and non-LR algorithms mostly compared LR with an artificial neural network (5/15, 33%) [161,163,165,166,170] and decision tree (5/15, 33%) [156,159,167-169], but decision trees tended to be paired with an LR compared with an artificial neural network (5/7, 71% vs 5/20, 25%).

The characteristics of study populations showed that pregnant women and fetuses or newborns were the populations of most studies developed using LR and non-LR models, respectively. Among pregnant women, the LR algorithm was mostly applied to develop predictions for outcome categories of obstetric labor (13/77, 17%) [36,46,47,54,57,62,64,70,83,86,91,97,103], pregnancy-induced hypertension (12/77, 16%) [30,31, 43,48,55,65,66,68,76,81,93,105], and gestational diabetes (7/77, 9%) [33,45,49,84,94,100,104]. Among fetus or newborn populations, non-LR algorithms were mostly applied to develop predictions for outcome categories of premature birth (12/50, 24%) [111,112,115,116,118,119,121,122,125,130,141,143] and fetal distress (9/50, 18%) [113,124,128,137,138,145,146, 152,155]. In addition, more non-LR algorithms (13/20, 65%) were applied for the outcome category of *in vitro* fertilization than for the LR algorithm.

**Table 2:** Machine learning algorithm and category of outcome.

Variable		Number of studies (percentage based on column total)			
		LR^a^ (n=77), n (%)	Non-LR (n=50), n (%)	Both (n=15), n (%)	Total (n=142), n (%)
**Machine learning algorithm**					
	Logistic regression	77 (100)	N/A^b^	15 (100)	92 (64.8)
	Artificial neural network	N/A	15 (30)	5 (33)	20 (14.1)
	Support vector machine	N/A	9 (18)	1 (7)	10 (7.0)
	Deep neural network	N/A	8 (16)	1 (7)	9 (6.3)
	Random forest	N/A	7 (14)	1 (7)	8 (5.6)
	Decision tree	N/A	2 (4)	5 (33)	7 (4.9)
	Gradient boosting	N/A	3 (6)	2 (13)	5 (3.5)
	Naïve Bayes	N/A	4 (8)	0 (0)	4 (2.8)
	Ensemble of algorithms	N/A	2 (4)	0 (0)	2 (1.4)
**Category of outcome**					
	Premature birth	9 (12)	12 (24)	3 (20)	24 (16.9)
	In vitro fertilization	7 (9)	13 (26)	2 (13)	22 (15.5)
	Obstetric labor	13 (17)	1 (2)	2 (13)	16 (11.3)
	Pregnancy-induced hypertension	12 (16)	4 (8)	0 (0)	16 (11.3)
	Fetal distress	1 (1)	9 (18)	0 (0)	10 (7.0)
	Gestational diabetes	7 (9)	2 (4)	1 (7)	10 (7.0)
	Cesarean section	4 (5)	3 (6)	2 (13)	9 (6.3)
	Fetal development	4 (5)	1 (2)	0 (0)	5 (3.5)
	Small-for-gestational-age infant	3 (4)	1 (2)	1 (7)	5 (3.5)
	Others	17 (22)	4 (8)	4 (27)	25 (17.6)

^a^LR: logistic regression.

^b^N/A: not applicable.

### ROB Within and Across Studies

ROB is described for each eligible study in Multimedia Appendix 1 [29-170]. Among the 142 eligible studies, there were 24 (16.9%) low ROB studies [38,61-63,71,98,104,110,113,115,117-119,128,134,141,142,145,147,149, 155,157,158,169], 117 (82.4%) high ROB studies [29-37,39-60,64-70,72-97,99-103,105-109,111,112,114,116,120-123, 125-127,129-133,135-140,143,144,146,148, 150-154,156,159-168,170], and 1 (0.7%) unclear ROB study (Table 3) [124]. Among the low ROB studies, the categories of outcomes were premature birth (7/24, 30%) [38,63,115,118,119,141,169], fetal distress (5/24, 21%) [71,113,128,145,155], *in vitro* fertilization (4/24, 17%) [61,110,134,158], gestational diabetes (2/24, 8%) [104,157], cesarean section (CS; 2/24, 8%) [117,142], obstetric labor (1/24, 4%) [62], pregnancy-induced hypertension (1/24, 4%) [147], central nervous system malformations (1/24, 4%) [149], and others (1/24, 4%) [98].

**Table 3:** Risk of bias within studies.

Assessment by domain		Studies by algorithm			
		LR^a^ (n=77), n (%)	Non-LR (n=50), n (%)	Both (n=15), n (%)	Total (n=142), n (%)
**Participants**					
	Low	60 (78)	44 (88)	11 (73)	115 (80.9)
	High	15 (19)	3 (6)	4 (27)	22 (15.5)
	Unclear	2 (3)	3 (6)	0 (0)	5 (3.5)
**Predictors**					
	Low	54 (70)	43 (86)	12 (80)	109 (76.8)
	High	20 (26)	5 (10)	1 (7)	26 (18.3)
	Unclear	3 (4)	2 (4)	2 (13)	7 (4.9)
**Outcome**					
	Low	51 (66)	40 (80)	11 (74)	102 (71.8)
	High	24 (31)	4 (8)	2 (13)	30 (21.1)
	Unclear	2 (3)	6 (12)	2 (13)	10 (7.1)
**Analysis**					
	Low	8 (10)	15 (30)	3 (20)	26 (18.3)
	High	69 (90)	35 (70)	12 (80)	116 (81.7)
	Unclear	0 (0)	0 (0)	0 (0)	0 (0.0)
**Overall**					
	Low	7 (9)	14 (28)	3 (20)	24 (16.9)
	High	70 (91)	35 (70)	12 (80)	117 (82.4)
	Unclear	0 (0)	1 (2)	0 (0)	1 (0.7)

^a^LR: logistic regression.

**Figure 2:**
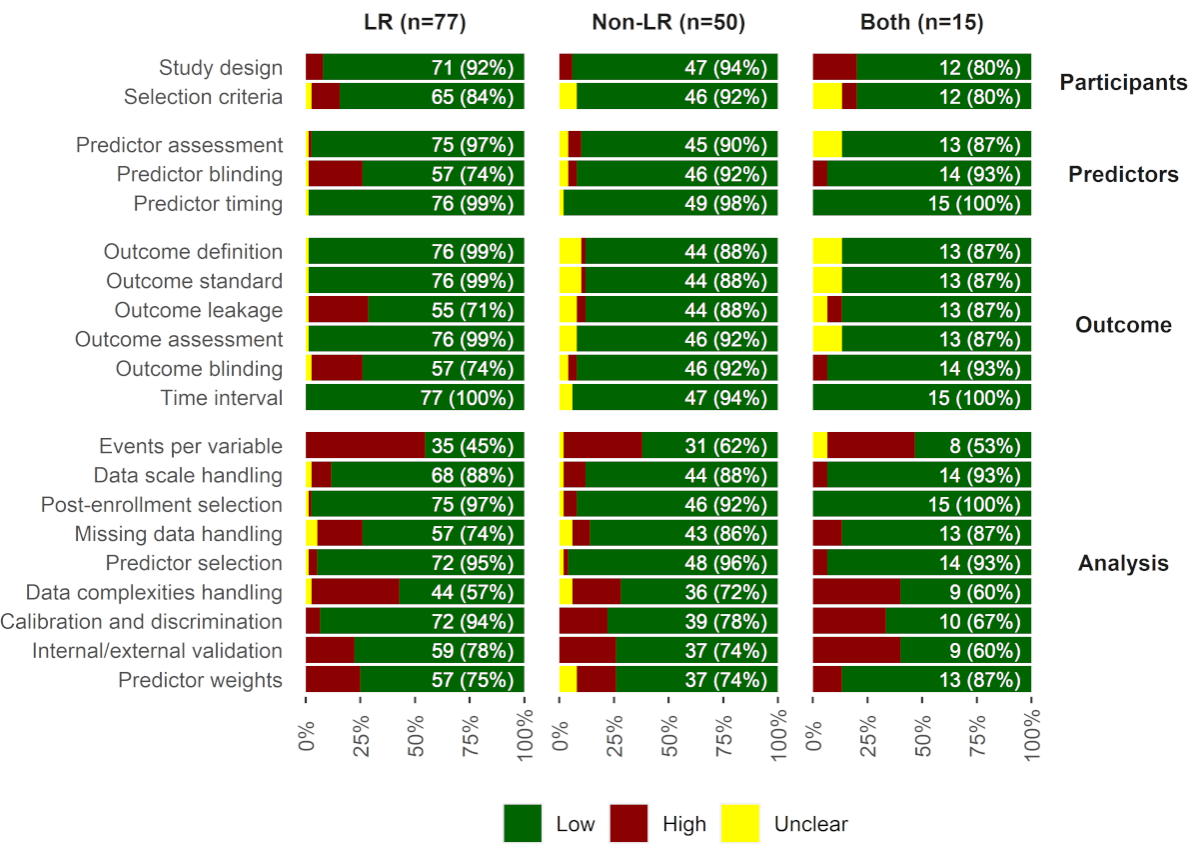
Signaling questions with respect to ROB domains across studies. Bars from low/high/unclear ROB are stacked to be 100%. Domains are described on the right-hand side. The number on the bar is the number of low ROB studies (total LR/non-LR/both at top) based on a single signaling question summarized as a term on the left-hand side. LR: logistic regression; ROB: risk of bias.

ROB is also described across the studies in Table 3 and Figure 2. The corresponding signaling questions for each term and the answers for each study are described in Multimedia Appendix 1. Low ROB studies were the fewest in the analysis domain (26/142, 18.3%), consisted of the LR (8/77, 10%) [38,61-64, 71,96,98,104], non-LR (15/50, 30%) [63,110,113,115, 117-119,124,128,134,141,142,145,147,155], and both algorithms (3/15, 20%) [157,158,169]. In the analysis domain, the fewest low ROB studies that achieved the minimum events per variable (EPV) consisted of LR (35/77, 45%) and non-LR (31/50, 62%) prediction studies. More calibration and discrimination tests were conducted using LR (72/77, 94%) than by non-LR (39/50, 78%) prediction studies. In contrast, more non-LR prediction studies appropriately handled missing data (43/50, 86%) compared with LR prediction studies (57/77, 74%).

### Comparison of the Predictive Performance

**Figure 3:**
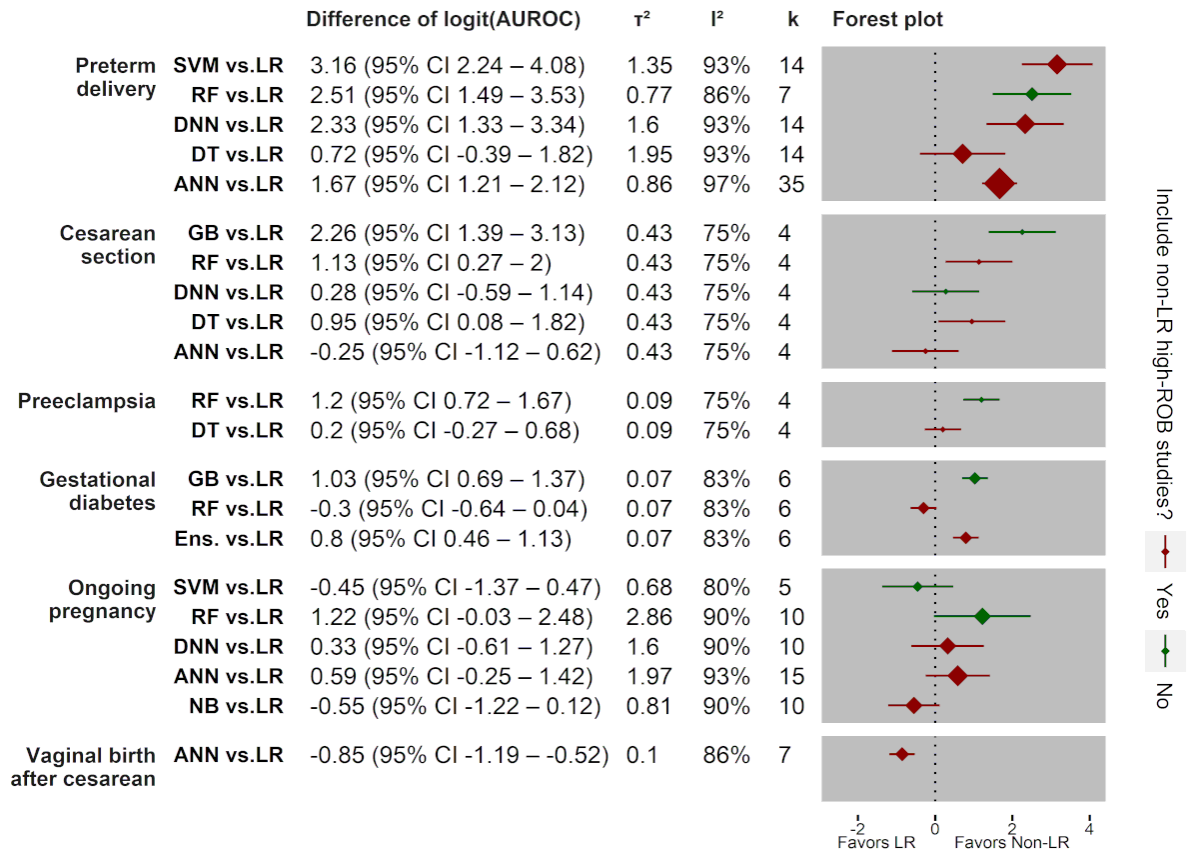
Forest plot of random effects models for differences in logit AUROCs from a non-LR with any LR prediction models. Plots were grouped by outcome. The lines indicate the 95% CI with diamonds whose sizes were determined by the number of pairwise comparisons (k). Absolute and relative values of between-study heterogeneities are denoted by τ2 and I2, respectively. Colors of the boxes and lines were determined based on the existence of high ROB studies among those using non-LR algorithms. ANN: artificial neural network; AUROC: area under the receiver operating characteristic curve; DNN: deep neural network; DT: decision tree; Ens: ensemble of multiple algorithms; GB: gradient boosting; LR: logistic regression; NB: naïve Bayes; RF: random forest; ROB: risk of bias; SVM: support vector machine.

There were 62 studies in the meta-analysis that had outcomes that were predicted by at least one non-LR and 3 LR models (see *Summary Measures* section). Overall, 21 random effects models of the predictive performance by non-LR versus LR models are shown in a forest plot (Figure 3). Forest plots of logit AUROC differences for each random effects model are described (Multimedia Appendix 1). With respect to candidate studies (n) included in the final random effects models, we developed 5 random effects models for preterm delivery (20/62, 32%) [32,44,60,63,75,87,96,111,112,115,118,119,121,125,130, 141,143,156,163,169], 5 for CS (7/62,11%) [79,90, 106,117,142,166,167], 2 for pre-eclampsia (6/62, 10%) [31,48,65,76,123,147], 3 for gestational diabetes (9/62, 15%) [33,45,84,94,100,104,108,139,157], 5 for ongoing pregnancy (13/62, 21%) [73,78,99,110,132,134-136,148,150,153,158,170], and 1 for vaginal birth after CS (7/62, 11%) [36,47,57, 64,83,97,165].

To determine the final random effects model for each comparison, we identified studies that were responsible as the source of heterogeneity and removed those AUROCs from the random effects model. We excluded a non-LR [121] and an LR study [84] that developed a prediction model for preterm delivery and gestational diabetes, respectively. This is because their AUROCs were outliers compared with those for the same outcome and algorithm. We also excluded 3 LR studies [32,63,87]. In those studies, preterm delivery was defined as delivering within 1 to 2 weeks of preterm labor presentation. Meanwhile, the majority of studies for this outcome defined preterm delivery as that before 37 weeks of gestation.

The non-LR models significantly outperformed the LR models in preterm delivery (4/5 non-LR models), CS (3/5 non-LR models), pre-eclampsia (1/2 non-LR models), and gestational diabetes (2/3 non-LR models). From those that examined preterm delivery, a prediction model did not include a non-LR high ROB study [115] compared with those from 7 LR studies [32,44,60,63,75,87,96]. This model applied a random forest (differences in logit AUROC 2.51; 95% CI 1.49-3.53). The same algorithm was applied to a prediction model from a non-LR low ROB study in pre-eclampsia [147]. For random effects modeling, this model also significantly outperformed those from 4 LR studies (1.2, 95% CI 0.72-1.67) [31,48,65,76]. Meanwhile, prediction models from non-LR low ROB studies of Saleem et al [142] and Artzi et al [157] significantly outperformed those from the corresponding LR studies as an aggregate for CS (2.26, 95% CI 1.39-3.13) and gestational diabetes (1.03, 95% CI 0.69-1.37). Interestingly, the models were developed using a gradient boosting algorithm that used multiple decision trees similar to a random forest.

In contrast, a prediction model using a non-LR algorithm significantly underperformed compared with those using an LR in a random effects model (−0.85, 95% CI −1.19 to −0.52). This applied an artificial neural network to predict vaginal birth after a CS [165]. This model underperformed compared with those from 7 LR studies [36,47,57,64,83,97,165]. However, the non-LR study was a high ROB study.

A random effects model developed for comparison of artificial neural networks and LR to predict preterm delivery had the highest heterogeneity by *I^2^* (97%; *k*=35). This number means that 97% of the total variability among 35 data points of differences in logit AUROCs was caused by between-studies heterogeneity instead of sampling error within each study [171]. This is reasonable because a higher variance occurs with a larger number of comparisons within a random effects model. In contrast, a random effects model with the smallest number of comparisons (*k*=4) also had the lowest heterogeneity by *I^2^* (75%). This random effects model was developed to analyze comparisons of non-LR and LR algorithms for either CS or pre-eclampsia. Nevertheless, a diverse target population and hyperparameter optimization conceivably caused the heterogeneity of the predictive performance, although the same outcome was predicted using the same data set and machine learning algorithm. The lowest *I^2^* in this meta-analysis remains classified as substantial heterogeneity instead of moderate or unimportant; thus, performing random effects instead of fixed effect modeling is recommended to address this issue [172].

However, *I^2^* only indicates that the difference in logit AUROCs substantively varies across studies but does not tell how much this metric varies [173]. To interpret the absolute heterogeneity for the difference in logit AUROCs, we needed to consider the observed AUROC of a non-LR model for each of the random effects models. The observed AUROCs were described for each of the original studies in this meta-analysis in Multimedia Appendix 1.

A random effects model developed for comparison of random forests and LR to predict ongoing pregnancy had the highest absolute value of heterogeneity (*τ^2^*=2.86). In this random effects model, random forests were applied to develop predictions in 2 studies that reported AUROCs of 0.740 (95% CI 0.710-0.770) [158] and 0.9820 [134]. We simulated a sequence of logit AUROCs to identify equivalent differences in AUROCs to approximate a difference of the logit value in the random effects model (1.22, 95% CI −0.03 to 2.48). AUROC differences of 0.206 and 0.026 were equivalent to a difference in the logit AUROC of 0.91, compared with those aggregated from LR models for the random forest models of Blank et al [158] and Mirroshandel et al [134], respectively. Using *τ^2^*, one can calculate the 95% prediction interval (PI) of the logit AUROC difference, as previously described [173]. This estimates the potential AUROC of the random forest to predict ongoing pregnancy with respect to an LR using different populations. For this random effects model, the 95% PI of the logit AUROC difference ranged from −4.75 to 7.19. This is equivalent to 0.257 lower and >0.73 higher than AUROCs of any LRs in the random effects model for the random forest model of Blank et al [158]. For the random forest model of Mirroshandel et al [134], the 95% PI was equivalent to 0.018 lower and 0.943 higher than the AUROCs of any LRs in the random effects model. This is a reasonably wide PI for the highest *τ^2^* in this meta-analysis, although the non-LR study had a low ROB. This is because ROB only reflects the risk of a predictive performance that differs from the true value of the training sample. However, the ROB does not reflect the difference if the predictive performance is compared with other samples across different populations.

For the random effects model with the lowest *τ^2^* and including a non-LR low ROB study, the random effects model had a logit AUROC difference of 1.03 (95% CI 0.69-1.37) for a prediction model of gestational diabetes using gradient boosting. The prediction study reported an AUROC of 0.875 (95% CI 0.868-0.885) [157]. The 95% PI of the logit AUROC difference estimated an equivalent AUROC that ranged from 0.0096 lower to 0.425 higher than the AUROCs of any LR in the random effects model. The gradient boosting model from this study is likely to outperform an LR to predict gestational diabetes.

In addition, we may need to know the *τ^2^* meaning for the random effects model with the highest *I^2^* and larger numbers of comparisons (*k*). This random effects model had an AUROC difference of 1.67 (95% CI 1.21-1.94; 95% PI −2.08 to 5.42; *k*=35) for a prediction model of preterm delivery using an artificial neural network. Overall, 5 non-LR studies were included in this random effects model. The remaining studies reported AUROCs of 0.88 [111], 0.94 [118], 0.945 [125], 0.9115 [163], and 0.911 (95% CI 0.862-0.960) [130]. Considering only the lowest (0.862) and highest (0.960) that covered all of the AUROCs, the artificial neural network model may have AUROCs of 0.119 lower and 0.864 higher than those of any LR. The AUROC interval was also as wide as that of the random effects model with the highest *τ^2^*.

### Descriptive Analysis of Predictors

A random effects model was selected for each outcome except for ongoing pregnancy, which fulfilled our criteria to describe the predictors. For each outcome in the meta-analysis, we selected random effects models in which either a non-LR algorithm significantly outperformed the LR or it was significantly underperformed by the LR. This was determined by the 95% CI of the difference in the logit AUROCs between the non-LR and LR models for an outcome. If any, we only selected those including only non-LR low ROB studies. The random effects models were random forest versus LR for preterm delivery, gradient boosting versus LR for CS, random forest versus LR for pre-eclampsia, gradient boosting versus LR for gestational diabetes, and artificial neural network versus LR for vaginal birth after a CS. As we only extracted the AUROC of either the best LR or non-LR model, only predictors and outcomes of that model were considered if there were multiple models for different subtypes of the outcome in a study.

For preterm delivery, Despotovic et al [115] developed a random forest model using a previously published standardized electrohysterogram (EHG) data set [174]. This data set was also used by other studies in this meta-analysis to predict the same outcome using different algorithms [118,125,130,141,143,169]. All predictors were features extracted from the multichannel EHG obtained at around 22 and 32 weeks of gestation to predict delivery after 39 and 34 to <37 weeks of gestation for term and preterm delivery, respectively. Compared with their counterparts, LR models used predictors consisting of maternal demographics or lifestyle [44,60,75,96,163], medical or obstetric histories [44,75,96,156,163], clinical predictors from obstetrical examinations [44,163], EHG [169], and biomarkers [75]. These were obtained before pregnancy [60,96,156,163], at 11 to 14 weeks of gestation [75], 18 to 34 weeks of gestation [44,163,169], or near events within 1 to 2 weeks [44]. The LR models were developed to predict preterm delivery at 20 to <37 weeks of gestation [44,75,96,163,169] and any delivery at <37 weeks of gestation (predictors could be taken before pregnancy) [60,156].

For CS, Saleem et al [142] developed a gradient boosting model using a previously published standardized cardiotocogram (CTG) data set [175]. This data set was also used by Fergus et al [117] in this meta-analysis to predict the same outcome using a deep neural network. All predictors were features extracted from the CTG data set obtained at first- and second-stage labor for a maximum of 90 min preceding delivery to predict a CS. Compared with their counterparts, LR models used predictors consisting of maternal characteristics [79,90,166], medical histories [167], obstetric histories [90,166,167], and clinical predictors from obstetric examinations [90,166,167], ultrasound measures [79], routine laboratory tests [90], and medications [90]. These were obtained before [90,166,167] and during pregnancy [79,90,166,167]. The LR models were developed to predict CS [166,167], emergency CS [79], and CS in pregnant women with gestational hypertension or mild pre-eclampsia at term [90].

For pre-eclampsia, Sufriyana et al [147] developed a random forest model that used a nationwide health insurance data set. The predictors consisted of maternal demographics and medical histories but excluded obstetric ones. These were obtained before and during pregnancy up to 2 days before the events (pre-eclampsia or eclampsia of any severity and timing). Meanwhile, the LR counterparts used maternal demographics or lifestyle [31,65,76], medical histories [31,65,76], obstetric histories [31,65,76], family histories [31,76], clinical or obstetric examinations [31,65], ultrasound measures [65], routine laboratory tests [76], and biomarkers [48,65]. These predictors were obtained before pregnancy [31], at 11 to 13 weeks of gestation [65], and at <20 weeks of gestation [48]. LR models were developed to predict pre-eclampsia of any severity and timing [31,48,65,76]. The predictors were taken before pregnancy, and this disorder occurs after 20 weeks of gestation by definition.

For gestational diabetes, Artzi et al [157] developed a gradient boosting model that used a nongovernmental, nationwide health care database. The predictors consisted of maternal demographics, medical histories, obstetric histories, clinical or obstetric examinations, routine laboratory tests, and medications. These predictors were obtained before pregnancy and up to 22 weeks of gestation to predict gestational diabetes diagnosed at 24 to 28 weeks of gestation. The LR counterparts used maternal demographics or lifestyle [33,100,104], medical histories [33], obstetric histories [104], family histories [33,45], clinical examinations [33], obstetric examinations [33], routine laboratory tests [33,45,94,100,104], medications, and biomarkers [33,45]. The predictor timing was 6 to 14 weeks of gestation [33,45,94,100,104] and >14 to 22 weeks of gestation [45,100,104]. Meanwhile, the outcome timing was 24 to 28 weeks of gestation [33,45,94,100,104].

For vaginal birth after a CS, Macones et al [165] developed an artificial neural network model that used a medical records database. The predictors used maternal characteristics, medical histories, obstetric histories, obstetric examinations, and labor procedures. These were obtained before pregnancy, during pregnancy, and at labor to predict successful vaginal birth after a CS. The LR counterparts used maternal characteristics [36,47,64,83,97], medical histories [57], obstetric histories [36,47,57,64,83,97], obstetric examinations [97], and labor procedures [97]. These were obtained before pregnancy [36,47,57,64,83,97], during pregnancy [97], and at labor [97]. The models predicted vaginal birth after a CS with the same definition as those of non-LR studies [36,47,57,64,83].

## Discussion

### Summary of Evidence

Of the 2093 records from 4 literature databases using 144 keywords, we found 142 eligible studies, among which 24 had a low ROB. These eligible studies developed prediction models for outcome categories of premature birth, *in vitro* fertilization, obstetric labor, pregnancy-induced hypertension, fetal distress, gestational diabetes, CS, fetal development, small-for-gestational-age infants, and others.

There were 4 models with non-LR algorithms from low ROB studies that had significantly higher differences in logit AUROCs than those with LR algorithms. The models used random forest algorithms to predict preterm delivery (2.51, 95% CI 1.49-3.53), gradient boosting algorithms to predict CS (2.26, 95% CI 1.39-3.13), random forest algorithms to predict pre-eclampsia (1.2, 95% CI 0.72-1.67), and gradient boosting algorithms to predict gestational diabetes (1.03, 95% CI 0.69-1.37). The first model that applied a random forest used only EHG records to predict preterm delivery. The second random forest model used only maternal demographics and medical histories but excluded obstetric ones for pre-eclampsia prediction. Meanwhile, the first model that applied a gradient boosting algorithm used only CTG records to predict CSs. The last model was developed by applying a gradient boosting algorithm for gestational diabetes. This model used maternal demographics, medical histories, obstetric histories, clinical or obstetric examinations, routine laboratory tests, and medications.

### Comparisons With Prior Work

We compared our systematic review and meta-analysis with prior works related to either machine learning algorithms or pregnancy outcomes similar to those in our study. A recent paper described applications of artificial intelligence in obstetrics and gynecology [176]. That paper was a narrative instead of a scoping or systematic review. Our systematic review and meta-analysis covered all pregnancy outcomes in obstetrics, as described in that paper. These were described as fetal heart monitoring and pregnancy surveillance, gestational diabetes mellitus, preterm labor, parturition, and *in vitro* fertilization.

Nevertheless, the predicted outcomes by non-LR models in our review were still insufficient. Diseases that cause maternal deaths should receive higher priority than those causing neonatal deaths. The risks were higher for pregnant women with antepartum hemorrhage (incidence rate ratio [IRR]=3.5, 95% CI 2.0-6.1) or hypertension (IRR=1.5, 95% CI 1.1-2.2) compared with those without these diseases [177]. Maternal sepsis was also associated with fetal or neonatal deaths (odds ratio [OR] 5.78, 95% CI 2.89-11.21) [178]. Accordingly, the impact of the prediction models may be insufficient to reduce both maternal and neonatal deaths.

LR was found in our study to be the most often used algorithm to develop a prediction model in pregnancy care, including predicted outcomes that caused the most maternal deaths, followed by artificial (shallow) neural networks, support vector machines, and deep neural networks. These corresponded to a systematic review and meta-analysis [13] that showed a similar majority of machine learning algorithms in medicine, except that the study reported classification and regression trees to be the second most often used algorithms (30/71, 42%). All models within eligible studies in that review were included instead of only choosing the best one within each study. Using the same summary measures as we did, the aforementioned review demonstrated that non-LR models from low ROB studies did not outperform LR models. A decision tree showed a difference of logit AUROCs of −0.34 (95% CI −0.65 to −0.04; *k*=16) compared with an LR. The review selected 125 eligible studies of 927 candidates from one database. Between-study heterogeneity was not described in that review.

Similar to a previous study [13], a systematic review and meta-analysis did not consider LR as a machine learning algorithm and only compared the predictive performances of non-LR algorithms [179]. This study compared machine learning models to predict any outcomes using routinely collected intensive care unit data. Most of the algorithms were artificial neural networks (72/169, 42.6%), support vector machines (40/169, 23.7%), and decision trees (35/169, 20.7%). However, since 2015, most of the algorithms were support vector machines (37/125, 29.6%) and random forests (72/169, 42.6%). These corresponded to the majority of machine learning algorithms for pregnancy care in our systematic review.

We hold a particular assumption to determine whether interaction of predictors and outcome may be best predicted by a prediction algorithm. If the same predictors and outcomes were used by the best prediction algorithm applied in either non-LR or LR models but not used by the other outcomes in this meta-analysis, then the prediction algorithm may be the best for the pregnancy outcome using those predictors. To predict preterm delivery with predictors that included EHG in either non-LR or LR models [115,169], the random forest outperformed the LR algorithm. Similar to this model in terms of using biomedical signals, gradient boosting also outperformed LR using CTG [142], but none of the LR counterparts used the same predictor. Other predictors were used across outcomes and algorithms (LR or non-LR). These included maternal demographics, lifestyle, medical or obstetric histories, clinical examinations, ultrasound measures, routine laboratory tests, biomarkers, and medication or procedures. Family histories were used in the LR models to predict gestational diabetes in this meta-analysis but were not used by the gradient boosting model (the non-LR counterpart). Therefore, we could not find a convincing pattern of predictors with respect to the best algorithms for each of the other pregnancy outcomes beyond preterm delivery.

Interestingly, the random forest significantly outperformed the LR for almost all of the pregnancy outcomes included in the meta-analysis. Although the gradient boosting algorithm significantly outperformed the LR for CS and gestational diabetes instead of the random forest, gradient boosting also uses multiple decision trees as in the random forest. For ongoing pregnancy predictions in *in vitro* fertilization, a random forest model from low ROB studies also showed the largest difference in logit AUROCs outperforming LR (1.22, 95% CI −0.03 to 2.48) compared with other non-LR algorithms. For predicting vaginal delivery after a CS, a non-LR algorithm, particularly an artificial neural network in our meta-analysis, did not significantly outperform LR.

Comparing differences in AUROCs and focusing on multiple prediction algorithms, a study with individual participant data also compared LR and non-LR algorithms, particularly Poisson regression, random forest, gradient boosting, and an ensemble of a random forest with either LR or support vector machine [180]. Several models were developed to predict all-cause readmissions in patients with heart failure within 30 and 180 days. The random forest significantly outperformed the LR (0.601, 95% CI 0.594-0.607 vs 0.533, 95% CI 0.527-0.538) for 30-day readmissions. Similar to the random forest, the gradient boosting algorithm (0.613, 95% CI 0.607-0.618) also significantly outperformed the LR. The predictors consisted of medical histories and routine laboratory tests.

Massive evaluation of 179 algorithms from 17 machine learning families was conducted using 121 data sets [181]. The best results were achieved using random forests. In our review, there were 13 studies in which the best models applied either a random forest [106,108,115,134,144,147,155,158] or gradient boosting [127,140,142,157,160]. Random forests used multiple subsets of all samples and predictors randomly with replacement to grow multiple parallel decision trees [182]. Although gradient boosting also uses multiple decision trees, the advantages of random forest over gradient boosting are robust to noise and overfitting [183]. Meanwhile, gradient boosting randomly uses multiple subsets of all samples without replacement to sequentially construct additive regression models [184]. The advantages of gradient boosting over random forests are state-of-the-art predictive performance on tabular data and the customizability of loss of function [181,185]. Hence, several gradient boosting algorithms were developed, and some studies in our review applied these algorithms. To predict gestational diabetes, Artzi et al [157] applied LightGBM, a scalable gradient boosting machine. This algorithm was optimized to speed up the training process by up to 20-fold with the same accuracy [186]. Another gradient boosting system (ie, XGBoost) [187] was applied in a study by Qiu et al [140] to predict live births after *in vitro* fertilization. This study was not included in our meta-analysis because there was an insufficient number of LR [61,69] and gradient boosting [140] algorithms for predicting live births.

Of the pregnancy outcomes predicted by non-LR algorithms in this review, most outcomes were *in vitro* fertilization, premature birth, and fetal distress, possibly because of several reasons. Using keywords of “machine learning IVF” in MEDLINE, we found a review paper from 2011 call for a need for artificial intelligence in *in vitro* fertilization [188]. Only one machine learning study for *in vitro* fertilization was found before that study [189]. All machine learning studies for *in vitro* fertilization were published after the review paper, and most studies were identified within 2093 records in our review [110,140,150,153,158,190-193]. As prediction for *in vitro* fertilization had already begun by 1989 [194], the machine learning prediction (non-LR) possibly arose because of the 2011 review. Meanwhile, for machine learning predictions of premature birth, fetal distress, and CS, many data sets (25/43, 58%) were secondary instead of primarily collected data. The secondary data sets consisted of predictors and outcomes of EHG and preterm delivery [174] (7/25, 28%), CTG, and acidotic blood pH of the umbilical artery [175] (4/25, 16%), CTG and CS [175] (2/25, 8%), CTG and acidotic blood pH of the umbilical artery [195] (3/25, 12%), EHG and preterm delivery [196] (2/25, 8%), and others (7/25, 28%). This implied that shared data sets drive more machine learning predictions compared with self-collected data sets. This indicates that the increase in publicly available data has driven progress in machine learning applications in health care [197].

For non-LR algorithms, the lack of shared data sets may have been the reason for few prediction studies for maternal outcomes compared with those for neonatal outcomes in this systematic review. Meanwhile, pregnancy-induced hypertension was found in pregnant women of newborns who were born prematurely [198]. Prematurity was also associated with maternal sepsis (OR 2.81, 95% CI 1.99-3.96), including antenatal cases [178]. Therefore, more shared data sets for maternal outcomes are needed. Future studies using machine learning algorithms should develop more prediction models for maternal outcomes in pregnancy care.

In addition, sample sizes of data sets for model development may contribute to bias in predictive performance. For example, in our meta-analysis, prediction models of ongoing pregnancy in *in vitro* fertilization had point estimates of AUROCs ranging from 0.575 to 0.982. These were developed using a support vector machine [110], artificial neural networks [132,136,170], random forests [134,158], deep neural networks [148,153], naïve Bayes algorithms [126,135,150], and LRs [73,78,99,158,170]. Compared with a recent systematic review focusing on prediction for *in vitro* fertilization [143,194], the range of AUROCs was wider than that of the previous review. The AUROCs ranged from 0.59 to 0.775 without non-LR machine learning predictions. A previous review also reported that the sample sizes ranged from 110 to 288,161 instances, whereas our review found that studies that applied non-LR algorithms alone or combined with LR had sample sizes ranging from only 46 [158] to 8836 [148] instances. Meanwhile, non-LR machine learning algorithms require larger sample sizes relative to the number of candidate predictors [199].

A meta-analysis of multivariable LR was also previously conducted for premature birth from 4 studies [200]. In a previous systematic review, the 2 highest AUROCs were 0.67 (95% CI 0.62-0.72; low ROB) and 0.64 (95% CI 0.60-0.68; high ROB). Non-LR models of premature birth in our systematic review showed AUROCs of 0.75 (95% CI 0.67-0.82) [121] and 0.911 (95% CI 0.862-0.96) [130], but these models were developed from high ROB studies. The other models only reported point estimates of the AUROC, which were a minimum of 0.6 by a decision tree [156] and a maximum of 0.991 by a support vector machine [143].

Minimizing the bias of model performance is the first thing to consider when developing a clinical prediction model. Several concerns need to be addressed when developing prognostic machine learning predictions of pregnancy care. In our review, most studies had problems of insufficient EPV (either LR and non-LR studies), single imputation (mostly LR studies), and no assessment of calibration (mostly non-LR studies). This may expose the studies to high ROBs [21]. The overestimation of the predictive performance is larger, with fewer participants with events relative to the number of predictor candidates, as described in the PROBAST guidelines. Most ROBs in our review were contributed by the domain of analysis, and answers to which the EPV signaling question mostly led studies to high ROB assessment results. Insufficient EPV mean that the study developed a model using a data set with a sample size that was less than the minimum requirement for events relative to the number of predictors. LR only requires 20 EPV, whereas non-LR algorithms require 50 to 200 EPV. Meanwhile, single imputation means that missing values are imputed by any random value, mean, median, mode, or one-time regression. Multiple imputations are more recommended than single imputations, in which the preferred method is multiple equations by chained equations. For the assessment of calibration, a study should show the incidence of events (true probability) for each subset of samples that belongs to the same range of predicted probability by the model. We recommend these based on PROBAST guidelines and other guidelines for machine learning prognostic predictions in pregnancy care [15,21].

### Strengths and Limitations

Our systematic review and meta-analysis will allow investigators or clinicians in pregnancy care to consider whether trying multiple machine learning models provides benefit to their studies. If more prediction models are needed for the outcomes with more specific problems or subpopulations, then predictive modeling may consider comparisons of LR and non-LR algorithms for specific outcomes that were compared in our meta-analysis. We also reported heterogeneity measures to interpret the predictive performances of algorithms across studies.

However, the diverse populations and hyperparameters caused substantial heterogeneity of predictive performance in our meta-analysis. Future meta-analyses will be needed if more machine learning models are developed for the same outcome using the same algorithm. However, we tried to minimize the heterogeneity by excluding several studies to ensure more homogenous outcome definitions and normally distributed AUROCs. We also applied random effects modeling as recommended [172].

### Conclusions

Prediction models using non-LR machine learning algorithms significantly outperformed those using LR for several pregnancy outcomes. These non-LR algorithms were random forests for predicting preterm delivery and pre-eclampsia and gradient boosting for predicting CS and gestational diabetes. In our review, studies that developed models using these algorithms had low ROBs. For predicting ongoing pregnancy in *in vitro* fertilization, non-LR algorithms did not significantly outperform LR. Prediction models using non-LR algorithms for vaginal birth after a CS significantly underperformed LR, but the study with the non-LR algorithm had a high ROB.

On the basis of our meta-analysis, we recommend comparing multiple machine learning models, which include both LR and non-LR algorithms, to develop a prediction model. In our systematic review, we also found that many studies had high ROBs in the domain of analysis. In this domain, many studies lacked EPV to develop a prediction model. Hence, we also recommend the future development of a prediction model to pursue standard EPV and other standards based on guidelines to minimize ROBs.

## Appendix

Multimedia Appendix 1 Details on forest plots, search filter, eligibility criteria, study selection, list of reviewed studies, risk of bias assessment, signaling questions and the answers, predictive performance and sample size, R code for meta-analysis, and records of studies.

## Abbreviations

AUROC: area under the receiver operating characteristic curve
CHARMS: checklist for critical appraisal and data extraction for systematic reviews of prediction modeling studies
CS: cesarean section
CTG: cardiotocogram
EHG: electrohysterogram
EPV: events per variable
IRR: incidence rate ratio
LR: logistic regression
MeSH: Medical Subject Heading
MLP-BIOM: guidelines for developing and reporting machine learning predictive models in biomedical research
OR: odds ratio
PI: prediction interval
PICOTS: population, index, comparator, outcomes, timing, and setting
PRISMA: Preferred Reporting Items for Systematic Reviews and Meta-Analyses
PROBAST: prediction model risk of bias assessment tool
ROB: risk of bias
ROC: receiver operating characteristic
TRIPOD: transparent reporting of a multivariable prediction model for individual prognosis or diagnosis

## References

[1] Domínguez-Almendros S, Benítez-Parejo N, Gonzalez-Ramirez A (2011). Logistic regression models. Allergol Immunopathol (Madr).

[2] Deo RC (2015). Machine learning in medicine. Circulation.

[3] Higgins JP (2002). Nonlinear systems in medicine. Yale J Biol Med.

[4] (2015). The Millennium Development Goals Report. United Nations.

[5] Say L, Chou D, Gemmill A, Tunçalp O, Moller A, Daniels J, Gülmezoglu AM, Temmerman M, Alkema L (2014). Global causes of maternal death: a WHO systematic analysis. Lancet Glob Health.

[6] Lehtonen L, Gimeno A, Parra-Llorca A, Vento M (2017). Early neonatal death: a challenge worldwide. Semin Fetal Neonatal Med.

[7] Burlinson CE, Sirounis D, Walley KR, Chau A (2018). Sepsis in pregnancy and the puerperium. Int J Obstet Anesth.

[8] Edwards HM (2018). Aetiology and treatment of severe postpartum haemorrhage. Dan Med J.

[9] Nair TM (2018). Statistical and artificial neural network-based analysis to understand complexity and heterogeneity in preeclampsia. Comput Biol Chem.

[10] Romero R, Dey SK, Fisher SJ (2014). Preterm labor: one syndrome, many causes. Science.

[11] Nindrea RD, Aryandono T, Lazuardi L, Dwiprahasto I (2018). Diagnostic accuracy of different machine learning algorithms for breast cancer risk calculation: a meta-analysis. Asian Pac J Cancer Prev.

[12] Lee Y, Ragguett R, Mansur RB, Boutilier JJ, Rosenblat JD, Trevizol A, Brietzke E, Lin K, Pan Z, Subramaniapillai M, Chan TC, Fus D, Park C, Musial N, Zuckerman H, Chen VC, Ho R, Rong C, McIntyre RS (2018). Applications of machine learning algorithms to predict therapeutic outcomes in depression: a meta-analysis and systematic review. J Affect Disord.

[13] Christodoulou E, Ma J, Collins GS, Steyerberg EW, Verbakel JY, Van Calster B (2019). A systematic review shows no performance benefit of machine learning over logistic regression for clinical prediction models. J Clin Epidemiol.

[14] Gregório Tiago, Pipa S, Cavaleiro P, Atanásio G, Albuquerque I, Chaves PC, Azevedo L (2018). Prognostic models for intracerebral hemorrhage: systematic review and meta-analysis. BMC Med Res Methodol.

[15] Luo W, Phung D, Tran T, Gupta S, Rana S, Karmakar C, Shilton A, Yearwood J, Dimitrova N, Ho TB, Venkatesh S, Berk M (2016). Guidelines for developing and reporting machine learning predictive models in biomedical research: a multidisciplinary view. J Med Internet Res.

[16] Nguyen AV, Blears EE, Ross E, Lall RR, Ortega-Barnett J (2018). Machine learning applications for the differentiation of primary central nervous system lymphoma from glioblastoma on imaging: a systematic review and meta-analysis. Neurosurg Focus.

[17] Wolff RF, Moons KG, Riley RD, Whiting PF, Westwood M, Collins GS, Reitsma JB, Kleijnen J, Mallett S, PROBAST Group† (2019). PROBAST: a tool to assess the risk of bias and applicability of prediction model studies. Ann Intern Med.

[18] Moher D, Liberati A, Tetzlaff J, Altman DG, PRISMA Group (2009). Preferred reporting items for systematic reviews and meta-analyses: the PRISMA statement. PLoS Med.

[19] Moons KG, de Groot JA, Bouwmeester W, Vergouwe Y, Mallett S, Altman DG, Reitsma JB, Collins GS (2014). Critical appraisal and data extraction for systematic reviews of prediction modelling studies: the CHARMS checklist. PLoS Med.

[20] Collins GS, Reitsma JB, Altman DG, Moons KG (2015). Transparent Reporting of a multivariable prediction model for individual prognosis or diagnosis (TRIPOD): the TRIPOD statement. Ann Intern Med.

[21] Moons KG, Wolff RF, Riley RD, Whiting PF, Westwood M, Collins GS, Reitsma JB, Kleijnen J, Mallett S (2019). PROBAST: a tool to assess risk of bias and applicability of prediction model studies: explanation and elaboration. Ann Intern Med.

[22] Mitchell TM (1997). Machine Learning.

[23] James SB, Bardenet R, Bengio Y, Balázs K (2011). Algorithms for Hyper-Parameter Optimization. Proceedings of the 24th International Conference on Neural Information Processing Systems.

[24] Snell KI, Hua H, Debray TP, Ensor J, Look MP, Moons KG, Riley RD (2016). Multivariate meta-analysis of individual participant data helped externally validate the performance and implementation of a prediction model. J Clin Epidemiol.

[25] Cheung M, Ho R, Lim Y, Mak A (2012). Conducting a meta-analysis: basics and good practices. Int J Rheum Dis.

[26] Riley RD, Higgins JP, Deeks JJ (2011). Interpretation of random effects meta-analyses. Br Med J.

[27] Viechtbauer W (2010). Conducting meta-analyses in with the package. J Stat Soft.

[28] Veroniki AA, Jackson D, Viechtbauer W, Bender R, Bowden J, Knapp G, Kuss O, Higgins JPT, Langan D, Salanti G (2016). Methods to estimate the between-study variance and its uncertainty in meta-analysis. Res Synth Methods.

[29] Allouche M, Huissoud C, Guyard-Boileau B, Rouzier R, Parant O (2011). Development and validation of nomograms for predicting preterm delivery. Am J Obstet Gynecol.

[30] Almeida S, Katz L, Coutinho I, Amorim M (2017). Validation of fullPIERS model for prediction of adverse outcomes among women with severe pre-eclampsia. Int J Gynaecol Obstet.

[31] Al-Rubaie ZT, Hudson HM, Jenkins G, Mahmoud I, Ray JG, Askie LM, Lord SJ (2020). Prediction of pre-eclampsia in nulliparous women using routinely collected maternal characteristics: a model development and validation study. BMC Pregnancy Childbirth.

[32] Bastek JA, Sammel MD, Srinivas SK, McShea MA, Foreman MN, Elovitz MA, Metlay JP (2012). Clinical prediction rules for preterm birth in patients presenting with preterm labor. Obstet Gynecol.

[33] Benhalima K, van Crombrugge P, Moyson C, Verhaeghe J, Vandeginste S, Verlaenen H, Vercammen C, Maes T, Dufraimont E, de Block C, Jacquemyn Y, Mekahli F, de Clippel K, Van Den Bruel A, Loccufier A, Laenen A, Minschart C, Devlieger R, Mathieu C (2020). Estimating the risk of gestational diabetes mellitus based on the 2013 WHO criteria: a prediction model based on clinical and biochemical variables in early pregnancy. Acta Diabetol.

[34] Berntorp K, Anderberg E, Claesson R, Ignell C, Källén K (2015). The relative importance of maternal body mass index and glucose levels for prediction of large-for-gestational-age births. BMC Pregnancy Childbirth.

[35] Broekmans FJ, Verweij PJ, Eijkemans MJ, Mannaerts BM, Witjes H (2014). Prognostic models for high and low ovarian responses in controlled ovarian stimulation using a GnRH antagonist protocol. Hum Reprod.

[36] Fagerberg MC, Källén K (2020). Third-trimester prediction of successful vaginal birth after one cesarean delivery-a Swedish model. Acta Obstet Gynecol Scand.

[37] Casikar I, Lu C, Reid S, Condous G (2013). Prediction of successful expectant management of first trimester miscarriage: development and validation of a new mathematical model. Aust N Z J Obstet Gynaecol.

[38] Cerqueira FR, Ferreira TG, de Paiva Oliveira A, Augusto DA, Krempser E, Corrêa Barbosa HJ, do Carmo Castro Franceschini S, de Freitas BA, Gomes AP, Siqueira-Batista R (2014). NICeSim: an open-source simulator based on machine learning techniques to support medical research on prenatal and perinatal care decision making. Artif Intell Med.

[39] Chandrasekaran S, Bastek JA, Turitz AL, Durnwald CP (2016). A prediction score to assess the risk of delivering a large for gestational age infant among obese women. J Matern Fetal Neonatal Med.

[40] Chen L, Luo D, Yu X, Jin M, Cai W (2018). Predicting stress urinary incontinence during pregnancy: combination of pelvic floor ultrasound parameters and clinical factors. Acta Obstet Gynecol Scand.

[41] Ciobanu A, Rouvali A, Syngelaki A, Akolekar R, Nicolaides KH (2019). Prediction of small for gestational age neonates: screening by maternal factors, fetal biometry, and biomarkers at 35-37 weeks' gestation. Am J Obstet Gynecol.

[42] Cortet M, Maucort-Boulch D, Deneux-Tharaux C, Dupont C, Rudigoz R, Roy P, Huissoud C (2015). Severity of post-partum hemorrhage after vaginal delivery is not predictable from clinical variables available at the time post-partum hemorrhage is diagnosed. J Obstet Gynaecol Res.

[43] Crovetto F, Figueras F, Triunfo S, Crispi F, Rodriguez-Sureda V, Dominguez C, Llurba E, Gratacós E (2015). First trimester screening for early and late preeclampsia based on maternal characteristics, biophysical parameters, and angiogenic factors. Prenat Diagn.

[44] de Oliveira RV, Martins MD, Rios LT, Araujo Júnior E, Simões VM, Nardozza LM, Moron AF (2012). Predictive model for spontaneous preterm labor among pregnant women with contractions and intact amniotic membranes. Arch Gynecol Obstet.

[45] de Wilde MA, Veltman-Verhulst SM, Goverde AJ, Lambalk CB, Laven JS, Franx A, Koster MP, Eijkemans MJ, Fauser BC (2014). Preconception predictors of gestational diabetes: a multicentre prospective cohort study on the predominant complication of pregnancy in polycystic ovary syndrome. Hum Reprod.

[46] Eggebø TM, Wilhelm-Benartzi C, Hassan WA, Usman S, Salvesen KA, Lees CC (2015). A model to predict vaginal delivery in nulliparous women based on maternal characteristics and intrapartum ultrasound. Am J Obstet Gynecol.

[47] Fagerberg MC, Maršál K, Källén K (2015). Predicting the chance of vaginal delivery after one cesarean section: validation and elaboration of a published prediction model. Eur J Obstet Gynecol Reprod Biol.

[48] Guo Z, Yang F, Zhang J, Zhang Z, Li K, Tian Q, Hou H, Xu C, Lu Q, Ren Z, Yang X, Lv Z, Wang K, Yang X, Wu Y, Yang X (2020). Whole-genome promoter profiling of plasma DNA exhibits diagnostic value for placenta-origin pregnancy complications. Adv Sci (Weinh).

[49] Harper LM, Glover AV, Biggio JR, Tita A (2016). Predicting failure of glyburide therapy in gestational diabetes. J Perinatol.

[50] Isono W, Nagamatsu T, Uemura Y, Fujii T, Hyodo H, Yamashita T, Kamei Y, Kozuma S, Taketani Y (2011). Prediction model for the incidence of emergent cesarean section during induction of labor specialized in nulliparous low-risk women. J Obstet Gynaecol Res.

[51] Kang J, Kim HS, Lee EB, Uh Y, Han K, Park EY, Lee HA, Kang DR, Chung I, Choi SJ (2020). Prediction model for massive transfusion in placenta previa during cesarean section. Yonsei Med J.

[52] Kawakita T, Mokhtari N, Huang JC, Landy HJ (2019). Evaluation of risk-assessment tools for severe postpartum hemorrhage in women undergoing cesarean delivery. Obstet Gynecol.

[53] Khan N, Ciobanu A, Karampitsakos T, Akolekar R, Nicolaides KH (2019). Prediction of large-for-gestational-age neonate by routine third-trimester ultrasound. Ultrasound Obstet Gynecol.

[54] Kok M, van der Steeg J, van der Post J, Mol B (2011). Prediction of success of external cephalic version after 36 weeks. Am J Perinatol.

[55] Lafalla O, Esteban LM, Lou AC, Cornudella R, Domínguez M, Sanz G, Borque-Fernando A (2019). Clinical utility of thrombophilia, anticoagulant treatment, and maternal variables as predictors of placenta-mediated pregnancy complications: an extensive analysis. J Matern Fetal Neonatal Med.

[56] Lee JS, Sultana R, Han NL, Sia AT, Sng BL (2018). Development and validation of a predictive risk factor model for epidural re-siting in women undergoing labour epidural analgesia: a retrospective cohort study. BMC Anesthesiol.

[57] Mardy AH, Ananth CV, Grobman WA, Gyamfi-Bannerman C (2016). A prediction model of vaginal birth after cesarean in the preterm period. Am J Obstet Gynecol.

[58] McCowan LM, Thompson JM, Taylor RS, Baker PN, North RA, Poston L, Roberts CT, Simpson NA, Walker JJ, Myers J, Kenny LC, SCOPE consortium (2017). Prediction of small for gestational age infants in healthy nulliparous women using clinical and ultrasound risk factors combined with early pregnancy biomarkers. PLoS One.

[59] McCowan LM, Thompson JM, Taylor RS, North RA, Poston L, Baker PN, Myers J, Roberts CT, Dekker GA, Simpson NA, Walker JJ, Kenny LC, SCOPE Consortium (2013). Clinical prediction in early pregnancy of infants small for gestational age by customised birthweight centiles: findings from a healthy nulliparous cohort. PLoS One.

[60] Mehta-Lee SS, Palma A, Bernstein PS, Lounsbury D, Schlecht NF (2017). A preconception nomogram to predict preterm delivery. Matern Child Health J.

[61] Meijerink A, Cissen M, Mochtar M, Fleischer K, Thoonen I, de Melker A, Meissner A, Repping S, Braat D, van Wely M, Ramos L (2016). Prediction model for live birth in ICSI using testicular extracted sperm. Hum Reprod.

[62] Meister M, Cahill A, Conner S, Woolfolk C, Lowder J (2016). Predicting obstetric anal sphincter injuries in a modern obstetric population. Am J Obstet Gynecol.

[63] Menon R, Bhat G, Saade GR, Spratt H (2014). Multivariate adaptive regression splines analysis to predict biomarkers of spontaneous preterm birth. Acta Obstet Gynecol Scand.

[64] Metz TD, Stoddard GJ, Henry E, Jackson M, Holmgren C, Esplin S (2013). Simple, validated vaginal birth after cesarean delivery prediction model for use at the time of admission. Obstet Gynecol.

[65] Murtoniemi K, Villa PM, Matomäki J, Keikkala E, Vuorela P, Hämäläinen E, Kajantie E, Pesonen A, Räikkönen K, Taipale P, Stenman U, Laivuori H (2018). Prediction of pre-eclampsia and its subtypes in high-risk cohort: hyperglycosylated human chorionic gonadotropin in multivariate models. BMC Pregnancy Childbirth.

[66] Myers J, Kenny L, McCowan L, Chan E, Dekker G, Poston L, Simpson N, North R, SCOPE consortium (2013). Angiogenic factors combined with clinical risk factors to predict preterm pre-eclampsia in nulliparous women: a predictive test accuracy study. BJOG.

[67] Oates J, Casikar I, Campain A, Müller S, Yang J, Reid S, Condous G (2013). A prediction model for viability at the end of the first trimester after a single early pregnancy evaluation. Aust N Z J Obstet Gynaecol.

[68] Payne BA, Groen H, Ukah UV, Ansermino JM, Bhutta Z, Grobman W, Hall DR, Hutcheon JA, Magee LA, von Dadelszen P, miniPIERS working group (2015). Development and internal validation of a multivariable model to predict perinatal death in pregnancy hypertension. Pregnancy Hypertens.

[69] Pettersson G, Andersen AN, Broberg P, Arce J (2010). Pre-stimulation parameters predicting live birth after IVF in the long GnRH agonist protocol. Reprod Biomed Online.

[70] Pettersson K, Yousaf K, Ranstam J, Westgren M, Ajne G (2017). Predictive value of traction force measurement in vacuum extraction: development of a multivariate prognostic model. PLoS One.

[71] Ramanah R, Omar S, Guillien A, Pugin A, Martin A, Riethmuller D, Mottet N (2018). Predicting umbilical artery pH during labour: development and validation of a nomogram using fetal heart rate patterns. Eur J Obstet Gynecol Reprod Biol.

[72] Reid S, Lu C, Condous G (2015). Can we improve the prediction of pouch of Douglas obliteration in women with suspected endometriosis using ultrasound-based models? A multicenter prospective observational study. Acta Obstet Gynecol Scand.

[73] Rinaudo P, Shen S, Hua J, Qian S, Prabhu U, Garcia E, Cedars M, Sukumaran D, Szyperski T, Andrews C (2012). (1)H NMR based profiling of spent culture media cannot predict success of implantation for day 3 human embryos. J Assist Reprod Genet.

[74] Ryu A, Cho NJ, Kim YS, Lee EY (2019). Predictive value of serum uric acid levels for adverse perinatal outcomes in preeclampsia. Medicine (Baltimore).

[75] Sananes N, Meyer N, Gaudineau A, Aissi G, Boudier E, Fritz G, Viville B, Nisand I, Langer B, Favre R (2013). Prediction of spontaneous preterm delivery in the first trimester of pregnancy. Eur J Obstet Gynecol Reprod Biol.

[76] Sandström A, Snowden JM, Höijer J, Bottai M, Wikström AK (2019). Clinical risk assessment in early pregnancy for preeclampsia in nulliparous women: a population based cohort study. PLoS One.

[77] Scheinhardt M, Lerman T, König IR, Griesinger G (2018). Performance of prognostic modelling of high and low ovarian response to ovarian stimulation for IVF. Hum Reprod.

[78] Shi W, Zhang S, Zhao W, Xia X, Wang M, Wang H, Bai H, Shi J (2013). Factors related to clinical pregnancy after vitrified-warmed embryo transfer: a retrospective and multivariate logistic regression analysis of 2313 transfer cycles. Hum Reprod.

[79] Sovio U, Smith GC (2018). Blinded ultrasound fetal biometry at 36 weeks and risk of emergency Cesarean delivery in a prospective cohort study of low-risk nulliparous women. Ultrasound Obstet Gynecol.

[80] Stamatopoulos N, Lu C, Casikar I, Reid S, Mongelli M, Hardy N, Condous G (2015). Prediction of subsequent miscarriage risk in women who present with a viable pregnancy at the first early pregnancy scan. Aust N Z J Obstet Gynaecol.

[81] Stott D, Bolten M, Salman M, Paraschiv D, Douiri A, Kametas NA (2017). A prediction model for the response to oral labetalol for the treatment of antenatal hypertension. J Hum Hypertens.

[82] Stroux L, Redman CW, Georgieva A, Payne SJ, Clifford GD (2017). Doppler-based fetal heart rate analysis markers for the detection of early intrauterine growth restriction. Acta Obstet Gynecol Scand.

[83] Tessmer-Tuck JA, El-Nashar SA, Racek AR, Lohse CM, Famuyide AO, Wick MJ (2014). Predicting vaginal birth after cesarean section: a cohort study. Gynecol Obstet Invest.

[84] Thériault S, Giguère Y, Massé J, Girouard J, Forest J (2016). Early prediction of gestational diabetes: a practical model combining clinical and biochemical markers. Clin Chem Lab Med.

[85] Timmerman E, Oude Rengerink K, Pajkrt E, Opmeer BC, van der Post JA, Bilardo CM (2010). Ductus venosus pulsatility index measurement reduces the false-positive rate in first-trimester screening. Ultrasound Obstet Gynecol.

[86] Tsur A, Batsry L, Toussia-Cohen S, Rosenstein MG, Barak O, Brezinov Y, Yoeli-Ullman R, Sivan E, Sirota M, Druzin ML, Stevenson DK, Blumenfeld YJ, Aran D (2020). Development and validation of a machine-learning model for prediction of shoulder dystocia. Ultrasound Obstet Gynecol.

[87] van Baaren GJ, Bruijn MM, Vis JY, Wilms FF, Oudijk MA, Kwee A, Porath MM, Oei G, Scheepers HC, Spaanderman ME, Bloemenkamp KW, Haak MC, Bolte AC, Bax CJ, Cornette JM, Duvekot JJ, Nij Bijvanck BW, van Eijck J, Franssen MT, Sollie KM, Vandenbussche FP, Woiski M, Bossuyt P, Opmeer B, Mol BW (2015). Risk factors for preterm delivery: do they add to fetal fibronectin testing and cervical length measurement in the prediction of preterm delivery in symptomatic women?. Eur J Obstet Gynecol Reprod Biol.

[88] Van Calster B, Condous G, Kirk E, Bourne T, Timmerman D, Van Huffel S (2009). An application of methods for the probabilistic three-class classification of pregnancies of unknown location. Artif Intell Med.

[89] van der Ham DP, van Kuijk S, Opmeer BC, Willekes C, van Beek JJ, Mulder AL, van Loon AJ, Groenewout M, Mantel GD, Bloemenkamp KW, Porath M, Kwee A, Akerboom BM, Papatsonis DN, Metz GC, Nijhuis JG, Mol BW, PPROMEXIL trial group (2014). Can neonatal sepsis be predicted in late preterm premature rupture of membranes? Development of a prediction model. Eur J Obstet Gynecol Reprod Biol.

[90] van der Tuuk K, van Pampus MG, Koopmans C, Aarnoudse J, van den Berg PP, van Beek JJ, Copraij F, Kleiverda G, Porath M, Rijnders R, van der Salm PC, Morssink LP, Stigter R, Mol B, Groen H, HYPITAT study group (2015). Prediction of cesarean section risk in women with gestational hypertension or mild preeclampsia at term. Eur J Obstet Gynecol Reprod Biol.

[91] Verhoeven CJ, Nuij C, Janssen-Rolf CR, Schuit E, Bais JM, Oei SG, Mol BW (2016). Predictors for failure of vacuum-assisted vaginal delivery: a case-control study. Eur J Obstet Gynecol Reprod Biol.

[92] Vieira MC, White SL, Patel N, Seed PT, Briley AL, Sandall J, Welsh P, Sattar N, Nelson SM, Lawlor DA, Poston L, Pasupathy D, UPBEAT Consortium (2017). Prediction of uncomplicated pregnancies in obese women: a prospective multicentre study. BMC Med.

[93] Visentin S, Londero AP, Camerin M, Grisan E, Cosmi E (2017). A possible new approach in the prediction of late gestational hypertension: the role of the fetal aortic intima-media thickness. Medicine (Baltimore).

[94] Wang C, Zhu W, Wei Y, Su R, Feng H, Lin L, Yang H (2016). The predictive effects of early pregnancy lipid profiles and fasting glucose on the risk of gestational diabetes mellitus stratified by body mass index. J Diabetes Res.

[95] Wang L, Matsunaga S, Mikami Y, Takai Y, Terui K, Seki H (2016). Pre-delivery fibrinogen predicts adverse maternal or neonatal outcomes in patients with placental abruption. J Obstet Gynaecol Res.

[96] Weber A, Darmstadt GL, Gruber S, Foeller ME, Carmichael SL, Stevenson DK, Shaw GM (2018). Application of machine-learning to predict early spontaneous preterm birth among nulliparous non-Hispanic black and white women. Ann Epidemiol.

[97] Xing Y, Qi X, Wang X, Yang F (2019). Development of a modified score system as prediction model for successful vaginal birth after cesarean delivery. Clin Transl Sci.

[98] Xu H, Feng G, Wei Y, Feng Y, Yang R, Wang L, Zhang H, Li R, Qiao J (2020). Predicting ectopic pregnancy using human chorionic gonadotropin (HCG) levels and main cause of infertility in women undergoing assisted reproductive treatment: retrospective observational cohort study. JMIR Med Inform.

[99] Xu H, Wei Y, Yang R, Feng G, Tang W, Zhang H, He Y, Feng Y, Li R, Qiao J (2019). Prospective observational cohort study: computational models for early prediction of ongoing pregnancy in fresh IVF/ICSI-ET protocols. Life Sci.

[100] Yang H, Zhu C, Ma Q, Long Y, Cheng Z (2015). Variations of blood cells in prediction of gestational diabetes mellitus. J Perinat Med.

[101] Yang T, Li N, Qiao C, Liu C (2019). Development of a novel nomogram for predicting placenta accreta in patients with scarred uterus: a retrospective cohort study. Front Med (Lausanne).

[102] Yu C, Zhang R, Li J (2018). A predictive model for high-quality blastocyst based on blastomere number, fragmentation, and symmetry. J Assist Reprod Genet.

[103] Zhao R, Zhang W, Zhou L, Chen Y (2019). Building a predictive model for successful vaginal delivery in nulliparas with term cephalic singleton pregnancies using decision tree analysis. J Obstet Gynaecol Res.

[104] Zheng T, Ye W, Wang X, Li X, Zhang J, Little J, Zhou L, Zhang L (2019). A simple model to predict risk of gestational diabetes mellitus from 8 to 20 weeks of gestation in Chinese women. BMC Pregnancy Childbirth.

[105] Zwertbroek E, Broekhuijsen K, Langenveld J, van Baaren G, van den Berg P, Bremer H, Ganzevoort W, van Loon A, Mol B, van Pampus M, Perquin D, Rijnders R, Scheepers H, Sikkema M, Woiski M, Groen H, Franssen M, HYPITAT-II Study Group (2017). Prediction of progression to severe disease in women with late preterm hypertensive disorders of pregnancy. Acta Obstet Gynecol Scand.

[106] Abbas SA, Riaz R, Kazmi SZ, Rizvi SS, Kwon SJ (2018). Cause analysis of caesarian sections and application of machine learning methods for classification of birth data. IEEE Access.

[107] Alberola-Rubio J, Garcia-Casado J, Prats-Boluda G, Ye-Lin Y, Desantes D, Valero J, Perales A (2017). Prediction of labor onset type: spontaneous vs induced; role of electrohysterography?. Comput Methods Programs Biomed.

[108] Balani J, Hyer S, Shehata H, Mohareb F (2018). Visceral fat mass as a novel risk factor for predicting gestational diabetes in obese pregnant women. Obstet Med.

[109] Benalcazar-Parra C, Ye-Lin Y, Garcia-Casado J, Monfort-Ortiz R, Alberola-Rubio J, Perales A, Prats-Boluda G (2019). Prediction of labor induction success from the uterine electrohysterogram. J Sensors.

[110] Borup R, Thuesen L, Andersen C, Nyboe-Andersen A, Ziebe S, Winther O, Grøndahl ML (2016). Competence classification of cumulus and granulosa cell transcriptome in embryos matched by morphology and female age. PLoS One.

[111] Chen L, Hao Y (2017). Feature extraction and classification of EHG between pregnancy and labour group using Hilbert-Huang transform and extreme learning machine. Comput Math Methods Med.

[112] Chen L, Hao Y, Hu X (2019). Detection of preterm birth in electrohysterogram signals based on wavelet transform and stacked sparse autoencoder. PLoS One.

[113] Cömert Z, Kocamaz AF, Subha V (2018). Prognostic model based on image-based time-frequency features and genetic algorithm for fetal hypoxia assessment. Comput Biol Med.

[114] Coppedè F, Grossi E, Migheli F, Migliore L (2010). Polymorphisms in folate-metabolizing genes, chromosome damage, and risk of Down syndrome in Italian women: identification of key factors using artificial neural networks. BMC Med Genomics.

[115] Despotovic D, Zec A, Mladenovic K, Radin N, Turukalo T (2018). A machine learning approach for an early prediction of preterm delivery. 16th International Symposium on Intelligent Systems and Informatics.

[116] Elaveyini U, Devi SP, Rao KS (2011). Neural networks prediction of preterm delivery with first trimester bleeding. Arch Gynecol Obstet.

[117] Fergus P, Hussain A, Al-Jumeily D, Huang D, Bouguila N (2017). Classification of caesarean section and normal vaginal deliveries using foetal heart rate signals and advanced machine learning algorithms. Biomed Eng Online.

[118] Fergus P, Idowu I, Hussain A, Dobbins C (2016). Advanced artificial neural network classification for detecting preterm births using EHG records. Neurocomputing.

[119] Fergus P, Montanez A, Abdulaimma B, Lisboa P, Chalmers C, Pineles B (2020). Utilizing deep learning and genome wide association studies for epistatic-driven preterm birth classification in African-American women. IEEE/ACM Trans Comput Biol Bioinform.

[120] Figueras F, Savchev S, Triunfo S, Crovetto F, Gratacos E (2015). An integrated model with classification criteria to predict small-for-gestational-age fetuses at risk of adverse perinatal outcome. Ultrasound Obstet Gynecol.

[121] Fiset S, Martel A, Glanc P, Barrett J, Melamed N (2019). Prediction of spontaneous preterm birth among twin gestations using machine learning and texture analysis of cervical ultrasound images. Univ Tor Med J.

[122] Gao C, Osmundson S, Velez Edwards DR, Jackson G, Malin B, Chen Y (2019). Deep learning predicts extreme preterm birth from electronic health records. J Biomed Inform.

[123] Garcés MF, Sanchez E, Cardona LF, Simanca EL, González I, Leal LG, Mora JA, Bedoya A, Alzate JP, Sánchez AY, Eslava-Schmalbach JH, Franco-Vega R, Parra MO, Ruíz-Parra AI, Diéguez C, Nogueiras R, Caminos JE (2015). Maternal serum meteorin levels and the risk of preeclampsia. PLoS One.

[124] Georgoulas G, Karvelis P, Spilka J, Chudáček V, Stylios CD, Lhotská L (2017). Investigating pH based evaluation of fetal heart rate (FHR) recordings. Health Technol (Berl).

[125] Hamdi M, Limem M, Maaref M (2019). Detection and classification of nonstationary signals: application to uterine EMG for prognostication of premature delivery. Neurophysiology.

[126] Hernández-González J, Inza I, Crisol-Ortíz L, Guembe M, Iñarra MJ, Lozano J (2018). Fitting the data from embryo implantation prediction: learning from label proportions. Stat Methods Med Res.

[127] Jhee JH, Lee S, Park Y, Lee SE, Kim YA, Kang S, Kwon J, Park JT (2019). Prediction model development of late-onset preeclampsia using machine learning-based methods. PLoS One.

[128] Leonarduzzi R, Spilka J, Frecon J, Wendt H, Pustelnik N, Jaffard S, Abry P, Doret M (2015). P-leader multifractal analysis and sparse SVM for intrapartum fetal acidosis detection. Annu Int Conf IEEE Eng Med Biol Soc.

[129] Li H, Luo M, Zheng J, Luo J, Zeng R, Feng N, Du Q, Fang J (2017). An artificial neural network prediction model of congenital heart disease based on risk factors: a hospital-based case-control study. Medicine (Baltimore).

[130] Mas-Cabo J, Prats-Boluda G, Garcia-Casado J, Alberola-Rubio J, Perales A, Ye-Lin Y (2019). Design and assessment of a robust and generalizable ANN-based classifier for the prediction of premature birth by means of multichannel electrohysterographic records. J Sensors.

[131] Mello G, Parretti E, Ognibene A, Mecacci F, Cioni R, Scarselli G, Messeri G (2001). Prediction of the development of pregnancy-induced hypertensive disorders in high-risk pregnant women by artificial neural networks. Clin Chem Lab Med.

[132] Milewski R, Kuczyńska A, Stankiewicz B, Kuczyński W (2017). How much information about embryo implantation potential is included in morphokinetic data? A prediction model based on artificial neural networks and principal component analysis. Adv Med Sci.

[133] Milewski R, Milewska AJ, Więsak T, Morgan A (2013). Comparison of artificial neural networks and logistic regression analysis in pregnancy prediction using the in vitro fertilization treatment. Stud Log Gramm Rhetor.

[134] Mirroshandel S, Ghasemian F, Monji-Azad S (2016). Applying data mining techniques for increasing implantation rate by selecting best sperms for intra-cytoplasmic sperm injection treatment. Comput Methods Programs Biomed.

[135] Morales DA, Bengoetxea E, Larrañaga P, García M, Franco Y, Fresnada M, Merino M (2008). Bayesian classification for the selection of in vitro human embryos using morphological and clinical data. Comput Methods Programs Biomed.

[136] Paydar K, Niakan Kalhori SR, Akbarian M, Sheikhtaheri A (2017). A clinical decision support system for prediction of pregnancy outcome in pregnant women with systemic lupus erythematosus. Int J Med Inform.

[137] Petrozziello A, Jordanov I, Aris Papageorghiou T, Christopher Redman WG, Georgieva A (2018). Deep learning for continuous electronic fetal monitoring in labor. Annu Int Conf IEEE Eng Med Biol Soc.

[138] Petrozziello A, Redman C, Papageorghiou A, Jordanov I, Georgieva A (2019). Multimodal convolutional neural networks to detect fetal compromise during labor and delivery. IEEE Access.

[139] Qiu H, Yu H, Wang L, Yao Q, Wu S, Yin C, Fu B, Zhu X, Zhang Y, Xing Y, Deng J, Yang H, Lei S (2017). Electronic health record driven prediction for gestational diabetes mellitus in early pregnancy. Sci Rep.

[140] Qiu J, Li P, Dong M, Xin X, Tan J (2019). Personalized prediction of live birth prior to the first in vitro fertilization treatment: a machine learning method. J Transl Med.

[141] Sadi-Ahmed N, Kacha B, Taleb H, Kedir-Talha M (2017). Relevant features selection for automatic prediction of preterm deliveries from pregnancy electrohysterograhic (EHG) records. J Med Syst.

[142] Saleem S, Naqvi S, Manzoor T, Saeed A, Ur Rehman N, Mirza J (2019). A strategy for classification of 'vaginal vs cesarean section' delivery: bivariate empirical mode decomposition of cardiotocographic recordings. Front Physiol.

[143] Shahbakhti M, Beiramvand M, Bavi M, Mohammadi Far S (2019). A new efficient algorithm for prediction of preterm labor. Annu Int Conf IEEE Eng Med Biol Soc.

[144] Signorini MG, Pini N, Malovini A, Bellazzi R, Magenes G (2020). Integrating machine learning techniques and physiology based heart rate features for antepartum fetal monitoring. Comput Methods Programs Biomed.

[145] Spilka J, Frecon J, Leonarduzzi R, Pustelnik N, Abry P, Doret M (2015). Intrapartum fetal heart rate classification from trajectory in Sparse SVM feature space. Annu Int Conf IEEE Eng Med Biol Soc.

[146] Spilka J, Frecon J, Leonarduzzi R, Pustelnik N, Abry P, Doret M (2017). Sparse support vector machine for intrapartum fetal heart rate classification. IEEE J Biomed Health Inform.

[147] Sufriyana H, Wu Y, Su EC (2020). Artificial intelligence-assisted prediction of preeclampsia: development and external validation of a nationwide health insurance dataset of the BPJS Kesehatan in Indonesia. EBioMedicine.

[148] Tran D, Cooke S, Illingworth P, Gardner D (2019). Deep learning as a predictive tool for fetal heart pregnancy following time-lapse incubation and blastocyst transfer. Hum Reprod.

[149] Troisi J, Landolfi A, Sarno L, Richards S, Symes S, Adair D, Ciccone C, Scala G, Martinelli P, Guida M (2018). A metabolomics-based approach for non-invasive screening of fetal central nervous system anomalies. Metabolomics.

[150] Uyar A, Bener A, Ciray HN (2015). Predictive modeling of implantation outcome in an in vitro fertilization setting: an application of machine learning methods. Med Decis Making.

[151] Uyar A, Bener A, Ciray H (2009). ROC Based Evaluation and Comparison of Classifiers for IVF Implantation Prediction. International Conference on Electronic Healthcare.

[152] Valensise H, Facchinetti F, Vasapollo B, Giannini F, Monte ID, Arduini D (2006). The computerized fetal heart rate analysis in post-term pregnancy identifies patients at risk for fetal distress in labour. Eur J Obstet Gynecol Reprod Biol.

[153] VerMilyea M, Hall J, Diakiw S, Johnston A, Nguyen T, Perugini D, Miller A, Picou A, Murphy A, Perugini M (2020). Development of an artificial intelligence-based assessment model for prediction of embryo viability using static images captured by optical light microscopy during IVF. Hum Reprod.

[154] Vogiatzi P, Pouliakis A, Siristatidis C (2019). An artificial neural network for the prediction of assisted reproduction outcome. J Assist Reprod Genet.

[155] Xu L, Georgieva A, Redman C, Payne S (2013). Feature selection for computerized fetal heart rate analysis using genetic algorithms. Annu Int Conf IEEE Eng Med Biol Soc.

[156] Amini P, Maroufizadeh S, Samani RO, Hamidi O, Sepidarkish M (2017). Prevalence and determinants of preterm birth in Tehran, Iran: a comparison between logistic regression and decision tree methods. Osong Public Health Res Perspect.

[157] Artzi NS, Shilo S, Hadar E, Rossman H, Barbash-Hazan S, Ben-Haroush A, Balicer RD, Feldman B, Wiznitzer A, Segal E (2020). Prediction of gestational diabetes based on nationwide electronic health records. Nat Med.

[158] Blank C, Wildeboer R, DeCroo I, Tilleman K, Weyers B, de Sutter P, Mischi M, Schoot B (2019). Prediction of implantation after blastocyst transfer in in vitro fertilization: a machine-learning perspective. Fertil Steril.

[159] Isakov O, Reicher L, Lavie A, Yogev Y, Maslovitz S (2019). Prediction of success in external cephalic version for breech presentation at term. Obstet Gynecol.

[160] Koivu A, Sairanen M (2020). Predicting risk of stillbirth and preterm pregnancies with machine learning. Health Inf Sci Syst.

[161] Kuhle S, Maguire B, Zhang H, Hamilton D, Allen AC, Joseph KS, Allen VM (2018). Comparison of logistic regression with machine learning methods for the prediction of fetal growth abnormalities: a retrospective cohort study. BMC Pregnancy Childbirth.

[162] Kumar SN, Saxena P, Patel R, Sharma A, Pradhan D, Singh H, Deval R, Bhardwaj SK, Borgohain D, Akhtar N, Raisuddin S, Jain AK (2020). Predicting risk of low birth weight offspring from maternal features and blood polycyclic aromatic hydrocarbon concentration. Reprod Toxicol.

[163] Lee K, Ahn KH (2019). Artificial neural network analysis of spontaneous preterm labor and birth and its major determinants. J Korean Med Sci.

[164] Liu B, Shi S, Wu Y, Thomas D, Symul L, Pierson E, Leskovec J (2019). Predicting pregnancy using large-scale data from a women's health tracking mobile application. Proc Int World Wide Web Conf.

[165] Macones GA, Hausman N, Edelstein R, Stamilio DM, Marder SJ (2001). Predicting outcomes of trials of labor in women attempting vaginal birth after cesarean delivery: a comparison of multivariate methods with neural networks. Am J Obstet Gynecol.

[166] Maroufizadeh S, Amini P, Hosseini M, Almasi-Hashiani A, Mohammadi M, Navid B, Omani-Samani R (2018). Determinants of cesarean section among primiparas: a comparison of classification methods. Iran J Public Health.

[167] Sims CJ, Meyn L, Caruana R, Rao R, Mitchell T, Krohn M (2000). Predicting cesarean delivery with decision tree models. Am J Obstet Gynecol.

[168] Agopian A, Lupo P, Tinker S, Canfield M, Mitchell L, National Birth Defects Prevention Study (2012). Working towards a risk prediction model for neural tube defects. Birth Defects Res A Clin Mol Teratol.

[169] Fergus P, Cheung P, Hussain A, Al-Jumeily D, Dobbins C, Iram S (2013). Prediction of preterm deliveries from EHG signals using machine learning. PLoS One.

[170] Wald M, Sparks A, Sandlow J, Van-Voorhis B, Syrop C, Niederberger C (2005). Computational models for prediction of IVF/ICSI outcomes with surgically retrieved spermatozoa. Reprod Biomed Online.

[171] Huedo-Medina TB, Sánchez-Meca J, Marín-Martínez F, Botella J (2006). Assessing heterogeneity in meta-analysis: Q statistic or I2 index?. Psychol Methods.

[172] Deeks J, Higgins J, Altman D, Higgins J, Green S (2008). Analysing data undertaking meta-analyses. Cochrane Handbook for Systematic Reviews of Interventions.

[173] Borenstein M, Higgins JP, Hedges LV, Rothstein HR (2017). Basics of meta-analysis: I^2^ is not an absolute measure of heterogeneity. Res Synth Methods.

[174] Fele-Zorz G, Kavsek G, Novak-Antolic Z, Jager F (2008). A comparison of various linear and non-linear signal processing techniques to separate uterine EMG records of term and pre-term delivery groups. Med Biol Eng Comput.

[175] Chudáček V, Spilka J, Burša M, Janků P, Hruban L, Huptych M, Lhotská L (2014). Open access intrapartum CTG database. BMC Pregnancy Childbirth.

[176] Iftikhar P, Kuijpers M, Khayyat A, Iftikhar A, DeGouvia de Sa M (2020). A comparison of various linear and non-linear signal processing techniques to separate uterine EMG records of term and pre-term delivery groups. Cureus.

[177] Khanam R, Ahmed S, Creanga AA, Begum N, Koffi AK, Mahmud A, Rosen H, Baqui AH, Projahnmo Study Group in Bangladesh (2017). Antepartum complications and perinatal mortality in rural Bangladesh. BMC Pregnancy Childbirth.

[178] Knowles SJ, O'Sullivan NP, Meenan AM, Hanniffy R, Robson M (2015). Maternal sepsis incidence, aetiology and outcome for mother and fetus: a prospective study. BJOG.

[179] Shillan D, Sterne JA, Champneys A, Gibbison B (2019). Use of machine learning to analyse routinely collected intensive care unit data: a systematic review. Crit Care.

[180] Mortazavi BJ, Downing NS, Bucholz EM, Dharmarajan K, Manhapra A, Li S, Negahban SN, Krumholz HM (2016). Analysis of machine learning techniques for heart failure readmissions. Circ Cardiovasc Qual Outcomes.

[181] Fernandez-Delgado M, Cernadas E, Barro S, Amorim D (2014). Do we need hundreds of classifiers to solve real world classification problems?. J Mach Learn Res.

[182] Breiman L (2001). Random Forests. Mach Learn.

[183] Fawagreh K, Gaber M, Elyan E (2014). Random forests: from early developments to recent advancements. Syst Sci Control Engin.

[184] Friedman J (2002). Stochastic gradient boosting. Comput Stat Data Analy.

[185] Natekin A, Knoll A (2013). Gradient boosting machines, a tutorial. Front Neurorobot.

[186] Ke G, Meng Q, Finley T, Wang T, Chen W, Ma W, Ye Q (2017). LightGBM: a highly efficient gradient boosting decision tree. Proceedings of the 31st International Conference on Neural Information Processing Systems.

[187] Chen T, Guestrin C (2016). XGBoost: a scalable tree boosting system. Proceedings of the 22nd ACM SIGKDD International Conference on Knowledge Discovery and Data Mining.

[188] Siristatidis C, Pouliakis A, Chrelias C, Kassanos D (2011). Artificial intelligence in IVF: a need. Syst Biol Reprod Med.

[189] Haake KW, List P, Baier D, Zimmermann G, Pretzsch G, Alexander H (1997). [Risk assessment in ovarian hyperstimulation syndrome (OHS) using the machine learning system (Decision Master) in 155 in-vitro fertilisations and embryo-transfer (IVF/ET) cycles with a long stimulation protocol]. Zentralbl Gynakol.

[190] Manna C, Nanni L, Lumini A, Pappalardo S (2013). Artificial intelligence techniques for embryo and oocyte classification. Reprod Biomed Online.

[191] Santos Filho E, Noble J, Poli M, Griffiths T, Emerson G, Wells D (2012). A method for semi-automatic grading of human blastocyst microscope images. Hum Reprod.

[192] Khosravi P, Kazemi E, Zhan Q, Malmsten JE, Toschi M, Zisimopoulos P, Sigaras A, Lavery S, Cooper LA, Hickman C, Meseguer M, Rosenwaks Z, Elemento O, Zaninovic N, Hajirasouliha I (2019). Deep learning enables robust assessment and selection of human blastocysts after in vitro fertilization. NPJ Digit Med.

[193] Liang B, Gao Y, Xu J, Song Y, Xuan L, Shi T, Wang N, Hou Z, Zhao Y, Huang WE, Chen Z (2019). Raman profiling of embryo culture medium to identify aneuploid and euploid embryos. Fertil Steril.

[194] Ratna M, Bhattacharya S, Abdulrahim B, McLernon D (2020). A systematic review of the quality of clinical prediction models in in vitro fertilisation. Hum Reprod.

[195] Doret M, Massoud M, Constans A, Gaucherand P (2011). Use of peripartum ST analysis of fetal electrocardiogram without blood sampling: a large prospective cohort study. Eur J Obstet Gynecol Reprod Biol.

[196] Alexandersson A, Steingrimsdottir T, Terrien J, Marque C, Karlsson B (2015). The Icelandic 16-electrode electrohysterogram database. Sci Data.

[197] Jordan MI, Mitchell TM (2015). Machine learning: trends, perspectives, and prospects. Science.

[198] Kennady G, Kottarathara MJ, Kottarathara AJ, Ajith R, Anandakesavan TM, Ambujam K (2017). Maternal and neonatal outcomes in pregnancy induced hypertension: an observational study. Clin Exp Obstet Gynecol.

[199] van der Ploeg T, Austin PC, Steyerberg EW (2014). Modern modelling techniques are data hungry: a simulation study for predicting dichotomous endpoints. BMC Med Res Methodol.

[200] Meertens LJ, van Montfort P, Scheepers HC, van Kuijk SM, Aardenburg R, Langenveld J, van Dooren IM, Zwaan IM, Spaanderman ME, Smits LJ (2018). Prediction models for the risk of spontaneous preterm birth based on maternal characteristics: a systematic review and independent external validation. Acta Obstet Gynecol Scand.

