# Supplementary material for: Comparison of Multivariable Logistic Regression and Other Machine Learning Algorithms for Prognostic Prediction Studies in Pregnancy Care: Systematic Review and Meta-Analysis
Source: JMIR Med Inform. 2020 Nov 17;8(11):e16503. doi: 10.2196/16503 (PMC7708089; doi:10.2196/16503)
Supplement: Multimedia Appendix 1 [file medinform_v8i11e16503_app1.docx]

**MULTIMEDIA APPENDIX 1**

Details on forest plots, search filter, eligibility criteria, study selection, list of reviewed studies, risk of bias assessment, signaling questions and the answers, predictive performance and sample size, R code for meta-analysis, and records of studies.

Comparison of multivariable logistic regression and other machine learning algorithms for prognostic prediction studies in pregnancy care: systematic review and meta-analysis

Herdiantri Sufriyana^1,2^, MD, MSc; Atina Husnayain^1,3^, MPH; Ya-Lin Chen^1,4^, Pharm.D; Chao-Yang Kuo^1^, MSc; Onkar Singh^5,6^, MSc; Tso-Yang Yeh^7^; Yu-Wei Wu^1,8^, PhD; Emily Chia-Yu Su^1,8^, PhD

^1^Graduate Institute of Biomedical Informatics, College of Medical Science and Technology, Taipei Medical University, Taipei, Taiwan.

^2^Department of Medical Physiology, College of Medicine, University of Nahdlatul Ulama Surabaya, Surabaya, Indonesia.

^3^Department of Biostatistics, Epidemiology, and Population Health, Faculty of Medicine, Public Health and Nursing, Universitas Gadjah Mada, Yogyakarta, Indonesia.

^4^College of Pharmacy, Taipei Medical University, Taiwan.

^5^Bioinformatics Program, Taiwan International Graduate Program, Institute of Information Science, Academia Sinica, Taipei, Taiwan.

^6^Institute of Biomedical Informatics, National Yang-Ming University, Taipei, Taiwan.

^7^School of Dentistry, College of Oral Medicine, Taipei Medical University, Taipei, Taiwan.

^8^Clinical Big Data Research Center, Taipei Medical University Hospital, Taipei, Taiwan.

Corresponding author:

Emily Chia-Yu Su, PhD

Graduate Institute of Biomedical Informatics

College of Medical Science and Technology

Taipei Medical University

250 Wu-Xing Street

Taipei 11031

Taiwan

Phone: 886 2 663 82736 ext 1515

Email: emilysu@tmu.edu.tw

Table of Contents

[Figure S1. Forest plot of logit AUROC differences for each random-effects model (1). 2](#_Toc49542494)

[Figure S2. Forest plot of logit AUROC differences for each random-effects model (2). 3](#_Toc49542495)

[Figure S3. Forest plot of logit AUROC differences for each random-effects model (3). 4](#_Toc49542496)

[Figure S4. Forest plot of logit AUROC differences for each random-effects model (4). 5](#_Toc49542497)

[Table S1. Search filter. 6](#_Toc49542498)

[Table S2. Eligibility criteria description. 10](#_Toc49542499)

[Table S3. Study selection for eligibility, applicability, and meta-analysis. ^a^ 13](#_Toc49542500)

[Table S4. List of studies for full text review. ^a, b^ 47](#_Toc49542501)

[Table S5. Description of eligible studies. 83](#_Toc49542502)

[Table S6. Risk of bias assessment. 96](#_Toc49542503)

[Table S7. Signaling questions. 100](#_Toc49542504)

[Table S8. Answer for each signaling question. 101](#_Toc49542505)

[Table S9. Predictive performance and sample size. 112](#_Toc49542506)

[Table S10. R code for meta-analysis. 124](#_Toc49542507)

[Table S11. Unduplicated and refined records of studies. 128](#_Toc49542508)


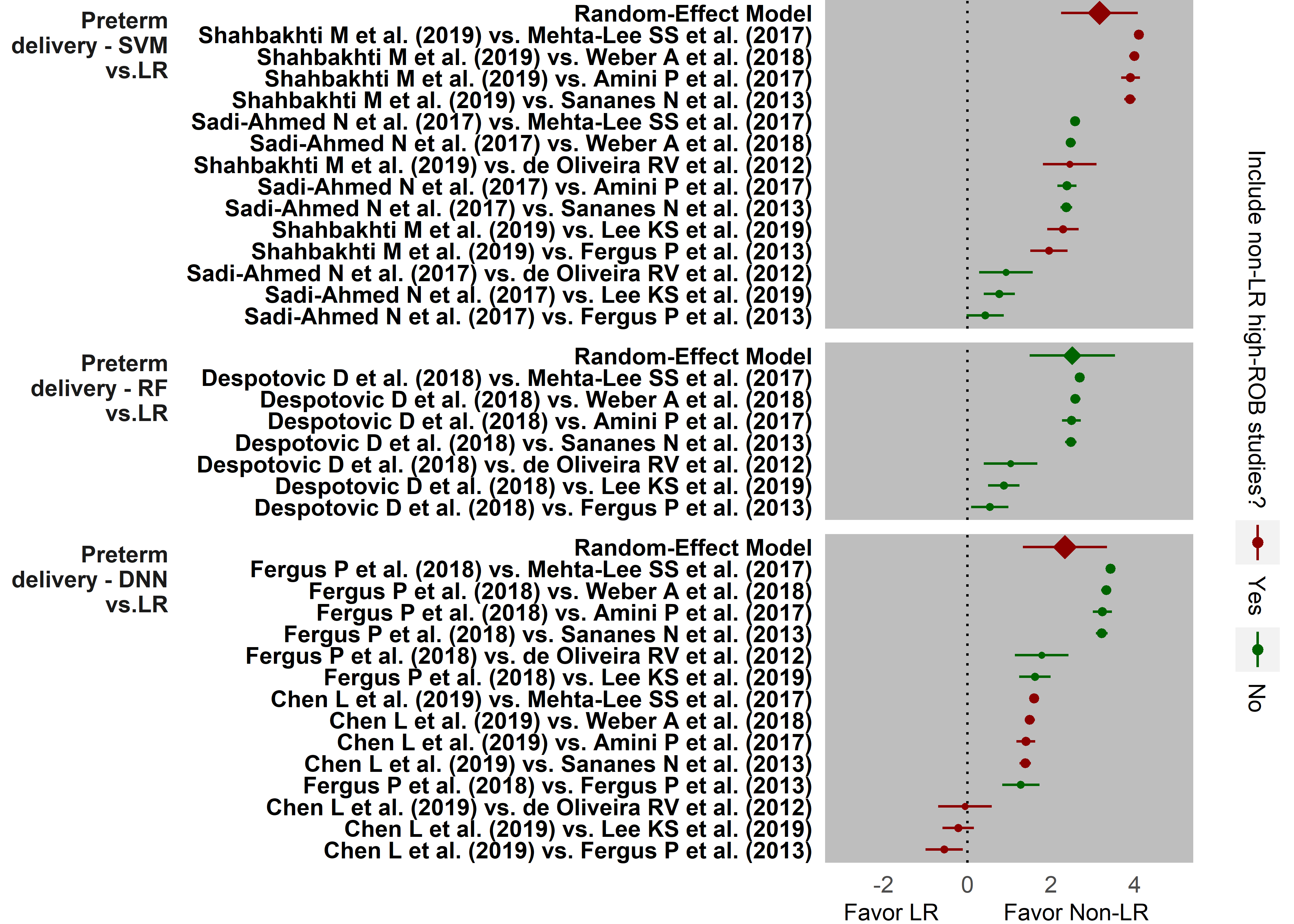


# Figure S1. Forest plot of logit area under the receiver operating characteristics curve (AUROC) differences for each random-effects model (1). This plot included those of preterm delivery from comparisons of support vector machine (SVM) vs. logistic regression (LR), random forest (RF) vs. LR, and deep neural network (DNN) vs. LR. The lines are ranges of the 95% confidence intervals (CIs) with diamonds or circles whose sizes were determined by the sample size. Colors of the boxes and lines indicate a high risk of bias (ROB). Please download the dataset [here](https://drive.google.com/file/d/1WTLAvp7WTSYZn0rB7ZvQ6w8VfgKbn9uK/view?usp=sharing). LR, logistic regression; SVM, support vector machine; DNN, deep neural network; RF, random forest.


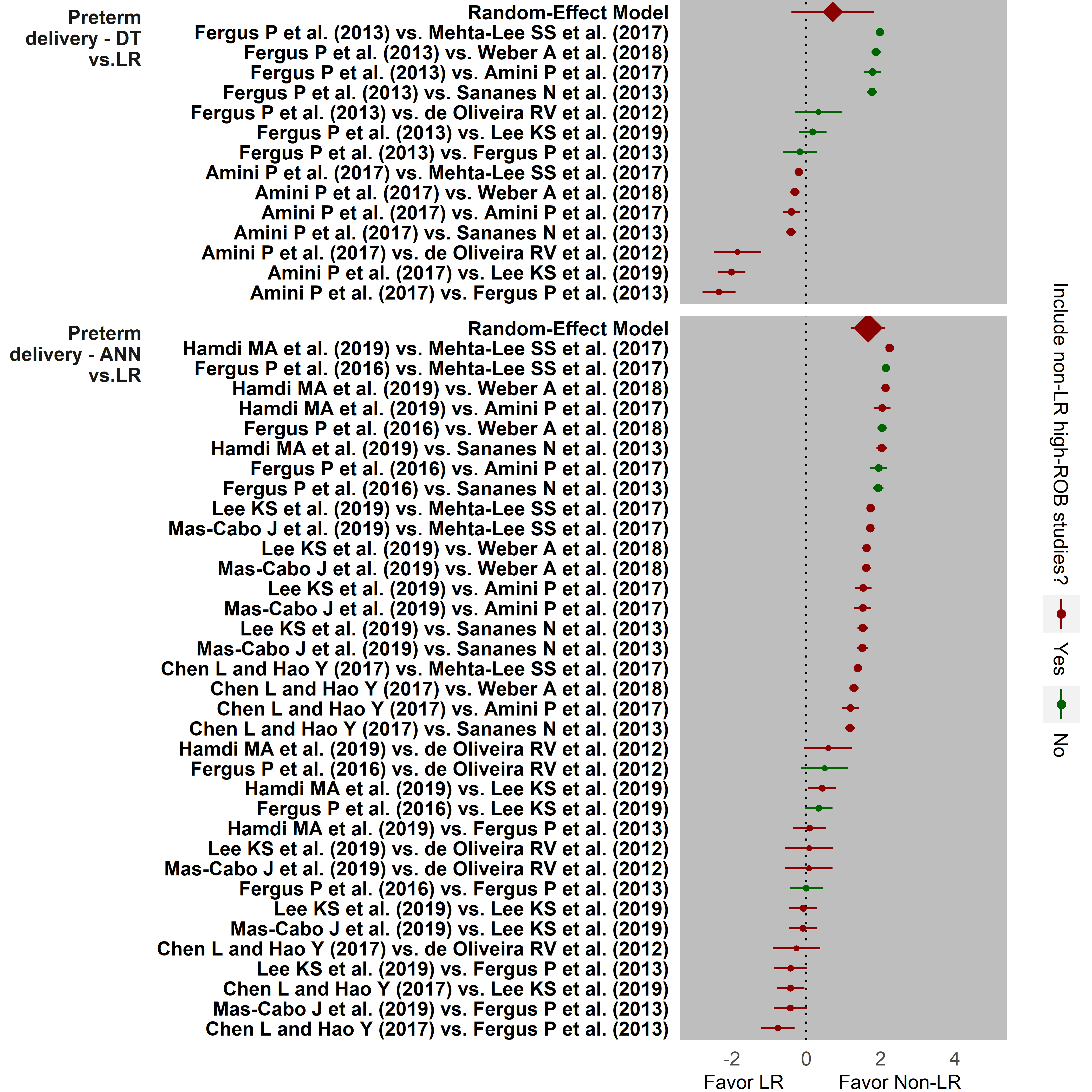


# Figure S2. Forest plot of logit area under the receiver operating characteristics curve (AUROC) differences for each random-effects model (2). This plot included those of preterm delivery from comparison of decision trees (DTs) vs. logistic regressions (LRs) and artificial neural networks (ANNs) vs. LRs. The lines are ranges of the 95% confidence intervals (CIs) with diamonds or circles whose sizes were determined by the sample size. Colors of the boxes and lines indicate a high risk of bias (ROB). Please download the dataset [here](https://drive.google.com/file/d/1WTLAvp7WTSYZn0rB7ZvQ6w8VfgKbn9uK/view?usp=sharing). LR, logistic regression; DT, decision tree; ANN, artificial neural network.


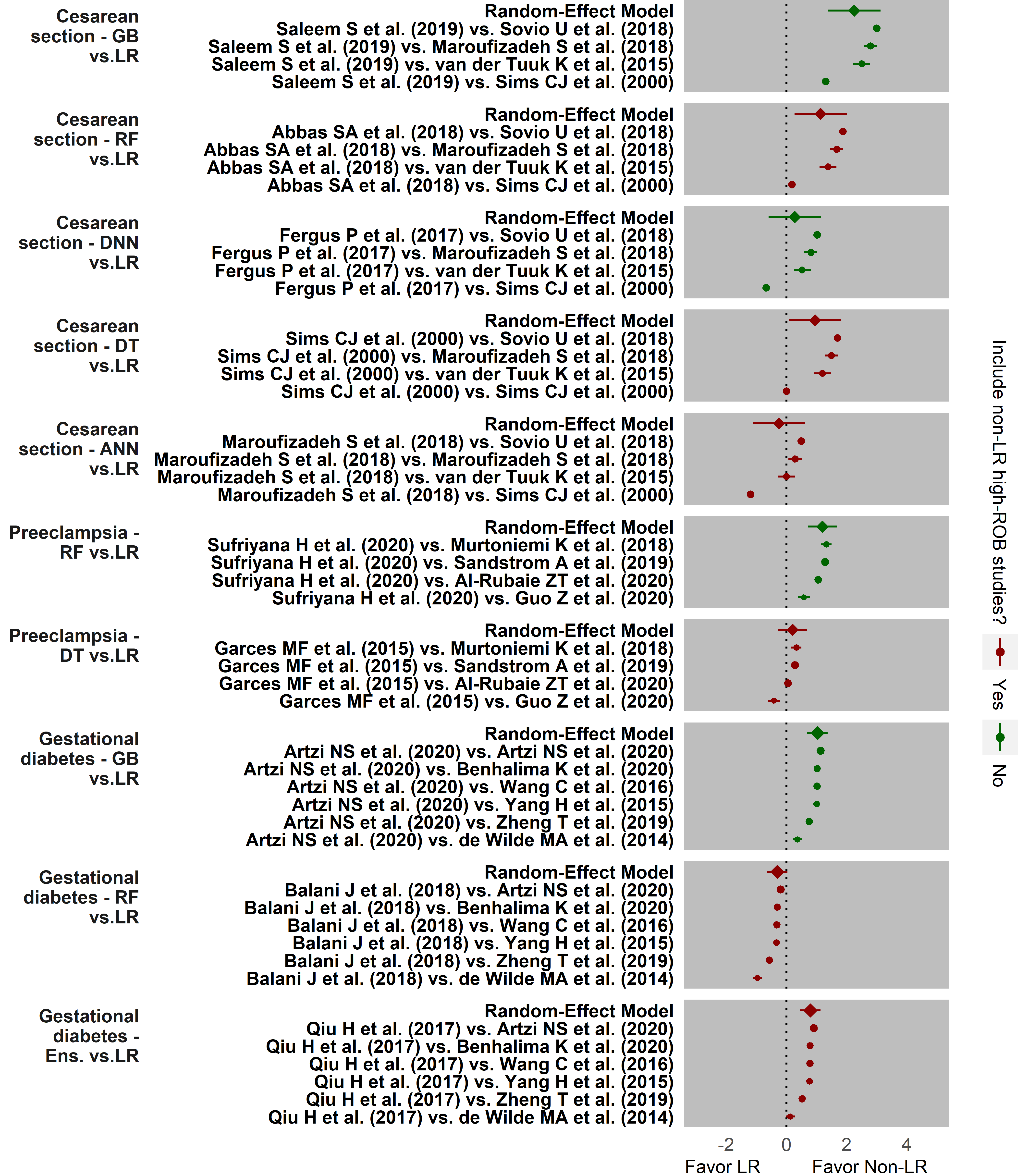


# Figure S3. Forest plot of logit area under the receiver operating characteristics curve (AUROC) differences for each random-effects model (3). This plot included those of cesarean section, preeclampsia, and gestational diabetes. The lines are ranges for the 95% confidence intervals (CIs) with diamonds or circles whose sizes were determined by the sample size. Colors of the boxes and lines indicate a high risk of bias (ROB). Please download the dataset [here](https://drive.google.com/file/d/1WTLAvp7WTSYZn0rB7ZvQ6w8VfgKbn9uK/view?usp=sharing). LR, logistic regression; RF, random forest; DT, decision tree; ANN, artificial neural network; GB, gradient boosting; Ens., ensemble of multiple algorithms.


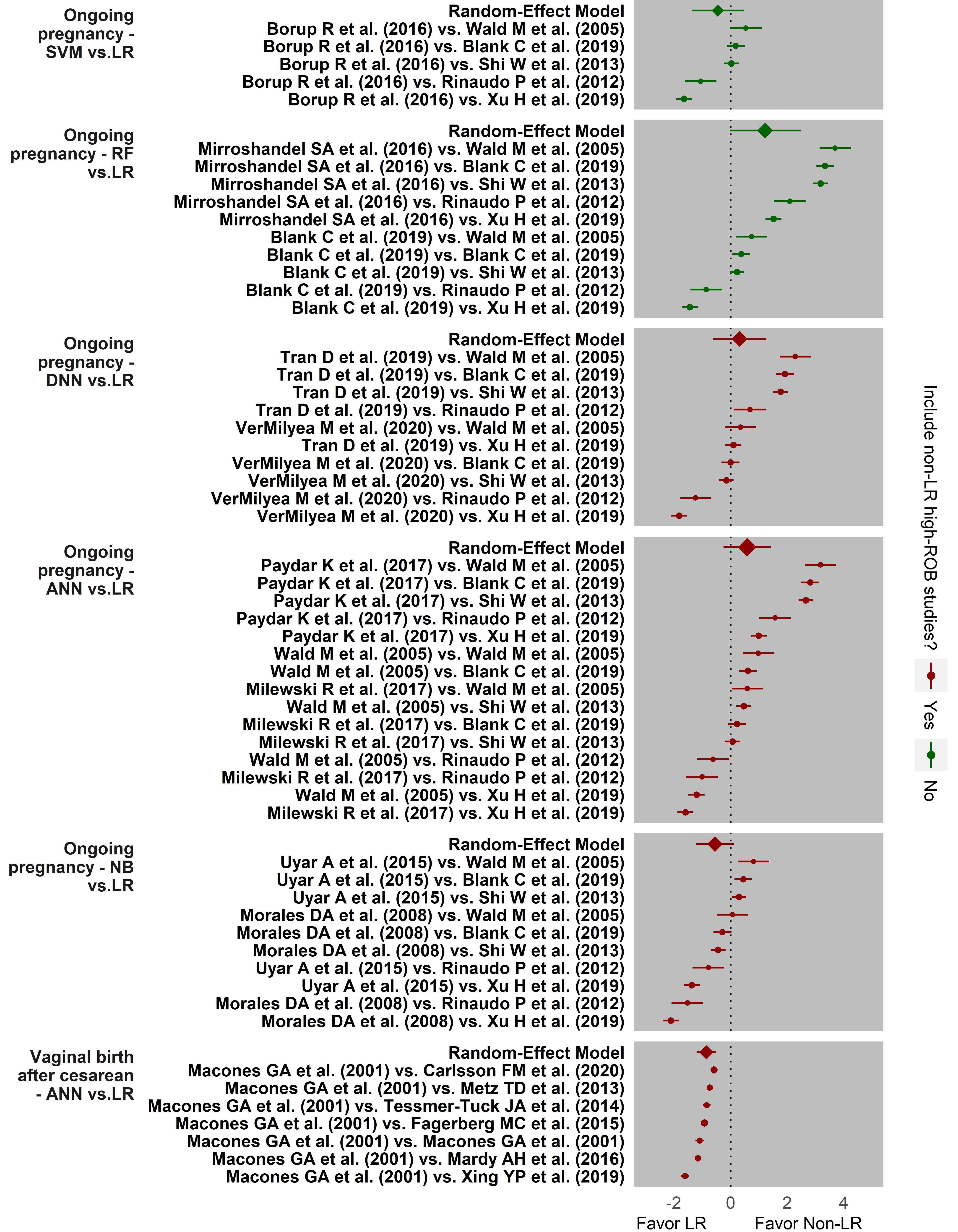


# Figure S4. Forest plot of logit area under the receiver characteristic curve (AUROC) differences for each random-effects model (4). This plot included those of ongoing pregnancy and vaginal birth after a cesarean section. The lines are ranges for the 95% confidence intervals (CIs) with diamonds or circles whose sizes were determined by the sample size. Colors of the boxes and lines indicate a high risk of bias (ROB). Please download the dataset [here](https://drive.google.com/file/d/1WTLAvp7WTSYZn0rB7ZvQ6w8VfgKbn9uK/view?usp=sharing). LR, logistic regression; SVM, support vector machine; DNN, deep neural network; RF, random forest; ANN, artificial neural network; GB, gradient boosting; NB, naïve Bayes.

# Table S1. Search filter.

| Keywords |
| --- |
|  |
| machine learning AND pregnancy |
| machine learning AND (central nervous system vascular malformations NOT pregnancy) |
| machine learning AND (cesarean section NOT pregnancy) |
| machine learning AND (congenital heart defects NOT pregnancy) |
| machine learning AND (embryo implantation NOT pregnancy) |
| machine learning AND (fertilization *in vitro* NOT pregnancy) |
| machine learning AND (fetal development NOT pregnancy) |
| machine learning AND (fetal distress NOT pregnancy) |
| machine learning AND (gestational diabetes NOT pregnancy) |
| machine learning AND infectious pregnancy complications |
| machine learning AND (low birth weight infant NOT pregnancy) |
| machine learning AND (obstetric labor NOT pregnancy) |
| machine learning AND pregnancy rate |
| machine learning AND pregnancy-induced hypertension |
| machine learning AND (premature birth NOT pregnancy) |
| machine learning AND (small for gestational age infant NOT pregnancy) |
| machine learning AND (stillbirth NOT pregnancy) |
| machine learning AND (trisomy NOT pregnancy) |
| decision tree AND pregnancy |
| decision tree AND (central nervous system vascular malformations NOT pregnancy) |
| decision tree AND (cesarean section NOT pregnancy) |
| decision tree AND (congenital heart defects NOT pregnancy) |
| decision tree AND (embryo implantation NOT pregnancy) |
| decision tree AND (fertilization *in vitro* NOT pregnancy) |
| decision tree AND (fetal development NOT pregnancy) |
| decision tree AND (fetal distress NOT pregnancy) |
| decision tree AND (gestational diabetes NOT pregnancy) |
| decision tree AND infectious pregnancy complications |
| decision tree AND (low birth weight infant NOT pregnancy) |
| decision tree AND (obstetric labor NOT pregnancy) |
| decision tree AND pregnancy rate |
| decision tree AND pregnancy-induced hypertension |
| decision tree AND (premature birth NOT pregnancy) |
| decision tree AND (small for gestational age infant NOT pregnancy) |
| decision tree AND (stillbirth NOT pregnancy) |
| decision tree AND (trisomy NOT pregnancy) |
| artificial neural network AND pregnancy |
| artificial neural network AND (central nervous system vascular malformations NOT pregnancy) |
| artificial neural network AND (cesarean section NOT pregnancy) |
| artificial neural network AND (congenital heart defects NOT pregnancy) |
| artificial neural network AND (embryo implantation NOT pregnancy) |
| artificial neural network AND (fertilization *in vitro* NOT pregnancy) |
| artificial neural network AND (fetal development NOT pregnancy) |
| artificial neural network AND (fetal distress NOT pregnancy) |
| artificial neural network AND (gestational diabetes NOT pregnancy) |
| artificial neural network AND infectious pregnancy complications |
| artificial neural network AND (low birth weight infant NOT pregnancy) |
| artificial neural network AND (obstetric labor NOT pregnancy) |
| artificial neural network AND pregnancy rate |
| artificial neural network AND pregnancy-induced hypertension |
| artificial neural network AND (premature birth NOT pregnancy) |
| artificial neural network AND (small for gestational age infant NOT pregnancy) |
| artificial neural network AND (stillbirth NOT pregnancy) |
| artificial neural network AND (trisomy NOT pregnancy) |
| support vector machine AND pregnancy |
| support vector machine AND (central nervous system vascular malformations NOT pregnancy) |
| support vector machine AND (cesarean section NOT pregnancy) |
| support vector machine AND (congenital heart defects NOT pregnancy) |
| support vector machine AND (embryo implantation NOT pregnancy) |
| support vector machine AND (fertilization *in vitro* NOT pregnancy) |
| support vector machine AND (fetal development NOT pregnancy) |
| support vector machine AND (fetal distress NOT pregnancy) |
| support vector machine AND (gestational diabetes NOT pregnancy) |
| support vector machine AND infectious pregnancy complications |
| support vector machine AND (low birth weight infant NOT pregnancy) |
| support vector machine AND (obstetric labor NOT pregnancy) |
| support vector machine AND pregnancy rate |
| support vector machine AND pregnancy-induced hypertension |
| support vector machine AND (premature birth NOT pregnancy) |
| support vector machine AND (small for gestational age infant NOT pregnancy) |
| support vector machine AND (stillbirth NOT pregnancy) |
| support vector machine AND (trisomy NOT pregnancy) |
| random forests AND pregnancy |
| random forests AND (central nervous system vascular malformations NOT pregnancy) |
| random forests AND (cesarean section NOT pregnancy) |
| random forests AND (congenital heart defects NOT pregnancy) |
| random forests AND (embryo implantation NOT pregnancy) |
| random forests AND (fertilization *in vitro* NOT pregnancy) |
| random forests AND (fetal development NOT pregnancy) |
| random forests AND (fetal distress NOT pregnancy) |
| random forests AND (gestational diabetes NOT pregnancy) |
| random forests AND infectious pregnancy complications |
| random forests AND (low birth weight infant NOT pregnancy) |
| random forests AND (obstetric labor NOT pregnancy) |
| random forests AND pregnancy rate |
| random forests AND pregnancy-induced hypertension |
| random forests AND (premature birth NOT pregnancy) |
| random forests AND (small for gestational age infant NOT pregnancy) |
| random forests AND (stillbirth NOT pregnancy) |
| random forests AND (trisomy NOT pregnancy) |
| artificial intelligence AND pregnancy |
| artificial intelligence AND (central nervous system vascular malformations NOT pregnancy) |
| artificial intelligence AND (cesarean section NOT pregnancy) |
| artificial intelligence AND (congenital heart defects NOT pregnancy) |
| artificial intelligence AND (embryo implantation NOT pregnancy) |
| artificial intelligence AND (fertilization *in vitro* NOT pregnancy) |
| artificial intelligence AND (fetal development NOT pregnancy) |
| artificial intelligence AND (fetal distress NOT pregnancy) |
| artificial intelligence AND (gestational diabetes NOT pregnancy) |
| artificial intelligence AND infectious pregnancy complications |
| artificial intelligence AND (low birth weight infant NOT pregnancy) |
| artificial intelligence AND (obstetric labor NOT pregnancy) |
| artificial intelligence AND pregnancy rate |
| artificial intelligence AND pregnancy-induced hypertension |
| artificial intelligence AND (premature birth NOT pregnancy) |
| artificial intelligence AND (small for gestational age infant NOT pregnancy) |
| artificial intelligence AND (stillbirth NOT pregnancy) |
| artificial intelligence AND (trisomy NOT pregnancy) |
| deep learning AND pregnancy |
| deep learning AND (central nervous system vascular malformations NOT pregnancy) |
| deep learning AND (cesarean section NOT pregnancy) |
| deep learning AND (congenital heart defects NOT pregnancy) |
| deep learning AND (embryo implantation NOT pregnancy) |
| deep learning AND (fertilization *in vitro* NOT pregnancy) |
| deep learning AND (fetal development NOT pregnancy) |
| deep learning AND (fetal distress NOT pregnancy) |
| deep learning AND (gestational diabetes NOT pregnancy) |
| deep learning AND infectious pregnancy complications |
| deep learning AND (low birth weight infant NOT pregnancy) |
| deep learning AND (obstetric labor NOT pregnancy) |
| deep learning AND pregnancy rate |
| deep learning AND pregnancy-induced hypertension |
| deep learning AND (premature birth NOT pregnancy) |
| deep learning AND (small for gestational age infant NOT pregnancy) |
| deep learning AND (stillbirth NOT pregnancy) |
| deep learning AND (trisomy NOT pregnancy) |
| logistic regression multivariable prediction AND pregnancy |
| logistic regression multivariable prediction AND (central nervous system vascular malformations NOT pregnancy) |
| logistic regression multivariable prediction AND (cesarean section NOT pregnancy) |
| logistic regression multivariable prediction AND (congenital heart defects NOT pregnancy) |
| logistic regression multivariable prediction AND (embryo implantation NOT pregnancy) |
| logistic regression multivariable prediction AND (fertilization *in vitro* NOT pregnancy) |
| logistic regression multivariable prediction AND (fetal development NOT pregnancy) |
| logistic regression multivariable prediction AND (fetal distress NOT pregnancy) |
| logistic regression multivariable prediction AND (gestational diabetes NOT pregnancy) |
| logistic regression multivariable prediction AND infectious pregnancy complications |
| logistic regression multivariable prediction AND (low birth weight infant NOT pregnancy) |
| logistic regression multivariable prediction AND (obstetric labor NOT pregnancy) |
| logistic regression multivariable prediction AND pregnancy rate |
| logistic regression multivariable prediction AND pregnancy-induced hypertension |
| logistic regression multivariable prediction AND (premature birth NOT pregnancy) |
| logistic regression multivariable prediction AND (small for gestational age infant NOT pregnancy) |
| logistic regression multivariable prediction AND (stillbirth NOT pregnancy) |
| logistic regression multivariable prediction AND (trisomy NOT pregnancy) |

# Table S2. Eligibility criteria description.

| Step | Section | Topic | Criterion | Source | Column name (Table S3) |
| --- | --- | --- | --- | --- | --- |
|  |  |  |  |  |  |
| 1 | Common | Language and accessibility | English and accessible | - | English/Accessible |
| 2 | Methods | Source of data | Describe the study design or source of data (e.g., randomized trial, cohort, or registry data), separately for the development and validation data sets, if applicable. | TRIPOD | Data Source |
| 3 | Methods | Outcome | Clearly define the outcome that is predicted by the prediction model, including how and when assessed. | TRIPOD | Outcome |
| 4 | Methods | Prediction problem | Defining quality metrics to assess model performance. | TRIPOD; MLP-BIOM | Evaluation Metric |
| 5 | Methods | Predictors | Clearly define all predictors used in developing or validating the multivariable prediction model, including how and when they were measured. Extra caution is needed to prevent information leakage from the response variable to predictor variables. | TRIPOD; MLP-BIOM | Predictors |
| 6 | Methods | Statistical analysis methods | Describe the basic statistics of the dataset, particularly of the response variable. These include the ratio of positive to negative classes for a classification problem and the distribution of the response variable for regression problem. | MLP-BIOM | Descriptive Statistics |
| 7 | Methods | Model building | Assess whether sufficient data are available for a good fit of the model. In particular, for classification, there should be a sufficient number of observations in both positive and negative classes. | MLP-BIOM | Event Sample Size |
| 8 | Methods | Model building | Determine a set of candidate modeling techniques (*e.g.* logistic regression, random forest, or deep learning). If only one type of model was used, justify the decision for using that model. | TRIPOD; MLP-BIOM | Modeling Method |
| 9 | Methods | Validation technique | Define the model validation strategies. Internal validation is the minimum requirement; external validation should also be performed whenever possible. | TRIPOD; MLP-BIOM | Model Validation |
| 10 | Methods | Validation technique | Development (D): Prediction model development without external validation. These studies may include internal validation methods, such as bootstrapping and cross-validation techniques. Development and validation (DV): Prediction model development combined with external validation in other participants in the same article. Validation (V): External validation of existing (previously developed) model in other participants. | PROBAST | Type of Study |
| 11 | Methods | Validation technique | For regression problems, the normalized root-mean-square error should be used. For classification problems, the metrics should include sensitivity, specificity, positive predictive value, negative predictive value, area under the ROC curve, and calibration plot | MLP-BIOM | Recommended Metrics |
| 12 | Applicability | Applicability | If low concerns regarding applicability for all domains, the prediction model evaluation is judged to have low concerns regarding applicability. | PROBAST | Applicability |
| 13 | Results | Model performance | If possible, report the parameter estimates in the model and their confidence intervals. When the direct calculation of confidence intervals is not possible, report nonparametric estimates from bootstrap samples. If there was at least three LR models and a non-LR model from any studies for an outcome, all of the studies with that outcome were included in meta-analysis. This is determined based on requirement of minimum data points to calculate variance as part of meta-analysis procedure. If studies did not report AUROC, we estimated that from the sensitivity and specificity using trapezoidal rule | TRIPOD; MLP-BIOM; defined by authors | Meta-analysis |

TRIPOD, transparent reporting of a multivariable prediction model for individual prognosis or diagnosis; MLP-BIOM, guidelines for developing and reporting machine learning predictive models in biomedical research; PROBAST, prediction model risk of bias assessment tool.

# Table S3. Study selection for eligibility, applicability, and meta-analysis. ^a^

| # | Author (Year)  *n*=680 | Type of Study | English/ Accessible  *n*=625 | Data Source  *n*=615 | Outcome  *n*=600 | Evaluation Metric  *n*=551 | Predictors  *n*=543 | Descriptive Statistics  *n*=430 | Event Sample Size  *n*=422 | Modeling Method  *n*=419 | Model Validation  *n*=275 | Recommended Metrics  *n*=256 | Applicability  *n*=142 | Meta-analysis  *n*=62 |
| --- | --- | --- | --- | --- | --- | --- | --- | --- | --- | --- | --- | --- | --- | --- |
|  |  |  |  |  |  |  |  |  |  |  |  |  |  |  |
| 1 | Artzi, et al. (2020) | DV | Yes | Yes | Yes | Yes | Yes | Yes | Yes | Yes | Yes | Yes | Yes | Yes |
| 2 | Benhalima, et al. (2020) | D | Yes | Yes | Yes | Yes | Yes | Yes | Yes | Yes | Yes | Yes | Yes | Yes |
| 3 | Blank C, et al. (2019) | D | Yes | Yes | Yes | Yes | Yes | Yes | Yes | Yes | Yes | Yes | Yes | Yes |
| 4 | Borup R, et al. (2016) | DV | Yes | Yes | Yes | Yes | Yes | Yes | Yes | Yes | Yes | Yes | Yes | Yes |
| 5 | de Wilde, et al. (2014) | D | Yes | Yes | Yes | Yes | Yes | Yes | Yes | Yes | Yes | Yes | Yes | Yes |
| 6 | Mardy, et al. (2016) | D | Yes | Yes | Yes | Yes | Yes | Yes | Yes | Yes | Yes | Yes | Yes | Yes |
| 7 | Maroufizadeh, et al. (2018) | D | Yes | Yes | Yes | Yes | Yes | Yes | Yes | Yes | Yes | Yes | Yes | Yes |
| 8 | Milewski, et al. (2013) | D | Yes | Yes | Yes | Yes | Yes | Yes | Yes | Yes | Yes | Yes | Yes | Yes |
| 9 | Rinaudo, et al. (2012) | D | Yes | Yes | Yes | Yes | Yes | Yes | Yes | Yes | Yes | Yes | Yes | Yes |
| 10 | Sananes, et al. (2013) | DV | Yes | Yes | Yes | Yes | Yes | Yes | Yes | Yes | Yes | Yes | Yes | Yes |
| 11 | Sandström, et al. (2019) | D | Yes | Yes | Yes | Yes | Yes | Yes | Yes | Yes | Yes | Yes | Yes | Yes |
| 12 | Shi, et al. (2013) | D | Yes | Yes | Yes | Yes | Yes | Yes | Yes | Yes | Yes | Yes | Yes | Yes |
| 13 | Sufriyana, et al. (2020) | DV | Yes | Yes | Yes | Yes | Yes | Yes | Yes | Yes | Yes | Yes | Yes | Yes |
| 14 | Tessmer-Tuck, et al. (2014) | DV | Yes | Yes | Yes | Yes | Yes | Yes | Yes | Yes | Yes | Yes | Yes | Yes |
| 15 | Thériault, et al. (2016) | D | Yes | Yes | Yes | Yes | Yes | Yes | Yes | Yes | Yes | Yes | Yes | Yes |
| 16 | Tran, et al. (2019) | D | Yes | Yes | Yes | Yes | Yes | Yes | Yes | Yes | Yes | Yes | Yes | Yes |
| 17 | Troisi J, et al. (2018) | D | Yes | Yes | Yes | Yes | Yes | Yes | Yes | Yes | Yes | Yes | Yes | Yes |
| 18 | van Baaren, et al. (2015) | D | Yes | Yes | Yes | Yes | Yes | Yes | Yes | Yes | Yes | Yes | Yes | Yes |
| 19 | van der Tuuk, et al. (2015) | D | Yes | Yes | Yes | Yes | Yes | Yes | Yes | Yes | Yes | Yes | Yes | Yes |
| 20 | Weber, et al. (2018) | D | Yes | Yes | Yes | Yes | Yes | Yes | Yes | Yes | Yes | Yes | Yes | Yes |
| 21 | Xing, et al. (2019) | D | Yes | Yes | Yes | Yes | Yes | Yes | Yes | Yes | Yes | Yes | Yes | Yes |
| 22 | Yang, et al. (2015) | D | Yes | Yes | Yes | Yes | Yes | Yes | Yes | Yes | Yes | Yes | Yes | Yes |
| 23 | Zheng, et al. (2019) | D | Yes | Yes | Yes | Yes | Yes | Yes | Yes | Yes | Yes | Yes | Yes | Yes |
| 24 | Abbas SA, et al. (2018) | D | Yes | Yes | Yes | Yes | Yes | Yes | Yes | Yes | Yes | Yes | Yes | Yes |
| 25 | Amini, et al. (2017) | D | Yes | Yes | Yes | Yes | Yes | Yes | Yes | Yes | Yes | Yes | Yes | Yes |
| 26 | Balani, et al. (2018) | D | Yes | Yes | Yes | Yes | Yes | Yes | Yes | Yes | Yes | Yes | Yes | Yes |
| 27 | Bastek, et al. (2012) | D | Yes | Yes | Yes | Yes | Yes | Yes | Yes | Yes | Yes | Yes | Yes | Yes |
| 28 | Carlsson Fagerberg, et al. (2020) | D | Yes | Yes | Yes | Yes | Yes | Yes | Yes | Yes | Yes | Yes | Yes | Yes |
| 29 | Chen L, et al. (2017) | D | Yes | Yes | Yes | Yes | Yes | Yes | Yes | Yes | Yes | Yes | Yes | Yes |
| 30 | Chen, et al. (2019) | D | Yes | Yes | Yes | Yes | Yes | Yes | Yes | Yes | Yes | Yes | Yes | Yes |
| 31 | de Oliveira, et al. (2012) | D | Yes | Yes | Yes | Yes | Yes | Yes | Yes | Yes | Yes | Yes | Yes | Yes |
| 32 | Despotovic D, et al. (2018) | D | Yes | Yes | Yes | Yes | Yes | Yes | Yes | Yes | Yes | Yes | Yes | Yes |
| 33 | Fagerberg, et al. (2015) | DV | Yes | Yes | Yes | Yes | Yes | Yes | Yes | Yes | Yes | Yes | Yes | Yes |
| 34 | Fergus P, et al. (2013) | D | Yes | Yes | Yes | Yes | Yes | Yes | Yes | Yes | Yes | Yes | Yes | Yes |
| 35 | Fergus P, et al. (2016) | D | Yes | Yes | Yes | Yes | Yes | Yes | Yes | Yes | Yes | Yes | Yes | Yes |
| 36 | Fergus P, et al. (2017) | D | Yes | Yes | Yes | Yes | Yes | Yes | Yes | Yes | Yes | Yes | Yes | Yes |
| 37 | Fergus P, et al. (2018) | D | Yes | Yes | Yes | Yes | Yes | Yes | Yes | Yes | Yes | Yes | Yes | Yes |
| 38 | Fiset S, et al. (2019) | D | Yes | Yes | Yes | Yes | Yes | Yes | Yes | Yes | Yes | Yes | Yes | Yes |
| 39 | Garcés, et al. (2015) | D | Yes | Yes | Yes | Yes | Yes | Yes | Yes | Yes | Yes | Yes | Yes | Yes |
| 40 | Guo, et al. (2020) | DV | Yes | Yes | Yes | Yes | Yes | Yes | Yes | Yes | Yes | Yes | Yes | Yes |
| 41 | Hamdi, et al. (2019) | D | Yes | Yes | Yes | Yes | Yes | Yes | Yes | Yes | Yes | Yes | Yes | Yes |
| 42 | Lee, et al. (2019) | D | Yes | Yes | Yes | Yes | Yes | Yes | Yes | Yes | Yes | Yes | Yes | Yes |
| 43 | Macones, et al. (2001) | D | Yes | Yes | Yes | Yes | Yes | Yes | Yes | Yes | Yes | Yes | Yes | Yes |
| 44 | Mas-Cabo, et al. (2019) | D | Yes | Yes | Yes | Yes | Yes | Yes | Yes | Yes | Yes | Yes | Yes | Yes |
| 45 | Mehta-Lee, et al. (2017) | D | Yes | Yes | Yes | Yes | Yes | Yes | Yes | Yes | Yes | Yes | Yes | Yes |
| 46 | Menon R, et al. (2014) | D | Yes | Yes | Yes | Yes | Yes | Yes | Yes | Yes | Yes | Yes | Yes | Yes |
| 47 | Mirroshandel SA, et al. (2016) | D | Yes | Yes | Yes | Yes | Yes | Yes | Yes | Yes | Yes | Yes | Yes | Yes |
| 48 | Morales, et al. (2008) | D | Yes | Yes | Yes | Yes | Yes | Yes | Yes | Yes | Yes | Yes | Yes | Yes |
| 49 | Murtoniemi, et al. (2018) | D | Yes | Yes | Yes | Yes | Yes | Yes | Yes | Yes | Yes | Yes | Yes | Yes |
| 50 | Paydar, et al. (2017) | D | Yes | Yes | Yes | Yes | Yes | Yes | Yes | Yes | Yes | Yes | Yes | Yes |
| 51 | Qiu H, et al. (2017) | D | Yes | Yes | Yes | Yes | Yes | Yes | Yes | Yes | Yes | Yes | Yes | Yes |
| 52 | Sadi-Ahmed N, et al. (2017) | D | Yes | Yes | Yes | Yes | Yes | Yes | Yes | Yes | Yes | Yes | Yes | Yes |
| 53 | Saleem S, et al. (2019) | D | Yes | Yes | Yes | Yes | Yes | Yes | Yes | Yes | Yes | Yes | Yes | Yes |
| 54 | Shahbakhti, et al. (2019) | D | Yes | Yes | Yes | Yes | Yes | Yes | Yes | Yes | Yes | Yes | Yes | Yes |
| 55 | Sims, et al. (2000) | D | Yes | Yes | Yes | Yes | Yes | Yes | Yes | Yes | Yes | Yes | Yes | Yes |
| 56 | Sovio, et al. (2018) | D | Yes | Yes | Yes | Yes | Yes | Yes | Yes | Yes | Yes | Yes | Yes | Yes |
| 57 | Uyar A, et al. (2015) | D | Yes | Yes | Yes | Yes | Yes | Yes | Yes | Yes | Yes | Yes | Yes | Yes |
| 58 | VerMilyea, et al. (2020) | D | Yes | Yes | Yes | Yes | Yes | Yes | Yes | Yes | Yes | Yes | Yes | Yes |
| 59 | Wald, et al. (2005) | D | Yes | Yes | Yes | Yes | Yes | Yes | Yes | Yes | Yes | Yes | Yes | Yes |
| 60 | Wang, et al. (2016) | D | Yes | Yes | Yes | Yes | Yes | Yes | Yes | Yes | Yes | Yes | Yes | Yes |
| 61 | Xu, et al. (2019) | D | Yes | Yes | Yes | Yes | Yes | Yes | Yes | Yes | Yes | Yes | Yes | Yes |
| 62 | Yang, et al. (2019) | D | Yes | Yes | Yes | Yes | Yes | Yes | Yes | Yes | Yes | Yes | Yes | Yes |
| 63 | Agopian, et al. (2012) | D | Yes | Yes | Yes | Yes | Yes | Yes | Yes | Yes | Yes | Yes | Yes | No |
| 64 | Almeida, et al. (2017) | DV | Yes | Yes | Yes | Yes | Yes | Yes | Yes | Yes | Yes | Yes | Yes | No |
| 65 | Al-Rubaie, et al. (2020) | DV | Yes | Yes | Yes | Yes | Yes | Yes | Yes | Yes | Yes | Yes | Yes | No |
| 66 | Chandrasekaran, et al. (2016) | D | Yes | Yes | Yes | Yes | Yes | Yes | Yes | Yes | Yes | Yes | Yes | No |
| 67 | Chen, et al. (2018) | DV | Yes | Yes | Yes | Yes | Yes | Yes | Yes | Yes | Yes | Yes | Yes | No |
| 68 | Ciobanu, et al. (2019) | D | Yes | Yes | Yes | Yes | Yes | Yes | Yes | Yes | Yes | Yes | Yes | No |
| 69 | Cömert, et al. (2018) | D | Yes | Yes | Yes | Yes | Yes | Yes | Yes | Yes | Yes | Yes | Yes | No |
| 70 | Coppedè, et al. (2010) | D | Yes | Yes | Yes | Yes | Yes | Yes | Yes | Yes | Yes | Yes | Yes | No |
| 71 | Cortet, et al. (2015) | D | Yes | Yes | Yes | Yes | Yes | Yes | Yes | Yes | Yes | Yes | Yes | No |
| 72 | Crovetto, et al. (2015) | D | Yes | Yes | Yes | Yes | Yes | Yes | Yes | Yes | Yes | Yes | Yes | No |
| 73 | Eggebø, et al. (2015) | D | Yes | Yes | Yes | Yes | Yes | Yes | Yes | Yes | Yes | Yes | Yes | No |
| 74 | Figueras, et al. (2015) | D | Yes | Yes | Yes | Yes | Yes | Yes | Yes | Yes | Yes | Yes | Yes | No |
| 75 | Gao, et al. (2019) | DV | Yes | Yes | Yes | Yes | Yes | Yes | Yes | Yes | Yes | Yes | Yes | No |
| 76 | Isakov, et al. (2019) | D | Yes | Yes | Yes | Yes | Yes | Yes | Yes | Yes | Yes | Yes | Yes | No |
| 77 | Isono, et al. (2011) | D | Yes | Yes | Yes | Yes | Yes | Yes | Yes | Yes | Yes | Yes | Yes | No |
| 78 | Kang, et al. (2019) | DV | Yes | Yes | Yes | Yes | Yes | Yes | Yes | Yes | Yes | Yes | Yes | No |
| 79 | Khan, et al. (2019) | D | Yes | Yes | Yes | Yes | Yes | Yes | Yes | Yes | Yes | Yes | Yes | No |
| 80 | Koivu, et al. (2020) | DV | Yes | Yes | Yes | Yes | Yes | Yes | Yes | Yes | Yes | Yes | Yes | No |
| 81 | Kok, et al. (2011) | D | Yes | Yes | Yes | Yes | Yes | Yes | Yes | Yes | Yes | Yes | Yes | No |
| 82 | Lee, et al. (2018) | DV | Yes | Yes | Yes | Yes | Yes | Yes | Yes | Yes | Yes | Yes | Yes | No |
| 83 | Leonarduzzi R, et al. (2015) | D | Yes | Yes | Yes | Yes | Yes | Yes | Yes | Yes | Yes | Yes | Yes | No |
| 84 | Li, et al. (2017) | D | Yes | Yes | Yes | Yes | Yes | Yes | Yes | Yes | Yes | Yes | Yes | No |
| 85 | McCowan, et al. (2017) | D | Yes | Yes | Yes | Yes | Yes | Yes | Yes | Yes | Yes | Yes | Yes | No |
| 86 | Meijerink, et al. (2016) | DV | Yes | Yes | Yes | Yes | Yes | Yes | Yes | Yes | Yes | Yes | Yes | No |
| 87 | Metz, et al. (2013) | DV | Yes | Yes | Yes | Yes | Yes | Yes | Yes | Yes | Yes | Yes | Yes | No |
| 88 | Milewski, et al. (2017) | D | Yes | Yes | Yes | Yes | Yes | Yes | Yes | Yes | Yes | Yes | Yes | No |
| 89 | Myers, et al. (2013) | D | Yes | Yes | Yes | Yes | Yes | Yes | Yes | Yes | Yes | Yes | Yes | No |
| 90 | Payne, et al. (2015) | D | Yes | Yes | Yes | Yes | Yes | Yes | Yes | Yes | Yes | Yes | Yes | No |
| 91 | Pettersson, et al. (2017) | D | Yes | Yes | Yes | Yes | Yes | Yes | Yes | Yes | Yes | Yes | Yes | No |
| 92 | Qiu, et al. (2019) | D | Yes | Yes | Yes | Yes | Yes | Yes | Yes | Yes | Yes | Yes | Yes | No |
| 93 | Reid, et al. (2015) | D | Yes | Yes | Yes | Yes | Yes | Yes | Yes | Yes | Yes | Yes | Yes | No |
| 94 | Ryu, et al. (2019) | D | Yes | Yes | Yes | Yes | Yes | Yes | Yes | Yes | Yes | Yes | Yes | No |
| 95 | Scheinhardt, et al. (2018) | V | Yes | Yes | Yes | Yes | Yes | Yes | Yes | Yes | Yes | Yes | Yes | No |
| 96 | Signorini, et al. (2020) | D | Yes | Yes | Yes | Yes | Yes | Yes | Yes | Yes | Yes | Yes | Yes | No |
| 97 | Spilka J, et al. (2015) | D | Yes | Yes | Yes | Yes | Yes | Yes | Yes | Yes | Yes | Yes | Yes | No |
| 98 | Stamatopoulos, et al. (2015) | DV | Yes | Yes | Yes | Yes | Yes | Yes | Yes | Yes | Yes | Yes | Yes | No |
| 99 | Stott, et al. (2017) | D | Yes | Yes | Yes | Yes | Yes | Yes | Yes | Yes | Yes | Yes | Yes | No |
| 100 | Timmerman, et al. (2010) | D | Yes | Yes | Yes | Yes | Yes | Yes | Yes | Yes | Yes | Yes | Yes | No |
| 101 | Tsur, et al. (2019) | DV | Yes | Yes | Yes | Yes | Yes | Yes | Yes | Yes | Yes | Yes | Yes | No |
| 102 | Uyar A, et al. (2010) | D | Yes | Yes | Yes | Yes | Yes | Yes | Yes | Yes | Yes | Yes | Yes | No |
| 103 | Van Calster, et al. (2009) | DV | Yes | Yes | Yes | Yes | Yes | Yes | Yes | Yes | Yes | Yes | Yes | No |
| 104 | van der Ham, et al. (2014) | D | Yes | Yes | Yes | Yes | Yes | Yes | Yes | Yes | Yes | Yes | Yes | No |
| 105 | Verhoeven, et al. (2016) | D | Yes | Yes | Yes | Yes | Yes | Yes | Yes | Yes | Yes | Yes | Yes | No |
| 106 | Vieira, et al. (2017) | D | Yes | Yes | Yes | Yes | Yes | Yes | Yes | Yes | Yes | Yes | Yes | No |
| 107 | Visentin, et al. (2017) | D | Yes | Yes | Yes | Yes | Yes | Yes | Yes | Yes | Yes | Yes | Yes | No |
| 108 | Wang, et al. (2013) | D | Yes | Yes | Yes | Yes | Yes | Yes | Yes | Yes | Yes | Yes | Yes | No |
| 109 | Yu, et al. (2018) | D | Yes | Yes | Yes | Yes | Yes | Yes | Yes | Yes | Yes | Yes | Yes | No |
| 110 | Zwertbroek, et al. (2017) | D | Yes | Yes | Yes | Yes | Yes | Yes | Yes | Yes | Yes | Yes | Yes | No |
| 111 | Alberola-Rubio J, et al. (2017) | D | Yes | Yes | Yes | Yes | Yes | Yes | Yes | Yes | Yes | Yes | Yes | No |
| 112 | Allouche, et al. (2011) | DV | Yes | Yes | Yes | Yes | Yes | Yes | Yes | Yes | Yes | Yes | Yes | No |
| 113 | Benalcazar-Parra, et al. (2019) | D | Yes | Yes | Yes | Yes | Yes | Yes | Yes | Yes | Yes | Yes | Yes | No |
| 114 | Berntorp, et al. (2015) | D | Yes | Yes | Yes | Yes | Yes | Yes | Yes | Yes | Yes | Yes | Yes | No |
| 115 | Broekmans, et al. (2014) | DV | Yes | Yes | Yes | Yes | Yes | Yes | Yes | Yes | Yes | Yes | Yes | No |
| 116 | Casikar, et al. (2013) | DV | Yes | Yes | Yes | Yes | Yes | Yes | Yes | Yes | Yes | Yes | Yes | No |
| 117 | Cerqueira FR, et al. (2014) | D | Yes | Yes | Yes | Yes | Yes | Yes | Yes | Yes | Yes | Yes | Yes | No |
| 118 | Elaveyini, et al. (2011) | D | Yes | Yes | Yes | Yes | Yes | Yes | Yes | Yes | Yes | Yes | Yes | No |
| 119 | Georgoulas, et al. (2017) | D | Yes | Yes | Yes | Yes | Yes | Yes | Yes | Yes | Yes | Yes | Yes | No |
| 120 | Harper, et al. (2016) | D | Yes | Yes | Yes | Yes | Yes | Yes | Yes | Yes | Yes | Yes | Yes | No |
| 121 | Hernandez-Gonzalez J, et al. (2018) | D | Yes | Yes | Yes | Yes | Yes | Yes | Yes | Yes | Yes | Yes | Yes | No |
| 122 | Jhee, et al. (2019) | D | Yes | Yes | Yes | Yes | Yes | Yes | Yes | Yes | Yes | Yes | Yes | No |
| 123 | Kawakita, et al. (2019) | D | Yes | Yes | Yes | Yes | Yes | Yes | Yes | Yes | Yes | Yes | Yes | No |
| 124 | Kuhle S, et al. (2018) | D | Yes | Yes | Yes | Yes | Yes | Yes | Yes | Yes | Yes | Yes | Yes | No |
| 125 | Kumar, et al. (2020) | DV | Yes | Yes | Yes | Yes | Yes | Yes | Yes | Yes | Yes | Yes | Yes | No |
| 126 | Lafalla, et al. (2019) | D | Yes | Yes | Yes | Yes | Yes | Yes | Yes | Yes | Yes | Yes | Yes | No |
| 127 | Liu, et al. (2019) | D | Yes | Yes | Yes | Yes | Yes | Yes | Yes | Yes | Yes | Yes | Yes | No |
| 128 | McCowan, et al. (2013) | D | Yes | Yes | Yes | Yes | Yes | Yes | Yes | Yes | Yes | Yes | Yes | No |
| 129 | Meister, et al. (2016) | D | Yes | Yes | Yes | Yes | Yes | Yes | Yes | Yes | Yes | Yes | Yes | No |
| 130 | Mello, et al. (2001) | D | Yes | Yes | Yes | Yes | Yes | Yes | Yes | Yes | Yes | Yes | Yes | No |
| 131 | Oates, et al. (2013) | D | Yes | Yes | Yes | Yes | Yes | Yes | Yes | Yes | Yes | Yes | Yes | No |
| 132 | Petrozziello, et al. (2018) | D | Yes | Yes | Yes | Yes | Yes | Yes | Yes | Yes | Yes | Yes | Yes | No |
| 133 | Petrozziello, et al. (2019) | D | Yes | Yes | Yes | Yes | Yes | Yes | Yes | Yes | Yes | Yes | Yes | No |
| 134 | Pettersson, et al. (2010) | D | Yes | Yes | Yes | Yes | Yes | Yes | Yes | Yes | Yes | Yes | Yes | No |
| 135 | Ramanah, et al. (2018) | D | Yes | Yes | Yes | Yes | Yes | Yes | Yes | Yes | Yes | Yes | Yes | No |
| 136 | Spilka J, et al. (2017) | D | Yes | Yes | Yes | Yes | Yes | Yes | Yes | Yes | Yes | Yes | Yes | No |
| 137 | Stroux, et al. (2017) | D | Yes | Yes | Yes | Yes | Yes | Yes | Yes | Yes | Yes | Yes | Yes | No |
| 138 | Valensise, et al. (2006) | D | Yes | Yes | Yes | Yes | Yes | Yes | Yes | Yes | Yes | Yes | Yes | No |
| 139 | Vogiatzi, et al. (2019) | D | Yes | Yes | Yes | Yes | Yes | Yes | Yes | Yes | Yes | Yes | Yes | No |
| 140 | Xu L, et al. (2013) | D | Yes | Yes | Yes | Yes | Yes | Yes | Yes | Yes | Yes | Yes | Yes | No |
| 141 | Xu, et al. (2020) | D | Yes | Yes | Yes | Yes | Yes | Yes | Yes | Yes | Yes | Yes | Yes | No |
| 142 | Zhao, et al. (2019) | DV | Yes | Yes | Yes | Yes | Yes | Yes | Yes | Yes | Yes | Yes | Yes | No |
| 143 | Abbas R, et al. (2018) | D | Yes | Yes | Yes | Yes | Yes | Yes | Yes | Yes | Yes | Yes | No |  |
| 144 | Abbas, et al. (2019) | D | Yes | Yes | Yes | Yes | Yes | Yes | Yes | Yes | Yes | Yes | No |  |
| 145 | Acharya, et al. (2017) | D | Yes | Yes | Yes | Yes | Yes | Yes | Yes | Yes | Yes | Yes | No |  |
| 146 | Ahmadzia, et al. (2018) | D | Yes | Yes | Yes | Yes | Yes | Yes | Yes | Yes | Yes | Yes | No |  |
| 147 | Akbarian, et al. (2015) | D | Yes | Yes | Yes | Yes | Yes | Yes | Yes | Yes | Yes | Yes | No |  |
| 148 | Akbulut A, et al. (2018) | D | Yes | Yes | Yes | Yes | Yes | Yes | Yes | Yes | Yes | Yes | No |  |
| 149 | Alavifard, et al. (2019) | D | Yes | Yes | Yes | Yes | Yes | Yes | Yes | Yes | Yes | Yes | No |  |
| 150 | Alexander, et al. (2018) | DV | Yes | Yes | Yes | Yes | Yes | Yes | Yes | Yes | Yes | Yes | No |  |
| 151 | Ambalavanan, et al. (2001) | D | Yes | Yes | Yes | Yes | Yes | Yes | Yes | Yes | Yes | Yes | No |  |
| 152 | Arav-Boger, et al. (2008) | D | Yes | Yes | Yes | Yes | Yes | Yes | Yes | Yes | Yes | Yes | No |  |
| 153 | Attallah, et al. (2019) | D | Yes | Yes | Yes | Yes | Yes | Yes | Yes | Yes | Yes | Yes | No |  |
| 154 | Attallah, et al. (2020) | D | Yes | Yes | Yes | Yes | Yes | Yes | Yes | Yes | Yes | Yes | No |  |
| 155 | Aung, et al. (2019) | D | Yes | Yes | Yes | Yes | Yes | Yes | Yes | Yes | Yes | Yes | No |  |
| 156 | Austdal, et al. (2015) | D | Yes | Yes | Yes | Yes | Yes | Yes | Yes | Yes | Yes | Yes | No |  |
| 157 | Bahado-Singh, et al. (2019) | D | Yes | Yes | Yes | Yes | Yes | Yes | Yes | Yes | Yes | Yes | No |  |
| 158 | Bahado-Singh, et al. (2020) | D | Yes | Yes | Yes | Yes | Yes | Yes | Yes | Yes | Yes | Yes | No |  |
| 159 | Baykal, et al. (1994) | D | Yes | Yes | Yes | Yes | Yes | Yes | Yes | Yes | Yes | Yes | No |  |
| 160 | Betts KS, et al. (2019) | DV | Yes | Yes | Yes | Yes | Yes | Yes | Yes | Yes | Yes | Yes | No |  |
| 161 | Binenbaum, et al. (2011) | D | Yes | Yes | Yes | Yes | Yes | Yes | Yes | Yes | Yes | Yes | No |  |
| 162 | Bottomley, et al. (2013) | D | Yes | Yes | Yes | Yes | Yes | Yes | Yes | Yes | Yes | Yes | No |  |
| 163 | Burai, et al. (2018) | D | Yes | Yes | Yes | Yes | Yes | Yes | Yes | Yes | Yes | Yes | No |  |
| 164 | Cairo, et al. (2018) | DV | Yes | Yes | Yes | Yes | Yes | Yes | Yes | Yes | Yes | Yes | No |  |
| 165 | Catic, et al. (2018) | D | Yes | Yes | Yes | Yes | Yes | Yes | Yes | Yes | Yes | Yes | No |  |
| 166 | Catley, et al. (2006) | DV | Yes | Yes | Yes | Yes | Yes | Yes | Yes | Yes | Yes | Yes | No |  |
| 167 | Chen, et al. (2015) | DV | Yes | Yes | Yes | Yes | Yes | Yes | Yes | Yes | Yes | Yes | No |  |
| 168 | Comert Z, et al. (2016) | DV | Yes | Yes | Yes | Yes | Yes | Yes | Yes | Yes | Yes | Yes | No |  |
| 169 | Czabanski, et al. (2010) | D | Yes | Yes | Yes | Yes | Yes | Yes | Yes | Yes | Yes | Yes | No |  |
| 170 | De Ramón Fernández, et al. (2019) | D | Yes | Yes | Yes | Yes | Yes | Yes | Yes | Yes | Yes | Yes | No |  |
| 171 | Devjak, et al. (2016) | D | Yes | Yes | Yes | Yes | Yes | Yes | Yes | Yes | Yes | Yes | No |  |
| 172 | Dhillon, et al. (2016) | DV | Yes | Yes | Yes | Yes | Yes | Yes | Yes | Yes | Yes | Yes | No |  |
| 173 | Dida, et al. (2014) | D | Yes | Yes | Yes | Yes | Yes | Yes | Yes | Yes | Yes | Yes | No |  |
| 174 | Dirvanauskas, et al. (2019) | D | Yes | Yes | Yes | Yes | Yes | Yes | Yes | Yes | Yes | Yes | No |  |
| 175 | Dithy, et al. (2019) | D | Yes | Yes | Yes | Yes | Yes | Yes | Yes | Yes | Yes | Yes | No |  |
| 176 | Du, et al. (2020) | D | Yes | Yes | Yes | Yes | Yes | Yes | Yes | Yes | Yes | Yes | No |  |
| 177 | Dukhovny, et al. (2012) | D | Yes | Yes | Yes | Yes | Yes | Yes | Yes | Yes | Yes | Yes | No |  |
| 178 | Esty A, et al. (2018) | D | Yes | Yes | Yes | Yes | Yes | Yes | Yes | Yes | Yes | Yes | No |  |
| 179 | Feng, et al. (2018) | D | Yes | Yes | Yes | Yes | Yes | Yes | Yes | Yes | Yes | Yes | No |  |
| 180 | Fries, et al. (2019) | D | Yes | Yes | Yes | Yes | Yes | Yes | Yes | Yes | Yes | Yes | No |  |
| 181 | Frigerio, et al. (2018) | D | Yes | Yes | Yes | Yes | Yes | Yes | Yes | Yes | Yes | Yes | No |  |
| 182 | Galderisi, et al. (2019) | D | Yes | Yes | Yes | Yes | Yes | Yes | Yes | Yes | Yes | Yes | No |  |
| 183 | Georgoulas, et al. (2017) | D | Yes | Yes | Yes | Yes | Yes | Yes | Yes | Yes | Yes | Yes | No |  |
| 184 | Gioacchini, et al. (2018) | D | Yes | Yes | Yes | Yes | Yes | Yes | Yes | Yes | Yes | Yes | No |  |
| 185 | Goodson, et al. (2017) | D | Yes | Yes | Yes | Yes | Yes | Yes | Yes | Yes | Yes | Yes | No |  |
| 186 | Hamilton, et al. (2020) | D | Yes | Yes | Yes | Yes | Yes | Yes | Yes | Yes | Yes | Yes | No |  |
| 187 | Hassan MR, et al. (2018) | D | Yes | Yes | Yes | Yes | Yes | Yes | Yes | Yes | Yes | Yes | No |  |
| 188 | He, et al. (2019) | DV | Yes | Yes | Yes | Yes | Yes | Yes | Yes | Yes | Yes | Yes | No |  |
| 189 | Heidari, et al. (2018) | D | Yes | Yes | Yes | Yes | Yes | Yes | Yes | Yes | Yes | Yes | No |  |
| 190 | Hu, et al. (2019) | D | Yes | Yes | Yes | Yes | Yes | Yes | Yes | Yes | Yes | Yes | No |  |
| 191 | Inbarani, et al. (2014) | D | Yes | Yes | Yes | Yes | Yes | Yes | Yes | Yes | Yes | Yes | No |  |
| 192 | Iraji, et al. (2019) | D | Yes | Yes | Yes | Yes | Yes | Yes | Yes | Yes | Yes | Yes | No |  |
| 193 | Jadhav S, et al. (2011) | DV | Yes | Yes | Yes | Yes | Yes | Yes | Yes | Yes | Yes | Yes | No |  |
| 194 | Jalali, et al. (2013) | D | Yes | Yes | Yes | Yes | Yes | Yes | Yes | Yes | Yes | Yes | No |  |
| 195 | Jang, et al. (2018) | D | Yes | Yes | Yes | Yes | Yes | Yes | Yes | Yes | Yes | Yes | No |  |
| 196 | Kalafat, et al. (2019) | D | Yes | Yes | Yes | Yes | Yes | Yes | Yes | Yes | Yes | Yes | No |  |
| 197 | Kanakasabapathy, et al. (2019) | D | Yes | Yes | Yes | Yes | Yes | Yes | Yes | Yes | Yes | Yes | No |  |
| 198 | Kang, et al. (2020) | DV | Yes | Yes | Yes | Yes | Yes | Yes | Yes | Yes | Yes | Yes | No |  |
| 199 | Kaur, et al. (2019) | D | Yes | Yes | Yes | Yes | Yes | Yes | Yes | Yes | Yes | Yes | No |  |
| 200 | Kayode, et al. (2016) | D | Yes | Yes | Yes | Yes | Yes | Yes | Yes | Yes | Yes | Yes | No |  |
| 201 | Khatibi, et al. (2019) | D | Yes | Yes | Yes | Yes | Yes | Yes | Yes | Yes | Yes | Yes | No |  |
| 202 | Khosravi, et al. (2019) | DV | Yes | Yes | Yes | Yes | Yes | Yes | Yes | Yes | Yes | Yes | No |  |
| 203 | Kim, et al. (2018) | D | Yes | Yes | Yes | Yes | Yes | Yes | Yes | Yes | Yes | Yes | No |  |
| 204 | Koivu A, et al. (2018) | D | Yes | Yes | Yes | Yes | Yes | Yes | Yes | Yes | Yes | Yes | No |  |
| 205 | Krupa, et al. (2011) | D | Yes | Yes | Yes | Yes | Yes | Yes | Yes | Yes | Yes | Yes | No |  |
| 206 | Lafuente-Ganuza, et al. (2020) | DV | Yes | Yes | Yes | Yes | Yes | Yes | Yes | Yes | Yes | Yes | No |  |
| 207 | Li, et al. (2019) | D | Yes | Yes | Yes | Yes | Yes | Yes | Yes | Yes | Yes | Yes | No |  |
| 208 | Li, et al. (2019) | D | Yes | Yes | Yes | Yes | Yes | Yes | Yes | Yes | Yes | Yes | No |  |
| 209 | Liu, et al. (2019) | D | Yes | Yes | Yes | Yes | Yes | Yes | Yes | Yes | Yes | Yes | No |  |
| 210 | Luo Y, et al. (2017) | DV | Yes | Yes | Yes | Yes | Yes | Yes | Yes | Yes | Yes | Yes | No |  |
| 211 | MacDowell, et al. (2001) | D | Yes | Yes | Yes | Yes | Yes | Yes | Yes | Yes | Yes | Yes | No |  |
| 212 | Macones, et al. (2013) | D | Yes | Yes | Yes | Yes | Yes | Yes | Yes | Yes | Yes | Yes | No |  |
| 213 | Magenes, et al. (2004) | D | Yes | Yes | Yes | Yes | Yes | Yes | Yes | Yes | Yes | Yes | No |  |
| 214 | Malacova, et al. (2020) | D | Yes | Yes | Yes | Yes | Yes | Yes | Yes | Yes | Yes | Yes | No |  |
| 215 | Milewski, et al. (2015) | DV | Yes | Yes | Yes | Yes | Yes | Yes | Yes | Yes | Yes | Yes | No |  |
| 216 | Moreira, et al. (2019) | D | Yes | Yes | Yes | Yes | Yes | Yes | Yes | Yes | Yes | Yes | No |  |
| 217 | Natarajan S, et al. (2017) | D | Yes | Yes | Yes | Yes | Yes | Yes | Yes | Yes | Yes | Yes | No |  |
| 218 | Ng, et al. (2019) | D | Yes | Yes | Yes | Yes | Yes | Yes | Yes | Yes | Yes | Yes | No |  |
| 219 | Ocak, et al. (2013) | D | Yes | Yes | Yes | Yes | Yes | Yes | Yes | Yes | Yes | Yes | No |  |
| 220 | Ochab, et al. (2016) | D | Yes | Yes | Yes | Yes | Yes | Yes | Yes | Yes | Yes | Yes | No |  |
| 221 | Pan I, et al. (2017) | D | Yes | Yes | Yes | Yes | Yes | Yes | Yes | Yes | Yes | Yes | No |  |
| 222 | Paternina-Caicedo, et al. (2017) | V | Yes | Yes | Yes | Yes | Yes | Yes | Yes | Yes | Yes | Yes | No |  |
| 223 | Payne, et al. (2018) | D | Yes | Yes | Yes | Yes | Yes | Yes | Yes | Yes | Yes | Yes | No |  |
| 224 | Rawashdeh, et al. (2020) | D | Yes | Yes | Yes | Yes | Yes | Yes | Yes | Yes | Yes | Yes | No |  |
| 225 | Ricard, et al. (2017) | DV | Yes | Yes | Yes | Yes | Yes | Yes | Yes | Yes | Yes | Yes | No |  |
| 226 | Rossi, et al. (2019) | DV | Yes | Yes | Yes | Yes | Yes | Yes | Yes | Yes | Yes | Yes | No |  |
| 227 | Sahin H, et al. (2015) | D | Yes | Yes | Yes | Yes | Yes | Yes | Yes | Yes | Yes | Yes | No |  |
| 228 | Sahli, et al. (2019) | D | Yes | Yes | Yes | Yes | Yes | Yes | Yes | Yes | Yes | Yes | No |  |
| 229 | Samanta, et al. (2009) | D | Yes | Yes | Yes | Yes | Yes | Yes | Yes | Yes | Yes | Yes | No |  |
| 230 | Samanta, et al. (2009) | D | Yes | Yes | Yes | Yes | Yes | Yes | Yes | Yes | Yes | Yes | No |  |
| 231 | Shah SAA, et al. (2015) | D | Yes | Yes | Yes | Yes | Yes | Yes | Yes | Yes | Yes | Yes | No |  |
| 232 | Shigemi, et al. (2019) | D | Yes | Yes | Yes | Yes | Yes | Yes | Yes | Yes | Yes | Yes | No |  |
| 233 | Sievert, et al. (2017) | D | Yes | Yes | Yes | Yes | Yes | Yes | Yes | Yes | Yes | Yes | No |  |
| 234 | Simpson, et al. (2020) | D | Yes | Yes | Yes | Yes | Yes | Yes | Yes | Yes | Yes | Yes | No |  |
| 235 | Siriwardhana, et al. (2017) | D | Yes | Yes | Yes | Yes | Yes | Yes | Yes | Yes | Yes | Yes | No |  |
| 236 | Srivastava Y, et al. (2019) | D | Yes | Yes | Yes | Yes | Yes | Yes | Yes | Yes | Yes | Yes | No |  |
| 237 | Street, et al. (2008) | D | Yes | Yes | Yes | Yes | Yes | Yes | Yes | Yes | Yes | Yes | No |  |
| 238 | Sullivan, et al. (2018) | D | Yes | Yes | Yes | Yes | Yes | Yes | Yes | Yes | Yes | Yes | No |  |
| 239 | Sultan, et al. (2016) | DV | Yes | Yes | Yes | Yes | Yes | Yes | Yes | Yes | Yes | Yes | No |  |
| 240 | Sun, et al. (2019) | D | Yes | Yes | Yes | Yes | Yes | Yes | Yes | Yes | Yes | Yes | No |  |
| 241 | Tabrizi, et al. (2017) | D | Yes | Yes | Yes | Yes | Yes | Yes | Yes | Yes | Yes | Yes | No |  |
| 242 | Tang H, et al. (2018) | DV | Yes | Yes | Yes | Yes | Yes | Yes | Yes | Yes | Yes | Yes | No |  |
| 243 | Tang, et al. (2009) | D | Yes | Yes | Yes | Yes | Yes | Yes | Yes | Yes | Yes | Yes | No |  |
| 244 | Tejera, et al. (2011) | DV | Yes | Yes | Yes | Yes | Yes | Yes | Yes | Yes | Yes | Yes | No |  |
| 245 | Vickram, et al. (2016) | V | Yes | Yes | Yes | Yes | Yes | Yes | Yes | Yes | Yes | Yes | No |  |
| 246 | Vijayalakshmi, et al. (2020) | D | Yes | Yes | Yes | Yes | Yes | Yes | Yes | Yes | Yes | Yes | No |  |
| 247 | Wang, et al. (2016) | D | Yes | Yes | Yes | Yes | Yes | Yes | Yes | Yes | Yes | Yes | No |  |
| 248 | Westerhuis, et al. (2012) | D | Yes | Yes | Yes | Yes | Yes | Yes | Yes | Yes | Yes | Yes | No |  |
| 249 | Wilson, et al. (2016) | DV | Yes | Yes | Yes | Yes | Yes | Yes | Yes | Yes | Yes | Yes | No |  |
| 250 | Xie, et al. (2020) | D | Yes | Yes | Yes | Yes | Yes | Yes | Yes | Yes | Yes | Yes | No |  |
| 251 | Yang J, et al. (2018) | DV | Yes | Yes | Yes | Yes | Yes | Yes | Yes | Yes | Yes | Yes | No |  |
| 252 | Zernikow, et al. (1998) | D | Yes | Yes | Yes | Yes | Yes | Yes | Yes | Yes | Yes | Yes | No |  |
| 253 | Zhang, et al. (2018) | D | Yes | Yes | Yes | Yes | Yes | Yes | Yes | Yes | Yes | Yes | No |  |
| 254 | Zhang, et al. (2019) | D | Yes | Yes | Yes | Yes | Yes | Yes | Yes | Yes | Yes | Yes | No |  |
| 255 | Zhang, et al. (2020) | D | Yes | Yes | Yes | Yes | Yes | Yes | Yes | Yes | Yes | Yes | No |  |
| 256 | Zhu, et al. (2019) | D | Yes | Yes | Yes | Yes | Yes | Yes | Yes | Yes | Yes | Yes | No |  |
| 257 | Borowska, et al. (2018) | D | Yes | Yes | Yes | Yes | Yes | Yes | Yes | Yes | Yes | No |  |  |
| 258 | Cannas, et al. (2019) | D | Yes | Yes | Yes | Yes | Yes | Yes | Yes | Yes | Yes | No |  |  |
| 259 | Dash, et al. (2014) | D | Yes | Yes | Yes | Yes | Yes | Yes | Yes | Yes | Yes | No |  |  |
| 260 | Fang, et al. (2011) | D | Yes | Yes | Yes | Yes | Yes | Yes | Yes | Yes | Yes | No |  |  |
| 261 | Frick, et al. (2020) | D | Yes | Yes | Yes | Yes | Yes | Yes | Yes | Yes | Yes | No |  |  |
| 262 | Hutton, et al. (2017) | D | Yes | Yes | Yes | Yes | Yes | Yes | Yes | Yes | Yes | No |  |  |
| 263 | Karvelis, et al. (2015) | D | Yes | Yes | Yes | Yes | Yes | Yes | Yes | Yes | Yes | No |  |  |
| 264 | Kragh, et al. (2019) | D | Yes | Yes | Yes | Yes | Yes | Yes | Yes | Yes | Yes | No |  |  |
| 265 | La Rosa, et al. (2008) | D | Yes | Yes | Yes | Yes | Yes | Yes | Yes | Yes | Yes | No |  |  |
| 266 | Moslem, et al. (2011) | D | Yes | Yes | Yes | Yes | Yes | Yes | Yes | Yes | Yes | No |  |  |
| 267 | Soslow, et al. (2013) | D | Yes | Yes | Yes | Yes | Yes | Yes | Yes | Yes | Yes | No |  |  |
| 268 | Teder, et al. (2019) | D | Yes | Yes | Yes | Yes | Yes | Yes | Yes | Yes | Yes | No |  |  |
| 269 | Warrick, et al. (2010) | D | Yes | Yes | Yes | Yes | Yes | Yes | Yes | Yes | Yes | No |  |  |
| 270 | Zhang, et al. (2020) | D | Yes | Yes | Yes | Yes | Yes | Yes | Yes | Yes | Yes | No |  |  |
| 271 | Chamidah N, et al. (2015) | D | Yes | Yes | Yes | Yes | Yes | Yes | Yes | Yes | Yes | No |  |  |
| 272 | Goodale BM, et al. (2019) | D | Yes | Yes | Yes | Yes | Yes | Yes | Yes | Yes | Yes | No |  |  |
| 273 | Gorthi A, et al. (2009) | D | Yes | Yes | Yes | Yes | Yes | Yes | Yes | Yes | Yes | No |  |  |
| 274 | Ocak H. A, et al. (2013) | D | Yes | Yes | Yes | Yes | Yes | Yes | Yes | Yes | Yes | No |  |  |
| 275 | Ravindran S, et al. (2015) | D | Yes | Yes | Yes | Yes | Yes | Yes | Yes | Yes | Yes | No |  |  |
| 276 | Abuelghar, et al. (2019) | D | Yes | Yes | Yes | Yes | Yes | Yes | Yes | Yes | No |  |  |  |
| 277 | Allen, et al. (2018) | D | Yes | Yes | Yes | Yes | Yes | Yes | Yes | Yes | No |  |  |  |
| 278 | Bakalis, et al. (2015) | D | Yes | Yes | Yes | Yes | Yes | Yes | Yes | Yes | No |  |  |  |
| 279 | Bakalis, et al. (2015) | D | Yes | Yes | Yes | Yes | Yes | Yes | Yes | Yes | No |  |  |  |
| 280 | Bakalis, et al. (2015) | D | Yes | Yes | Yes | Yes | Yes | Yes | Yes | Yes | No |  |  |  |
| 281 | Bakalis, et al. (2015) | D | Yes | Yes | Yes | Yes | Yes | Yes | Yes | Yes | No |  |  |  |
| 282 | Belfort, et al. (2012) | D | Yes | Yes | Yes | Yes | Yes | Yes | Yes | Yes | No |  |  |  |
| 283 | Bertozzi, et al. (2011) | D | Yes | Yes | Yes | Yes | Yes | Yes | Yes | Yes | No |  |  |  |
| 284 | Bourdages, et al. (2018) | D | Yes | Yes | Yes | Yes | Yes | Yes | Yes | Yes | No |  |  |  |
| 285 | Burgos-Artizzu, et al. (2019) | D | Yes | Yes | Yes | Yes | Yes | Yes | Yes | Yes | No |  |  |  |
| 286 | Calí, et al. (2020) | D | Yes | Yes | Yes | Yes | Yes | Yes | Yes | Yes | No |  |  |  |
| 287 | Carvalho Neto, et al. (2019) | D | Yes | Yes | Yes | Yes | Yes | Yes | Yes | Yes | No |  |  |  |
| 288 | Cavallaro, et al. (2018) | D | Yes | Yes | Yes | Yes | Yes | Yes | Yes | Yes | No |  |  |  |
| 289 | Cetinkaya, et al. (2013) | D | Yes | Yes | Yes | Yes | Yes | Yes | Yes | Yes | No |  |  |  |
| 290 | Chen, et al. (2017) | D | Yes | Yes | Yes | Yes | Yes | Yes | Yes | Yes | No |  |  |  |
| 291 | Chetty, et al. (2011) | D | Yes | Yes | Yes | Yes | Yes | Yes | Yes | Yes | No |  |  |  |
| 292 | Chu, et al. (2019) | D | Yes | Yes | Yes | Yes | Yes | Yes | Yes | Yes | No |  |  |  |
| 293 | Ciobanou, et al. (2019) | D | Yes | Yes | Yes | Yes | Yes | Yes | Yes | Yes | No |  |  |  |
| 294 | Ciobanu, et al. (2019) | D | Yes | Yes | Yes | Yes | Yes | Yes | Yes | Yes | No |  |  |  |
| 295 | Ciobanu, et al. (2019) | D | Yes | Yes | Yes | Yes | Yes | Yes | Yes | Yes | No |  |  |  |
| 296 | Crovetto, et al. (2014) | D | Yes | Yes | Yes | Yes | Yes | Yes | Yes | Yes | No |  |  |  |
| 297 | Cruz-Martinez, et al. (2010) | D | Yes | Yes | Yes | Yes | Yes | Yes | Yes | Yes | No |  |  |  |
| 298 | Damaso, et al. (2019) | D | Yes | Yes | Yes | Yes | Yes | Yes | Yes | Yes | No |  |  |  |
| 299 | D'Antonio, et al. (2018) | D | Yes | Yes | Yes | Yes | Yes | Yes | Yes | Yes | No |  |  |  |
| 300 | Delić, et al. (2014) | D | Yes | Yes | Yes | Yes | Yes | Yes | Yes | Yes | No |  |  |  |
| 301 | Di Lorenzo, et al. (2012) | D | Yes | Yes | Yes | Yes | Yes | Yes | Yes | Yes | No |  |  |  |
| 302 | Di Martino, et al. (2016) | D | Yes | Yes | Yes | Yes | Yes | Yes | Yes | Yes | No |  |  |  |
| 303 | Direkvand-Moghadam, et al. (2012) | D | Yes | Yes | Yes | Yes | Yes | Yes | Yes | Yes | No |  |  |  |
| 304 | Doulaveris, et al. (2018) | D | Yes | Yes | Yes | Yes | Yes | Yes | Yes | Yes | No |  |  |  |
| 305 | Ducarme, et al. (2019) | D | Yes | Yes | Yes | Yes | Yes | Yes | Yes | Yes | No |  |  |  |
| 306 | Eggebø, et al. (2014) | D | Yes | Yes | Yes | Yes | Yes | Yes | Yes | Yes | No |  |  |  |
| 307 | El-Achi, et al. (2020) | D | Yes | Yes | Yes | Yes | Yes | Yes | Yes | Yes | No |  |  |  |
| 308 | Ellaithy, et al. (2018) | D | Yes | Yes | Yes | Yes | Yes | Yes | Yes | Yes | No |  |  |  |
| 309 | Fadigas, et al. (2015) | D | Yes | Yes | Yes | Yes | Yes | Yes | Yes | Yes | No |  |  |  |
| 310 | Fadigas, et al. (2015) | D | Yes | Yes | Yes | Yes | Yes | Yes | Yes | Yes | No |  |  |  |
| 311 | Fadigas, et al. (2015) | D | Yes | Yes | Yes | Yes | Yes | Yes | Yes | Yes | No |  |  |  |
| 312 | Familiari, et al. (2016) | D | Yes | Yes | Yes | Yes | Yes | Yes | Yes | Yes | No |  |  |  |
| 313 | Familiari, et al. (2016) | D | Yes | Yes | Yes | Yes | Yes | Yes | Yes | Yes | No |  |  |  |
| 314 | Farina, et al. (2011) | D | Yes | Yes | Yes | Yes | Yes | Yes | Yes | Yes | No |  |  |  |
| 315 | Fiolna, et al. (2019) | D | Yes | Yes | Yes | Yes | Yes | Yes | Yes | Yes | No |  |  |  |
| 316 | Fiolna, et al. (2019) | D | Yes | Yes | Yes | Yes | Yes | Yes | Yes | Yes | No |  |  |  |
| 317 | Fishel, et al. (2018) | D | Yes | Yes | Yes | Yes | Yes | Yes | Yes | Yes | No |  |  |  |
| 318 | Fontanella, et al. (2019) | D | Yes | Yes | Yes | Yes | Yes | Yes | Yes | Yes | No |  |  |  |
| 319 | Gasse, et al. (2018) | D | Yes | Yes | Yes | Yes | Yes | Yes | Yes | Yes | No |  |  |  |
| 320 | Giguère, et al. (2015) | D | Yes | Yes | Yes | Yes | Yes | Yes | Yes | Yes | No |  |  |  |
| 321 | Gómez-Arriaga, et al. (2014) | D | Yes | Yes | Yes | Yes | Yes | Yes | Yes | Yes | No |  |  |  |
| 322 | Grynnerup, et al. (2019) | D | Yes | Yes | Yes | Yes | Yes | Yes | Yes | Yes | No |  |  |  |
| 323 | Gurgel Alves, et al. (2014) | D | Yes | Yes | Yes | Yes | Yes | Yes | Yes | Yes | No |  |  |  |
| 324 | Guzman, et al. (2013) | D | Yes | Yes | Yes | Yes | Yes | Yes | Yes | Yes | No |  |  |  |
| 325 | Hamilton, et al. (1994) | D | Yes | Yes | Yes | Yes | Yes | Yes | Yes | Yes | No |  |  |  |
| 326 | Hao, et al. (2017) | D | Yes | Yes | Yes | Yes | Yes | Yes | Yes | Yes | No |  |  |  |
| 327 | Hassan, et al. (2017) | D | Yes | Yes | Yes | Yes | Yes | Yes | Yes | Yes | No |  |  |  |
| 328 | Hernández-Martínez, et al. (2016) | D | Yes | Yes | Yes | Yes | Yes | Yes | Yes | Yes | No |  |  |  |
| 329 | Hiersch, et al. (2017) | D | Yes | Yes | Yes | Yes | Yes | Yes | Yes | Yes | No |  |  |  |
| 330 | Hilal, et al. (2017) | D | Yes | Yes | Yes | Yes | Yes | Yes | Yes | Yes | No |  |  |  |
| 331 | Holst, et al. (2011) | D | Yes | Yes | Yes | Yes | Yes | Yes | Yes | Yes | No |  |  |  |
| 332 | Huang, et al. (2018) | D | Yes | Yes | Yes | Yes | Yes | Yes | Yes | Yes | No |  |  |  |
| 333 | Huang, et al. (2019) | D | Yes | Yes | Yes | Yes | Yes | Yes | Yes | Yes | No |  |  |  |
| 334 | Iwatani, et al. (2013) | D | Yes | Yes | Yes | Yes | Yes | Yes | Yes | Yes | No |  |  |  |
| 335 | Izci-Balserak, et al. (2019) | D | Yes | Yes | Yes | Yes | Yes | Yes | Yes | Yes | No |  |  |  |
| 336 | Kaur, et al. (2012) | D | Yes | Yes | Yes | Yes | Yes | Yes | Yes | Yes | No |  |  |  |
| 337 | Kienast, et al. (2016) | D | Yes | Yes | Yes | Yes | Yes | Yes | Yes | Yes | No |  |  |  |
| 338 | Kim, et al. (2018) | D | Yes | Yes | Yes | Yes | Yes | Yes | Yes | Yes | No |  |  |  |
| 339 | Kowalski, et al. (2017) | D | Yes | Yes | Yes | Yes | Yes | Yes | Yes | Yes | No |  |  |  |
| 340 | Kurakazu, et al. (2019) | D | Yes | Yes | Yes | Yes | Yes | Yes | Yes | Yes | No |  |  |  |
| 341 | Lédée, et al. (2013) | D | Yes | Yes | Yes | Yes | Yes | Yes | Yes | Yes | No |  |  |  |
| 342 | Lesmes, et al. (2015) | D | Yes | Yes | Yes | Yes | Yes | Yes | Yes | Yes | No |  |  |  |
| 343 | Lesmes, et al. (2015) | D | Yes | Yes | Yes | Yes | Yes | Yes | Yes | Yes | No |  |  |  |
| 344 | Lesmes, et al. (2015) | D | Yes | Yes | Yes | Yes | Yes | Yes | Yes | Yes | No |  |  |  |
| 345 | Li, et al. (2016) | D | Yes | Yes | Yes | Yes | Yes | Yes | Yes | Yes | No |  |  |  |
| 346 | Li, et al. (2018) | D | Yes | Yes | Yes | Yes | Yes | Yes | Yes | Yes | No |  |  |  |
| 347 | Li, et al. (2018) | D | Yes | Yes | Yes | Yes | Yes | Yes | Yes | Yes | No |  |  |  |
| 348 | Liu, et al. (2015) | D | Yes | Yes | Yes | Yes | Yes | Yes | Yes | Yes | No |  |  |  |
| 349 | Llaneza-Suarez, et al. (2014) | D | Yes | Yes | Yes | Yes | Yes | Yes | Yes | Yes | No |  |  |  |
| 350 | Lu, et al. (2018) | D | Yes | Yes | Yes | Yes | Yes | Yes | Yes | Yes | No |  |  |  |
| 351 | Lukaszuk, et al. (2013) | D | Yes | Yes | Yes | Yes | Yes | Yes | Yes | Yes | No |  |  |  |
| 352 | Manley, et al. (2016) | D | Yes | Yes | Yes | Yes | Yes | Yes | Yes | Yes | No |  |  |  |
| 353 | Mastrodima, et al. (2016) | D | Yes | Yes | Yes | Yes | Yes | Yes | Yes | Yes | No |  |  |  |
| 354 | McKeating, et al. (2020) | D | Yes | Yes | Yes | Yes | Yes | Yes | Yes | Yes | No |  |  |  |
| 355 | Miranda, et al. (2017) | D | Yes | Yes | Yes | Yes | Yes | Yes | Yes | Yes | No |  |  |  |
| 356 | Mizrachi, et al. (2018) | D | Yes | Yes | Yes | Yes | Yes | Yes | Yes | Yes | No |  |  |  |
| 357 | Moore Simas, et al. (2014) | D | Yes | Yes | Yes | Yes | Yes | Yes | Yes | Yes | No |  |  |  |
| 358 | Morales-Roselló, et al. (2019) | D | Yes | Yes | Yes | Yes | Yes | Yes | Yes | Yes | No |  |  |  |
| 359 | Morales-Roselló, et al. (2019) | D | Yes | Yes | Yes | Yes | Yes | Yes | Yes | Yes | No |  |  |  |
| 360 | Moro, et al. (2016) | D | Yes | Yes | Yes | Yes | Yes | Yes | Yes | Yes | No |  |  |  |
| 361 | Murali, et al. (2014) | D | Yes | Yes | Yes | Yes | Yes | Yes | Yes | Yes | No |  |  |  |
| 362 | Oh, et al. (2012) | D | Yes | Yes | Yes | Yes | Yes | Yes | Yes | Yes | No |  |  |  |
| 363 | Ohkuchi, et al. (2012) | D | Yes | Yes | Yes | Yes | Yes | Yes | Yes | Yes | No |  |  |  |
| 364 | Olusanya, et al. (2017) | D | Yes | Yes | Yes | Yes | Yes | Yes | Yes | Yes | No |  |  |  |
| 365 | Orabona, et al. (2015) | D | Yes | Yes | Yes | Yes | Yes | Yes | Yes | Yes | No |  |  |  |
| 366 | Ouzounian, et al. (2016) | D | Yes | Yes | Yes | Yes | Yes | Yes | Yes | Yes | No |  |  |  |
| 367 | Oylumlu, et al. (2014) | D | Yes | Yes | Yes | Yes | Yes | Yes | Yes | Yes | No |  |  |  |
| 368 | Oztas, et al. (2016) | D | Yes | Yes | Yes | Yes | Yes | Yes | Yes | Yes | No |  |  |  |
| 369 | Palatnik, et al. (2016) | D | Yes | Yes | Yes | Yes | Yes | Yes | Yes | Yes | No |  |  |  |
| 370 | Papaioannou, et al. (2011) | D | Yes | Yes | Yes | Yes | Yes | Yes | Yes | Yes | No |  |  |  |
| 371 | Park, et al. (2015) | D | Yes | Yes | Yes | Yes | Yes | Yes | Yes | Yes | No |  |  |  |
| 372 | Perales, et al. (2017) | D | Yes | Yes | Yes | Yes | Yes | Yes | Yes | Yes | No |  |  |  |
| 373 | Poon, et al. (2010) | D | Yes | Yes | Yes | Yes | Yes | Yes | Yes | Yes | No |  |  |  |
| 374 | Poon, et al. (2015) | D | Yes | Yes | Yes | Yes | Yes | Yes | Yes | Yes | No |  |  |  |
| 375 | Posthumus, et al. (2016) | D | Yes | Yes | Yes | Yes | Yes | Yes | Yes | Yes | No |  |  |  |
| 376 | Punnose, et al. (2020) | D | Yes | Yes | Yes | Yes | Yes | Yes | Yes | Yes | No |  |  |  |
| 377 | Ramos-Medina, et al. (2013) | D | Yes | Yes | Yes | Yes | Yes | Yes | Yes | Yes | No |  |  |  |
| 378 | Ren, et al. (2020) | D | Yes | Yes | Yes | Yes | Yes | Yes | Yes | Yes | No |  |  |  |
| 379 | Rhenman, et al. (2015) | D | Yes | Yes | Yes | Yes | Yes | Yes | Yes | Yes | No |  |  |  |
| 380 | Riboni, et al. (2012) | D | Yes | Yes | Yes | Yes | Yes | Yes | Yes | Yes | No |  |  |  |
| 381 | Rizzo, et al. (2019) | D | Yes | Yes | Yes | Yes | Yes | Yes | Yes | Yes | No |  |  |  |
| 382 | Rizzo, et al. (2019) | D | Yes | Yes | Yes | Yes | Yes | Yes | Yes | Yes | No |  |  |  |
| 383 | Rizzo, et al. (2019) | D | Yes | Yes | Yes | Yes | Yes | Yes | Yes | Yes | No |  |  |  |
| 384 | Rizzo, et al. (2019) | D | Yes | Yes | Yes | Yes | Yes | Yes | Yes | Yes | No |  |  |  |
| 385 | Sallmon, et al. (2018) | D | Yes | Yes | Yes | Yes | Yes | Yes | Yes | Yes | No |  |  |  |
| 386 | Sanhal, et al. (2016) | D | Yes | Yes | Yes | Yes | Yes | Yes | Yes | Yes | No |  |  |  |
| 387 | Schneuer, et al. (2012) | D | Yes | Yes | Yes | Yes | Yes | Yes | Yes | Yes | No |  |  |  |
| 388 | Schneuer, et al. (2014) | D | Yes | Yes | Yes | Yes | Yes | Yes | Yes | Yes | No |  |  |  |
| 389 | Schneuer, et al. (2014) | D | Yes | Yes | Yes | Yes | Yes | Yes | Yes | Yes | No |  |  |  |
| 390 | Schneuer, et al. (2015) | D | Yes | Yes | Yes | Yes | Yes | Yes | Yes | Yes | No |  |  |  |
| 391 | Scifres, et al. (2011) | D | Yes | Yes | Yes | Yes | Yes | Yes | Yes | Yes | No |  |  |  |
| 392 | Sepúlveda-Martínez, et al. (2017) | D | Yes | Yes | Yes | Yes | Yes | Yes | Yes | Yes | No |  |  |  |
| 393 | Sepúlveda-Martínez, et al. (2019) | D | Yes | Yes | Yes | Yes | Yes | Yes | Yes | Yes | No |  |  |  |
| 394 | Seravalli, et al. (2014) | D | Yes | Yes | Yes | Yes | Yes | Yes | Yes | Yes | No |  |  |  |
| 395 | Sharma, et al. (2019) | D | Yes | Yes | Yes | Yes | Yes | Yes | Yes | Yes | No |  |  |  |
| 396 | Shinohara, et al. (2017) | D | Yes | Yes | Yes | Yes | Yes | Yes | Yes | Yes | No |  |  |  |
| 397 | Shinohara, et al. (2020) | D | Yes | Yes | Yes | Yes | Yes | Yes | Yes | Yes | No |  |  |  |
| 398 | Sirico, et al. (2019) | D | Yes | Yes | Yes | Yes | Yes | Yes | Yes | Yes | No |  |  |  |
| 399 | Skupski, et al. (2013) | D | Yes | Yes | Yes | Yes | Yes | Yes | Yes | Yes | No |  |  |  |
| 400 | Sonalkar, et al. (2020) | D | Yes | Yes | Yes | Yes | Yes | Yes | Yes | Yes | No |  |  |  |
| 401 | Srinivas, et al. (2010) | D | Yes | Yes | Yes | Yes | Yes | Yes | Yes | Yes | No |  |  |  |
| 402 | Teoh, et al. (2019) | D | Yes | Yes | Yes | Yes | Yes | Yes | Yes | Yes | No |  |  |  |
| 403 | Torricelli, et al. (2011) | D | Yes | Yes | Yes | Yes | Yes | Yes | Yes | Yes | No |  |  |  |
| 404 | Tsiartas, et al. (2012) | D | Yes | Yes | Yes | Yes | Yes | Yes | Yes | Yes | No |  |  |  |
| 405 | Tuuli, et al. (2011) | D | Yes | Yes | Yes | Yes | Yes | Yes | Yes | Yes | No |  |  |  |
| 406 | Vallikkannu, et al. (2017) | D | Yes | Yes | Yes | Yes | Yes | Yes | Yes | Yes | No |  |  |  |
| 407 | Weiner, et al. (2016) | D | Yes | Yes | Yes | Yes | Yes | Yes | Yes | Yes | No |  |  |  |
| 408 | White, et al. (2012) | D | Yes | Yes | Yes | Yes | Yes | Yes | Yes | Yes | No |  |  |  |
| 409 | Wilson, et al. (2013) | D | Yes | Yes | Yes | Yes | Yes | Yes | Yes | Yes | No |  |  |  |
| 410 | Xiang, et al. (2019) | D | Yes | Yes | Yes | Yes | Yes | Yes | Yes | Yes | No |  |  |  |
| 411 | Xu, et al. (2020) | D | Yes | Yes | Yes | Yes | Yes | Yes | Yes | Yes | No |  |  |  |
| 412 | Yang, et al. (2018) | D | Yes | Yes | Yes | Yes | Yes | Yes | Yes | Yes | No |  |  |  |
| 413 | Yefet, et al. (2020) | D | Yes | Yes | Yes | Yes | Yes | Yes | Yes | Yes | No |  |  |  |
| 414 | Yerlikaya, et al. (2016) | D | Yes | Yes | Yes | Yes | Yes | Yes | Yes | Yes | No |  |  |  |
| 415 | Youssef, et al. (2014) | D | Yes | Yes | Yes | Yes | Yes | Yes | Yes | Yes | No |  |  |  |
| 416 | Zalel, et al. (2017) | D | Yes | Yes | Yes | Yes | Yes | Yes | Yes | Yes | No |  |  |  |
| 417 | Zanardini, et al. (2014) | D | Yes | Yes | Yes | Yes | Yes | Yes | Yes | Yes | No |  |  |  |
| 418 | Zhong, et al. (2011) | D | Yes | Yes | Yes | Yes | Yes | Yes | Yes | Yes | No |  |  |  |
| 419 | Shaniba Asmi P, et al. (2018) | D | Yes | Yes | Yes | Yes | Yes | Yes | Yes | Yes | No |  |  |  |
| 420 | Carty, et al. (2011) | DV | Yes | Yes | Yes | Yes | Yes | Yes | Yes | No |  |  |  |  |
| 421 | Cashen, et al. (2018) | D | Yes | Yes | Yes | Yes | Yes | Yes | Yes | No |  |  |  |  |
| 422 | Ghi, et al. (2016) | D | Yes | Yes | Yes | Yes | Yes | Yes | Yes | No |  |  |  |  |
| 423 | Ambalavanan, et al. (2005) | D | Yes | Yes | Yes | Yes | Yes | Yes | No |  |  |  |  |  |
| 424 | Beksac, et al. (2018) | D | Yes | Yes | Yes | Yes | Yes | Yes | No |  |  |  |  |  |
| 425 | Bolón-Canedo, et al. (2015) | D | Yes | Yes | Yes | Yes | Yes | Yes | No |  |  |  |  |  |
| 426 | Demailly, et al. (20-0) | D | Yes | Yes | Yes | Yes | Yes | Yes | No |  |  |  |  |  |
| 427 | Devoe, et al. (1995) | D | Yes | Yes | Yes | Yes | Yes | Yes | No |  |  |  |  |  |
| 428 | Frick, et al. (2016) | D | Yes | Yes | Yes | Yes | Yes | Yes | No |  |  |  |  |  |
| 429 | Bahado-Singh RO, et al. (2018) | D | Yes | Yes | Yes | Yes | Yes | Yes | No |  |  |  |  |  |
| 430 | Borowska M, et al. (2018) | D | Yes | Yes | Yes | Yes | Yes | Yes | No |  |  |  |  |  |
| 431 | Cai, et al. (2019) | D | Yes | Yes | Yes | Yes | Yes | No |  |  |  |  |  |  |
| 432 | Caruana, et al. (2002) | D | Yes | Yes | Yes | Yes | Yes | No |  |  |  |  |  |  |
| 433 | Courtney, et al. (2008) | D | Yes | Yes | Yes | Yes | Yes | No |  |  |  |  |  |  |
| 434 | Moreira MWL, et al. (2018) | D | Yes | Yes | Yes | Yes | Yes | No |  |  |  |  |  |  |
| 435 | Moreira MWL, et al. (2019) | D | Yes | Yes | Yes | Yes | Yes | No |  |  |  |  |  |  |
| 436 | Moreira MWL, et al. (2019) | D | Yes | Yes | Yes | Yes | Yes | No |  |  |  |  |  |  |
| 437 | Vullings R, et al. (2013) | D | Yes | Yes | Yes | Yes | Yes | No |  |  |  |  |  |  |
| 438 | Yarlapati AR, et al. (2017) | D | Yes | Yes | Yes | Yes | Yes | No |  |  |  |  |  |  |
| 439 | Adeyinka, et al. (2019) | D | Yes | Yes | Yes | Yes | Yes |  |  |  |  |  |  |  |
| 440 | Anuwutnavin, et al. (2016) | D | Yes | Yes | Yes | Yes | Yes |  |  |  |  |  |  |  |
| 441 | Assawapalanggool, et al. (2017) | D | Yes | Yes | Yes | Yes | Yes |  |  |  |  |  |  |  |
| 442 | Ben-Haroush, et al. (2011) | D | Yes | Yes | Yes | Yes | Yes |  |  |  |  |  |  |  |
| 443 | Berger, et al. (2017) | D | Yes | Yes | Yes | Yes | Yes |  |  |  |  |  |  |  |
| 444 | Bharatha, et al. (2012) | D | Yes | Yes | Yes | Yes | Yes |  |  |  |  |  |  |  |
| 445 | Brown, et al. (2018) | D | Yes | Yes | Yes | Yes | Yes |  |  |  |  |  |  |  |
| 446 | Campbell, et al. (2016) | D | Yes | Yes | Yes | Yes | Yes |  |  |  |  |  |  |  |
| 447 | Chalam, et al. (2011) | D | Yes | Yes | Yes | Yes | Yes |  |  |  |  |  |  |  |
| 448 | Chen, et al. (2012) | D | Yes | Yes | Yes | Yes | Yes |  |  |  |  |  |  |  |
| 449 | Cheng, et al. (2012) | D | Yes | Yes | Yes | Yes | Yes |  |  |  |  |  |  |  |
| 450 | Cobo, et al. (2020) | D | Yes | Yes | Yes | Yes | Yes |  |  |  |  |  |  |  |
| 451 | Do, et al. (2020) | D | Yes | Yes | Yes | Yes | Yes |  |  |  |  |  |  |  |
| 452 | Du, et al. (2020) | D | Yes | Yes | Yes | Yes | Yes |  |  |  |  |  |  |  |
| 453 | Ekizler, et al. (2019) | D | Yes | Yes | Yes | Yes | Yes |  |  |  |  |  |  |  |
| 454 | El-Haieg, et al. (2019) | D | Yes | Yes | Yes | Yes | Yes |  |  |  |  |  |  |  |
| 455 | Fernández, et al. (2016) | D | Yes | Yes | Yes | Yes | Yes |  |  |  |  |  |  |  |
| 456 | Fu, et al. (2016) | D | Yes | Yes | Yes | Yes | Yes |  |  |  |  |  |  |  |
| 457 | Gadaras, et al. (2009) | D | Yes | Yes | Yes | Yes | Yes |  |  |  |  |  |  |  |
| 458 | Gao, et al. (2015) | D | Yes | Yes | Yes | Yes | Yes |  |  |  |  |  |  |  |
| 459 | Graham, et al. (2014) | D | Yes | Yes | Yes | Yes | Yes |  |  |  |  |  |  |  |
| 460 | Grobman, et al. (2016) | D | Yes | Yes | Yes | Yes | Yes |  |  |  |  |  |  |  |
| 461 | Grossi, et al. (2016) | DV | Yes | Yes | Yes | Yes | Yes |  |  |  |  |  |  |  |
| 462 | Gutiérrez-Fragoso, et al. (2017) | D | Yes | Yes | Yes | Yes | Yes |  |  |  |  |  |  |  |
| 463 | Guzman-Barcenas, et al. (2016) | D | Yes | Yes | Yes | Yes | Yes |  |  |  |  |  |  |  |
| 464 | Hou, et al. (2018) | D | Yes | Yes | Yes | Yes | Yes |  |  |  |  |  |  |  |
| 465 | Hu, et al. (2019) | D | Yes | Yes | Yes | Yes | Yes |  |  |  |  |  |  |  |
| 466 | Irles, et al. (2018) | D | Yes | Yes | Yes | Yes | Yes |  |  |  |  |  |  |  |
| 467 | Kang, et al. (2012) | D | Yes | Yes | Yes | Yes | Yes |  |  |  |  |  |  |  |
| 468 | Kebapcilar, et al. (2016) | D | Yes | Yes | Yes | Yes | Yes |  |  |  |  |  |  |  |
| 469 | Kim, et al. (2017) | D | Yes | Yes | Yes | Yes | Yes |  |  |  |  |  |  |  |
| 470 | Kim, et al. (2019) | D | Yes | Yes | Yes | Yes | Yes |  |  |  |  |  |  |  |
| 471 | Kulan, et al. (2019) | D | Yes | Yes | Yes | Yes | Yes |  |  |  |  |  |  |  |
| 472 | Kuwata, et al. (2018) | D | Yes | Yes | Yes | Yes | Yes |  |  |  |  |  |  |  |
| 473 | Labenne, et al. (2011) | D | Yes | Yes | Yes | Yes | Yes |  |  |  |  |  |  |  |
| 474 | Lédée, et al. (2010) | D | Yes | Yes | Yes | Yes | Yes |  |  |  |  |  |  |  |
| 475 | Lemmens, et al. (2016) | D | Yes | Yes | Yes | Yes | Yes |  |  |  |  |  |  |  |
| 476 | Li, et al. (2017) | D | Yes | Yes | Yes | Yes | Yes |  |  |  |  |  |  |  |
| 477 | Litwińska, et al. (2017) | D | Yes | Yes | Yes | Yes | Yes |  |  |  |  |  |  |  |
| 478 | Ludwin, et al. (2014) | D | Yes | Yes | Yes | Yes | Yes |  |  |  |  |  |  |  |
| 479 | Melamed, et al. (2014) | D | Yes | Yes | Yes | Yes | Yes |  |  |  |  |  |  |  |
| 480 | Miao, et al. (2018) | D | Yes | Yes | Yes | Yes | Yes |  |  |  |  |  |  |  |
| 481 | Mumusoglu, et al. (2017) | D | Yes | Yes | Yes | Yes | Yes |  |  |  |  |  |  |  |
| 482 | Nadim, et al. (2020) | D | Yes | Yes | Yes | Yes | Yes |  |  |  |  |  |  |  |
| 483 | Namburete, et al. (2015) | D | Yes | Yes | Yes | Yes | Yes |  |  |  |  |  |  |  |
| 484 | Nombo, et al. (2018) | D | Yes | Yes | Yes | Yes | Yes |  |  |  |  |  |  |  |
| 485 | Osmanağaoğlu, et al. (2014) | D | Yes | Yes | Yes | Yes | Yes |  |  |  |  |  |  |  |
| 486 | Özdemir, et al. (2018) | D | Yes | Yes | Yes | Yes | Yes |  |  |  |  |  |  |  |
| 487 | Peled, et al. (2011) | D | Yes | Yes | Yes | Yes | Yes |  |  |  |  |  |  |  |
| 488 | Pereira, et al. (2017) | D | Yes | Yes | Yes | Yes | Yes |  |  |  |  |  |  |  |
| 489 | Pisani, et al. (2016) | D | Yes | Yes | Yes | Yes | Yes |  |  |  |  |  |  |  |
| 490 | Pomorski, et al. (2014) | D | Yes | Yes | Yes | Yes | Yes |  |  |  |  |  |  |  |
| 491 | Porcelli, et al. (2014) | D | Yes | Yes | Yes | Yes | Yes |  |  |  |  |  |  |  |
| 492 | Quantin, et al. (2013) | D | Yes | Yes | Yes | Yes | Yes |  |  |  |  |  |  |  |
| 493 | Rad, et al. (2019) | D | Yes | Yes | Yes | Yes | Yes |  |  |  |  |  |  |  |
| 494 | Raihan-Al-Masud, et al. (2020) | D | Yes | Yes | Yes | Yes | Yes |  |  |  |  |  |  |  |
| 495 | Rizzo, et al. (2016) | D | Yes | Yes | Yes | Yes | Yes |  |  |  |  |  |  |  |
| 496 | Romero-Ruiz, et al. (2019) | D | Yes | Yes | Yes | Yes | Yes |  |  |  |  |  |  |  |
| 497 | Rosenbloom, et al. (2019) | D | Yes | Yes | Yes | Yes | Yes |  |  |  |  |  |  |  |
| 498 | Rubio, et al. (2017) | D | Yes | Yes | Yes | Yes | Yes |  |  |  |  |  |  |  |
| 499 | Ruiz, et al. (2019) | D | Yes | Yes | Yes | Yes | Yes |  |  |  |  |  |  |  |
| 500 | Siristatidis, et al. (2016) | D | Yes | Yes | Yes | Yes | Yes |  |  |  |  |  |  |  |
| 501 | Sittiparn, et al. (2017) | D | Yes | Yes | Yes | Yes | Yes |  |  |  |  |  |  |  |
| 502 | Slaughter, et al. (2016) | D | Yes | Yes | Yes | Yes | Yes |  |  |  |  |  |  |  |
| 503 | Solis-Paredes, et al. (2017) | D | Yes | Yes | Yes | Yes | Yes |  |  |  |  |  |  |  |
| 504 | Stanfield, et al. (2019) | D | Yes | Yes | Yes | Yes | Yes |  |  |  |  |  |  |  |
| 505 | Thompson, et al. (2019) | D | Yes | Yes | Yes | Yes | Yes |  |  |  |  |  |  |  |
| 506 | Van Belle, et al. (2012) | D | Yes | Yes | Yes | Yes | Yes |  |  |  |  |  |  |  |
| 507 | Vigdor, et al. (2006) | D | Yes | Yes | Yes | Yes | Yes |  |  |  |  |  |  |  |
| 508 | Vilhena, et al. (2017) | D | Yes | Yes | Yes | Yes | Yes |  |  |  |  |  |  |  |
| 509 | Vollmar, et al. (2008) | D | Yes | Yes | Yes | Yes | Yes |  |  |  |  |  |  |  |
| 510 | Wang, et al. (2018) | D | Yes | Yes | Yes | Yes | Yes |  |  |  |  |  |  |  |
| 511 | Wen, et al. (2016) | D | Yes | Yes | Yes | Yes | Yes |  |  |  |  |  |  |  |
| 512 | Yafi, et al. (2013) | D | Yes | Yes | Yes | Yes | Yes |  |  |  |  |  |  |  |
| 513 | Yang, et al. (2018) | D | Yes | Yes | Yes | Yes | Yes |  |  |  |  |  |  |  |
| 514 | Yaqub, et al. (2014) | D | Yes | Yes | Yes | Yes | Yes |  |  |  |  |  |  |  |
| 515 | Ye, et al. (2019) | D | Yes | Yes | Yes | Yes | Yes |  |  |  |  |  |  |  |
| 516 | Ying, et al. (2016) | D | Yes | Yes | Yes | Yes | Yes |  |  |  |  |  |  |  |
| 517 | Younis, et al. (2010) | D | Yes | Yes | Yes | Yes | Yes |  |  |  |  |  |  |  |
| 518 | Yu, et al. (2008) | D | Yes | Yes | Yes | Yes | Yes |  |  |  |  |  |  |  |
| 519 | Yu, et al. (2009) | D | Yes | Yes | Yes | Yes | Yes |  |  |  |  |  |  |  |
| 520 | Yu, et al. (2017) | D | Yes | Yes | Yes | Yes | Yes |  |  |  |  |  |  |  |
| 521 | Zamora, et al. (2014) | D | Yes | Yes | Yes | Yes | Yes |  |  |  |  |  |  |  |
| 522 | Zandbaaf, et al. (2020) | D | Yes | Yes | Yes | Yes | Yes |  |  |  |  |  |  |  |
| 523 | Zhang, et al. (2016) | D | Yes | Yes | Yes | Yes | Yes |  |  |  |  |  |  |  |
| 524 | Zhao, et al. (2019) | D | Yes | Yes | Yes | Yes | Yes |  |  |  |  |  |  |  |
| 525 | Zhong, et al. (2019) | D | Yes | Yes | Yes | Yes | Yes |  |  |  |  |  |  |  |
| 526 | Caballero-Ruiz E, et al. (2016) | D | Yes | Yes | Yes | Yes | Yes |  |  |  |  |  |  |  |
| 527 | De Carli MM, et al. (2017) | D | Yes | Yes | Yes | Yes | Yes |  |  |  |  |  |  |  |
| 528 | Gentillon H, et al. (2017) | D | Yes | Yes | Yes | Yes | Yes |  |  |  |  |  |  |  |
| 529 | González-Recio O, et al. (2011) | D | Yes | Yes | Yes | Yes | Yes |  |  |  |  |  |  |  |
| 530 | Khan SR, et al. (2019) | D | Yes | Yes | Yes | Yes | Yes |  |  |  |  |  |  |  |
| 531 | Lin HC, et al. (2011) | D | Yes | Yes | Yes | Yes | Yes |  |  |  |  |  |  |  |
| 532 | Manna C, et al. (2013) | D | Yes | Yes | Yes | Yes | Yes |  |  |  |  |  |  |  |
| 533 | Maraci MA, et al. (2017) | D | Yes | Yes | Yes | Yes | Yes |  |  |  |  |  |  |  |
| 534 | Naimi AI, et al. (2018) | DV | Yes | Yes | Yes | Yes | Yes |  |  |  |  |  |  |  |
| 535 | Orlandi S, et al. (2016) | D | Yes | Yes | Yes | Yes | Yes |  |  |  |  |  |  |  |
| 536 | Papageorghiou AT, et al. (2016) | DV | Yes | Yes | Yes | Yes | Yes |  |  |  |  |  |  |  |
| 537 | Rittenhouse KJ, et al. (2019) | D | Yes | Yes | Yes | Yes | Yes |  |  |  |  |  |  |  |
| 538 | Rodriguez LM, et al. (2015) | D | Yes | Yes | Yes | Yes | Yes |  |  |  |  |  |  |  |
| 539 | Sarker A, et al. (2017) | D | Yes | Yes | Yes | Yes | Yes |  |  |  |  |  |  |  |
| 540 | Sridar P, et al. (2019) | D | Yes | Yes | Yes | Yes | Yes |  |  |  |  |  |  |  |
| 541 | Tylee DS, et al. (2017) | D | Yes | Yes | Yes | Yes | Yes |  |  |  |  |  |  |  |
| 542 | Volk M, et al. (2013) | DV | Yes | Yes | Yes | Yes | Yes |  |  |  |  |  |  |  |
| 543 | Yilmaz E, et al. (2013) | D | Yes | Yes | Yes | Yes | Yes |  |  |  |  |  |  |  |
| 544 | Akhtar, et al. (2019) | D | Yes | Yes | Yes | Yes | No |  |  |  |  |  |  |  |
| 545 | Alonso-Betanzos, et al. (1999) | D | Yes | Yes | Yes | Yes | No |  |  |  |  |  |  |  |
| 546 | Ayachi, et al. (2018) | D | Yes | Yes | Yes | Yes | No |  |  |  |  |  |  |  |
| 547 | Broeze, et al. (2012) | D | Yes | Yes | Yes | Yes | No |  |  |  |  |  |  |  |
| 548 | Chiogna, et al. (1996) | D | Yes | Yes | Yes | Yes | No |  |  |  |  |  |  |  |
| 549 | Mantini, et al. (2005) | D | Yes | Yes | Yes | Yes | No |  |  |  |  |  |  |  |
| 550 | Ahmed F, et al. (2019) | D | Yes | Yes | Yes | Yes | No |  |  |  |  |  |  |  |
| 551 | Moreira MWL, et al. (2018) | D | Yes | Yes | Yes | Yes | No |  |  |  |  |  |  |  |
| 552 | Akhavan, et al. (2017) | D | Yes | Yes | Yes | No |  |  |  |  |  |  |  |  |
| 553 | Alsayyari, et al. (2019) | D | Yes | Yes | Yes | No |  |  |  |  |  |  |  |  |
| 554 | Comani, et al. (2007) | D | Yes | Yes | Yes | No |  |  |  |  |  |  |  |  |
| 555 | Dietz, et al. (2010) | D | Yes | Yes | Yes | No |  |  |  |  |  |  |  |  |
| 556 | Du, et al. (2016) | D | Yes | Yes | Yes | No |  |  |  |  |  |  |  |  |
| 557 | Elson, et al. (2004) | D | Yes | Yes | Yes | No |  |  |  |  |  |  |  |  |
| 558 | Elson, et al. (2005) | D | Yes | Yes | Yes | No |  |  |  |  |  |  |  |  |
| 559 | Fruscalzo, et al. (2015) | D | Yes | Yes | Yes | No |  |  |  |  |  |  |  |  |
| 560 | Gasse, et al. (2019) | D | Yes | Yes | Yes | No |  |  |  |  |  |  |  |  |
| 561 | Guzmán-Huerta, et al. (2013) | D | Yes | Yes | Yes | No |  |  |  |  |  |  |  |  |
| 562 | Kondo, et al. (2018) | D | Yes | Yes | Yes | No |  |  |  |  |  |  |  |  |
| 563 | Leão Bde, et al. (1994) | D | Yes | Yes | Yes | No |  |  |  |  |  |  |  |  |
| 564 | Lee, et al. (2018) | D | Yes | Yes | Yes | No |  |  |  |  |  |  |  |  |
| 565 | Lim, et al. (2015) | D | Yes | Yes | Yes | No |  |  |  |  |  |  |  |  |
| 566 | Liu, et al. (2020) | D | Yes | Yes | Yes | No |  |  |  |  |  |  |  |  |
| 567 | Manuck, et al. (2016) | D | Yes | Yes | Yes | No |  |  |  |  |  |  |  |  |
| 568 | Marvin, et al. (1999) | D | Yes | Yes | Yes | No |  |  |  |  |  |  |  |  |
| 569 | McPherson, et al. (2014) | D | Yes | Yes | Yes | No |  |  |  |  |  |  |  |  |
| 570 | Milewska, et al. (2017) | D | Yes | Yes | Yes | No |  |  |  |  |  |  |  |  |
| 571 | Millischer, et al. (2015) | D | Yes | Yes | Yes | No |  |  |  |  |  |  |  |  |
| 572 | Monteith, et al. (2017) | D | Yes | Yes | Yes | No |  |  |  |  |  |  |  |  |
| 573 | Murphy, et al. (2015) | D | Yes | Yes | Yes | No |  |  |  |  |  |  |  |  |
| 574 | Naito, et al. (2010) | D | Yes | Yes | Yes | No |  |  |  |  |  |  |  |  |
| 575 | Oztas, et al. (2016) | D | Yes | Yes | Yes | No |  |  |  |  |  |  |  |  |
| 576 | Papadimitriou, et al. (1997) | D | Yes | Yes | Yes | No |  |  |  |  |  |  |  |  |
| 577 | Papadimitriou, et al. (1999) | D | Yes | Yes | Yes | No |  |  |  |  |  |  |  |  |
| 578 | Papanna, et al. (2015) | D | Yes | Yes | Yes | No |  |  |  |  |  |  |  |  |
| 579 | Pare, et al. (2014) | D | Yes | Yes | Yes | No |  |  |  |  |  |  |  |  |
| 580 | Parra-Saavedra, et al. (2013) | D | Yes | Yes | Yes | No |  |  |  |  |  |  |  |  |
| 581 | Payne, et al. (2020) | D | Yes | Yes | Yes | No |  |  |  |  |  |  |  |  |
| 582 | Reed, et al. (1997) | D | Yes | Yes | Yes | No |  |  |  |  |  |  |  |  |
| 583 | Ren, et al. (2015) | D | Yes | Yes | Yes | No |  |  |  |  |  |  |  |  |
| 584 | Roberts, et al. (2018) | D | Yes | Yes | Yes | No |  |  |  |  |  |  |  |  |
| 585 | Semenova, et al. (2018) | D | Yes | Yes | Yes | No |  |  |  |  |  |  |  |  |
| 586 | Simon, et al. (2014) | D | Yes | Yes | Yes | No |  |  |  |  |  |  |  |  |
| 587 | Sinclair, et al. (2018) | D | Yes | Yes | Yes | No |  |  |  |  |  |  |  |  |
| 588 | Siristatidis, et al. (2011) | D | Yes | Yes | Yes | No |  |  |  |  |  |  |  |  |
| 589 | Smith, et al. (2014) | D | Yes | Yes | Yes | No |  |  |  |  |  |  |  |  |
| 590 | Sur, et al. (2010) | D | Yes | Yes | Yes | No |  |  |  |  |  |  |  |  |
| 591 | Torgersen, et al. (1979) | D | Yes | Yes | Yes | No |  |  |  |  |  |  |  |  |
| 592 | Van Ravenswaaij, et al. (2011) | D | Yes | Yes | Yes | No |  |  |  |  |  |  |  |  |
| 593 | Velzel, et al. (2018) | D | Yes | Yes | Yes | No |  |  |  |  |  |  |  |  |
| 594 | Wang, et al. (2019) | D | Yes | Yes | Yes | No |  |  |  |  |  |  |  |  |
| 595 | Webster, et al. (2019) | D | Yes | Yes | Yes | No |  |  |  |  |  |  |  |  |
| 596 | Weiss, et al. (2019) | D | Yes | Yes | Yes | No |  |  |  |  |  |  |  |  |
| 597 | Ye, et al. (2018) | D | Yes | Yes | Yes | No |  |  |  |  |  |  |  |  |
| 598 | Zhang, et al. (2018) | D | Yes | Yes | Yes | No |  |  |  |  |  |  |  |  |
| 599 | Zhu, et al. (2012) | D | Yes | Yes | Yes | No |  |  |  |  |  |  |  |  |
| 600 | Dey A, et al. (2018) | D | Yes | Yes | Yes | No |  |  |  |  |  |  |  |  |
| 601 | Alansary, et al. (2019) | D | Yes | Yes | No |  |  |  |  |  |  |  |  |  |
| 602 | Altin, et al. (2018) | D | Yes | Yes | No |  |  |  |  |  |  |  |  |  |
| 603 | Alves, et al. (2014) | D | Yes | Yes | No |  |  |  |  |  |  |  |  |  |
| 604 | Amabebe, et al. (2019) | D | Yes | Yes | No |  |  |  |  |  |  |  |  |  |
| 605 | Arfi, et al. (2019) | D | Yes | Yes | No |  |  |  |  |  |  |  |  |  |
| 606 | Beksaç, et al. (1996) | D | Yes | Yes | No |  |  |  |  |  |  |  |  |  |
| 607 | Beksaç, et al. (20-0) | D | Yes | Yes | No |  |  |  |  |  |  |  |  |  |
| 608 | Beksaç, et al. (2034) | D | Yes | Yes | No |  |  |  |  |  |  |  |  |  |
| 609 | Braaten, et al. (1996) | D | Yes | Yes | No |  |  |  |  |  |  |  |  |  |
| 610 | Forkert, et al. (2011) | D | Yes | Yes | No |  |  |  |  |  |  |  |  |  |
| 611 | Raef, et al. (2019) | D | Yes | Yes | No |  |  |  |  |  |  |  |  |  |
| 612 | Spilka, et al. (2015) | D | Yes | Yes | No |  |  |  |  |  |  |  |  |  |
| 613 | Uyar, et al. (2009) | D | Yes | Yes | No |  |  |  |  |  |  |  |  |  |
| 614 | Shiyu Sara Huang CIL, et al. (2018) | D | Yes | Yes | No |  |  |  |  |  |  |  |  |  |
| 615 | Weber KA, et al. (2019) | D | Yes | Yes | No |  |  |  |  |  |  |  |  |  |
| 616 | Avni, et al. (2012) | D | Yes | No |  |  |  |  |  |  |  |  |  |  |
| 617 | Cao, et al. (2016) | D | Yes | No |  |  |  |  |  |  |  |  |  |  |
| 618 | Diab, et al. (2005) | D | Yes | No |  |  |  |  |  |  |  |  |  |  |
| 619 | Jurisica, et al. (1998) | D | Yes | No |  |  |  |  |  |  |  |  |  |  |
| 620 | Looney, et al. (2018) | D | Yes | No |  |  |  |  |  |  |  |  |  |  |
| 621 | Lovell, et al. (1997) | D | Yes | No |  |  |  |  |  |  |  |  |  |  |
| 622 | Grzymala-Busse JW, et al. (1994) | D | Yes | No |  |  |  |  |  |  |  |  |  |  |
| 623 | Guidi G, et al. (2014) | D | Yes | No |  |  |  |  |  |  |  |  |  |  |
| 624 | Gupta L, et al. (2011) | D | Yes | No |  |  |  |  |  |  |  |  |  |  |
| 625 | Patil SN, et al. (2019) | D | Yes | No |  |  |  |  |  |  |  |  |  |  |
| 626 | Bao, et al. (2019) |  | No |  |  |  |  |  |  |  |  |  |  |  |
| 627 | Boo, et al. (2015) |  | No |  |  |  |  |  |  |  |  |  |  |  |
| 628 | Branger, et al. (2018) |  | No |  |  |  |  |  |  |  |  |  |  |  |
| 629 | Bruno, et al. (2019) |  | No |  |  |  |  |  |  |  |  |  |  |  |
| 630 | Caballero Sanz, et al. (2018) |  | No |  |  |  |  |  |  |  |  |  |  |  |
| 631 | Chéles, et al. (2020) |  | No |  |  |  |  |  |  |  |  |  |  |  |
| 632 | Chen, et al. (2008) |  | No |  |  |  |  |  |  |  |  |  |  |  |
| 633 | Chen, et al. (2018) |  | No |  |  |  |  |  |  |  |  |  |  |  |
| 634 | Chiarelli, et al. (2018) |  | No |  |  |  |  |  |  |  |  |  |  |  |
| 635 | Czabański, et al. (2013) |  | No |  |  |  |  |  |  |  |  |  |  |  |
| 636 | Dithy, et al. (2019) |  | No |  |  |  |  |  |  |  |  |  |  |  |
| 637 | EBRAHIMZADEH, et al. (2015) |  | No |  |  |  |  |  |  |  |  |  |  |  |
| 638 | Etikan, et al. (2005) |  | No |  |  |  |  |  |  |  |  |  |  |  |
| 639 | Forest, et al. (2012) |  | No |  |  |  |  |  |  |  |  |  |  |  |
| 640 | Forest, et al. (2012) |  | No |  |  |  |  |  |  |  |  |  |  |  |
| 641 | GONG, et al. (2006) |  | No |  |  |  |  |  |  |  |  |  |  |  |
| 642 | Gratacos, et al. (2010) |  | No |  |  |  |  |  |  |  |  |  |  |  |
| 643 | Guo, et al. (2013) |  | No |  |  |  |  |  |  |  |  |  |  |  |
| 644 | He, et al. (2013) |  | No |  |  |  |  |  |  |  |  |  |  |  |
| 645 | He, et al. (2015) |  | No |  |  |  |  |  |  |  |  |  |  |  |
| 646 | Heredia-Olivera, et al. (2016) |  | No |  |  |  |  |  |  |  |  |  |  |  |
| 647 | Iftikhar, et al. (2020) |  | No |  |  |  |  |  |  |  |  |  |  |  |
| 648 | Jeschke, et al. |  | No |  |  |  |  |  |  |  |  |  |  |  |
| 649 | Leão, et al. (1995) |  | No |  |  |  |  |  |  |  |  |  |  |  |
| 650 | Leão, et al. (1996) |  | No |  |  |  |  |  |  |  |  |  |  |  |
| 651 | Liao, et al. (2018) |  | No |  |  |  |  |  |  |  |  |  |  |  |
| 652 | Lin, et al. (2018) |  | No |  |  |  |  |  |  |  |  |  |  |  |
| 653 | Lu, et al. (2016) |  | No |  |  |  |  |  |  |  |  |  |  |  |
| 654 | Marini, et al. (2007) |  | No |  |  |  |  |  |  |  |  |  |  |  |
| 655 | Mateen, et al. (2020) |  | No |  |  |  |  |  |  |  |  |  |  |  |
| 656 | Meng-yao, et al. (2019) |  | No |  |  |  |  |  |  |  |  |  |  |  |
| 657 | Muscatello, et al. (2014) |  | No |  |  |  |  |  |  |  |  |  |  |  |
| 658 | Patumanond, et al. (2012) |  | No |  |  |  |  |  |  |  |  |  |  |  |
| 659 | Prema, et al. (2019) |  | No |  |  |  |  |  |  |  |  |  |  |  |
| 660 | Qiu, et al. (2018) |  | No |  |  |  |  |  |  |  |  |  |  |  |
| 661 | Rezaei, et al. (2019) |  | No |  |  |  |  |  |  |  |  |  |  |  |
| 662 | Rezaei, et al. (2020) |  | No |  |  |  |  |  |  |  |  |  |  |  |
| 663 | Saadati, et al. (2018) |  | No |  |  |  |  |  |  |  |  |  |  |  |
| 664 | SADAT, et al. (2004) |  | No |  |  |  |  |  |  |  |  |  |  |  |
| 665 | Seufert, et al. (2000) |  | No |  |  |  |  |  |  |  |  |  |  |  |
| 666 | Steffann, et al. (2005) |  | No |  |  |  |  |  |  |  |  |  |  |  |
| 667 | Tan, et al. (2019) |  | No |  |  |  |  |  |  |  |  |  |  |  |
| 668 | Tylcz, et al. (2020) |  | No |  |  |  |  |  |  |  |  |  |  |  |
| 669 | Wald, et al. (2007) |  | No |  |  |  |  |  |  |  |  |  |  |  |
| 670 | Wang, et al. (2007) |  | No |  |  |  |  |  |  |  |  |  |  |  |
| 671 | Wang, et al. (2019) |  | No |  |  |  |  |  |  |  |  |  |  |  |
| 672 | Wang, et al. (2019) |  | No |  |  |  |  |  |  |  |  |  |  |  |
| 673 | Wischnik, et al. (1993) |  | No |  |  |  |  |  |  |  |  |  |  |  |
| 674 | Wu, et al. (2005) |  | No |  |  |  |  |  |  |  |  |  |  |  |
| 675 | Xiong, et al. (2017) |  | No |  |  |  |  |  |  |  |  |  |  |  |
| 676 | Xu, et al. (2014) |  | No |  |  |  |  |  |  |  |  |  |  |  |
| 677 | Yi, et al. (1998) |  | No |  |  |  |  |  |  |  |  |  |  |  |
| 678 | Yoffe, et al. (2019) |  | No |  |  |  |  |  |  |  |  |  |  |  |
| 679 | Zhou, et al. (2008) |  | No |  |  |  |  |  |  |  |  |  |  |  |
| 680 | Francis F, et al. |  | No |  |  |  |  |  |  |  |  |  |  |  |

^a^ Sorted from the studies with most complete to incomplete criteria

# Table S4. List of studies for full text review. ^a, b^

| # | Full Citation |
| --- | --- |
|  |  |
| 1 | Artzi, N. S., Shilo, S., Hadar, E., Rossman, H., Barbash-Hazan, S., Ben-Haroush, A., . . . Segal, E. (2020). Prediction of gestational diabetes based on nationwide electronic health records. Nat Med, 26(1), 71-76. doi:10.1038/s41591-019-0724-8 |
| 2 | Benhalima, K., Van Crombrugge, P., Moyson, C., Verhaeghe, J., Vandeginste, S., Verlaenen, H., . . . Mathieu, C. (2020). Estimating the risk of gestational diabetes mellitus based on the 2013 WHO criteria: a prediction model based on clinical and biochemical variables in early pregnancy. Acta Diabetol. doi:10.1007/s00592-019-01469-5 |
| 3 | Blank C, Wildeboer RR, DeCroo I, et al. Prediction of implantation after blastocyst transfer in *in vitro* fertilization: A machine-learning perspective. Fertility and Sterility 2019;111:318-26. doi: https://doi.org/10.1016/j.fertnstert.2018.10.030. |
| 4 | Borup R, Thuesen LL, Andersen CY, et al. Competence classification of cumulus and granulosa cell transcriptome in embryos matched by morphology and female age. PLoS ONE 2016;11. doi: https://doi.org/10.1371/journal.pone.0153562. |
| 5 | de Wilde, M. A., Veltman-Verhulst, S. M., Goverde, A. J., Lambalk, C. B., Laven, J. S., Franx, A., . . . Fauser, B. C. (2014). Preconception predictors of gestational diabetes: a multicentre prospective cohort study on the predominant complication of pregnancy in polycystic ovary syndrome. Hum Reprod, 29(6), 1327-1336. doi:10.1093/humrep/deu077 |
| 6 | Mardy, A. H., Ananth, C. V., Grobman, W. A., & Gyamfi-Bannerman, C. (2016). A prediction model of vaginal birth after cesarean in the preterm period. Am J Obstet Gynecol, 215(4), 513.e511-517. doi:10.1016/j.ajog.2016.05.039 |
| 7 | Maroufizadeh, S., Amini, P., Hosseini, M., Almasi-Hashiani, A., Mohammadi, M., Navid, B., & Omani-Samani, R. (2018). Determinants of Cesarean Section among Primiparas: A Comparison of Classification Methods. Iran J Public Health, 47(12), 1913-1922. |
| 8 | Milewski, R., Milewska, A. J., Więsak, T., & Morgan, A. (2013). Comparison of artificial neural networks and logistic regression analysis in pregnancy prediction using the *in vitro* fertilization treatment. Studies in Logic, Grammar and Rhetoric, 35(1), 39-48. |
| 9 | Rinaudo, P., Shen, S., Hua, J., Qian, S., Prabhu, U., Garcia, E., . . . Andrews, C. (2012). (1)H NMR based profiling of spent culture media cannot predict success of implantation for day 3 human embryos. J Assist Reprod Genet, 29(12), 1435-1442. doi:10.1007/s10815-012-9877-9 |
| 10 | Sananes, N., Meyer, N., Gaudineau, A., Aissi, G., Boudier, E., Fritz, G., . . . Favre, R. (2013). Prediction of spontaneous preterm delivery in the first trimester of pregnancy. Eur J Obstet Gynecol Reprod Biol, 171(1), 18-22. doi:10.1016/j.ejogrb.2013.07.042 |
| 11 | Sandström, A., Snowden, J. M., Höijer, J., Bottai, M., & Wikström, A. K. (2019). Clinical risk assessment in early pregnancy for preeclampsia in nulliparous women: A population based cohort study. PLoS One, 14(11), e0225716. doi:10.1371/journal.pone.0225716 |
| 12 | Shi, W., Zhang, S., Zhao, W., Xia, X., Wang, M., Wang, H., . . . Shi, J. (2013). Factors related to clinical pregnancy after vitrified–warmed embryo transfer: a retrospective and multivariate logistic regression analysis of 2313 transfer cycles. Human Reproduction, 28(7), 1768-1775. |
| 13 | Sufriyana, H., Wu, Y. W., & Su, E. C. (2020). Artificial intelligence-assisted prediction of preeclampsia: Development and external validation of a nationwide health insurance dataset of the BPJS Kesehatan in Indonesia. EBioMedicine, 54, 102710. doi:10.1016/j.ebiom.2020.102710 |
| 14 | Tessmer-Tuck, J. A., El-Nashar, S. A., Racek, A. R., Lohse, C. M., Famuyide, A. O., & Wick, M. J. (2014). Predicting vaginal birth after cesarean section: a cohort study. Gynecol Obstet Invest, 77(2), 121-126. doi:10.1159/000357757 |
| 15 | Thériault, S., Giguère, Y., Massé, J., Girouard, J., & Forest, J. C. (2016). Early prediction of gestational diabetes: a practical model combining clinical and biochemical markers. Clin Chem Lab Med, 54(3), 509-518. doi:10.1515/cclm-2015-0537 |
| 16 | Tran, D., Cooke, S., Illingworth, P. J., & Gardner, D. K. (2019). Deep learning as a predictive tool for fetal heart pregnancy following time-lapse incubation and blastocyst transfer. Hum Reprod, 34(6), 1011-1018. doi:10.1093/humrep/dez064 |
| 17 | Troisi J, Landolfi A, Sarno L, et al. A metabolomics-based approach for non-invasive screening of fetal central nervous system anomalies. Metabolomics 2018;14. doi: https://doi.org/10.1007/s11306-018-1370-8. |
| 18 | van Baaren, G. J., Bruijn, M. M., Vis, J. Y., Wilms, F. F., Oudijk, M. A., Kwee, A., . . . Mol, B. W. (2015). Risk factors for preterm delivery: do they add to fetal fibronectin testing and cervical length measurement in the prediction of preterm delivery in symptomatic women? Eur J Obstet Gynecol Reprod Biol, 192, 79-85. doi:10.1016/j.ejogrb.2015.05.004 |
| 19 | van der Tuuk, K., van Pampus, M. G., Koopmans, C. M., Aarnoudse, J. G., van den Berg, P. P., van Beek, J. J., . . . Groen, H. (2015). Prediction of cesarean section risk in women with gestational hypertension or mild preeclampsia at term. Eur J Obstet Gynecol Reprod Biol, 191, 23-27. doi:10.1016/j.ejogrb.2015.05.009 |
| 20 | Weber, A., Darmstadt, G. L., Gruber, S., Foeller, M. E., Carmichael, S. L., Stevenson, D. K., & Shaw, G. M. (2018). Application of machine-learning to predict early spontaneous preterm birth among nulliparous non-Hispanic black and white women. Ann Epidemiol, 28(11), 783-789.e781. doi:10.1016/j.annepidem.2018.08.008 |
| 21 | Xing, Y. P., Qi, X. Y., Wang, X. Z., & Yang, F. Z. (2019). Development of a Modified Score System as Prediction Model for Successful Vaginal Birth After Cesarean Delivery. Clin Transl Sci, 12(1), 53-57. doi:10.1111/cts.12603 |
| 22 | Yang, H., Zhu, C., Ma, Q., Long, Y., & Cheng, Z. (2015). Variations of blood cells in prediction of gestational diabetes mellitus. J Perinat Med, 43(1), 89-93. doi:10.1515/jpm-2014-0007 |
| 23 | Zheng, T., Ye, W., Wang, X., Li, X., Zhang, J., Little, J., . . . Zhang, L. (2019). A simple model to predict risk of gestational diabetes mellitus from 8 to 20 weeks of gestation in Chinese women. BMC Pregnancy Childbirth, 19(1), 252. doi:10.1186/s12884-019-2374-8 |
| 24 | Abbas SA, Riaz R, Kazmi SZH, Rizvi SS, Kwon SJ. Cause analysis of caesarian sections and application of machine learning methods for classification of birth data. IEEE Access 2018;6:67555-61. doi: https://doi.org/10.1109/ACCESS.2018.2879115. |
| 25 | Amini, P., Maroufizadeh, S., Samani, R. O., Hamidi, O., & Sepidarkish, M. (2017). Prevalence and Determinants of Preterm Birth in Tehran, Iran: A Comparison between Logistic Regression and Decision Tree Methods. Osong Public Health Res Perspect, 8(3), 195-200. doi:10.24171/j.phrp.2017.8.3.06 |
| 26 | Balani, J., Hyer, S. L., Shehata, H., & Mohareb, F. (2018). Visceral fat mass as a novel risk factor for predicting gestational diabetes in obese pregnant women. Obstet Med, 11(3), 121-125. doi:10.1177/1753495x17754149 |
| 27 | Bastek, J. A., Sammel, M. D., Srinivas, S. K., McShea, M. A., Foreman, M. N., Elovitz, M. A., & Metlay, J. P. (2012). Clinical prediction rules for preterm birth in patients presenting with preterm labor. Obstet Gynecol, 119(6), 1119-1128. doi:10.1097/AOG.0b013e31825503e5 |
| 28 | Carlsson Fagerberg, M., & Källén, K. (2020). Third-trimester prediction of successful vaginal birth after one cesarean delivery-A Swedish model. Acta Obstet Gynecol Scand, 99(5), 660-668. doi:10.1111/aogs.13783 |
| 29 | Chen L, Hao Y. Feature extraction and classification of ehg between pregnancy and labour group using hilbert-huang transform and extreme learning machine. Comput Math Methods Med 2017;2017:7949507. doi: https://doi.org/10.1155/2017/7949507. |
| 30 | Chen, L., Hao, Y., & Hu, X. (2019). Detection of preterm birth in electrohysterogram signals based on wavelet transform and stacked sparse autoencoder. PLoS One, 14(4), e0214712. doi:10.1371/journal.pone.0214712 |
| 31 | de Oliveira, R. V., Martins Mda, G., Rios, L. T., Araujo Júnior, E., Simões, V. M., Nardozza, L. M., & Moron, A. F. (2012). Predictive model for spontaneous preterm labor among pregnant women with contractions and intact amniotic membranes. Arch Gynecol Obstet, 286(4), 893-900. doi:10.1007/s00404-012-2397-0 |
| 32 | Despotovic D, Zec A, Mladenovic K, Radin N, Turukalo TL. A machine learning approach for an early prediction of preterm delivery. 16th International Symposium on Intelligent Systems and Informatics (SISY) Year:265-70. doi: https://doi.org/https://doi.org/10.1109/SISY.2018.8524818. |
| 33 | Fagerberg, M. C., Maršál, K., & Källén, K. (2015). Predicting the chance of vaginal delivery after one cesarean section: validation and elaboration of a published prediction model. Eur J Obstet Gynecol Reprod Biol, 188, 88-94. doi:10.1016/j.ejogrb.2015.02.031 |
| 34 | Fergus P, Cheung P, Hussain A, Al-Jumeily D, Dobbins C, Iram S. Prediction of preterm deliveries from ehg signals using machine learning. PLoS One 2013;8. doi: https://doi.org/10.1371/journal.pone.0077154. |
| 35 | Fergus P, Idowu I, Hussain A, Dobbins C. Advanced artificial neural network classification for detecting preterm births using ehg records. Neurocomputing 2016;188:42-49. doi: https://doi.org/https://doi.org/10.1016/j.neucom.2015.01.107. |
| 36 | Fergus P, Hussain A, Al-Jumeily D, Huang DS, Bouguila N. Classification of caesarean section and normal vaginal deliveries using foetal heart rate signals and advanced machine learning algorithms. Biomed Eng Online 2017;16:89. doi: https://doi.org/10.1186/s12938-017-0378-z. |
| 37 | Fergus P, Montanez A, Abdulaimma B, Lisboa P, Chalmers C, Pineles B. Utilising deep learning and genome wide association studies for epistatic-driven preterm birth classification in african-american women. IEEE/ACM Trans Comput Biol Bioinform 2018. doi: https://doi.org/10.1109/TCBB.2018.2868667. |
| 38 | Fiset S, Martel A, Glanc P, Barrett J, Melamed N. Prediction of spontaneous preterm birth among twin gestations using machine learning and texture analysis of cervical ultrasound images. University of Toronto Medical Journal 2019;96:6-9. doi, PMID. |
| 39 | Garcés, M. F., Sanchez, E., Cardona, L. F., Simanca, E. L., González, I., Leal, L. G., . . . Caminos, J. E. (2015). Maternal Serum Meteorin Levels and the Risk of Preeclampsia. PLoS One, 10(6), e0131013. doi:10.1371/journal.pone.0131013 |
| 40 | Guo, Z., Yang, F., Zhang, J., Zhang, Z., Li, K., Tian, Q., . . . Yang, X. (2020). Whole-Genome Promoter Profiling of Plasma DNA Exhibits Diagnostic Value for Placenta-Origin Pregnancy Complications. Adv Sci (Weinh), 7(7), 1901819. doi:10.1002/advs.201901819 |
| 41 | Hamdi, M. A., Limem, M., & Maaref, M. A. (2019). Detection and Classification of Nonstationary Signals: Application to Uterine EMG for Prognostication of Premature Delivery. Neurophysiology, 51(4), 272-280. doi:10.1007/s11062-019-09821-9 |
| 42 | Lee, K. S., & Ahn, K. H. (2019). Artificial Neural Network Analysis of Spontaneous Preterm Labor and Birth and Its Major Determinants. J Korean Med Sci, 34(16), e128. doi:10.3346/jkms.2019.34.e128 |
| 43 | Macones, G. A., Hausman, N., Edelstein, R., Stamilio, D. M., & Marder, S. J. (2001). Predicting outcomes of trials of labor in women attempting vaginal birth after cesarean delivery: a comparison of multivariate methods with neural networks. Am J Obstet Gynecol, 184(3), 409-413. doi:10.1067/mob.2001.109386 |
| 44 | Mas-Cabo, J., Prats-Boluda, G., Garcia-Casado, J., Alberola-Rubio, J., Perales, A., & Ye-Lin, Y. (2019). Design and Assessment of a Robust and Generalizable ANN-Based Classifier for the Prediction of Premature Birth by means of Multichannel Electrohysterographic Records. Journal of Sensors, 2019, 13. doi:10.1155/2019/5373810 |
| 45 | Mehta-Lee, S. S., Palma, A., Bernstein, P. S., Lounsbury, D., & Schlecht, N. F. (2017). A Preconception Nomogram to Predict Preterm Delivery. Matern Child Health J, 21(1), 118-127. doi:10.1007/s10995-016-2100-3 |
| 46 | Menon R, Bhat G, Saade GR, Spratt H. Multivariate adaptive regression splines analysis to predict biomarkers of spontaneous preterm birth. Acta Obstet Gynecol Scand 2014;93:382-91. doi: https://doi.org/10.1111/aogs.12344. |
| 47 | Mirroshandel SA, Ghasemian F, Monji-Azad S. Applying data mining techniques for increasing implantation rate by selecting best sperms for intra-cytoplasmic sperm injection treatment. Computer Methods and Programs in Biomedicine 2016;137:215-29. doi: https://doi.org/10.1016/j.cmpb.2016.09.013. |
| 48 | Morales, D. A., Bengoetxea, E., Larrañaga, P., García, M., Franco, Y., Fresnada, M., & Merino, M. (2008). Bayesian classification for the selection of *in vitro* human embryos using morphological and clinical data. Comput Methods Programs Biomed, 90(2), 104-116. doi:10.1016/j.cmpb.2007.11.018 |
| 49 | Murtoniemi, K., Villa, P. M., Matomäki, J., Keikkala, E., Vuorela, P., Hämäläinen, E., . . . Laivuori, H. (2018). Prediction of pre-eclampsia and its subtypes in high-risk cohort: hyperglycosylated human chorionic gonadotropin in multivariate models. BMC Pregnancy Childbirth, 18(1), 279. doi:10.1186/s12884-018-1908-9 |
| 50 | Paydar, K., Kalhori, S. R. N., Akbarian, M., & Sheikhtaheri, A. (2017). A clinical decision support system for prediction of pregnancy outcome in pregnant women with systemic lupus erythematosus. International Journal of Medical Informatics, 97, 239-246. doi:10.1016/j.ijmedinf.2016.10.018 |
| 51 | Qiu H, Yu HY, Wang LY, et al. Electronic health record driven prediction for gestational diabetes mellitus in early pregnancy. Scientific Reports 2017;7. doi: https://doi.org/10.1038/s41598-017-16665-y. |
| 52 | Sadi-Ahmed N, Kacha B, Taleb H, Kedir-Talha M. Relevant features selection for automatic prediction of preterm deliveries from pregnancy electrohysterograhic (ehg) records. J Med Syst 2017;41:204. doi: https://doi.org/10.1007/s10916-017-0847-8. |
| 53 | Saleem S, Naqvi SS, Manzoor T, Saeed A, Rehman NU, Mirza J. A strategy for classification of "vaginal vs. Cesarean section" delivery: Bivariate empirical mode decomposition of cardiotocographic recordings. Frontiers in Physiology 2019;10. doi: https://doi.org/10.3389/fphys.2019.00246. |
| 54 | Shahbakhti, M., Beiramvand, M., Bavi, M. R., & Mohammadi Far, S. (2019). A New Efficient Algorithm for Prediction of Preterm Labor. Conf Proc IEEE Eng Med Biol Soc, 2019, 4669-4672. doi:10.1109/embc.2019.8857837 |
| 55 | Sims, C. J., Meyn, L., Caruana, R., Rao, R. B., Mitchell, T., & Krohn, M. (2000). Predicting cesarean delivery with decision tree models. Am J Obstet Gynecol, 183(5), 1198-1206. doi:10.1067/mob.2000.108891 |
| 56 | Sovio, U., & Smith, G. C. S. (2018). Blinded ultrasound fetal biometry at 36 weeks and risk of emergency Cesarean delivery in a prospective cohort study of low-risk nulliparous women. Ultrasound Obstet Gynecol, 52(1), 78-86. doi:10.1002/uog.17513 |
| 57 | Uyar A, Bener A, Ciray HN. Predictive modeling of implantation outcome in an *in vitro* fertilization setting: An application of machine learning methods. Med Decis Making 2015;35:714-25. doi: https://doi.org/10.1177/0272989x14535984. |
| 58 | VerMilyea, M., Hall, J. M. M., Diakiw, S. M., Johnston, A., Nguyen, T., Perugini, D., . . . Perugini, M. (2020). Development of an artificial intelligence-based assessment model for prediction of embryo viability using static images captured by optical light microscopy during IVF. Hum Reprod, 35(4), 770-784. doi:10.1093/humrep/deaa013 |
| 59 | Wald, M., Sparks, A., Sandlow, J., Van-Voorhis, B., Syrop, C. H., & Niederberger, C. S. (2005). Computational models for prediction of IVF/ICSI outcomes with surgically retrieved spermatozoa. Reprod Biomed Online, 11(3), 325-331. doi:10.1016/s1472-6483(10)60840-1 |
| 60 | Wang, C., Zhu, W., Wei, Y., Su, R., Feng, H., Lin, L., & Yang, H. (2016). The Predictive Effects of Early Pregnancy Lipid Profiles and Fasting Glucose on the Risk of Gestational Diabetes Mellitus Stratified by Body Mass Index. J Diabetes Res, 2016, 3013567. doi:10.1155/2016/3013567 |
| 61 | Xu, H., Wei, Y., Yang, R., Feng, G., Tang, W., Zhang, H., . . . Qiao, J. (2019). Prospective observational cohort study: Computational models for early prediction of ongoing pregnancy in fresh IVF/ICSI-ET protocols. Life Sci, 222, 221-227. doi:10.1016/j.lfs.2019.03.012 |
| 62 | Yang, T., Li, N., Qiao, C., & Liu, C. (2019). Development of a Novel Nomogram for Predicting Placenta Accreta in Patients With Scarred Uterus: A Retrospective Cohort Study. Front Med (Lausanne), 6, 289. doi:10.3389/fmed.2019.00289 |
| 63 | Agopian, A. J., Lupo, P. J., Tinker, S. C., Canfield, M. A., Mitchell, L. E., & Natl Birth Defects Prevention, S. (2012). Working towards a risk prediction model for neural tube defects. Birth Defects Research Part a-Clinical and Molecular Teratology, 94(3), 141-146. doi:10.1002/bdra.22883 |
| 64 | Almeida, S. T., Katz, L., Coutinho, I., & Amorim, M. M. R. (2017). Validation of fullPIERS model for prediction of adverse outcomes among women with severe pre-eclampsia. Int J Gynaecol Obstet, 138(2), 142-147. doi:10.1002/ijgo.12197 |
| 65 | Al-Rubaie, Z. T. A., Hudson, H. M., Jenkins, G., Mahmoud, I., Ray, J. G., Askie, L. M., & Lord, S. J. (2020). Prediction of pre-eclampsia in nulliparous women using routinely collected maternal characteristics: a model development and validation study. BMC Pregnancy Childbirth, 20(1), 23. doi:10.1186/s12884-019-2712-x |
| 66 | Chandrasekaran, S., Bastek, J. A., Turitz, A. L., & Durnwald, C. P. (2016). A prediction score to assess the risk of delivering a large for gestational age infant among obese women. J Matern Fetal Neonatal Med, 29(1), 22-26. doi:10.3109/14767058.2014.991709 |
| 67 | Chen, L., Luo, D., Yu, X., Jin, M., & Cai, W. (2018). Predicting stress urinary incontinence during pregnancy: combination of pelvic floor ultrasound parameters and clinical factors. Acta Obstet Gynecol Scand. doi:10.1111/aogs.13368 |
| 68 | Ciobanu, A., Rouvali, A., Syngelaki, A., Akolekar, R., & Nicolaides, K. H. (2019). Prediction of small for gestational age neonates: screening by maternal factors, fetal biometry, and biomarkers at 35-37 weeks' gestation. Am J Obstet Gynecol, 220(5), 486.e481-486.e411. doi:10.1016/j.ajog.2019.01.227 |
| 69 | Cömert, Z., Kocamaz, A. F., & Subha, V. (2018). Prognostic model based on image-based time-frequency features and genetic algorithm for fetal hypoxia assessment. Comput Biol Med, 99, 85-97. doi:10.1016/j.compbiomed.2018.06.003 |
| 70 | Coppedè, F., Grossi, E., Migheli, F., & Migliore, L. (2010). Polymorphisms in folate-metabolizing genes, chromosome damage, and risk of Down syndrome in Italian women: identification of key factors using artificial neural networks. BMC Med Genomics, 3, 42. doi:10.1186/1755-8794-3-42 |
| 71 | Cortet, M., Maucort-Boulch, D., Deneux-Tharaux, C., Dupont, C., Rudigoz, R. C., Roy, P., & Huissoud, C. (2015). Severity of post-partum hemorrhage after vaginal delivery is not predictable from clinical variables available at the time post-partum hemorrhage is diagnosed. J Obstet Gynaecol Res, 41(2), 199-206. doi:10.1111/jog.12528 |
| 72 | Crovetto, F., Figueras, F., Triunfo, S., Crispi, F., Rodriguez-Sureda, V., Dominguez, C., . . . Gratacós, E. (2015). First trimester screening for early and late preeclampsia based on maternal characteristics, biophysical parameters, and angiogenic factors. Prenat Diagn, 35(2), 183-191. doi:10.1002/pd.4519 |
| 73 | Eggebø, T. M., Wilhelm-Benartzi, C., Hassan, W. A., Usman, S., Salvesen, K. A., & Lees, C. C. (2015). A model to predict vaginal delivery in nulliparous women based on maternal characteristics and intrapartum ultrasound. Am J Obstet Gynecol, 213(3), 362.e361-366. doi:10.1016/j.ajog.2015.05.044 |
| 74 | Figueras, F., Savchev, S., Triunfo, S., Crovetto, F., & Gratacos, E. (2015). An integrated model with classification criteria to predict small-for-gestational-age fetuses at risk of adverse perinatal outcome. Ultrasound Obstet Gynecol, 45(3), 279-285. doi:10.1002/uog.14714 |
| 75 | Gao, C., Osmundson, S., Edwards, D. R. V., Jackson, G. P., Malin, B. A., & Chen, Y. (2019). Deep learning predicts extreme preterm birth from electronic health records. Journal of Biomedical Informatics, 100, 9. doi:10.1016/j.jbi.2019.103334 |
| 76 | Isakov, O., Reicher, L., Lavie, A., Yogev, Y., & Maslovitz, S. (2019). Prediction of Success in External Cephalic Version for Breech Presentation at Term. Obstet Gynecol, 133(5), 857-866. doi:10.1097/aog.0000000000003196 |
| 77 | Isono, W., Nagamatsu, T., Uemura, Y., Fujii, T., Hyodo, H., Yamashita, T., . . . Taketani, Y. (2011). Prediction model for the incidence of emergent cesarean section during induction of labor specialized in nulliparous low-risk women. J Obstet Gynaecol Res, 37(12), 1784-1791. doi:10.1111/j.1447-0756.2011.01607.x |
| 78 | Kang, X., Liang, Y., Wang, S., Hua, T., Cui, J., Zhang, M., . . . Xiao, J. (2019). Prediction model comparison for gestational diabetes mellitus with macrosomia based on risk factor investigation. J Matern Fetal Neonatal Med, 1-10. doi:10.1080/14767058.2019.1668922 |
| 79 | Khan, N., Ciobanu, A., Karampitsakos, T., Akolekar, R., & Nicolaides, K. H. (2019). Prediction of large-for-gestational-age neonate by routine third-trimester ultrasound. Ultrasound Obstet Gynecol, 54(3), 326-333. doi:10.1002/uog.20377 |
| 80 | Koivu, A., & Sairanen, M. (2020). Predicting risk of stillbirth and preterm pregnancies with machine learning. Health Inf Sci Syst, 8(1), 14. doi:10.1007/s13755-020-00105-9 |
| 81 | Kok, M., van der Steeg, J. W., van der Post, J. A., & Mol, B. W. (2011). Prediction of success of external cephalic version after 36 weeks. Am J Perinatol, 28(2), 103-110. doi:10.1055/s-0030-1262909 |
| 82 | Lee, J. S. E., Sultana, R., Han, N. L. R., Sia, A. T. H., & Sng, B. L. (2018). Development and validation of a predictive risk factor model for epidural re-siting in women undergoing labour epidural analgesia: a retrospective cohort study. BMC Anesthesiol, 18(1), 176. doi:10.1186/s12871-018-0638-x |
| 83 | Leonarduzzi R, Spilka J, Frecon J, et al. P-leader multifractal analysis and sparse svm for intrapartum fetal acidosis detection. Conf Proc IEEE Eng Med Biol Soc 2015;2015:1971-4. doi: https://doi.org/10.1109/embc.2015.7318771. |
| 84 | Li, H., Luo, M., Zheng, J., Luo, J., Zeng, R., Feng, N., . . . Fang, J. (2017). An artificial neural network prediction model of congenital heart disease based on risk factors: A hospital-based case-control study. Medicine (Baltimore), 96(6), e6090. doi:10.1097/md.0000000000006090 |
| 85 | McCowan, L. M., Thompson, J. M., Taylor, R. S., Baker, P. N., North, R. A., Poston, L., . . . Kenny, L. C. (2017). Prediction of Small for Gestational Age Infants in Healthy Nulliparous Women Using Clinical and Ultrasound Risk Factors Combined with Early Pregnancy Biomarkers. PLoS One, 12(1), e0169311. doi:10.1371/journal.pone.0169311 |
| 86 | Meijerink, A. M., Cissen, M., Mochtar, M. H., Fleischer, K., Thoonen, I., de Melker, A. A., . . . Ramos, L. (2016). Prediction model for live birth in ICSI using testicular extracted sperm. Hum Reprod, 31(9), 1942-1951. doi:10.1093/humrep/dew146 |
| 87 | Metz, T. D., Stoddard, G. J., Henry, E., Jackson, M., Holmgren, C., & Esplin, S. (2013). Simple, validated vaginal birth after cesarean delivery prediction model for use at the time of admission. Obstet Gynecol, 122(3), 571-578. doi:10.1097/AOG.0b013e31829f8ced |
| 88 | Milewski, R., Kuczyńska, A., Stankiewicz, B., & Kuczyński, W. (2017). How much information about embryo implantation potential is included in morphokinetic data? A prediction model based on artificial neural networks and principal component analysis. Adv Med Sci, 62(1), 202-206. doi:10.1016/j.advms.2017.02.001 |
| 89 | Myers, J. E., Kenny, L. C., McCowan, L. M., Chan, E. H., Dekker, G. A., Poston, L., . . . North, R. A. (2013). Angiogenic factors combined with clinical risk factors to predict preterm pre-eclampsia in nulliparous women: a predictive test accuracy study. Bjog, 120(10), 1215-1223. doi:10.1111/1471-0528.12195 |
| 90 | Payne, B. A., Groen, H., Ukah, U. V., Ansermino, J. M., Bhutta, Z., Grobman, W., . . . von Dadelszen, P. (2015). Development and internal validation of a multivariable model to predict perinatal death in pregnancy hypertension. Pregnancy Hypertens, 5(4), 315-321. doi:10.1016/j.preghy.2015.08.006 |
| 91 | Pettersson, K., Yousaf, K., Ranstam, J., Westgren, M., & Ajne, G. (2017). Predictive value of traction force measurement in vacuum extraction: Development of a multivariate prognostic model. PLoS One, 12(3), e0171938. doi:10.1371/journal.pone.0171938 |
| 92 | Qiu, J., Li, P., Dong, M., Xin, X., & Tan, J. (2019). Personalized prediction of live birth prior to the first *in vitro* fertilization treatment: a machine learning method. J Transl Med, 17(1), 317. doi:10.1186/s12967-019-2062-5 |
| 93 | Reid, S., Lu, C., & Condous, G. (2015). Can we improve the prediction of pouch of Douglas obliteration in women with suspected endometriosis using ultrasound-based models? A multicenter prospective observational study. Acta Obstet Gynecol Scand, 94(12), 1297-1306. doi:10.1111/aogs.12779 |
| 94 | Ryu, A., Cho, N. J., Kim, Y. S., & Lee, E. Y. (2019). Predictive value of serum uric acid levels for adverse perinatal outcomes in preeclampsia. Medicine (Baltimore), 98(18), e15462. doi:10.1097/md.0000000000015462 |
| 95 | Scheinhardt, M. O., Lerman, T., König, I. R., & Griesinger, G. (2018). Performance of prognostic modelling of high and low ovarian response to ovarian stimulation for IVF. Hum Reprod, 33(8), 1499-1505. doi:10.1093/humrep/dey236 |
| 96 | Signorini, M. G., Pini, N., Malovini, A., Bellazzi, R., & Magenes, G. (2020). Integrating machine learning techniques and physiology based heart rate features for antepartum fetal monitoring. Comput Methods Programs Biomed, 185, 105015. doi:10.1016/j.cmpb.2019.105015 |
| 97 | Spilka J, Frecon J, Leonarduzzi R, Pustelnik N, Abry P, Doret M. Intrapartum fetal heart rate classification from trajectory in sparse svm feature space. Conf Proc IEEE Eng Med Biol Soc 2015;2015:2335-8. doi: https://doi.org/10.1109/embc.2015.7318861. |
| 98 | Stamatopoulos, N., Lu, C., Casikar, I., Reid, S., Mongelli, M., Hardy, N., & Condous, G. (2015). Prediction of subsequent miscarriage risk in women who present with a viable pregnancy at the first early pregnancy scan. Aust N Z J Obstet Gynaecol, 55(5), 464-472. doi:10.1111/ajo.12395 |
| 99 | Stott, D., Bolten, M., Salman, M., Paraschiv, D., Douiri, A., & Kametas, N. A. (2017). A prediction model for the response to oral labetalol for the treatment of antenatal hypertension. J Hum Hypertens, 31(2), 126-131. doi:10.1038/jhh.2016.50 |
| 100 | Timmerman, E., Oude Rengerink, K., Pajkrt, E., Opmeer, B. C., van der Post, J. A., & Bilardo, C. M. (2010). Ductus venosus pulsatility index measurement reduces the false-positive rate in first-trimester screening. Ultrasound Obstet Gynecol, 36(6), 661-667. doi:10.1002/uog.7706 |
| 101 | Tsur, A., Batsry, L., Toussia-Cohen, S., Rosenstein, M. G., Barak, O., Brezinov, Y., . . . Aran, D. (2019). Development and validation of a machine learning model for prediction of shoulder dystocia. Ultrasound Obstet Gynecol. doi:10.1002/uog.21878 |
| 102 | Uyar A, Bener A, Ciray HN, Bahceci M. Roc based evaluation and comparison of classifiers for ivf implantation prediction. 2010;27 LNICST:108-11 |
| 103 | Van Calster, B., Condous, G., Kirk, E., Bourne, T., Timmerman, D., & Van Huffel, S. (2009). An application of methods for the probabilistic three-class classification of pregnancies of unknown location. Artif Intell Med, 46(2), 139-154. doi:10.1016/j.artmed.2008.12.003 |
| 104 | van der Ham, D. P., van Kuijk, S., Opmeer, B. C., Willekes, C., van Beek, J. J., Mulder, A. L., . . . Mol, B. W. (2014). Can neonatal sepsis be predicted in late preterm premature rupture of membranes? Development of a prediction model. Eur J Obstet Gynecol Reprod Biol, 176, 90-95. doi:10.1016/j.ejogrb.2014.02.003 |
| 105 | Verhoeven, C. J., Nuij, C., Janssen-Rolf, C. R., Schuit, E., Bais, J. M., Oei, S. G., & Mol, B. W. (2016). Predictors for failure of vacuum-assisted vaginal delivery: a case-control study. Eur J Obstet Gynecol Reprod Biol, 200, 29-34. doi:10.1016/j.ejogrb.2016.02.008 |
| 106 | Vieira, M. C., White, S. L., Patel, N., Seed, P. T., Briley, A. L., Sandall, J., . . . Pasupathy, D. (2017). Prediction of uncomplicated pregnancies in obese women: a prospective multicentre study. BMC Med, 15(1), 194. doi:10.1186/s12916-017-0956-8 |
| 107 | Visentin, S., Londero, A. P., Camerin, M., Grisan, E., & Cosmi, E. (2017). A possible new approach in the prediction of late gestational hypertension: The role of the fetal aortic intima-media thickness. Medicine (Baltimore), 96(2), e5515. doi:10.1097/md.0000000000005515 |
| 108 | Wang, Y., Moussavi, F., & Lorenzen, P. (2013). Automated embryo stage classification in time-lapse microscopy video of early human embryo development. Med Image Comput Comput Assist Interv, 16(Pt 2), 460-467. doi:10.1007/978-3-642-40763-5_57 |
| 109 | Yu, C. H., Zhang, R. P., Li, J., & A, Z. C. (2018). A predictive model for high-quality blastocyst based on blastomere number, fragmentation, and symmetry. J Assist Reprod Genet, 35(5), 809-816. doi:10.1007/s10815-018-1132-6 |
| 110 | Zwertbroek, E. F., Broekhuijsen, K., Langenveld, J., van Baaren, G. J., van den Berg, P. P., Bremer, H. A., . . . Franssen, M. T. (2017). Prediction of progression to severe disease in women with late preterm hypertensive disorders of pregnancy. Acta Obstet Gynecol Scand, 96(1), 96-105. doi:10.1111/aogs.13051 |
| 111 | Alberola-Rubio J, Garcia-Casado J, Prats-Boluda G, et al. Prediction of labor onset type: Spontaneous vs induced; role of electrohysterography? Comput Methods Programs Biomed 2017;144:127-33. doi: https://doi.org/10.1016/j.cmpb.2017.03.018. |
| 112 | Allouche, M., Huissoud, C., Guyard-Boileau, B., Rouzier, R., & Parant, O. (2011). Development and validation of nomograms for predicting preterm delivery. Am J Obstet Gynecol, 204(3), 242.e241-248. doi:10.1016/j.ajog.2010.09.030 |
| 113 | Benalcazar-Parra, C., Ye-Lin, Y. Y., Garcia-Casado, J., Monfort-Ortiz, R., Alberola-Rubio, J., Perales, A., & Prats-Boluda, G. (2019). Prediction of Labor Induction Success from the Uterine Electrohysterogram. Journal of Sensors, 2019, 12. doi:10.1155/2019/6916251 |
| 114 | Berntorp, K., Anderberg, E., Claesson, R., Ignell, C., & Källén, K. (2015). The relative importance of maternal body mass index and glucose levels for prediction of large-for-gestational-age births. BMC Pregnancy Childbirth, 15, 280. doi:10.1186/s12884-015-0722-x |
| 115 | Broekmans, F. J., Verweij, P. J. M., Eijkemans, M. J. C., Mannaerts, B., & Witjes, H. (2014). Prognostic models for high and low ovarian responses in controlled ovarian stimulation using a GnRH antagonist protocol. Human Reproduction, 29(8), 1688-1697. doi:10.1093/humrep/deu090 |
| 116 | Casikar, I., Lu, C., Reid, S., & Condous, G. (2013). Prediction of successful expectant management of first trimester miscarriage: development and validation of a new mathematical model. Aust N Z J Obstet Gynaecol, 53(1), 58-63. doi:10.1111/ajo.12053 |
| 117 | Cerqueira FR, Ferreira TG, de Paiva Oliveira A, et al. Nicesim: An open-source simulator based on machine learning techniques to support medical research on prenatal and perinatal care decision making. Artif Intell Med 2014;62:193-201. doi: https://doi.org/10.1016/j.artmed.2014.10.001. |
| 118 | Elaveyini, U., Devi, S. P., & Rao, K. S. (2011). Neural networks prediction of preterm delivery with first trimester bleeding. Arch Gynecol Obstet, 283(5), 971-979. doi:10.1007/s00404-010-1469-2 |
| 119 | Georgoulas, G., Karvelis, P., Gavrilis, D., Stylios, C. D., & Nikolakopoulos, G. (2017). An ordinal classification approach for CTG categorization. Conf Proc IEEE Eng Med Biol Soc, 2017, 2642-2645. doi:10.1109/embc.2017.8037400 |
| 120 | Harper, L. M., Glover, A. V., Biggio, J. R., & Tita, A. (2016). Predicting failure of glyburide therapy in gestational diabetes. J Perinatol, 36(5), 347-351. doi:10.1038/jp.2015.216 |
| 121 | Hernandez-Gonzalez J, Inza I, Crisol-Ortiz L, Guembe MA, Inarra MJ, Lozano JA. Fitting the data from embryo implantation prediction: Learning from label proportions. Statistical Methods in Medical Research 2018;27:1056-66. doi: https://doi.org/10.1177/0962280216651098. |
| 122 | Jhee, J. H., Lee, S., Park, Y., Lee, S. E., Kim, Y. A., Kang, S. W., . . . Park, J. T. (2019). Prediction model development of late-onset preeclampsia using machine learning-based methods. PLoS One, 14(8), e0221202. doi:10.1371/journal.pone.0221202 |
| 123 | Kawakita, T., Mokhtari, N., Huang, J. C., & Landy, H. J. (2019). Evaluation of Risk-Assessment Tools for Severe Postpartum Hemorrhage in Women Undergoing Cesarean Delivery. Obstet Gynecol, 134(6), 1308-1316. doi:10.1097/aog.0000000000003574 |
| 124 | Kuhle S, Maguire B, Zhang H, et al. Comparison of logistic regression with machine learning methods for the prediction of fetal growth abnormalities: A retrospective cohort study. BMC Pregnancy Childbirth 2018;18:333. doi: https://doi.org/10.1186/s12884-018-1971-2. |
| 125 | Kumar, S. N., Saxena, P., Patel, R., Sharma, A., Pradhan, D., Singh, H., . . . Jain, A. K. (2020). Predicting risk of low birth weight offspring from maternal features and blood polycyclic aromatic hydrocarbon concentration. Reprod Toxicol. doi:10.1016/j.reprotox.2020.03.009 |
| 126 | Lafalla, O., Esteban, L. M., Lou, A. C., Cornudella, R., Domínguez, M., Sanz, G., & Borque-Fernando, Á. (2019). Clinical utility of thrombophilia, anticoagulant treatment, and maternal variables as predictors of placenta-mediated pregnancy complications: an extensive analysis. J Matern Fetal Neonatal Med, 1-11. doi:10.1080/14767058.2019.1611764 |
| 127 | Liu, Z. H., Huang, B., Cui, Y. Q., Xu, Y. F., Zhang, B., Zhu, L. X., . . . Wu, D. R. (2019). Multi-Task Deep Learning With Dynamic Programming for Embryo Early Development Stage Classification From Time-Lapse Videos. IEEE Access, 7, 122153-122163. doi:10.1109/access.2019.2937765 |
| 128 | McCowan, L. M., Thompson, J. M., Taylor, R. S., North, R. A., Poston, L., Baker, P. N., . . . Kenny, L. C. (2013). Clinical prediction in early pregnancy of infants small for gestational age by customised birthweight centiles: findings from a healthy nulliparous cohort. PLoS One, 8(8), e70917. doi:10.1371/journal.pone.0070917 |
| 129 | Meister, M. R., Cahill, A. G., Conner, S. N., Woolfolk, C. L., & Lowder, J. L. (2016). Predicting obstetric anal sphincter injuries in a modern obstetric population. Am J Obstet Gynecol, 215(3), 310.e311-317. doi:10.1016/j.ajog.2016.02.041 |
| 130 | Mello, G., Parretti, E., Ognibene, A., Mecacci, F., Cioni, R., Scarselli, G., & Messeri, G. (2001). Prediction of the development of pregnancy-induced hypertensive disorders in high-risk pregnant women by artificial neural networks. Clin Chem Lab Med, 39(9), 801-805. doi:10.1515/cclm.2001.132 |
| 131 | Oates, J., Casikar, I., Campain, A., Müller, S., Yang, J., Reid, S., & Condous, G. (2013). A prediction model for viability at the end of the first trimester after a single early pregnancy evaluation. Aust N Z J Obstet Gynaecol, 53(1), 51-57. doi:10.1111/ajo.12046 |
| 132 | Petrozziello, A., Jordanov, I., Aris Papageorghiou, T., Christopher Redman, W. G., & Georgieva, A. (2018). Deep Learning for Continuous Electronic Fetal Monitoring in Labor. Conf Proc IEEE Eng Med Biol Soc, 2018, 5866-5869. doi:10.1109/embc.2018.8513625 |
| 133 | Petrozziello, A., Redman, C. W. G., Papageorghiou, A. T., Jordanov, I., & Georgieva, A. (2019). Multimodal Convolutional Neural Networks to Detect Fetal Compromise During Labor and Delivery. IEEE Access, 7, 112026-112036. doi:10.1109/access.2019.2933368 |
| 134 | Pettersson, G., Andersen, A. N., Broberg, P., & Arce, J. C. (2010). Pre-stimulation parameters predicting live birth after IVF in the long GnRH agonist protocol. Reprod Biomed Online, 20(5), 572-581. doi:10.1016/j.rbmo.2010.02.014 |
| 135 | Ramanah, R., Omar, S., Guillien, A., Pugin, A., Martin, A., Riethmuller, D., & Mottet, N. (2018). Predicting umbilical artery pH during labour: Development and validation of a nomogram using fetal heart rate patterns. Eur J Obstet Gynecol Reprod Biol, 225, 166-171. doi:10.1016/j.ejogrb.2018.04.008 |
| 136 | Spilka J, Frecon J, Leonarduzzi R, Pustelnik N, Abry P, Doret M. Sparse support vector machine for intrapartum fetal heart rate classification. IEEE J Biomed Health Inform 2017;21:664-71. doi: https://doi.org/10.1109/jbhi.2016.2546312. |
| 137 | Stroux, L., Redman, C. W., Georgieva, A., Payne, S. J., & Clifford, G. D. (2017). Doppler-based fetal heart rate analysis markers for the detection of early intrauterine growth restriction. Acta Obstet Gynecol Scand, 96(11), 1322-1329. doi:10.1111/aogs.13228 |
| 138 | Valensise, H., Facchinetti, F., Vasapollo, B., Giannini, F., Monte, I. D., & Arduini, D. (2006). The computerized fetal heart rate analysis in post-term pregnancy identifies patients at risk for fetal distress in labour. Eur J Obstet Gynecol Reprod Biol, 125(2), 185-192. doi:10.1016/j.ejogrb.2005.06.034 |
| 139 | Vogiatzi, P., Pouliakis, A., & Siristatidis, C. (2019). An artificial neural network for the prediction of assisted reproduction outcome. J Assist Reprod Genet, 36(7), 1441-1448. doi:10.1007/s10815-019-01498-7 |
| 140 | Xu L, Georgieva A, Redman CW, Payne SJ. Feature selection for computerized fetal heart rate analysis using genetic algorithms. Conf Proc IEEE Eng Med Biol Soc 2013;2013:445-8. doi: https://doi.org/10.1109/embc.2013.6609532. |
| 141 | Xu, H., Feng, G., Wei, Y., Feng, Y., Yang, R., Wang, L., . . . Qiao, J. (2020). Predicting Ectopic Pregnancy Using Human Chorionic Gonadotropin (hCG) Levels and Main Cause of Infertility in Women Undergoing Assisted Reproductive Treatment: Retrospective Observational Cohort Study. JMIR Med Inform, 8(4), e17366. doi:10.2196/17366 |
| 142 | Zhao, R. F., Zhang, W. Y., Zhou, L., & Chen, Y. (2019). Building a predictive model for successful vaginal delivery in nulliparas with term cephalic singleton pregnancies using decision tree analysis. J Obstet Gynaecol Res, 45(8), 1536-1544. doi:10.1111/jog.14011 |
| 143 | Abbas R, Hussain AJ, Al-Jumeily D, Baker T, Khattak A. Classification of foetal distress and hypoxia using machine learning approaches. 2018;10956 LNAI:767-76 |
| 144 | Abbas, Z., Saad, A., Ayache, M., & Fakih, C. (2019). Applications of Logistic Regression and Artificial Neural Network for ICSI Prediction. International Arab Journal of Information Technology, 16(3A), 557-564. |
| 145 | Acharya, U. R., Sudarshan, V. K., Rong, S. Q., Tan, Z., Lim, C. M., Koh, J. E., . . . Bhandary, S. V. (2017). Automated detection of premature delivery using empirical mode and wavelet packet decomposition techniques with uterine electromyogram signals. Comput Biol Med, 85, 33-42. doi:10.1016/j.compbiomed.2017.04.013 |
| 146 | Ahmadzia, H. K., Phillips, J. M., James, A. H., Rice, M. M., & Amdur, R. L. (2018). Predicting peripartum blood transfusion in women undergoing cesarean delivery: A risk prediction model. PLoS One, 13(12), e0208417. doi:10.1371/journal.pone.0208417 |
| 147 | Akbarian, M., Paydar, K., Kalhori, S. R. N., & Sheikhtaheri, A. (2015). Designing an artificial neural network for prediction of pregnancy outcomes in women with systemic lupus erythematosus in Iran. Tehran University Medical Journal, 73(4), 251-259. |
| 148 | Akbulut A, Ertugrul E, Topcu V. Fetal health status prediction based on maternal clinical history using machine learning techniques. Comput Methods Programs Biomed 2018;163:87-100. doi: https://doi.org/10.1016/j.cmpb.2018.06.010. |
| 149 | Alavifard, S., Meier, K., Shulman, Y., Tomlinson, G., & D'Souza, R. (2019). Derivation and validation of a model predicting the likelihood of vaginal birth following labour induction. BMC Pregnancy Childbirth, 19(1), 130. doi:10.1186/s12884-019-2232-8 |
| 150 | Alexander, P. M. A., DiOrio, M., Andren, K., Gauvreau, K., Mistry, K. P., Mathieu, D., . . . Bergersen, L. (2018). Accurate Prediction of Congenital Heart Surgical Length of Stay Incorporating a Procedure-Based Categorical Variable. Pediatr Crit Care Med, 19(10), 949-956. doi:10.1097/pcc.0000000000001668 |
| 151 | Ambalavanan, N., & Carlo, W. A. (2001). Comparison of the prediction of extremely low birth weight neonatal mortality by regression analysis and by neural networks. Early Hum Dev, 65(2), 123-137. doi:10.1016/s0378-3782(01)00228-6 |
| 152 | Arav-Boger, R., Boger, Y. S., Foster, C. B., & Boger, Z. (2008). The use of artificial neural networks in prediction of congenital CMV outcome from sequence data. Bioinform Biol Insights, 2, 281-289. doi:10.4137/bbi.s764 |
| 153 | Attallah, O., Sharkas, M. A., & Gadelkarim, H. (2019). Fetal Brain Abnormality Classification from MRI Images of Different Gestational Age. Brain Sci, 9(9). doi:10.3390/brainsci9090231 |
| 154 | Attallah, O., Sharkas, M. A., & Gadelkarim, H. (2020). Deep Learning Techniques for Automatic Detection of Embryonic Neurodevelopmental Disorders. Diagnostics, 10(1), 23. doi:10.3390/diagnostics10010027 |
| 155 | Aung, M. T., Yu, Y., Ferguson, K. K., Cantonwine, D. E., Zeng, L., McElrath, T. F., . . . Meeker, J. D. (2019). Prediction and associations of preterm birth and its subtypes with eicosanoid enzymatic pathways and inflammatory markers. Sci Rep, 9(1), 17049. doi:10.1038/s41598-019-53448-z |
| 156 | Austdal, M., Tangerås, L. H., Skråstad, R. B., Salvesen, K., Austgulen, R., Iversen, A. C., & Bathen, T. F. (2015). First Trimester Urine and Serum Metabolomics for Prediction of Preeclampsia and Gestational Hypertension: A Prospective Screening Study. Int J Mol Sci, 16(9), 21520-21538. doi:10.3390/ijms160921520 |
| 157 | Bahado-Singh, R. O., Yilmaz, A., Bisgin, H., Turkoglu, O., Kumar, P., Sherman, E., . . . Graham, S. F. (2019). Artificial intelligence and the analysis of multi-platform metabolomics data for the detection of intrauterine growth restriction. PLoS One, 14(4), e0214121. doi:10.1371/journal.pone.0214121 |
| 158 | Bahado-Singh, R. O., Vishweswaraiah, S., Aydas, B., Yilmaz, A., Saiyed, N. M., Mishra, N. K., . . . Radhakrishna, U. (2020). Precision cardiovascular medicine: artificial intelligence and epigenetics for the pathogenesis and prediction of coarctation in neonates. J Matern Fetal Neonatal Med, 1-8. doi:10.1080/14767058.2020.1722995 |
| 159 | Baykal, N., Reggia, J. A., Yalabik, N., Erkmen, A., & Beksac, M. S. (1994). Interpretation of Doppler blood flow velocity waveforms using neural networks. Proc Annu Symp Comput Appl Med Care, 865-869. |
| 160 | Betts KS, Kisely S, Alati R. Predicting common maternal postpartum complications: Leveraging health administrative data and machine learning. Bjog 2019;126:702-09. doi: https://doi.org/10.1111/1471-0528.15607. |
| 161 | Binenbaum, G., Ying, G. S., Quinn, G. E., Dreiseitl, S., Karp, K., Roberts, R. S., & Kirpalani, H. (2011). A clinical prediction model to stratify retinopathy of prematurity risk using postnatal weight gain. Pediatrics, 127(3), e607-614. doi:10.1542/peds.2010-2240 |
| 162 | Bottomley, C., Van Belle, V., Kirk, E., Van Huffel, S., Timmerman, D., & Bourne, T. (2013). Accurate prediction of pregnancy viability by means of a simple scoring system. Hum Reprod, 28(1), 68-76. doi:10.1093/humrep/des352 |
| 163 | Burai, P., Hajdu, A., Manuel, F. E., & Harangi, B. (2018). Segmentation of the uterine wall by an ensemble of fully convolutional neural networks. Conf Proc IEEE Eng Med Biol Soc, 2018, 49-52. doi:10.1109/embc.2018.8512245 |
| 164 | Cairo, S. B., Tabak, B. D., Berman, L., Berkelhamer, S. K., Yu, G., & Rothstein, D. H. (2018). Mortality after emergency abdominal operations in premature infants. J Pediatr Surg, 53(11), 2105-2111. doi:10.1016/j.jpedsurg.2018.01.009 |
| 165 | Catic, A., Gurbeta, L., Kurtovic-Kozaric, A., Mehmedbasic, S., & Badnjevic, A. (2018). Application of Neural Networks for classification of Patau, Edwards, Down, Turner and Klinefelter Syndrome based on first trimester maternal serum screening data, ultrasonographic findings and patient demographics. BMC Med Genomics, 11(1), 19. doi:10.1186/s12920-018-0333-2 |
| 166 | Catley, C., Frize, M., Walker, C. R., & Petriu, D. C. (2006). Predicting high-risk preterm birth using artificial neural networks. IEEE Trans Inf Technol Biomed, 10(3), 540-549. doi:10.1109/titb.2006.872069 |
| 167 | Chen, H., Ni, D., Qin, J., Li, S., Yang, X., Wang, T., & Heng, P. A. (2015). Standard Plane Localization in Fetal Ultrasound via Domain Transferred Deep Neural Networks. IEEE J Biomed Health Inform, 19(5), 1627-1636. doi:10.1109/jbhi.2015.2425041 |
| 168 | Comert Z, Kocamaz AF, Gungor S. Classification and comparison of cardiotocography signals with artificial neural network and extreme learning machine. Year:1493-96. doi: https://doi.org/10.1109/SIU.2016.7496034. |
| 169 | Czabanski, R., Jezewski, M., Wrobel, J., Jezewski, J., & Horoba, K. (2010). Predicting the risk of low-fetal birth weight from cardiotocographic signals using ANBLIR system with deterministic annealing and epsilon-insensitive learning. IEEE Trans Inf Technol Biomed, 14(4), 1062-1074. doi:10.1109/titb.2009.2039644 |
| 170 | De Ramón Fernández, A., Ruiz Fernández, D., & Prieto Sánchez, M. T. (2019). A decision support system for predicting the treatment of ectopic pregnancies. Int J Med Inform, 129, 198-204. doi:10.1016/j.ijmedinf.2019.06.002 |
| 171 | Devjak, R., Burnik Papler, T., Verdenik, I., Fon Tacer, K., & Vrtačnik Bokal, E. (2016). Embryo quality predictive models based on cumulus cells gene expression. Balkan J Med Genet, 19(1), 5-12. doi:10.1515/bjmg-2016-0001 |
| 172 | Dhillon, R. K., McLernon, D. J., Smith, P. P., Fishel, S., Dowell, K., Deeks, J. J., . . . Coomarasamy, A. (2016). Predicting the chance of live birth for women undergoing IVF: a novel pretreatment counselling tool. Hum Reprod, 31(1), 84-92. doi:10.1093/humrep/dev268 |
| 173 | Dida, N., Birhanu, Z., Gerbaba, M., Tilahun, D., & Morankar, S. (2014). Modeling the probability of giving birth at health institutions among pregnant women attending antenatal care in West Shewa Zone, Oromia, Ethiopia: a cross sectional study. Afr Health Sci, 14(2), 288-298. doi:10.4314/ahs.v14i2.3 |
| 174 | Dirvanauskas, D., Maskeliunas, R., Raudonis, V., & Damasevicius, R. (2019). Embryo development stage prediction algorithm for automated time lapse incubators. Comput Methods Programs Biomed, 177, 161-174. doi:10.1016/j.cmpb.2019.05.027 |
| 175 | Dithy, M. D., & Krishnapriya, V. (2019). Anemia selection in pregnant women by using random prediction (Rp) classification algorithm. International Journal of Recent Technology and Engineering, 8(2), 2623-2630. doi:10.35940/ijrte.B3016.078219 |
| 176 | Du, Y., Fang, Z., Jiao, B. D. J., Xi, G., Zhu, C., Ren, Y., . . . Wang, Y. (2020). Application of ultrasound-based radiomics technology in fetal lung texture analysis in pregnancies complicated by gestational diabetes or pre-eclampsia. Ultrasound Obstet Gynecol. doi:10.1002/uog.22037 |
| 177 | Dukhovny, D., Dukhovny, S., Pursley, D. M., Escobar, G. J., McCormick, M. C., Mao, W. Y., & Zupancic, J. A. (2012). The impact of maternal characteristics on the moderately premature infant: an antenatal maternal transport clinical prediction rule. J Perinatol, 32(7), 532-538. doi:10.1038/jp.2011.155 |
| 178 | Esty A, Frize M, Gilchrist J, Bariciak E. Applying data preprocessing methods to predict premature birth. Conf Proc IEEE Eng Med Biol Soc 2018;2018:6096-99. doi: https://doi.org/10.1109/embc.2018.8513681. |
| 179 | Feng, B., Hoskins, W., Zhang, Y., Meng, Z. B., Samuels, D. C., Wang, J. D., . . . Guo, Y. (2018). Bi-stream CNN Down Syndrome screening model based on genotyping array. Bmc Medical Genomics, 11, 9. doi:10.1186/s12920-018-0416-0 |
| 180 | Fries, J. A., Varma, P., Chen, V. S., Xiao, K., Tejeda, H., Saha, P., . . . Priest, J. R. (2019). Weakly supervised classification of aortic valve malformations using unlabeled cardiac MRI sequences. Nat Commun, 10(1), 3111. doi:10.1038/s41467-019-11012-3 |
| 181 | Frigerio, M., Manodoro, S., Bernasconi, D. P., Verri, D., Milani, R., & Vergani, P. (2018). Incidence and risk factors of third- and fourth-degree perineal tears in a single Italian scenario. Eur J Obstet Gynecol Reprod Biol, 221, 139-143. doi:10.1016/j.ejogrb.2017.12.042 |
| 182 | Galderisi, A., Zammataro, L., Losiouk, E., Lanzola, G., Kraemer, K., Facchinetti, A., . . . Steil, G. M. (2019). Continuous Glucose Monitoring Linked to an Artificial Intelligence Risk Index: Early Footprints of Intraventricular Hemorrhage in Preterm Neonates. Diabetes Technol Ther, 21(3), 146-153. doi:10.1089/dia.2018.0383 |
| 183 | Georgoulas, G., Karvelis, P., Spilka, J., Chudáček, V., Stylios, C. D., & Lhotská, L. (2017). Investigating pH based evaluation of fetal heart rate (FHR) recordings. Health Technol (Berl), 7(2), 241-254. doi:10.1007/s12553-017-0201-7 |
| 184 | Gioacchini, G., Notarstefano, V., Sereni, E., Zacà, C., Coticchio, G., Giorgini, E., . . . Borini, A. (2018). Does the molecular and metabolic profile of human granulosa cells correlate with oocyte fate? New insights by Fourier transform infrared microspectroscopy analysis. Mol Hum Reprod, 24(11), 521-532. doi:10.1093/molehr/gay035 |
| 185 | Goodson, S. G., White, S., Stevans, A. M., Bhat, S., Kao, C. Y., Jaworski, S., . . . O'Brien, D. A. (2017). CASAnova: a multiclass support vector machine model for the classification of human sperm motility patterns. Biol Reprod, 97(5), 698-708. doi:10.1093/biolre/iox120 |
| 186 | Hamilton, E. F., Dyachenko, A., Ciampi, A., Maurel, K., Warrick, P. A., & Garite, T. J. (2020). Estimating risk of severe neonatal morbidity in preterm births under 32 weeks of gestation. J Matern Fetal Neonatal Med, 33(1), 73-80. doi:10.1080/14767058.2018.1487395 |
| 187 | Hassan MR, Al-Insaif S, Hossain MI, Kamruzzaman J. A machine learning approach for prediction of pregnancy outcome following ivf treatment. Neural Computing and Applications 2018. doi: https://doi.org/10.1007/s00521-018-3693-9. |
| 188 | He, L., Zhou, W., Zhao, X., Liu, X., Rong, X., & Song, Y. (2019). Development and validation of a novel scoring system to predict severe intraventricular hemorrhage in very low birth weight infants. Brain Dev, 41(8), 671-677. doi:10.1016/j.braindev.2019.04.013 |
| 189 | Heidari, R., Akbariqomi, M., Motevaseli, E., Omrani, M. D., Kooshki, H., Shamshiri, A. R., . . . Tavoosidana, G. (2018). Performance and Predictive Value of First Trimester Screening Markers for Down Syndrome in Iranian Pregnancies. J Family Reprod Health, 12(3), 121-128. |
| 190 | Hu, J., Chen, Y., Zhong, J., Ju, R., & Yi, Z. (2019). Automated Analysis for Retinopathy of Prematurity by Deep Neural Networks. IEEE Trans Med Imaging, 38(1), 269-279. doi:10.1109/tmi.2018.2863562 |
| 191 | Inbarani, H. H., Banu, P. K. N., & Azar, A. T. (2014). Feature selection using swarm-based relative reduct technique for fetal heart rate. Neural Computing & Applications, 25(3-4), 793-806. doi:10.1007/s00521-014-1552-x |
| 192 | Iraji, M. S. (2019). Prediction of fetal state from the cardiotocogram recordings using neural network models. Artif Intell Med, 96, 33-44. doi:10.1016/j.artmed.2019.03.005 |
| 193 | Jadhav S, Nalbalwar S, Ghatol A. Modular neural network model based foetal state classification. Year:915-17. doi: https://doi.org/10.1109/BIBMW.2011.6112501. |
| 194 | Jalali, A., Licht, D. J., & Nataraj, C. (2013). Discovering hidden relationships in physiological signals for prediction of Periventricular Leukomalacia. Conf Proc IEEE Eng Med Biol Soc, 2013, 7080-7083. doi:10.1109/embc.2013.6611189 |
| 195 | Jang, J., Park, Y., Kim, B., Lee, S. M., Kwon, J. Y., & Seo, J. K. (2018). Automatic Estimation of Fetal Abdominal Circumference From Ultrasound Images. IEEE J Biomed Health Inform, 22(5), 1512-1520. doi:10.1109/jbhi.2017.2776116 |
| 196 | Kalafat, E., Morales-Rosello, J., Thilaganathan, B., Dhother, J., & Khalil, A. (2019). Risk of neonatal care unit admission in small for gestational age fetuses at term: a prediction model and internal validation. J Matern Fetal Neonatal Med, 32(14), 2361-2368. doi:10.1080/14767058.2018.1437412 |
| 197 | Kanakasabapathy, M. K., Thirumalaraju, P., Bormann, C. L., Kandula, H., Dimitriadis, I., Souter, I., . . . Shafiee, H. (2019). Development and evaluation of inexpensive automated deep learning-based imaging systems for embryology. Lab Chip, 19(24), 4139-4145. doi:10.1039/c9lc00721k |
| 198 | Kang, J., Kim, H. S., Lee, E. B., Uh, Y., Han, K. H., Park, E. Y., . . . Choi, S. J. (2020). Prediction Model for Massive Transfusion in Placenta Previa during Cesarean Section. Yonsei Med J, 61(2), 154-160. doi:10.3349/ymj.2020.61.2.154 |
| 199 | Kaur, P., Singh, G., & Kaur, P. (2019). An intelligent validation system for diagnostic and prognosis of ultrasound fetal growth analysis using Neuro-Fuzzy based on genetic algorithm. Egyptian Informatics Journal, 20(1), 55-87. doi:10.1016/j.eij.2018.10.002 |
| 200 | Kayode, G. A., Grobbee, D. E., Amoakoh-Coleman, M., Adeleke, I. T., Ansah, E., de Groot, J. A., & Klipstein-Grobusch, K. (2016). Predicting stillbirth in a low resource setting. BMC Pregnancy Childbirth, 16, 274. doi:10.1186/s12884-016-1061-2 |
| 201 | Khatibi, T., Kheyrikoochaksarayee, N., & Sepehri, M. M. (2019). Analysis of big data for prediction of provider-initiated preterm birth and spontaneous premature deliveries and ranking the predictive features. Arch Gynecol Obstet, 300(6), 1565-1582. doi:10.1007/s00404-019-05325-3 |
| 202 | Khosravi, P., Kazemi, E., Zhan, Q., Malmsten, J. E., Toschi, M., Zisimopoulos, P., . . . Hajirasouliha, I. (2019). Deep learning enables robust assessment and selection of human blastocysts after *in vitro* fertilization. NPJ Digit Med, 2, 21. doi:10.1038/s41746-019-0096-y |
| 203 | Kim, B., Kim, K. C., Park, Y., Kwon, J. Y., Jang, J., & Seo, J. K. (2018). Machine-learning-based automatic identification of fetal abdominal circumference from ultrasound images. Physiol Meas, 39(10), 105007. doi:10.1088/1361-6579/aae255 |
| 204 | Koivu A, Korpimäki T, Kivelä P, Pahikkala T, Sairanen M. Evaluation of machine learning algorithms for improved risk assessment for down's syndrome. Computers in Biology and Medicine 2018;98:1-7. doi: https://doi.org/10.1016/j.compbiomed.2018.05.004. |
| 205 | Krupa, N., Ali, M., Zahedi, E., Ahmed, S., & Hassan, F. M. (2011). Antepartum fetal heart rate feature extraction and classification using empirical mode decomposition and support vector machine. Biomed Eng Online, 10, 6. doi:10.1186/1475-925x-10-6 |
| 206 | Lafuente-Ganuza, P., Lequerica-Fernandez, P., Carretero, F., Escudero, A. I., Martinez-Morillo, E., Sabria, E., . . . Alvarez, F. V. (2020). A more accurate prediction to rule in and rule out pre-eclampsia using the sFlt-1/PlGF ratio and NT-proBNP as biomarkers. Clin Chem Lab Med, 58(3), 399-407. doi:10.1515/cclm-2019-0939 |
| 207 | Li, J. Q., Chen, Z. Z., Huang, L. X., Fang, M., Li, B., Fu, X. H., . . . Zhao, Q. G. (2019). Automatic Classification of Fetal Heart Rate Based on Convolutional Neural Network. Ieee Internet of Things Journal, 6(2), 1394-1401. doi:10.1109/jiot.2018.2845128 |
| 208 | Li, L., Liu, W. Y., Zhang, H. G., Jiang, Y. T., Hu, X. N., & Liu, R. Z. (2019). Down Syndrome Prediction Using a Cascaded Machine Learning Framework Designed for Imbalanced and Feature-correlated Data. IEEE Access, 7, 97582-97593. doi:10.1109/access.2019.2929681 |
| 209 | Liu, B., Shi, S., Wu, Y., Thomas, D., Symul, L., Pierson, E., & Leskovec, J. (2019). Predicting pregnancy using large-scale data from a women's health tracking mobile application. Proc Int World Wide Web Conf, 2019, 2999-3005. doi:10.1145/3308558.3313512 |
| 210 | Luo Y, Li Z, Guo H, et al. Predicting congenital heart defects: A comparison of three data mining methods. PLoS One 2017;12:e0177811. doi: https://doi.org/10.1371/journal.pone.0177811. |
| 211 | MacDowell, M., Somoza, E., Rothe, K., Fry, R., Brady, K., & Bocklet, A. (2001). Understanding birthing mode decision making using artificial neural networks. Med Decis Making, 21(6), 433-443. doi:10.1177/0272989x0102100601 |
| 212 | Macones, G. A., Chang, J. J., Stamilio, D. M., Odibo, A. O., Wang, J., & Cahill, A. G. (2013). Prediction of cesarean delivery using the fetal-pelvic index. Am J Obstet Gynecol, 209(5), 431.e431-438. doi:10.1016/j.ajog.2013.06.026 |
| 213 | Magenes, G., Pedrinazzi, L., & Signorini, M. G. (2004). Identification of fetal sufferance antepartum through a multiparametric analysis and a support vector machine. Conf Proc IEEE Eng Med Biol Soc, 2006, 462-465. doi:10.1109/iembs.2004.1403194 |
| 214 | Malacova, E., Tippaya, S., Bailey, H. D., Chai, K., Farrant, B. M., Gebremedhin, A. T., . . . Pereira, G. (2020). Stillbirth risk prediction using machine learning for a large cohort of births from Western Australia, 1980-2015. Sci Rep, 10(1), 5354. doi:10.1038/s41598-020-62210-9 |
| 215 | Milewski, R., Kuć, P., Kuczyńska, A., Stankiewicz, B., Łukaszuk, K., & Kuczyński, W. (2015). A predictive model for blastocyst formation based on morphokinetic parameters in time-lapse monitoring of embryo development. J Assist Reprod Genet, 32(4), 571-579. doi:10.1007/s10815-015-0440-3 |
| 216 | Moreira, M. W. L., Rodrigues, J., Carvalho, F. H. C., Chilamkurti, N., Al-Muhtadi, J., & Denisov, V. (2019). Biomedical data analytics in mobile-health environments for high-risk pregnancy outcome prediction. Journal of Ambient Intelligence and Humanized Computing, 10(10), 4121-4134. doi:10.1007/s12652-019-01230-4 |
| 217 | Natarajan S, Prabhakar A, Ramanan N, Bagilone A, Siek K, Connelly K. Boosting for postpartum depression prediction. Year:232-40. doi: https://doi.org/10.1109/CHASE.2017.82. |
| 218 | Ng, T. W., Xi, Y., Schindel, D., Beavers, A., Santiago-Munoz, P., Bailey, A. A., & Twickler, D. M. (2019). Fetal Head and Neck Masses: MRI Prediction of Significant Morbidity. AJR Am J Roentgenol, 212(1), 215-221. doi:10.2214/ajr.18.19753 |
| 219 | Ocak, H., & Ertunc, H. M. (2013). Prediction of fetal state from the cardiotocogram recordings using adaptive neuro-fuzzy inference systems. Neural Computing & Applications, 23(6), 1583-1589. doi:10.1007/s00521-012-1110-3 |
| 220 | Ochab, M., & Wajs, W. (2016). Expert system supporting an early prediction of the bronchopulmonary dysplasia. Comput Biol Med, 69, 236-244. doi:10.1016/j.compbiomed.2015.08.016 |
| 221 | Pan I, Nolan LB, Brown RR, et al. Machine learning for social services: A study of prenatal case management in illinois. Am J Public Health 2017;107:938-44. doi: https://doi.org/10.2105/ajph.2017.303711. |
| 222 | Paternina-Caicedo, A., Miranda, J., Bourjeily, G., Levinson, A., Dueñas, C., Bello-Muñoz, C., & Rojas-Suarez, J. A. (2017). Performance of the Obstetric Early Warning Score in critically ill patients for the prediction of maternal death. Am J Obstet Gynecol, 216(1), 58.e51-58.e58. doi:10.1016/j.ajog.2016.09.103 |
| 223 | Payne, B. A., Ryan, H., Bone, J., Magee, L. A., Aarvold, A. B., Mark Ansermino, J., . . . von Dadelszen, P. (2018). Development and internal validation of the multivariable CIPHER (Collaborative Integrated Pregnancy High-dependency Estimate of Risk) clinical risk prediction model. Crit Care, 22(1), 278. doi:10.1186/s13054-018-2215-6 |
| 224 | Rawashdeh, H., Awawdeh, S., Shannag, F., Henawi, E., Faris, H., Obeid, N., & Hyett, J. (2020). Intelligent system based on data mining techniques for prediction of preterm birth for women with cervical cerclage. Comput Biol Chem, 85, 107233. doi:10.1016/j.compbiolchem.2020.107233 |
| 225 | Ricard, C. A., Dammann, C. E. L., & Dammann, O. (2017). Screening Tool for Early Postnatal Prediction of Retinopathy of Prematurity in Preterm Newborns (STEP-ROP). Neonatology, 112(2), 130-136. doi:10.1159/000464459 |
| 226 | Rossi, R. M., Requarth, E. W., Warshak, C. R., Dufendach, K., Hall, E. S., & DeFranco, E. A. (2019). Predictive Model for Failed Induction of Labor Among Obese Women. Obstet Gynecol, 134(3), 485-493. doi:10.1097/aog.0000000000003377 |
| 227 | Sahin H, Subasi A. Classification of the cardiotocogram data for anticipation of fetal risks using machine learning techniques. Applied Soft Computing Journal 2015;33:231-38. doi: https://doi.org/10.1016/j.asoc.2015.04.038. |
| 228 | Sahli, H., Mouelhi, A., Ben Slama, A., Sayadi, M., & Rachdi, R. (2019). Supervised classification approach of biometric measures for automatic fetal defect screening in head ultrasound images. J Med Eng Technol, 43(5), 279-286. doi:10.1080/03091902.2019.1653389 |
| 229 | Samanta, B., Bird, G. L., Kuijpers, M., Zimmerman, R. A., Jarvik, G. P., Wernovsky, G., . . . Nataraj, C. (2009). Prediction of periventricular leukomalacia. Part I: Selection of hemodynamic features using logistic regression and decision tree algorithms. Artif Intell Med, 46(3), 201-215. doi:10.1016/j.artmed.2008.12.005 |
| 230 | Samanta, B., Bird, G. L., Kuijpers, M., Zimmerman, R. A., Jarvik, G. P., Wernovsky, G., . . . Nataraj, C. (2009). Prediction of periventricular leukomalacia. Part II: Selection of hemodynamic features using computational intelligence. Artif Intell Med, 46(3), 217-231. doi:10.1016/j.artmed.2008.12.004 |
| 231 | Shah SAA, Aziz W, Arif M, Nadeem MSA. Decision trees based classification of cardiotocograms using bagging approach. Year:12-17. doi: https://doi.org/10.1109/FIT.2015.14. |
| 232 | Shigemi, D., Yamaguchi, S., Aso, S., & Yasunaga, H. (2019). Predictive model for macrosomia using maternal parameters without sonography information. J Matern Fetal Neonatal Med, 32(22), 3859-3863. doi:10.1080/14767058.2018.1484090 |
| 233 | Sievert, R. A., Kuper, S. G., Jauk, V. C., Parrish, M., Biggio, J. R., & Harper, L. M. (2017). Predictors of vaginal delivery in medically indicated early preterm induction of labor. Am J Obstet Gynecol, 217(3), 375.e371-375.e377. doi:10.1016/j.ajog.2017.05.025 |
| 234 | Simpson, N. B., Shankar-Hari, M., Rowan, K. M., Cecconi, M., Von Dadelszen, P., Huning, E. Y. S., . . . Harrison, D. A. (2020). Maternal Risk Modeling in Critical Care-Development of a Multivariable Risk Prediction Model for Death and Prolonged Intensive Care∗. Crit Care Med, 663-672. doi:10.1097/CCM.0000000000004223 |
| 235 | Siriwardhana, C., Fang, R., Salanti, A., Leke, R. G. F., Bobbili, N., Taylor, D. W., & Chen, J. J. (2017). Statistical prediction of immunity to placental malaria based on multi-assay antibody data for malarial antigens. Malar J, 16(1), 391. doi:10.1186/s12936-017-2041-3 |
| 236 | Srivastava Y, Khanna P, Kumar S. Estimation of gestational diabetes mellitus using azure ai services. 2019 Amity International Conference on Artificial Intelligence (AICAI) Year:321-26. doi: https://doi.org/10.1109/AICAI.2019.8701307. |
| 237 | Street, M. E., Grossi, E., Volta, C., Faleschini, E., & Bernasconi, S. (2008). Placental determinants of fetal growth: identification of key factors in the insulin-like growth factor and cytokine systems using artificial neural networks. BMC Pediatr, 8, 24. doi:10.1186/1471-2431-8-24 |
| 238 | Sullivan, B. A., Wallman-Stokes, A., Isler, J., Sahni, R., Moorman, J. R., Fairchild, K. D., & Lake, D. E. (2018). Early Pulse Oximetry Data Improves Prediction of Death and Adverse Outcomes in a Two-Center Cohort of Very Low Birth Weight Infants. Am J Perinatol, 35(13), 1331-1338. doi:10.1055/s-0038-1654712 |
| 239 | Sultan, A. A., West, J., Grainge, M. J., Riley, R. D., Tata, L. J., Stephansson, O., . . . Ludvigsson, J. F. (2016). Development and validation of risk prediction model for venous thromboembolism in postpartum women: multinational cohort study. Bmj, 355, i6253. doi:10.1136/bmj.i6253 |
| 240 | Sun, H., Qu, H., Chen, L., Wang, W., Liao, Y., Zou, L., . . . Zhou, S. (2019). Identification of suspicious invasive placentation based on clinical MRI data using textural features and automated machine learning. Eur Radiol, 29(11), 6152-6162. doi:10.1007/s00330-019-06372-9 |
| 241 | Tabrizi, P. R., Obeid, R., Mansoor, A., Ensel, S., Cerrolaza, J. J., Penn, A., & Linguraru, M. G. (2017). Cranial ultrasound-based prediction of post hemorrhagic hydrocephalus outcome in premature neonates with intraventricular hemorrhage. Conf Proc IEEE Eng Med Biol Soc, 2017, 169-172. doi:10.1109/embc.2017.8036789 |
| 242 | Tang H, Wang T, Li M, Yang X. The design and implementation of cardiotocography signals classification algorithm based on neural network. Comput Math Methods Med 2018;2018:8568617. doi: https://doi.org/10.1155/2018/8568617. |
| 243 | Tang, S., & Chen, S. P. (2009). A fast automatic recognition and location algorithm for fetal genital organs in ultrasound images. J Zhejiang Univ Sci B, 10(9), 648-658. doi:10.1631/jzus.B0930162 |
| 244 | Tejera, E., Jose Areias, M., Rodrigues, A., Ramõa, A., Manuel Nieto-Villar, J., & Rebelo, I. (2011). Artificial neural network for normal, hypertensive, and preeclamptic pregnancy classification using maternal heart rate variability indexes. J Matern Fetal Neonatal Med, 24(9), 1147-1151. doi:10.3109/14767058.2010.545916 |
| 245 | Vickram, A. S., Kamini, A. R., Das, R., Pathy, M. R., Parameswari, R., Archana, K., & Sridharan, T. B. (2016). Validation of artificial neural network models for predicting biochemical markers associated with male infertility. Systems Biology in Reproductive Medicine, 62(4), 258-265. doi:10.1080/19396368.2016.1185654 |
| 246 | Vijayalakshmi, C., Sakthivel, P., & Vinekar, A. (2020). Automated Detection and Classification of Telemedical Retinopathy of Prematurity Images. Telemedicine and e-Health, 26(3), 354-358. doi:10.1089/tmj.2019.0004 |
| 247 | Wang, L., Matsunaga, S., Mikami, Y., Takai, Y., Terui, K., & Seki, H. (2016). Pre-delivery fibrinogen predicts adverse maternal or neonatal outcomes in patients with placental abruption. J Obstet Gynaecol Res, 42(7), 796-802. doi:10.1111/jog.12988 |
| 248 | Westerhuis, M. E., Schuit, E., Kwee, A., Zuithoff, N. P., Groenwold, R. H., Van Den Akker, E. S., . . . Moons, K. G. (2012). Prediction of neonatal metabolic acidosis in women with a singleton term pregnancy in cephalic presentation. Am J Perinatol, 29(3), 167-174. doi:10.1055/s-0031-1284226 |
| 249 | Wilson, K., Hawken, S., Potter, B. K., Chakraborty, P., Walker, M., Ducharme, R., & Little, J. (2016). Accurate prediction of gestational age using newborn screening analyte data. Am J Obstet Gynecol, 214(4), 513.e511-513.e519. doi:10.1016/j.ajog.2015.10.017 |
| 250 | Xie, H., Wang, N., He, M., Zhang, L., Cai, H., Xian, J., . . . Yang, Y. (2020). Using deep learning algorithms to classify fetal brain ultrasound images as normal or abnormal. Ultrasound Obstet Gynecol. doi:10.1002/uog.21967 |
| 251 | Yang J, Ding X, Zhu W. Improving the calling of non-invasive prenatal testing on 13-/18-/21-trisomy by support vector machine discrimination. PLoS One 2018;13:e0207840. doi: https://doi.org/10.1371/journal.pone.0207840. |
| 252 | Zernikow, B., Holtmannspoetter, K., Michel, E., Pielemeier, W., Hornschuh, F., Westermann, A., & Hennecke, K. H. (1998). Artificial neural network for risk assessment in preterm neonates. Arch Dis Child Fetal Neonatal Ed, 79(2), F129-134. doi:10.1136/fn.79.2.f129 |
| 253 | Zhang, L., Wang, Y., Han, J., Shen, H., Zhao, M., & Cai, S. (2018). Neutrophil-lymphocyte ratio, gamma-glutamyl transpeptidase, lipase, high-density lipoprotein as a panel of factors to predict acute pancreatitis in pregnancy. Medicine (Baltimore), 97(26), e11189. doi:10.1097/md.0000000000011189 |
| 254 | Zhang, B., Cui, Y., Wang, M., Li, J., Jin, L., & Wu, D. (2019). *In vitro* Fertilization (IVF) Cumulative Pregnancy Rate Prediction from Basic Patient Characteristics. IEEE Access, 7, 130460-130467. doi:10.1109/ACCESS.2019.2940588 |
| 255 | Zhang, W., Liu, H., Silenzio, V. M. B., Qiu, P., & Gong, W. (2020). Machine Learning Models for the Prediction of Postpartum Depression: Application and Comparison Based on a Cohort Study. JMIR Med Inform, 8(4), e15516. doi:10.2196/15516 |
| 256 | Zhu, W., Chen, X., Wang, Y., & Wang, L. (2019). Arrhythmia Recognition and Classification Using ECG Morphology and Segment Feature Analysis. IEEE/ACM Transactions on Computational Biology and Bioinformatics, 16(1), 131-138. doi:10.1109/TCBB.2018.2846611 |
| 257 | Borowska, M., Brzozowska, E., Kuć, P., Oczeretko, E., Mosdorf, R., & Laudański, P. (2018). Identification of preterm birth based on RQA analysis of electrohysterograms. Comput Methods Programs Biomed, 153, 227-236. doi:10.1016/j.cmpb.2017.10.018 |
| 258 | Cannas, M., & Arpino, B. (2019). A comparison of machine learning algorithms and covariate balance measures for propensity score matching and weighting. Biom J, 61(4), 1049-1072. doi:10.1002/bimj.201800132 |
| 259 | Dash, S., Quirk, J. G., & Djurić, P. M. (2014). Fetal heart rate classification using generative models. IEEE Trans Biomed Eng, 61(11), 2796-2805. doi:10.1109/tbme.2014.2330556 |
| 260 | Fang, H., Johnson, C., Stopp, C., & Espy, K. A. (2011). A new look at quantifying tobacco exposure during pregnancy using fuzzy clustering. Neurotoxicol Teratol, 33(1), 155-165. doi:10.1016/j.ntt.2010.08.003 |
| 261 | Frick, A., Kostiv, V., Vojtassakova, D., Akolekar, R., & Nicolaides, K. H. (2020). Comparison of different methods of measuring angle of progression in prediction of labor outcome. Ultrasound Obstet Gynecol, 55(3), 391-400. doi:10.1002/uog.21913 |
| 262 | Hutton, E. K., Simioni, J. C., & Thabane, L. (2017). Predictors of success of external cephalic version and cephalic presentation at birth among 1253 women with non-cephalic presentation using logistic regression and classification tree analyses. Acta Obstet Gynecol Scand, 96(8), 1012-1020. doi:10.1111/aogs.13161 |
| 263 | Karvelis, P., Spilka, J., Georgoulas, G., Chudáček, V., Stylios, C. D., & Lhotská, L. (2015). Combining latent class analysis labeling with multiclass approach for fetal heart rate categorization. Physiol Meas, 36(5), 1001-1024. doi:10.1088/0967-3334/36/5/1001 |
| 264 | Kragh, M. F., Rimestad, J., Berntsen, J., & Karstoft, H. (2019). Automatic grading of human blastocysts from time-lapse imaging. Comput Biol Med, 115, 103494. doi:10.1016/j.compbiomed.2019.103494 |
| 265 | La Rosa, P. S., Nehorai, A., Eswaran, H., Lowery, C. L., & Preissl, H. (2008). Detection of uterine MMG contractions using a multiple change point estimator and the K-means cluster algorithm. IEEE Trans Biomed Eng, 55(2 Pt 1), 453-467. doi:10.1109/tbme.2007.912663 |
| 266 | Moslem, B., Diab, M. O., Marque, C., & Khalil, M. (2011). Classification of multichannel uterine EMG signals. Conf Proc IEEE Eng Med Biol Soc, 2011, 2602-2605. doi:10.1109/iembs.2011.6090718 |
| 267 | Soslow, J. H., Kavanaugh-McHugh, A., Wang, L., Saurers, D. L., Kaushik, N., Killen, S. A., & Parra, D. A. (2013). A clinical prediction model to estimate the risk for coarctation of the aorta in the presence of a patent ductus arteriosus. J Am Soc Echocardiogr, 26(12), 1379-1387. doi:10.1016/j.echo.2013.08.016 |
| 268 | Teder, H., Paluoja, P., Rekker, K., Salumets, A., Krjutskov, K., & Palta, P. (2019). Computational framework for targeted high coverage sequencing based NIPT Computational framework for targeted high coverage sequencing based NIPT. PLoS One, 14(7), 19. doi:10.1371/journal.pone.0209139 |
| 269 | Warrick, P. A., Hamilton, E. F., Precup, D., & Kearney, R. E. (2010). Classification of normal and hypoxic fetuses from systems modeling of intrapartum cardiotocography. IEEE Trans Biomed Eng, 57(4), 771-779. doi:10.1109/tbme.2009.2035818 |
| 270 | Zhang, Z., & Han, Y. (2020). Detection of Ovarian Tumors in Obstetric Ultrasound Imaging Using Logistic Regression Classifier with an Advanced Machine Learning Approach. IEEE Access, 8, 44999-45008. doi:10.1109/ACCESS.2020.2977962 |
| 271 | Chamidah N, Wasito I. Fetal state classification from cardiotocography based on feature extraction using hybrid k-means and support vector machine. Year:37-41. doi: https://doi.org/10.1109/ICACSIS.2015.7415166. |
| 272 | Goodale BM, Shilaih M, Falco L, Dammeier F, Hamvas G, Leeners B. Wearable sensors reveal menses-driven changes in physiology and enable prediction of the fertile window: Observational study. Journal of Medical Internet Research 2019;21. doi: https://doi.org/10.2196/13404. |
| 273 | Gorthi A, Firtion C, Vepa J. Automated risk assessment tool for pregnancy care. Conference proceedings : Annual International Conference of the IEEE Engineering in Medicine and Biology Society IEEE Engineering in Medicine and Biology Society Conference 2009:6222-25. doi, PMID. |
| 274 | Ocak H. A, medical decision support system based on support vector machines and the genetic algorithm for the evaluation of fetal well-being. J Med Syst 2013;37:9913. doi: https://doi.org/10.1007/s10916-012-9913-4. |
| 275 | Ravindran S, Jambek AB, Muthusamy H, Neoh SC. A novel clinical decision support system using improved adaptive genetic algorithm for the assessment of fetal well-being. Comput Math Methods Med 2015;2015:283532. doi: https://doi.org/10.1155/2015/283532. |
| 276 | Abuelghar, W. M., Ellaithy, M. I., Swidan, K. H., Allam, I. S., & Haggag, H. M. (2019). Prediction of spontaneous preterm birth: salivary progesterone assay and transvaginal cervical length assessment after 24 weeks of gestation, another critical window of opportunity. J Matern Fetal Neonatal Med, 32(22), 3847-3858. doi:10.1080/14767058.2018.1482872 |
| 277 | Allen, R., & Aquilina, J. (2018). Prospective observational study to determine the accuracy of first-trimester serum biomarkers and uterine artery Dopplers in combination with maternal characteristics and arteriography for the prediction of women at risk of preeclampsia and other adverse pregnancy outcomes. J Matern Fetal Neonatal Med, 31(21), 2789-2806. doi:10.1080/14767058.2017.1355903 |
| 278 | Bakalis, S., Gallo, D. M., Mendez, O., Poon, L. C., & Nicolaides, K. H. (2015). Prediction of small-for-gestational-age neonates: screening by maternal biochemical markers at 30-34 weeks. Ultrasound Obstet Gynecol, 46(2), 208-215. doi:10.1002/uog.14861 |
| 279 | Bakalis, S., Peeva, G., Gonzalez, R., Poon, L. C., & Nicolaides, K. H. (2015). Prediction of small-for-gestational-age neonates: screening by biophysical and biochemical markers at 30-34 weeks. Ultrasound Obstet Gynecol, 46(4), 446-451. doi:10.1002/uog.14863 |
| 280 | Bakalis, S., Silva, M., Akolekar, R., Poon, L. C., & Nicolaides, K. H. (2015). Prediction of small-for-gestational-age neonates: screening by fetal biometry at 30-34 weeks. Ultrasound Obstet Gynecol, 45(5), 551-558. doi:10.1002/uog.14771 |
| 281 | Bakalis, S., Stoilov, B., Akolekar, R., Poon, L. C., & Nicolaides, K. H. (2015). Prediction of small-for-gestational-age neonates: screening by uterine artery Doppler and mean arterial pressure at 30-34 weeks. Ultrasound Obstet Gynecol, 45(6), 707-714. doi:10.1002/uog.14777 |
| 282 | Belfort, M. A., White, G. L., & Vermeulen, F. M. (2012). Association of fetal cranial shape with shoulder dystocia. Ultrasound Obstet Gynecol, 39(3), 304-309. doi:10.1002/uog.9066 |
| 283 | Bertozzi, S., Londero, A. P., Salvador, S., Grassi, T., Fruscalzo, A., Driul, L., & Marchesoni, D. (2011). Influence of the couple on hypertensive disorders during pregnancy: A retrospective cohort study. Pregnancy Hypertens, 1(2), 156-163. doi:10.1016/j.preghy.2011.01.005 |
| 284 | Bourdages, M., Demers, M., Dubé, S., Gasse, C., Girard, M., Boutin, A., . . . Demers, S. (2018). First-Trimester Abdominal Adipose Tissue Thickness to Predict Gestational Diabetes. J Obstet Gynaecol Can, 40(7), 883-887. doi:10.1016/j.jogc.2017.09.026 |
| 285 | Burgos-Artizzu, X. P., Perez-Moreno, A., Coronado-Gutierrez, D., Gratacos, E., & Palacio, M. (2019). Evaluation of an improved tool for non-invasive prediction of neonatal respiratory morbidity based on fully automated fetal lung ultrasound analysis. Sci Rep, 9, 7. doi:10.1038/s41598-019-38576-w |
| 286 | Calí, G., Timor-Tritsch, I. E., Forlani, F., Palacios-Jaraquemada, J., Monteagudo, A., Agten, A. K., . . . D'Antonio, F. (2020). Value of first-trimester ultrasound in prediction of third-trimester sonographic stage of placenta accreta spectrum disorder and surgical outcome. Ultrasound Obstet Gynecol, 55(4), 450-459. doi:10.1002/uog.21939 |
| 287 | Carvalho Neto, R. H., Viana Junior, A. B., Moron, A. F., Araujo Júnior, E., Carvalho, F. H. C., & Feitosa, H. N. (2019). Assessment of the angle of progression and distance perineum-head in the prediction of type of delivery and duration of labor using intrapartum ultrasonography. J Matern Fetal Neonatal Med, 1-9. doi:10.1080/14767058.2019.1666818 |
| 288 | Cavallaro, A., Veglia, M., Svirko, E., Vannuccini, S., Volpe, G., & Impey, L. (2018). Using fetal abdominal circumference growth velocity in the prediction of adverse outcome in near-term small-for-gestational-age fetuses. Ultrasound Obstet Gynecol, 52(4), 494-500. doi:10.1002/uog.18988 |
| 289 | Cetinkaya, E. S., Berker, B., Aytac, R., Atabekoglu, C., Sonmezer, M., & Ozmen, B. (2013). The value of the progesterone-to-estradiol ratio on the day of hCG administration in predicting ongoing pregnancy and live birth rates in normoresponders undergoing GnRH antagonist cycles. Eur J Obstet Gynecol Reprod Biol, 170(2), 452-457. doi:10.1016/j.ejogrb.2013.07.033 |
| 290 | Chen, T., Xu, X. Q., Shi, H. B., Yang, Z. Q., Zhou, X., & Pan, Y. (2017). Conventional MRI features for predicting the clinical outcome of patients with invasive placenta. Diagn Interv Radiol, 23(3), 173-179. doi:10.5152/dir.2016.16412 |
| 291 | Chetty, M., Sawyer, E., Dew, T., Chapman, A. J., & Elson, J. (2011). The use of novel biochemical markers in predicting spontaneously resolving 'pregnancies of unknown location'. Hum Reprod, 26(6), 1318-1323. doi:10.1093/humrep/der064 |
| 292 | Chu, C., Zhao, S., Ding, M., Liu, M., Zhang, Y., Bao, L., . . . Li, W. (2019). Combining Clinical Characteristics and Specific Magnetic Resonance Imaging Features to Predict Placenta Accreta. J Comput Assist Tomogr, 43(5), 775-779. doi:10.1097/rct.0000000000000894 |
| 293 | Ciobanou, A., Jabak, S., De Castro, H., Frei, L., Akolekar, R., & Nicolaides, K. H. (2019). Biomarkers of impaired placentation at 35-37 weeks' gestation in the prediction of adverse perinatal outcome. Ultrasound Obstet Gynecol, 54(1), 79-86. doi:10.1002/uog.20346 |
| 294 | Ciobanu, A., Anthoulakis, C., Syngelaki, A., Akolekar, R., & Nicolaides, K. H. (2019). Prediction of small-for-gestational-age neonates at 35-37 weeks' gestation: contribution of maternal factors and growth velocity between 32 and 36 weeks. Ultrasound Obstet Gynecol, 53(5), 630-637. doi:10.1002/uog.20267 |
| 295 | Ciobanu, A., Formuso, C., Syngelaki, A., Akolekar, R., & Nicolaides, K. H. (2019). Prediction of small-for-gestational-age neonates at 35-37 weeks' gestation: contribution of maternal factors and growth velocity between 20 and 36 weeks. Ultrasound Obstet Gynecol, 53(4), 488-495. doi:10.1002/uog.20243 |
| 296 | Crovetto, F., Figueras, F., Triunfo, S., Crispi, F., Rodriguez-Sureda, V., Peguero, A., . . . Gratacos, E. (2014). Added value of angiogenic factors for the prediction of early and late preeclampsia in the first trimester of pregnancy. Fetal Diagn Ther, 35(4), 258-266. doi:10.1159/000358302 |
| 297 | Cruz-Martinez, R., Moreno-Alvarez, O., Hernandez-Andrade, E., Castañon, M., Done, E., Martinez, J. M., . . . Gratacos, E. (2010). Contribution of intrapulmonary artery Doppler to improve prediction of survival in fetuses with congenital diaphragmatic hernia treated with fetal endoscopic tracheal occlusion. Ultrasound Obstet Gynecol, 35(5), 572-577. doi:10.1002/uog.7593 |
| 298 | Damaso, E. L., Rolnik, D. L., Cavalli, R. C., Quintana, S. M., Duarte, G., da Silva Costa, F., & Marcolin, A. (2019). Prediction of Preterm Birth by Maternal Characteristics and Medical History in the Brazilian Population. J Pregnancy, 2019, 4395217. doi:10.1155/2019/4395217 |
| 299 | D'Antonio, F., Thilaganathan, B., Laoreti, A., & Khalil, A. (2018). Birth-weight discordance and neonatal morbidity in twin pregnancy: analysis of STORK multiple pregnancy cohort. Ultrasound Obstet Gynecol, 52(5), 586-592. doi:10.1002/uog.18916 |
| 300 | Delić, R., Štefanović, M., Krivec, Š., & Weber, V. (2014). Statistical regression model of standard and new laboratory markers and its usefulness in prediction of preeclampsia. J Matern Fetal Neonatal Med, 27(4), 388-392. doi:10.3109/14767058.2013.818121 |
| 301 | Di Lorenzo, G., Ceccarello, M., Cecotti, V., Ronfani, L., Monasta, L., Vecchi Brumatti, L., . . . D'Ottavio, G. (2012). First trimester maternal serum PIGF, free β-hCG, PAPP-A, PP-13, uterine artery Doppler and maternal history for the prediction of preeclampsia. Placenta, 33(6), 495-501. doi:10.1016/j.placenta.2012.03.003 |
| 302 | Di Martino, D. D., Stampalija, T., Rosti, E., Casati, D., Signorelli, V., Zullino, S., . . . Ferrazzi, E. (2016). Bedside cardiovascular maternal interrogation in the first trimester to predict different phenotypes of hypertensive disorders in pregnancy. Pregnancy Hypertens, 6(4), 300-305. doi:10.1016/j.preghy.2016.06.002 |
| 303 | Direkvand-Moghadam, A., Khosravi, A., & Sayehmiri, K. (2012). Predictive factors for preeclampsia in pregnant women: a unvariate and multivariate logistic regression analysis. Acta Biochim Pol, 59(4), 673-677. |
| 304 | Doulaveris, G., Gallagher, P., Romney, E., Richley, M., Gebb, J., Rosner, M., & Dar, P. (2018). Fetal abdominal circumference in the second trimester and prediction of small for gestational age at birth. J Matern Fetal Neonatal Med, 1-181. doi:10.1080/14767058.2018.1554039 |
| 305 | Ducarme, G., Desroys du Roure, F., Grange, J., Vital, M., Le Thuaut, A., & Crespin-Delcourt, I. (2019). Predictive factors of subsequent insulin requirement for glycemic control during pregnancy at diagnosis of gestational diabetes mellitus. Int J Gynaecol Obstet, 144(3), 265-270. doi:10.1002/ijgo.12753 |
| 306 | Eggebø, T. M., Hassan, W. A., Salvesen, K., Lindtjørn, E., & Lees, C. C. (2014). Sonographic prediction of vaginal delivery in prolonged labor: a two-center study. Ultrasound Obstet Gynecol, 43(2), 195-201. doi:10.1002/uog.13210 |
| 307 | El-Achi, V., de Vries, B., O'Brien, C., Park, F., Tooher, J., & Hyett, J. (2020). First-Trimester Prediction of Preterm Prelabour Rupture of Membranes. Fetal Diagn Ther, 1-6. doi:10.1159/000506541 |
| 308 | Ellaithy, M., Asiri, M., Rateb, A., Altraigey, A., & Abdallah, K. (2018). Prediction of recurrent ectopic pregnancy: A five-year follow-up cohort study. Eur J Obstet Gynecol Reprod Biol, 225, 70-78. doi:10.1016/j.ejogrb.2018.04.007 |
| 309 | Fadigas, C., Guerra, L., Garcia-Tizon Larroca, S., Poon, L. C., & Nicolaides, K. H. (2015). Prediction of small-for-gestational-age neonates: screening by uterine artery Doppler and mean arterial pressure at 35-37 weeks. Ultrasound Obstet Gynecol, 45(6), 715-721. doi:10.1002/uog.14847 |
| 310 | Fadigas, C., Peeva, G., Mendez, O., Poon, L. C., & Nicolaides, K. H. (2015). Prediction of small-for-gestational-age neonates: screening by placental growth factor and soluble fms-like tyrosine kinase-1 at 35-37 weeks. Ultrasound Obstet Gynecol, 46(2), 191-197. doi:10.1002/uog.14862 |
| 311 | Fadigas, C., Saiid, Y., Gonzalez, R., Poon, L. C., & Nicolaides, K. H. (2015). Prediction of small-for-gestational-age neonates: screening by fetal biometry at 35-37 weeks. Ultrasound Obstet Gynecol, 45(5), 559-565. doi:10.1002/uog.14816 |
| 312 | Familiari, A., Bhide, A., Morlando, M., Scala, C., Khalil, A., & Thilaganathan, B. (2016). Mid-pregnancy fetal biometry, uterine artery Doppler indices and maternal demographic characteristics: role in prediction of small-for-gestational-age birth. Acta Obstet Gynecol Scand, 95(2), 238-244. doi:10.1111/aogs.12804 |
| 313 | Familiari, A., Scala, C., Morlando, M., Bhide, A., Khalil, A., & Thilaganathan, B. (2016). Mid-pregnancy fetal growth, uteroplacental Doppler indices and maternal demographic characteristics: role in prediction of stillbirth. Acta Obstet Gynecol Scand, 95(11), 1313-1318. doi:10.1111/aogs.13012 |
| 314 | Farina, A., Rapacchia, G., Freni Sterrantino, A., Pula, G., Morano, D., & Rizzo, N. (2011). Prospective evaluation of ultrasound and biochemical-based multivariable models for the prediction of late pre-eclampsia. Prenat Diagn, 31(12), 1147-1152. doi:10.1002/pd.2849 |
| 315 | Fiolna, M., Kostiv, V., Anthoulakis, C., Akolekar, R., & Nicolaides, K. H. (2019). Prediction of adverse perinatal outcome by cerebroplacental ratio in women undergoing induction of labor. Ultrasound Obstet Gynecol, 53(4), 473-480. doi:10.1002/uog.20173 |
| 316 | Fiolna, M., Machuca, M., Karampitsakos, T., Akolekar, R., & Nicolaides, K. H. (2019). Prediction of adverse perinatal outcome by serum placental growth factor and soluble fms-like tyrosine kinase-1 in women undergoing induction of labor. Ultrasound Obstet Gynecol, 54(5), 604-608. doi:10.1002/uog.20853 |
| 317 | Fishel, S., Campbell, A., Montgomery, S., Smith, R., Nice, L., Duffy, S., . . . Beccles, A. (2018). Time-lapse imaging algorithms rank human preimplantation embryos according to the probability of live birth. Reprod Biomed Online, 37(3), 304-313. doi:10.1016/j.rbmo.2018.05.016 |
| 318 | Fontanella, F., van Scheltema, P. N. A., Duin, L., Cohen-Overbeek, T. E., Pajkrt, E., Bekker, M. N., . . . Bilardo, C. M. (2019). Antenatal staging of congenital lower urinary tract obstruction. Ultrasound Obstet Gynecol, 53(4), 520-524. doi:10.1002/uog.19172 |
| 319 | Gasse, C., Boutin, A., Coté, M., Chaillet, N., Bujold, E., & Demers, S. (2018). First-trimester mean arterial blood pressure and the risk of preeclampsia: The Great Obstetrical Syndromes (GOS) study. Pregnancy Hypertens, 12, 178-182. doi:10.1016/j.preghy.2017.11.005 |
| 320 | Giguère, Y., Massé, J., Thériault, S., Bujold, E., Lafond, J., Rousseau, F., & Forest, J. C. (2015). Screening for pre-eclampsia early in pregnancy: performance of a multivariable model combining clinical characteristics and biochemical markers. Bjog, 122(3), 402-410. doi:10.1111/1471-0528.13050 |
| 321 | Gómez-Arriaga, P. I., Herraiz, I., López-Jiménez, E. A., Escribano, D., Denk, B., & Galindo, A. (2014). Uterine artery Doppler and sFlt-1/PlGF ratio: prognostic value in early-onset pre-eclampsia. Ultrasound Obstet Gynecol, 43(5), 525-532. doi:10.1002/uog.13224 |
| 322 | Grynnerup, A. G., Løssl, K., Pilsgaard, F., Lunding, S. A., Storgaard, M., Bogstad, J. W., . . . Pinborg, A. (2019). Prediction of the lower serum anti-Müllerian hormone threshold for ovarian stimulation prior to in-vitro fertilization using the Elecsys® AMH assay: a prospective observational study. Reprod Biol Endocrinol, 17(1), 11. doi:10.1186/s12958-019-0452-4 |
| 323 | Gurgel Alves, J. A., Praciano De Sousa, P. C., Bezerra Maia E Holanda Moura, S., Kane, S. C., & Da Silva Costa, F. (2014). First-trimester maternal ophthalmic artery Doppler analysis for prediction of pre-eclampsia. Ultrasound in Obstetrics and Gynecology, 44(4), 411-418. doi:10.1002/uog.13338 |
| 324 | Guzman, L., Ortega-Hrepich, C., Polyzos, N. P., Anckaert, E., Verheyen, G., Coucke, W., . . . De Vos, M. (2013). A prediction model to select PCOS patients suitable for IVM treatment based on anti-Mullerian hormone and antral follicle count. Hum Reprod, 28(5), 1261-1266. doi:10.1093/humrep/det034 |
| 325 | Hamilton, E., & Kimanani, E. K. (1994). Intrapartum prediction of fetal status and assessment of labour progress. Baillieres Clin Obstet Gynaecol, 8(3), 567-581. doi:10.1016/s0950-3552(05)80199-3 |
| 326 | Hao, M., & Lin, L. (2017). Fasting plasma glucose and body mass index during the first trimester of pregnancy as predictors of gestational diabetes mellitus in a Chinese population. Endocr J, 64(5), 561-569. doi:10.1507/endocrj.EJ16-0359 |
| 327 | Hassan, A. M. A., Kotb, M. M. M., AwadAllah, A. M. A., Shehata, N. A. A., & Wahba, A. (2017). Follicular sensitivity index (FSI): a novel tool to predict clinical pregnancy rate in IVF/ICSI cycles. J Assist Reprod Genet, 34(10), 1317-1324. doi:10.1007/s10815-017-0984-5 |
| 328 | Hernández-Martínez, A., Pascual-Pedreño, A. I., Baño-Garnés, A. B., Melero-Jiménez, M. R., Tenías-Burillo, J. M., & Molina-Alarcón, M. (2016). Predictive model for risk of cesarean section in pregnant women after induction of labor. Arch Gynecol Obstet, 293(3), 529-538. doi:10.1007/s00404-015-3856-1 |
| 329 | Hiersch, L., Krispin, E., Aviram, A., Mor-Shacham, M., Gabbay-Benziv, R., Yogev, Y., & Ashwal, E. (2017). Predictors for prolonged interval from premature rupture of membranes to spontaneous onset of labor at term. J Matern Fetal Neonatal Med, 30(12), 1465-1470. doi:10.1080/14767058.2016.1219992 |
| 330 | Hilal, Z., Mrkvicka, J., Rezniczek, G. A., Dogan, A., & Tempfer, C. B. (2017). Accuracy of intrapartum fetal blood gas analysis by scalp sampling: A retrospective cohort study. Medicine (Baltimore), 96(49), e8839. doi:10.1097/md.0000000000008839 |
| 331 | Holst, R. M., Hagberg, H., Wennerholm, U. B., Skogstrand, K., Thorsen, P., & Jacobsson, B. (2011). Prediction of microbial invasion of the amniotic cavity in women with preterm labour: analysis of multiple proteins in amniotic and cervical fluids. Bjog, 118(2), 240-249. doi:10.1111/j.1471-0528.2010.02765.x |
| 332 | Huang, Y., Chen, X., Chen, X., Feng, Y., Guo, H., Li, S., . . . Hu, J. (2018). Angiopoietin-like protein 8 in early pregnancy improves the prediction of gestational diabetes. Diabetologia, 61(3), 574-580. doi:10.1007/s00125-017-4505-y |
| 333 | Huang, J., Lin, J., Gao, H., Wang, Y., Zhu, X., Lu, X., . . . Kuang, Y. (2019). Anti-müllerian Hormone for the Prediction of Ovarian Response in Progestin-Primed Ovarian Stimulation Protocol for IVF. Front Endocrinol (Lausanne), 10, 325. doi:10.3389/fendo.2019.00325 |
| 334 | Iwatani, S., Mizobuchi, M., Tanaka, S., Inomata, K., Sakai, H., Yoshimoto, S., & Nakao, H. (2013). Increased volume of tracheal aspirate fluid predicts the development of bronchopulmonary dysplasia. Early Hum Dev, 89(2), 113-117. doi:10.1016/j.earlhumdev.2012.08.007 |
| 335 | Izci-Balserak, B., Zhu, B., Gurubhagavatula, I., Keenan, B. T., & Pien, G. W. (2019). A Screening Algorithm for Obstructive Sleep Apnea in Pregnancy. Ann Am Thorac Soc, 16(10), 1286-1294. doi:10.1513/AnnalsATS.201902-131OC |
| 336 | Kaur, S., Chawla, D., Pathak, U., & Jain, S. (2012). Predischarge non-invasive risk assessment for prediction of significant hyperbilirubinemia in term and late preterm neonates. J Perinatol, 32(9), 716-721. doi:10.1038/jp.2011.170 |
| 337 | Kienast, C., Moya, W., Rodriguez, O., Jijon, A., & Geipel, A. (2016). Predictive value of angiogenic factors, clinical risk factors and uterine artery Doppler for pre-eclampsia and fetal growth restriction in second and third trimester pregnancies in an Ecuadorian population. Journal of Maternal-Fetal & Neonatal Medicine, 29(4), 537-543. doi:10.3109/14767058.2015.1012063 |
| 338 | Kim, D. H., Shin, S. H., Kim, E. K., & Kim, H. S. (2018). Association of increased cord blood soluble endoglin with the development of bronchopulmonary dysplasia in preterm infants with maternal preeclampsia. Pregnancy Hypertens, 13, 148-153. doi:10.1016/j.preghy.2018.06.002 |
| 339 | Kowalski, J. T., Melero, G. H., Mahal, A., Genadry, R., & Bradley, C. S. (2017). Do patient characteristics impact the relationship between anatomic prolapse and vaginal bulge symptoms? Int Urogynecol J, 28(3), 391-396. doi:10.1007/s00192-016-3151-0 |
| 340 | Kurakazu, M., Yotsumoto, F., Arima, H., Izuchi, D., Urushiyama, D., Miyata, K., . . . Miyamoto, S. (2019). The combination of maternal blood and amniotic fluid biomarkers improves the predictive accuracy of histologic chorioamnionitis. Placenta, 80, 4-7. doi:10.1016/j.placenta.2019.03.007 |
| 341 | Lédée, N., Gridelet, V., Ravet, S., Jouan, C., Gaspard, O., Wenders, F., . . . Perrier d'Hauterive, S. (2013). Impact of follicular G-CSF quantification on subsequent embryo transfer decisions: a proof of concept study. Hum Reprod, 28(2), 406-413. doi:10.1093/humrep/des354 |
| 342 | Lesmes, C., Gallo, D. M., Gonzalez, R., Poon, L. C., & Nicolaides, K. H. (2015). Prediction of small-for-gestational-age neonates: screening by maternal serum biochemical markers at 19-24 weeks. Ultrasound Obstet Gynecol, 46(3), 341-349. doi:10.1002/uog.14899 |
| 343 | Lesmes, C., Gallo, D. M., Panaiotova, J., Poon, L. C., & Nicolaides, K. H. (2015). Prediction of small-for-gestational-age neonates: screening by fetal biometry at 19-24 weeks. Ultrasound Obstet Gynecol, 46(2), 198-207. doi:10.1002/uog.14826 |
| 344 | Lesmes, C., Gallo, D. M., Saiid, Y., Poon, L. C., & Nicolaides, K. H. (2015). Prediction of small-for-gestational-age neonates: screening by uterine artery Doppler and mean arterial pressure at 19-24 weeks. Ultrasound Obstet Gynecol, 46(3), 332-340. doi:10.1002/uog.14855 |
| 345 | Li, P., Yin, Y., Lin, S., Cui, J., Zhou, S., Li, L., & Fan, J. (2016). Utility of Pregestational Body Mass Index and Initial Fasting Plasma Glucose in Predicting Gestational Diabetes Mellitus. Am J Med Sci, 351(4), 420-425. doi:10.1016/j.amjms.2016.02.007 |
| 346 | Li, P., Lin, S., Cui, J., Li, L., Zhou, S., & Fan, J. (2018). First Trimester Neck Circumference as a Predictor for the Development of Gestational Diabetes Mellitus. Am J Med Sci, 355(2), 149-152. doi:10.1016/j.amjms.2017.09.012 |
| 347 | Li, B., Lin, L., Yang, H., Zhu, Y., Wei, Y., Li, X., . . . Xin, H. (2018). The value of the 24-h proteinuria in evaluating the severity of preeclampsia and predicting its adverse maternal outcomes. Hypertens Pregnancy, 37(3), 118-125. doi:10.1080/10641955.2018.1487564 |
| 348 | Liu, Y., Traskin, M., Lorch, S. A., George, E. I., & Small, D. (2015). Ensemble of trees approaches to risk adjustment for evaluating a hospital's performance. Health Care Manag Sci, 18(1), 58-66. doi:10.1007/s10729-014-9272-4 |
| 349 | Llaneza-Suarez, D., Llaneza, P., González, C., De-La-Fuente, P., García-Ochoa, C., Garrido, P., . . . Pérez-López, F. R. (2014). Assessment of follicular fluid leptin levels and insulin resistance as outcome predictors in women undergoing *in vitro* fertilization-intracytoplasmic sperm injection. Fertil Steril, 102(6), 1619-1625. doi:10.1016/j.fertnstert.2014.09.012 |
| 350 | Lu, X., Khor, S., Zhu, Q., Sun, L., Wang, Y., Chen, Q., . . . Kuang, Y. (2018). Decrease in preovulatory serum estradiol is a valuable marker for predicting premature ovulation in natural/unstimulated *in vitro* fertilization cycle. J Ovarian Res, 11(1), 96. doi:10.1186/s13048-018-0469-x |
| 351 | Lukaszuk, K., Kunicki, M., Liss, J., Lukaszuk, M., & Jakiel, G. (2013). Use of ovarian reserve parameters for predicting live births in women undergoing *in vitro* fertilization. Eur J Obstet Gynecol Reprod Biol, 168(2), 173-177. doi:10.1016/j.ejogrb.2013.01.013 |
| 352 | Manley, B. J., Doyle, L. W., Owen, L. S., & Davis, P. G. (2016). Extubating Extremely Preterm Infants: Predictors of Success and Outcomes following Failure. J Pediatr, 173, 45-49. doi:10.1016/j.jpeds.2016.02.016 |
| 353 | Mastrodima, S., Akolekar, R., Yerlikaya, G., Tzelepis, T., & Nicolaides, K. H. (2016). Prediction of stillbirth from biochemical and biophysical markers at 11-13 weeks. Ultrasound Obstet Gynecol, 48(5), 613-617. doi:10.1002/uog.17289 |
| 354 | McKeating, D. R., Clifton, V. L., Hurst, C. P., Fisher, J. J., Bennett, W. W., & Perkins, A. V. (2020). Elemental Metabolomics for Prediction of Term Gestational Outcomes Utilising 18-Week Maternal Plasma and Urine Samples. Biol Trace Elem Res. doi:10.1007/s12011-020-02127-6 |
| 355 | Miranda, J., Rodriguez-Lopez, M., Triunfo, S., Sairanen, M., Kouru, H., Parra-Saavedra, M., . . . Gratacós, E. (2017). Prediction of fetal growth restriction using estimated fetal weight vs a combined screening model in the third trimester. Ultrasound Obstet Gynecol, 50(5), 603-611. doi:10.1002/uog.17393 |
| 356 | Mizrachi, Y., Barber, E., Kovo, M., Bar, J., & Lurie, S. (2018). Prediction of vaginal birth after one ceasarean delivery for non-progressive labor. Arch Gynecol Obstet, 297(1), 85-91. doi:10.1007/s00404-017-4569-4 |
| 357 | Moore Simas, T. A., Crawford, S. L., Bathgate, S., Yan, J., Robidoux, L., Moore, M., & Maynard, S. E. (2014). Angiogenic biomarkers for prediction of early preeclampsia onset in high-risk women. J Matern Fetal Neonatal Med, 27(10), 1038-1048. doi:10.3109/14767058.2013.847415 |
| 358 | Morales-Roselló, J., Galindo, A., Herraiz, I., Gil, M. M., Brik, M., De Paco-Matallana, C., . . . Perales-Marín, A. (2019). Is it possible to predict late antepartum stillbirth by means of cerebroplacental ratio and maternal characteristics? J Matern Fetal Neonatal Med, 1-7. doi:10.1080/14767058.2019.1566900 |
| 359 | Morales-Roselló, J., Khalil, A., Fornés-Ferrer, V., & Perales-Marín, A. (2019). Accuracy of the fetal cerebroplacental ratio for the detection of intrapartum compromise in nonsmall fetuses(). J Matern Fetal Neonatal Med, 32(17), 2842-2852. doi:10.1080/14767058.2018.1450380 |
| 360 | Moro, F., Tropea, A., Scarinci, E., Leoncini, E., Boccia, S., Federico, A., . . . Apa, R. (2016). Anti-Müllerian hormone concentrations and antral follicle counts for the prediction of pregnancy outcomes after intrauterine insemination. Int J Gynaecol Obstet, 133(1), 64-68. doi:10.1016/j.ijgo.2015.08.021 |
| 361 | Murali, A. R., Devarbhavi, H., Venkatachala, P. R., Singh, R., & Sheth, K. A. (2014). Factors that predict 1-month mortality in patients with pregnancy-specific liver disease. Clin Gastroenterol Hepatol, 12(1), 109-113. doi:10.1016/j.cgh.2013.06.018 |
| 362 | Oh, K. J., Park, K. H., Jeong, E. H., Lee, S. Y., Ryu, A., & Kim, S. N. (2012). The change in cervical length over time as a predictor of preterm delivery in asymptomatic women with twin pregnancies who have a normal mid-trimester cervical length. Twin Res Hum Genet, 15(4), 516-521. doi:10.1017/thg.2012.27 |
| 363 | Ohkuchi, A., Ishibashi, O., Hirashima, C., Takahashi, K., Matsubara, S., Takizawa, T., & Suzuki, M. (2012). Plasma level of hydroxysteroid (17-β) dehydrogenase 1 in the second trimester is an independent risk factor for predicting preeclampsia after adjusting for the effects of mean blood pressure, bilateral notching and plasma level of soluble fms-like tyrosine kinase 1/placental growth factor ratio. Hypertens Res, 35(12), 1152-1158. doi:10.1038/hr.2012.109 |
| 364 | Olusanya, B. O., Slusher, T. M., Imosemi, D. O., & Emokpae, A. A. (2017). Maternal detection of neonatal jaundice during birth hospitalization using a novel two-color icterometer. PLoS One, 12(8), e0183882. doi:10.1371/journal.pone.0183882 |
| 365 | Orabona, R., Gerosa, V., Gregorini, M. E., Pagani, G., Prefumo, F., Valcamonico, A., & Frusca, T. (2015). The prognostic role of various indices and ratios of Doppler velocimetry in patients with pre-eclampsia. Clin Exp Hypertens, 37(1), 57-62. doi:10.3109/10641963.2014.897723 |
| 366 | Ouzounian, J. G., Korst, L. M., Sanchez, M., Chauhan, S., Gherman, R. B., Opper, N., & Wilson, M. L. (2016). Clinical Risk Factors Do Not Predict Shoulder Dystocia. J Reprod Med, 61(11-12), 575-580. |
| 367 | Oylumlu, M., Ozler, A., Yildiz, A., Oylumlu, M., Acet, H., Polat, N., . . . Ertas, F. (2014). New inflammatory markers in pre-eclampsia: echocardiographic epicardial fat thickness and neutrophil to lymphocyte ratio. Clin Exp Hypertens, 36(7), 503-507. doi:10.3109/10641963.2013.863324 |
| 368 | Oztas, E., Ozler, S., Caglar, A. T., & Yucel, A. (2016). Analysis of first and second trimester maternal serum analytes for the prediction of morbidly adherent placenta requiring hysterectomy. Kaohsiung J Med Sci, 32(11), 579-585. doi:10.1016/j.kjms.2016.08.011 |
| 369 | Palatnik, A., Grobman, W. A., Hellendag, M. G., Janetos, T. M., Gossett, D. R., & Miller, E. S. (2016). Predictors of shoulder dystocia at the time of operative vaginal delivery. Am J Obstet Gynecol, 215(5), 624.e621-624.e625. doi:10.1016/j.ajog.2016.06.001 |
| 370 | Papaioannou, G. I., Syngelaki, A., Maiz, N., Ross, J. A., & Nicolaides, K. H. (2011). Ultrasonographic prediction of early miscarriage. Hum Reprod, 26(7), 1685-1692. doi:10.1093/humrep/der130 |
| 371 | Park, H. J., Lyu, S. W., Seok, H. H., Yoon, T. K., & Lee, W. S. (2015). Anti-Müllerian hormone levels as a predictor of clinical pregnancy in *in vitro* fertilization/intracytoplasmic sperm injection-embryo transfer cycles in patients over 40 years of age. Clin Exp Reprod Med, 42(4), 143-148. doi:10.5653/cerm.2015.42.4.143 |
| 372 | Perales, A., Delgado, J. L., de la Calle, M., García-Hernández, J. A., Escudero, A. I., Campillos, J. M., . . . Álvarez, F. V. (2017). sFlt-1/PlGF for prediction of early-onset pre-eclampsia: STEPS (Study of Early Pre-eclampsia in Spain). Ultrasound Obstet Gynecol, 50(3), 373-382. doi:10.1002/uog.17373 |
| 373 | Poon, L. C., Akolekar, R., Lachmann, R., Beta, J., & Nicolaides, K. H. (2010). Hypertensive disorders in pregnancy: screening by biophysical and biochemical markers at 11-13 weeks. Ultrasound Obstet Gynecol, 35(6), 662-670. doi:10.1002/uog.7628 |
| 374 | Poon, L. C., Lesmes, C., Gallo, D. M., Akolekar, R., & Nicolaides, K. H. (2015). Prediction of small-for-gestational-age neonates: screening by biophysical and biochemical markers at 19-24 weeks. Ultrasound Obstet Gynecol, 46(4), 437-445. doi:10.1002/uog.14904 |
| 375 | Posthumus, A. G., Birnie, E., van Veen, M. J., Steegers, E. A., & Bonsel, G. J. (2016). An antenatal prediction model for adverse birth outcomes in an urban population: The contribution of medical and non-medical risks. Midwifery, 38, 78-86. doi:10.1016/j.midw.2015.11.006 |
| 376 | Punnose, J., Malhotra, R. K., Sukhija, K., Mathew, A., Sharma, A., & Choudhary, N. (2020). Glycated haemoglobin in the first trimester: A predictor of gestational diabetes mellitus in pregnant Asian Indian women. Diabetes Res Clin Pract, 159, 107953. doi:10.1016/j.diabres.2019.107953 |
| 377 | Ramos-Medina, R., García-Segovia, Á., León, J. A., Alonso, B., Tejera-Alhambra, M., Gil, J., . . . Sánchez-Ramón, S. (2013). New decision-tree model for defining the risk of reproductive failure. Am J Reprod Immunol, 70(1), 59-68. doi:10.1111/aji.12098 |
| 378 | Ren, Z., Zhe, D., Li, Z., Sun, X. P., Yang, K., & Lin, L. (2020). Study on the correlation and predictive value of serum pregnancy-associated plasma protein A, triglyceride and serum 25-hydroxyvitamin D levels with gestational diabetes mellitus. World J Clin Cases, 8(5), 864-873. doi:10.12998/wjcc.v8.i5.864 |
| 379 | Rhenman, A., Berglund, L., Brodin, T., Olovsson, M., Milton, K., Hadziosmanovic, N., & Holte, J. (2015). Which set of embryo variables is most predictive for live birth? A prospective study in 6252 single embryo transfers to construct an embryo score for the ranking and selection of embryos. Hum Reprod, 30(1), 28-36. doi:10.1093/humrep/deu295 |
| 380 | Riboni, F., Vitulo, A., Plebani, M., Dell'avanzo, M., Battagliarin, G., & Paternoster, D. (2012). Combination of biochemical markers in predicting pre-term delivery. Arch Gynecol Obstet, 285(1), 61-66. doi:10.1007/s00404-011-1915-9 |
| 381 | Rizzo, G., Mattioli, C., Mappa, I., Bitsadze, V., Khizroeva, J., Makatsariya, A., & D'Antonio, F. (2019). Antepartum ultrasound prediction of failed vacuum-assisted operative delivery: a prospective cohort study. J Matern Fetal Neonatal Med, 1-7. doi:10.1080/14767058.2019.1683540 |
| 382 | Rizzo, G., Aloisio, F., Yacoub, M., Bitsadze, V., Słodki, M., Makatsariya, A., & D'Antonio, F. (2019). Ultrasound assessment of the cervix in predicting successful membrane sweeping: a prospective observational study. J Matern Fetal Neonatal Med, 1-7. doi:10.1080/14767058.2019.1619689 |
| 383 | Rizzo, G., Mappa, I., Bitsadze, V., Słodki, M., Khizroeva, J., Makatsarya, A., & D'Antonio, F. (2019). Role of Doppler ultrasound in predicting perinatal outcome in pregnancies complicated by late-onset fetal growth restriction at the time of diagnosis: a prospective cohort study. Ultrasound Obstet Gynecol. doi:10.1002/uog.20406 |
| 384 | Rizzo, G., Mappa, I., Bitsadze, V., Słodki, M., Khizroeva, J., Makatsarya, A., & D'Antonio, F. (2019). Role of first-trimester umbilical vein flow in predicting fetal macrosomia: a matched case-control study. Ultrasound Obstet Gynecol. doi:10.1002/uog.20408 |
| 385 | Sallmon, H., Weber, S. C., Dirks, J., Schiffer, T., Klippstein, T., Stein, A., . . . Koehne, P. (2018). Association between Platelet Counts before and during Pharmacological Therapy for Patent Ductus Arteriosus and Treatment Failure in Preterm Infants. Front Pediatr, 6, 41. doi:10.3389/fped.2018.00041 |
| 386 | Sanhal, C. Y., Can Kavcar, M., Yucel, A., Erkeneklı, K., Erkaya, S., & Uygur, D. (2016). Comparison of plasma fetuin A levels in patients with early-onset pre-eclampsia vs late-onset pre-eclampsia. Eur J Obstet Gynecol Reprod Biol, 200, 108-112. doi:10.1016/j.ejogrb.2016.03.011 |
| 387 | Schneuer, F. J., Nassar, N., Tasevski, V., Morris, J. M., & Roberts, C. L. (2012). Association and predictive accuracy of high TSH serum levels in first trimester and adverse pregnancy outcomes. J Clin Endocrinol Metab, 97(9), 3115-3122. doi:10.1210/jc.2012-1193 |
| 388 | Schneuer, F. J., Roberts, C. L., Ashton, A. W., Guilbert, C., Tasevski, V., Morris, J. M., & Nassar, N. (2014). Angiopoietin 1 and 2 serum concentrations in first trimester of pregnancy as biomarkers of adverse pregnancy outcomes. Am J Obstet Gynecol, 210(4), 345.e341-345.e349. doi:10.1016/j.ajog.2013.11.012 |
| 389 | Schneuer, F. J., Roberts, C. L., Guilbert, C., Simpson, J. M., Algert, C. S., Khambalia, A. Z., . . . Nassar, N. (2014). Effects of maternal serum 25-hydroxyvitamin D concentrations in the first trimester on subsequent pregnancy outcomes in an Australian population. Am J Clin Nutr, 99(2), 287-295. doi:10.3945/ajcn.113.065672 |
| 390 | Schneuer, F. J., Nassar, N., Guilbert, C., Tasevski, V., Ashton, A. W., Morris, J. M., & Roberts, C. L. (2015). Evaluation of first trimester serum soluble endothelial cell-specific tyrosine kinase receptor in normal and affected pregnancies. J Matern Fetal Neonatal Med, 28(15), 1815-1821. doi:10.3109/14767058.2014.969233 |
| 391 | Scifres, C. M., Rohn, A., Odibo, A., Stamilio, D., & Macones, G. A. (2011). Predicting significant maternal morbidity in women attempting vaginal birth after cesarean section. Am J Perinatol, 28(3), 181-186. doi:10.1055/s-0030-1266159 |
| 392 | Sepúlveda-Martínez, A., Garrido, M., Caamano, E., Vega, M., Romero, C., & Parra-Cordero, M. (2017). Maternal Plasma Nerve Growth Factor at the 11+0-13+6 Weeks' Scan as a Potential Angiogenic Marker of Preeclampsia: A Pilot Study. Fetal Diagn Ther, 41(3), 202-208. doi:10.1159/000448035 |
| 393 | Sepúlveda-Martínez, A., Rencoret, G., Silva, M. C., Ahumada, P., Pedraza, D., Muñoz, H., . . . Parra-Cordero, M. (2019). First trimester screening for preterm and term pre-eclampsia by maternal characteristics and biophysical markers in a low-risk population. J Obstet Gynaecol Res, 45(1), 104-112. doi:10.1111/jog.13809 |
| 394 | Seravalli, V., Block-Abraham, D. M., Turan, O. M., Doyle, L. E., Kopelman, J. N., Atlas, R. O., . . . Baschat, A. A. (2014). First-trimester prediction of small-for-gestational age neonates incorporating fetal Doppler parameters and maternal characteristics. Am J Obstet Gynecol, 211(3), 261.e261-268. doi:10.1016/j.ajog.2014.03.022 |
| 395 | Sharma, A., Xin, Y., Chen, X., & Sood, B. G. (2019). Early prediction of moderate to severe bronchopulmonary dysplasia in extremely premature infants. Pediatr Neonatol. doi:10.1016/j.pedneo.2019.12.001 |
| 396 | Shinohara, S., Uchida, Y., Kasai, M., & Sunami, R. (2017). Association between the high soluble fms-like tyrosine kinase-1 to placental growth factor ratio and adverse outcomes in asymptomatic women with early-onset fetal growth restriction. Hypertens Pregnancy, 36(3), 269-275. doi:10.1080/10641955.2017.1334800 |
| 397 | Shinohara, S., Okuda, Y., Hirata, S., & Suzuki, K. (2020). Predictive possibility of the transverse cerebellar diameter to abdominal circumference ratio for small-for-gestational-age fetus suspected as a cause of maternal placental syndromes: a retrospective cohort study. Hypertens Pregnancy, 39(2), 145-151. doi:10.1080/10641955.2020.1747487 |
| 398 | Sirico, A., Lanzone, A., Mappa, I., Sarno, L., Słodki, M., Pitocco, D., . . . Rizzo, G. (2019). The role of first trimester fetal heart rate in the prediction of gestational diabetes: A multicenter study. Eur J Obstet Gynecol Reprod Biol, 243, 158-161. doi:10.1016/j.ejogrb.2019.10.019 |
| 399 | Skupski, D. W., Luks, F. I., Papanna, R., Walker, M., Bebbington, M., Ryan, G., . . . Bahtiyar, O. (2013). Laser ablation of placental anastomoses in twin-to-twin transfusion syndrome: preoperative predictors of death by recursive partitioning. Prenat Diagn, 33(3), 279-283. doi:10.1002/pd.4059 |
| 400 | Sonalkar, S., Koelper, N., Creinin, M. D., Atrio, J. M., Sammel, M. D., Mc, A. A., & Schreiber, C. A. (2020). Management of early pregnancy loss with mifepristone and misoprostol: clinical predictors of success from a randomized trial. Am J Obstet Gynecol. doi:10.1016/j.ajog.2020.04.006 |
| 401 | Srinivas, S. K., Larkin, J., Sammel, M. D., Appleby, D., Bastek, J., Andrela, C. M., . . . Elovitz, M. A. (2010). The use of angiogenic factors in discriminating preeclampsia: are they ready for prime time? J Matern Fetal Neonatal Med, 23(11), 1294-1300. doi:10.3109/14767051003677988 |
| 402 | Teoh, S. S. Y., Wang, Y., Li, Y., Leemaqz, S. Y., Dekker, G. A., Roberts, C. T., & Nie, G. (2019). Low Serum Levels of HtrA3 at 15 Weeks of Gestation Are Associated with Late-Onset Preeclampsia Development and Small for Gestational Age Birth. Fetal Diagn Ther, 46(6), 392-401. doi:10.1159/000497144 |
| 403 | Torricelli, M., Novembri, R., Voltolini, C., Conti, N., Biliotti, G., Piccolini, E., . . . Petraglia, F. (2011). Biochemical and biophysical predictors of the response to the induction of labor in nulliparous postterm pregnancy. Am J Obstet Gynecol, 204(1), 39.e31-36. doi:10.1016/j.ajog.2010.08.014 |
| 404 | Tsiartas, P., Holst, R. M., Wennerholm, U. B., Hagberg, H., Hougaard, D. M., Skogstrand, K., . . . Jacobsson, B. (2012). Prediction of spontaneous preterm delivery in women with threatened preterm labour: a prospective cohort study of multiple proteins in maternal serum. Bjog, 119(7), 866-873. doi:10.1111/j.1471-0528.2012.03328.x |
| 405 | Tuuli, M. G., Cahill, A., Stamilio, D., Macones, G., & Odibo, A. O. (2011). Comparative efficiency of measures of early fetal growth restriction for predicting adverse perinatal outcomes. Obstet Gynecol, 117(6), 1331-1340. doi:10.1097/AOG.0b013e31821ae239 |
| 406 | Vallikkannu, N., Lam, W. K., Omar, S. Z., & Tan, P. C. (2017). Insulin-like growth factor binding protein 1, Bishop score, and sonographic cervical length: tolerability and prediction of vaginal birth and vaginal birth within 24 hours following labour induction in nulliparous women. Bjog, 124(8), 1274-1283. doi:10.1111/1471-0528.14175 |
| 407 | Weiner, E., Mizrachi, Y., Grinstein, E., Feldstein, O., Rymer-Haskel, N., Juravel, E., . . . Kovo, M. (2016). The role of placental histopathological lesions in predicting recurrence of preeclampsia. Prenat Diagn, 36(10), 953-960. doi:10.1002/pd.4918 |
| 408 | White, C. R., Doherty, D. A., Henderson, J. J., Kohan, R., Newnham, J. P., & Pennell, C. E. (2012). Accurate prediction of hypoxic-ischaemic encephalopathy at delivery: a cohort study. J Matern Fetal Neonatal Med, 25(9), 1653-1659. doi:10.3109/14767058.2011.653421 |
| 409 | Wilson, D. L., Walker, S. P., Fung, A. M., O'Donoghue, F., Barnes, M., & Howard, M. (2013). Can we predict sleep-disordered breathing in pregnancy? The clinical utility of symptoms. J Sleep Res, 22(6), 670-678. doi:10.1111/jsr.12063 |
| 410 | Xiang, S., Li, L., Wang, L., Liu, J., Tan, Y., & Hu, J. (2019). A decision tree model of cerebral palsy based on risk factors. J Matern Fetal Neonatal Med, 1-6. doi:10.1080/14767058.2019.1702944 |
| 411 | Xu, L., Dai, S., Sun, L., Shen, J., Lv, C., & Chen, X. (2020). Evaluation of 2 ultrasonic indicators as predictors of difficult laryngoscopy in pregnant women: A prospective, double blinded study. Medicine (Baltimore), 99(3), e18305. doi:10.1097/md.0000000000018305 |
| 412 | Yang, W., Yang, R., Lin, M., Yang, Y., Song, X., Zhang, J., . . . Jiao, J. (2018). Body mass index and basal androstenedione are independent risk factors for miscarriage in polycystic ovary syndrome. Reprod Biol Endocrinol, 16(1), 119. doi:10.1186/s12958-018-0438-7 |
| 413 | Yefet, E., Jeda, E., Tzur, A., & Nachum, Z. (2020). Markers for undiagnosed type 2 diabetes mellitus during pregnancy-A population-based retrospective cohort study. J Diabetes, 12(3), 205-214. doi:10.1111/1753-0407.12985 |
| 414 | Yerlikaya, G., Akolekar, R., McPherson, K., Syngelaki, A., & Nicolaides, K. H. (2016). Prediction of stillbirth from maternal demographic and pregnancy characteristics. Ultrasound Obstet Gynecol, 48(5), 607-612. doi:10.1002/uog.17290 |
| 415 | Youssef, A., Maroni, E., Cariello, L., Bellussi, F., Montaguti, E., Salsi, G., . . . Ghi, T. (2014). Fetal head-symphysis distance and mode of delivery in the second stage of labor. Acta Obstet Gynecol Scand, 93(10), 1011-1017. doi:10.1111/aogs.12454 |
| 416 | Zalel, Y., Zemet, R., & Kivilevitch, Z. (2017). The added value of detailed early anomaly scan in fetuses with increased nuchal translucency. Prenat Diagn, 37(3), 235-243. doi:10.1002/pd.4997 |
| 417 | Zanardini, C., Prefumo, F., Fichera, A., Botteri, E., & Frusca, T. (2014). Fetal cardiac parameters for prediction of twin-to-twin transfusion syndrome. Ultrasound Obstet Gynecol, 44(4), 434-440. doi:10.1002/uog.13442 |
| 418 | Zhong, Y., Longman, R., Bradshaw, R., & Odibo, A. O. (2011). The genetic sonogram: comparing the use of likelihood ratios versus logistic regression coefficients for Down syndrome screening. J Ultrasound Med, 30(4), 463-469. doi:10.7863/jum.2011.30.4.463 |
| 419 | Shaniba Asmi P, Subramaniam K, Iqbal NV, Venkatesan. Entropy based feature extraction of electrohysterogram signal for the prediction of preterm birth. Indian Journal of Public Health Research and Development 2018;9:1580-83. doi: https://doi.org/10.5958/0976-5506.2018.00756.8. |
| 420 | Carty, D. M., Siwy, J., Brennand, J. E., Zürbig, P., Mullen, W., Franke, J., . . . Delles, C. (2011). Urinary proteomics for prediction of preeclampsia. Hypertension, 57(3), 561-569. doi:10.1161/hypertensionaha.110.164285 |
| 421 | Cashen, K., Costello, J. M., Grimaldi, L. M., Narayana Gowda, K. M., Moser, E. A. S., Piggott, K. D., . . . Mastropietro, C. W. (2018). Multicenter Validation of the Vasoactive-Ventilation-Renal Score as a Predictor of Prolonged Mechanical Ventilation After Neonatal Cardiac Surgery. Pediatr Crit Care Med, 19(11), 1015-1023. doi:10.1097/pcc.0000000000001694 |
| 422 | Ghi, T., Youssef, A., Martelli, F., Bellussi, F., Aiello, E., Pilu, G., . . . Rizzo, G. (2016). Narrow subpubic arch angle is associated with higher risk of persistent occiput posterior position at delivery. Ultrasound Obstet Gynecol, 48(4), 511-515. doi:10.1002/uog.15808 |
| 423 | Ambalavanan, N., Carlo, W. A., Bobashev, G., Mathias, E., Liu, B., Poole, K., . . . Wright, L. L. (2005). Prediction of death for extremely low birth weight neonates. Pediatrics, 116(6), 1367-1373. doi:10.1542/peds.2004-2099 |
| 424 | Beksac, M. S., Tanacan, A., Bacak, H. O., & Leblebicioglu, K. (2018). Computerized prediction system for the route of delivery (vaginal birth versus cesarean section). J Perinat Med, 46(8), 881-884. doi:10.1515/jpm-2018-0022 |
| 425 | Bolón-Canedo, V., Ataer-Cansizoglu, E., Erdogmus, D., Kalpathy-Cramer, J., Fontenla-Romero, O., Alonso-Betanzos, A., & Chiang, M. F. (2015). Dealing with inter-expert variability in retinopathy of prematurity: A machine learning approach. Comput Methods Programs Biomed, 122(1), 1-15. doi:10.1016/j.cmpb.2015.06.004 |
| 426 | Demailly, R., Escolano, S., Haramburu, F., Tubert-Bitter, P., & Ahmed, I. Identifying Drugs Inducing Prematurity by Mining Claims Data with High-Dimensional Confounder Score Strategies. Drug Safety, 11. doi:10.1007/s40264-020-00916-5 |
| 427 | Devoe, L. D., Carlton, E., & Prescott, P. (1995). Neural network prediction of nonstress test results: how often should we perform nonstress tests? Am J Obstet Gynecol, 173(4), 1128-1131. doi:10.1016/0002-9378(95)91338-6 |
| 428 | Frick, A. P., Syngelaki, A., Zheng, M., Poon, L. C., & Nicolaides, K. H. (2016). Prediction of large-for-gestational-age neonates: screening by maternal factors and biomarkers in the three trimesters of pregnancy. Ultrasound Obstet Gynecol, 47(3), 332-339. doi:10.1002/uog.15780 |
| 429 | Bahado-Singh RO, Sonek J, McKenna D, et al. Artificial intelligence and amniotic fluid multiomics analysis: The prediction of perinatal outcome in asymptomatic short cervix. Ultrasound Obstet Gynecol 2018. doi: https://doi.org/10.1002/uog.20168. |
| 430 | Borowska M, Brzozowska E, Kuc P, Oczeretko E, Mosdorf R, Laudanski P. Identification of preterm birth based on rqa analysis of electrohysterograms. Comput Methods Programs Biomed 2018;153:227-36. doi: https://doi.org/10.1016/j.cmpb.2017.10.018. |
| 431 | Cai, M., Wang, Y. M., Luo, Q., & Wei, G. (2019). Factor Analysis of the Prediction of the Postpartum Depression Screening Scale. International Journal of Environmental Research and Public Health, 16(24), 13. doi:10.3390/ijerph16245025 |
| 432 | Caruana, R., Niculescu, R. S., Rao, R. B., & Simms, C. (2002). Machine learning for sub-population assessment: evaluating the C-section rate of different physician practices. Proc AMIA Symp, 126-130. |
| 433 | Courtney, K. L., Stewart, S., Popescu, M., & Goodwin, L. K. (2008). Predictors of preterm birth in birth certificate data. Stud Health Technol Inform, 136, 555-560. |
| 434 | Moreira MWL, Rodrigues JJPC, Marcondes GAB, Neto AJV, Kumar N, Diez IDLT. A preterm birth risk prediction system for mobile health applications based on the support vector machine algorithm. Year. doi: https://doi.org/10.1109/ICC.2018.8422616. |
| 435 | Moreira MWL, Rodrigues JJPC, Carvalho FHC, Chilamkurti N, Al-Muhtadi J, Denisov V. Biomedical data analytics in mobile-health environments for high-risk pregnancy outcome prediction. Journal of Ambient Intelligence and Humanized Computing 2019. doi: https://doi.org/10.1007/s12652-019-01230-4. |
| 436 | Moreira MWL, Rodrigues J, Kumar N, Saleem K, Illin IV. Postpartum depression prediction through pregnancy data analysis for emotion-aware smart systems updates. Information Fusion 2019;47:23-31. doi: https://doi.org/10.1016/j.inffus.2018.07.001. |
| 437 | Vullings R, Mischi M. Vectorcardiographic loop alignment for fetal movement detection using the expectation-maximization algorithm and support vector machines. Conf Proc IEEE Eng Med Biol Soc 2013;2013:2915-8. doi: https://doi.org/10.1109/embc.2013.6610150. |
| 438 | Yarlapati AR, Roy Dey S, Saha S. Early prediction of lbw cases via minimum error rate classifier: A statistical machine learning approach. Year. doi: https://doi.org/10.1109/SMARTCOMP.2017.7947002. |
| 439 | Adeyinka, D. A., Olakunde, B. O., & Muhajarine, N. (2019). Evidence of health inequity in child survival: spatial and Bayesian network analyses of stillbirth rates in 194 countries. Sci Rep, 9(1), 19755. doi:10.1038/s41598-019-56326-w |
| 440 | Anuwutnavin, S., Satou, G., Chang, R. K., DeVore, G. R., Abuel, A., & Sklansky, M. (2016). Prenatal Sonographic Predictors of Neonatal Coarctation of the Aorta. J Ultrasound Med, 35(11), 2353-2364. doi:10.7863/ultra.15.06049 |
| 441 | Assawapalanggool, S., Kasatpibal, N., Sirichotiyakul, S., Arora, R., & Suntornlimsiri, W. (2017). A Prognostic Scoring Tool for Cesarean Organ/Space Surgical Site Infections: Derivation and Internal Validation. Surg Infect (Larchmt), 18(6), 694-701. doi:10.1089/sur.2016.264 |
| 442 | Ben-Haroush, A., Farhi, J., Zahalka, Y., Sapir, O., Meizner, I., & Fisch, B. (2011). Small antral follicle count (2-5 mm) and ovarian volume for prediction of pregnancy in *in vitro* fertilization cycles. Gynecol Endocrinol, 27(10), 748-752. doi:10.3109/09513590.2010.526668 |
| 443 | Berger, R. P., Pak, B. J., Kolesnikova, M. D., Fromkin, J., Saladino, R., Herman, B. E., . . . Kochanek, P. M. (2017). Derivation and Validation of a Serum Biomarker Panel to Identify Infants With Acute Intracranial Hemorrhage. JAMA Pediatr, 171(6), e170429. doi:10.1001/jamapediatrics.2017.0429 |
| 444 | Bharatha, A., Faughnan, M. E., Kim, H., Pourmohamad, T., Krings, T., Bayrak-Toydemir, P., . . . Terbrugge, K. G. (2012). Brain arteriovenous malformation multiplicity predicts the diagnosis of hereditary hemorrhagic telangiectasia: quantitative assessment. Stroke, 43(1), 72-78. doi:10.1161/strokeaha.111.629865 |
| 445 | Brown, J. M., Campbell, J. P., Beers, A., Chang, K., Ostmo, S., Chan, R. V. P., . . . Chiang, M. F. (2018). Automated Diagnosis of Plus Disease in Retinopathy of Prematurity Using Deep Convolutional Neural Networks. JAMA Ophthalmol, 136(7), 803-810. doi:10.1001/jamaophthalmol.2018.1934 |
| 446 | Campbell, J. P., Ataer-Cansizoglu, E., Bolon-Canedo, V., Bozkurt, A., Erdogmus, D., Kalpathy-Cramer, J., . . . Chiang, M. F. (2016). Expert Diagnosis of Plus Disease in Retinopathy of Prematurity From Computer-Based Image Analysis. JAMA Ophthalmol, 134(6), 651-657. doi:10.1001/jamaophthalmol.2016.0611 |
| 447 | Chalam, K. V., Lin, S., Murthy, R. K., Brar, V. S., Gupta, S. K., & Radhakrishnan, R. (2011). Evaluation of modified retinopathy of prematurity screening guidelines using birth weight as the sole inclusion criterion. Middle East Afr J Ophthalmol, 18(3), 214-219. doi:10.4103/0974-9233.84048 |
| 448 | Chen, Z. Y., Liu, J. H., Liang, K., Liang, W. X., Ma, S. H., Zeng, G. J., . . . He, J. G. (2012). The diagnostic value of a multivariate logistic regression analysis model with transvaginal power Doppler ultrasonography for the prediction of ectopic pregnancy. J Int Med Res, 40(1), 184-193. doi:10.1177/147323001204000119 |
| 449 | Cheng, Y. C., Yan, G. L., Chiu, Y. H., Chang, F. M., Chang, C. H., & Chung, K. C. (2012). Efficient fetal size classification combined with artificial neural network for estimation of fetal weight. Taiwan J Obstet Gynecol, 51(4), 545-553. doi:10.1016/j.tjog.2012.09.009 |
| 450 | Cobo, T., Aldecoa, V., Figueras, F., Herranz, A., Ferrero, S., Izquierdo, M., . . . Palacio, M. (2020). Development and validation of a multivariable prediction model of spontaneous preterm delivery and microbial invasion of the amniotic cavity in women with preterm labor. Am J Obstet Gynecol. doi:10.1016/j.ajog.2020.02.049 |
| 451 | Do, Q. N., Lewis, M. A., Xi, Y., Madhuranthakam, A. J., Happe, S. K., Dashe, J. S., . . . Twickler, D. M. (2020). MRI of the Placenta Accreta Spectrum (PAS) Disorder: Radiomics Analysis Correlates With Surgical and Pathological Outcome. J Magn Reson Imaging, 51(3), 936-946. doi:10.1002/jmri.26883 |
| 452 | Du, X., Song, Y., Liu, Y., Zhang, Y., Liu, H., Chen, B., & Li, S. (2020). An integrated deep learning framework for joint segmentation of blood pool and myocardium. Med Image Anal, 62, 101685. doi:10.1016/j.media.2020.101685 |
| 453 | Ekizler, F. A., Cay, S., Kafes, H., Ozeke, O., Ozcan, F., Topaloglu, S., . . . Aras, D. (2019). The prognostic value of positive T wave in lead aVR: A novel marker of adverse cardiac outcomes in peripartum cardiomyopathy. Ann Noninvasive Electrocardiol, 24(3), e12631. doi:10.1111/anec.12631 |
| 454 | El-Haieg, D. O., Madkour, N. M., Basha, M. A. A., Ahmad, R. A., Sadek, S. M., Ibrahim, S. A., . . . Azmy, T. M. (2019). An Ultrasound Scoring Model for the Prediction of Intrapartum Morbidly Adherent Placenta and Maternal Morbidity: A Cross-Sectional Study. Ultraschall Med. doi:10.1055/a-0891-0772 |
| 455 | Fernández, L., Mediano, P., García, R., Rodríguez, J. M., & Marín, M. (2016). Risk Factors Predicting Infectious Lactational Mastitis: Decision Tree Approach versus Logistic Regression Analysis. Matern Child Health J, 20(9), 1895-1903. doi:10.1007/s10995-016-2000-6 |
| 456 | Fu, Q., & Lin, J. (2016). Predictive accuracy of three clinical risk assessment systems for cardiac complications among Chinese pregnant women with congenital heart disease. Int J Gynaecol Obstet, 134(2), 140-144. doi:10.1016/j.ijgo.2016.02.010 |
| 457 | Gadaras, I., & Mikhailov, L. (2009). An interpretable fuzzy rule-based classification methodology for medical diagnosis. Artif Intell Med, 47(1), 25-41. doi:10.1016/j.artmed.2009.05.003 |
| 458 | Gao, Y., Zhang, X., Xiong, S., Han, W., Liu, J., & Huang, G. (2015). Motile sperm organelle morphology examination (MSOME) can predict outcomes of conventional *in vitro* fertilization: A prospective pilot diagnostic study. Hum Fertil (Camb), 18(4), 258-264. doi:10.3109/14647273.2015.1072645 |
| 459 | Graham, E. M., Adami, R. R., McKenney, S. L., Jennings, J. M., Burd, I., & Witter, F. R. (2014). Diagnostic accuracy of fetal heart rate monitoring in the identification of neonatal encephalopathy. Obstet Gynecol, 124(3), 507-513. doi:10.1097/aog.0000000000000424 |
| 460 | Grobman, W. A., Lai, Y., Iams, J. D., Reddy, U. M., Mercer, B. M., Saade, G., . . . Caritis, S. N. (2016). Prediction of Spontaneous Preterm Birth Among Nulliparous Women With a Short Cervix. J Ultrasound Med, 35(6), 1293-1297. doi:10.7863/ultra.15.08035 |
| 461 | Grossi, E., Veggo, F., Narzisi, A., Compare, A., & Muratori, F. (2016). Pregnancy risk factors in autism: a pilot study with artificial neural networks. Pediatr Res, 79(2), 339-347. doi:10.1038/pr.2015.222 |
| 462 | Gutiérrez-Fragoso, K., Acosta-Mesa, H. G., Cruz-Ramírez, N., & Hernández-Jiménez, R. (2017). Optimization of Classification Strategies of Acetowhite Temporal Patterns towards Improving Diagnostic Performance of Colposcopy. Comput Math Methods Med, 2017, 5989105. doi:10.1155/2017/5989105 |
| 463 | Guzman-Barcenas, J., Hernandez, J. A., Arias-Martinez, J., Baptista-Gonzalez, H., Ceballos-Reyes, G., & Irles, C. (2016). Estimation of umbilical cord blood leptin and insulin based on anthropometric data by means of artificial neural network approach: identifying key maternal and neonatal factors. BMC Pregnancy Childbirth, 16, 11. doi:10.1186/s12884-016-0967-z |
| 464 | Hou, C. J., Wei, R., Tang, J. L., Hu, Q. H., He, H. F., & Fan, X. M. (2018). Diagnostic value of ultrasound features and sex of fetuses in female patients with papillary thyroid microcarcinoma. Sci Rep, 8(1), 7510. doi:10.1038/s41598-018-26003-5 |
| 465 | Hu, X., & Yu, Z. B. (2019). Diagnosis of mesothelioma with deep learning. Oncology Letters, 17(2), 1483-1490. doi:10.3892/ol.2018.9761 |
| 466 | Irles, C., González-Pérez, G., Carrera Muiños, S., Michel Macias, C., Sánchez Gómez, C., Martínez-Zepeda, A., . . . Laresgoiti Servitje, E. (2018). Estimation of Neonatal Intestinal Perforation Associated with Necrotizing Enterocolitis by Machine Learning Reveals New Key Factors. Int J Environ Res Public Health, 15(11). doi:10.3390/ijerph15112509 |
| 467 | Kang, W. D., Choi, H. S., & Kim, S. M. (2012). Prediction of persistent gestational trophobalstic neoplasia: the role of hCG level and ratio in 2 weeks after evacuation of complete mole. Gynecol Oncol, 124(2), 250-253. doi:10.1016/j.ygyno.2011.10.035 |
| 468 | Kebapcilar, L., Kebapcilar, A. G., Ilhan, T. T., Ipekci, S. H., Baldane, S., Pekin, A., . . . Celik, C. (2016). Is the Mean Platelet Volume a Predictive Marker of a Low Apgar Score and Insulin Resistance in Gestational Diabetes Mellitus? A Retrospective Case-Control Study. J Clin Diagn Res, 10(10), Oc06-oc10. doi:10.7860/jcdr/2016/20874.8611 |
| 469 | Kim, J. I., Park, I. Y., Yim, J. M., Cheon, J. Y., Yun, H. G., & Kwon, J. Y. (2017). Serum β-hCG concentration is a predictive factor for successful early medical abortion with vaginal misoprostol within 24 hours. Obstet Gynecol Sci, 60(5), 427-432. doi:10.5468/ogs.2017.60.5.427 |
| 470 | Kim, M. A., Han, G. H., & Kim, Y. H. (2019). Prediction of small-for-gestational age by fetal growth rate according to gestational age. PLoS One, 14(4), e0215737. doi:10.1371/journal.pone.0215737 |
| 471 | Kulan, H., & Dag, T. (2019). In silico identification of critical proteins associated with learning process and immune system for Down syndrome. PLoS One, 14(1), e0210954. doi:10.1371/journal.pone.0210954 |
| 472 | Kuwata, S., Suehiro, K., Juri, T., Tsujimoto, S., Mukai, A., Tanaka, K., . . . Nishikawa, K. (2018). Pleth variability index can predict spinal anaesthesia-induced hypotension in patients undergoing caesarean delivery. Acta Anaesthesiol Scand, 62(1), 75-84. doi:10.1111/aas.13012 |
| 473 | Labenne, M., Lizard, G., Ferdynus, C., Montange, T., Iacobelli, S., Bonsante, F., & Gouyon, J. B. (2011). A clinic-biological score for diagnosing early-onset neonatal infection in critically ill preterm infants. Pediatr Crit Care Med, 12(2), 203-209. doi:10.1097/PCC.0b013e3181e2a53b |
| 474 | Lédée, N., Munaut, C., Sérazin, V., Perrier d'Hauterive, S., Lombardelli, L., Logiodice, F., . . . Piccinni, M. P. (2010). Performance evaluation of microbead and ELISA assays for follicular G-CSF: a non-invasive biomarker of oocyte developmental competence for embryo implantation. J Reprod Immunol, 86(2), 126-132. doi:10.1016/j.jri.2010.05.003 |
| 475 | Lemmens, L., Kos, S., Beijer, C., Brinkman, J. W., van der Horst, F. A., van den Hoven, L., . . . Wetzels, A. M. (2016). Predictive value of sperm morphology and progressively motile sperm count for pregnancy outcomes in intrauterine insemination. Fertil Steril, 105(6), 1462-1468. doi:10.1016/j.fertnstert.2016.02.012 |
| 476 | Li, W. P., Neradilek, M. B., Gu, F. S., Isquith, D. A., Sun, Z. J., Wu, X., . . . Zhao, X. Q. (2017). Pregnancy-associated plasma protein-A is a stronger predictor for adverse cardiovascular outcomes after acute coronary syndrome in type-2 diabetes mellitus. Cardiovasc Diabetol, 16(1), 45. doi:10.1186/s12933-017-0526-6 |
| 477 | Litwińska, E., Litwińska, M., Oszukowski, P., Szaflik, K., & Kaczmarek, P. (2017). Combined screening for early and late pre-eclampsia and intrauterine growth restriction by maternal history, uterine artery Doppler, mean arterial pressure and biochemical markers. Adv Clin Exp Med, 26(3), 439-448. doi:10.17219/acem/62214 |
| 478 | Ludwin, A., Ludwin, I., Pityński, K., Banas, T., & Jach, R. (2014). Role of morphologic characteristics of the uterine septum in the prediction and prevention of abnormal healing outcomes after hysteroscopic metroplasty. Hum Reprod, 29(7), 1420-1431. doi:10.1093/humrep/deu110 |
| 479 | Melamed, N., Hiersch, L., Meizner, I., Bardin, R., Wiznitzer, A., & Yogev, Y. (2014). Is measurement of cervical length an accurate predictive tool in women with a history of preterm delivery who present with threatened preterm labor? Ultrasound Obstet Gynecol, 44(6), 661-668. doi:10.1002/uog.13395 |
| 480 | Miao, J. H., & Miao, K. H. (2018). Cardiotocographic diagnosis of fetal health based on multiclass morphologic pattern predictions using deep learning classification. International Journal of Advanced Computer Science and Applications, 9(5), 1-11. doi:10.14569/IJACSA.2018.090501 |
| 481 | Mumusoglu, S., Yarali, I., Bozdag, G., Ozdemir, P., Polat, M., Sokmensuer, L. K., & Yarali, H. (2017). Time-lapse morphokinetic assessment has low to moderate ability to predict euploidy when patient- and ovarian stimulation-related factors are taken into account with the use of clustered data analysis. Fertil Steril, 107(2), 413-421.e414. doi:10.1016/j.fertnstert.2016.11.005 |
| 482 | Nadim, B., Leonardi, M., Infante, F., Lattouf, I., Reid, S., & Condous, G. (2020). Rationalizing the management of pregnancies of unknown location: Diagnostic accuracy of human chorionic gonadotropin ratio-based decision tree compared with the risk prediction model M4. Acta Obstet Gynecol Scand, 99(3), 381-390. doi:10.1111/aogs.13752 |
| 483 | Namburete, A. I., Stebbing, R. V., Kemp, B., Yaqub, M., Papageorghiou, A. T., & Alison Noble, J. (2015). Learning-based prediction of gestational age from ultrasound images of the fetal brain. Med Image Anal, 21(1), 72-86. doi:10.1016/j.media.2014.12.006 |
| 484 | Nombo, A. P., Mwanri, A. W., Brouwer-Brolsma, E. M., Ramaiya, K. L., & Feskens, E. J. M. (2018). Gestational diabetes mellitus risk score: A practical tool to predict gestational diabetes mellitus risk in Tanzania. Diabetes Res Clin Pract, 145, 130-137. doi:10.1016/j.diabres.2018.05.001 |
| 485 | Osmanağaoğlu, M. A., Karahan, S. C., Aran, T., Güven, S., Turgut, E., Menteşe, A., & Bozkaya, H. (2014). The Diagnostic Value of β-Human Chorionic Gonadotropin, Progesterone, and Ischemia-Modified Albumin and Their Combined Use in the Prediction of First Trimester Abortions. Int Sch Res Notices, 2014, 846531. doi:10.1155/2014/846531 |
| 486 | Özdemir, M. E., Telatar, Z., Eroğul, O., & Tunca, Y. (2018). Classifying dysmorphic syndromes by using artificial neural network based hierarchical decision tree. Australas Phys Eng Sci Med, 41(2), 451-461. doi:10.1007/s13246-018-0643-x |
| 487 | Peled, Y., Ben-Haroush, A., Eitan, R., Eiger, M., Pardo, J., & Krissi, H. (2011). The accuracy of the preoperative diagnosis in women undergoing emergent gynecological laparoscopy for acute abdominal pain. Arch Gynecol Obstet, 284(6), 1439-1442. doi:10.1007/s00404-010-1835-0 |
| 488 | Pereira, N., Elias, R. T., Christos, P. J., Petrini, A. C., Hancock, K., Lekovich, J. P., & Rosenwaks, Z. (2017). Supraphysiologic estradiol is an independent predictor of low birth weight in full-term singletons born after fresh embryo transfer. Hum Reprod, 32(7), 1410-1417. doi:10.1093/humrep/dex095 |
| 489 | Pisani, F., Facini, C., Pelosi, A., Mazzotta, S., Spagnoli, C., & Pavlidis, E. (2016). Neonatal seizures in preterm newborns: A predictive model for outcome. Eur J Paediatr Neurol, 20(2), 243-251. doi:10.1016/j.ejpn.2015.12.007 |
| 490 | Pomorski, M., Fuchs, T., & Zimmer, M. (2014). Prediction of uterine dehiscence using ultrasonographic parameters of cesarean section scar in the nonpregnant uterus: a prospective observational study. BMC Pregnancy Childbirth, 14, 365. doi:10.1186/s12884-014-0365-3 |
| 491 | Porcelli, P. J., & Rosenbloom, S. T. (2014). Comparison of new modeling methods for postnatal weight in ELBW infants using prenatal and postnatal data. J Pediatr Gastroenterol Nutr, 59(1), e2-8. doi:10.1097/mpg.0000000000000342 |
| 492 | Quantin, C., Benzenine, E., Ferdynus, C., Sediki, M., Auverlot, B., Abrahamowicz, M., . . . Sagot, P. (2013). Advantages and limitations of using national administrative data on obstetric blood transfusions to estimate the frequency of obstetric hemorrhages. J Public Health (Oxf), 35(1), 147-156. doi:10.1093/pubmed/fds057 |
| 493 | Rad, R. M., Saeedi, P., Au, J., & Havelock, J. (2019). Cell-Net: Embryonic Cell Counting and Centroid Localization via Residual Incremental Atrous Pyramid and Progressive Upsampling Convolution. IEEE Access, 7, 81945-81955. doi:10.1109/access.2019.2920933 |
| 494 | Raihan-Al-Masud, M., & Mondal, M. R. H. (2020). Data-driven diagnosis of spinal abnormalities using feature selection and machine learning algorithms. PLoS One, 15(2), e0228422. doi:10.1371/journal.pone.0228422 |
| 495 | Rizzo, G., Aiello, E., Pietrolucci, M. E., & Arduini, D. (2016). Ultrasonographic assessment of cervical length in pregnancies scheduled for a cesarean delivery: prediction of early spontaneous onset of labor. J Perinat Med, 44(7), 807-811. doi:10.1515/jpm-2015-0238 |
| 496 | Romero-Ruiz, A., Avendaño, M. S., Dominguez, F., Lozoya, T., Molina-Abril, H., Sangiao-Alvarellos, S., . . . Tena-Sempere, M. (2019). Deregulation of miR-324/KISS1/kisspeptin in early ectopic pregnancy: mechanistic findings with clinical and diagnostic implications. Am J Obstet Gynecol, 220(5), 480.e481-480.e417. doi:10.1016/j.ajog.2019.01.228 |
| 497 | Rosenbloom, J. I., Tuuli, M. G., Stout, M. J., Young, O. M., Woolfolk, C. L., López, J. D., . . . Cahill, A. G. (2019). A Prediction Model for Severe Maternal Morbidity in Laboring Patients at Term. Am J Perinatol, 36(1), 8-14. doi:10.1055/s-0038-1626716 |
| 498 | Rubio, A., Epiard, C., Gebus, M., Deiber, M., Samperiz, S., Genty, C., . . . Debillon, T. (2017). Diagnosis Accuracy of Transcutaneous Bilirubinometry in Very Preterm Newborns. Neonatology, 111(1), 1-7. doi:10.1159/000447736 |
| 499 | Ruiz, V. M., Saenz, L., Lopez-Magallon, A., Shields, A., Ogoe, H. A., Suresh, S., . . . Tsui, F. R. (2019). Early prediction of critical events for infants with single-ventricle physiology in critical care using routinely collected data. J Thorac Cardiovasc Surg, 158(1), 234-243.e233. doi:10.1016/j.jtcvs.2019.01.130 |
| 500 | Siristatidis, C., Vogiatzi, P., Pouliakis, A., Trivella, M., Papantoniou, N., & Bettocchi, S. (2016). Predicting IVF Outcome: A Proposed Web-based System Using Artificial Intelligence. In Vivo, 30(4), 507-512. |
| 501 | Sittiparn, W., & Siwadune, T. (2017). Risk Score for Prediction of Postpartum Hemorrhages in Normal Labor at Chonburi Hospital. J Med Assoc Thai, 100(4), 382-388. |
| 502 | Slaughter, L. A., Bonfante-Mejia, E., Hintz, S. R., Dvorchik, I., & Parikh, N. A. (2016). Early Conventional MRI for Prediction of Neurodevelopmental Impairment in Extremely-Low-Birth-Weight Infants. Neonatology, 110(1), 47-54. doi:10.1159/000444179 |
| 503 | Solis-Paredes, M., Estrada-Gutierrez, G., Perichart-Perera, O., Montoya-Estrada, A., Guzmán-Huerta, M., Borboa-Olivares, H., . . . Irles, C. (2017). Key Clinical Factors Predicting Adipokine and Oxidative Stress Marker Concentrations among Normal, Overweight and Obese Pregnant Women Using Artificial Neural Networks. Int J Mol Sci, 19(1). doi:10.3390/ijms19010086 |
| 504 | Stanfield, Z., Johnson, M. R., Blanks, A. M., Romero, R., Chance, M. R., Mesiano, S., & Koyuturkm, M. (2019). Myometrial Transcriptional Signatures of Human Parturition. Frontiers in Genetics, 10, 21. doi:10.3389/fgene.2019.00185 |
| 505 | Thompson, W. R., Reinisch, A. J., Unterberger, M. J., & Schriefl, A. J. (2019). Artificial Intelligence-Assisted Auscultation of Heart Murmurs: Validation by Virtual Clinical Trial. Pediatr Cardiol, 40(3), 623-629. doi:10.1007/s00246-018-2036-z |
| 506 | Van Belle, V. M., Van Calster, B., Timmerman, D., Bourne, T., Bottomley, C., Valentin, L., . . . Boyd, S. (2012). A mathematical model for interpretable clinical decision support with applications in gynecology. PLoS One, 7(3), e34312. doi:10.1371/journal.pone.0034312 |
| 507 | Vigdor, B., & Lerner, B. (2006). Accurate and fast off and online fuzzy ARTMAP-based image classification with application to genetic abnormality diagnosis. IEEE Trans Neural Netw, 17(5), 1288-1300. doi:10.1109/tnn.2006.877532 |
| 508 | Vilhena, J., Rosário Martins, M., Vicente, H., Grañeda, J. M., Caldeira, F., Gusmão, R., . . . Neves, J. (2017). An Integrated Soft Computing Approach to Hughes Syndrome Risk Assessment. J Med Syst, 41(3), 40. doi:10.1007/s10916-017-0688-5 |
| 509 | Vollmar, T., Maus, B., Wurtz, R. P., Gillessen-Kaesbach, G., Horsthemke, B., Wieczorek, D., & Boehringer, S. (2008). Impact of geometry and viewing angle on classification accuracy of 2D based analysis of dysmorphic faces. Eur J Med Genet, 51(1), 44-53. doi:10.1016/j.ejmg.2007.10.002 |
| 510 | Wang, Y., Wang, Z., & Zhang, H. (2018). Identification of diagnostic biomarker in patients with gestational diabetes mellitus based on transcriptome-wide gene expression and pattern recognition. J Cell Biochem. doi:10.1002/jcb.27279 |
| 511 | Wen, W. H., Huang, C. W., Chie, W. C., Yeung, C. Y., Zhao, L. L., Lin, W. T., . . . Chen, H. L. (2016). Quantitative maternal hepatitis B surface antigen predicts maternally transmitted hepatitis B virus infection. Hepatology, 64(5), 1451-1461. doi:10.1002/hep.28589 |
| 512 | Yafi, F. A., & Zini, A. (2013). Percutaneous epididymal sperm aspiration for men with obstructive azoospermia: predictors of successful sperm retrieval. Urology, 82(2), 341-344. doi:10.1016/j.urology.2013.04.014 |
| 513 | Yang, J. F., Ding, X. F., & Zhu, W. D. (2018). Improving the calling of non-invasive prenatal testing on 13-/18-/21-trisomy by support vector machine discrimination. PLoS One, 13(12), 19. doi:10.1371/journal.pone.0207840 |
| 514 | Yaqub, M., Javaid, M. K., Cooper, C., & Noble, J. A. (2014). Investigation of the role of feature selection and weighted voting in random forests for 3-D volumetric segmentation. IEEE Trans Med Imaging, 33(2), 258-271. doi:10.1109/tmi.2013.2284025 |
| 515 | Ye, Z., Ai, X., Zheng, J., Hu, X., You, C., Andrew, M. F., & Fang, F. (2019). Extravasation of contrast (Spot Sign) predicts in-hospital mortality in ruptured arteriovenous malformation. Br J Neurosurg, 33(2), 149-155. doi:10.1080/02688697.2017.1384792 |
| 516 | Ying, G. S., VanderVeen, D., Daniel, E., Quinn, G. E., & Baumritter, A. (2016). Risk Score for Predicting Treatment-Requiring Retinopathy of Prematurity (ROP) in the Telemedicine Approaches to Evaluating Acute-Phase ROP Study. Ophthalmology, 123(10), 2176-2182. doi:10.1016/j.ophtha.2016.06.037 |
| 517 | Younis, J. S., Jadaon, J., Izhaki, I., Haddad, S., Radin, O., Bar-Ami, S., & Ben-Ami, M. (2010). A simple multivariate score could predict ovarian reserve, as well as pregnancy rate, in infertile women. Fertil Steril, 94(2), 655-661. doi:10.1016/j.fertnstert.2009.03.036 |
| 518 | Yu, J., Wang, Y., & Chen, P. (2008). Fetal ultrasound image segmentation system and its use in fetal weight estimation. Med Biol Eng Comput, 46(12), 1227-1237. doi:10.1007/s11517-008-0407-y |
| 519 | Yu, J. H., Wang, Y. Y., & Chen, P. (2009). Fetal Weight Estimation Using the Evolutionary Fuzzy Support Vector Regression for Low-Birth-Weight Fetuses. IEEE Transactions on Information Technology in Biomedicine, 13(1), 57-66. doi:10.1109/titb.2008.2007080 |
| 520 | Yu, L., Guo, Y., Wang, Y., Yu, J., & Chen, P. (2017). Determination of Fetal Left Ventricular Volume Based on Two-Dimensional Echocardiography. J Healthc Eng, 2017, 4797315. doi:10.1155/2017/4797315 |
| 521 | Zamora, I. J., Olutoye, O. O., Cass, D. L., Fallon, S. C., Lazar, D. A., Cassady, C. I., . . . Lee, T. C. (2014). Prenatal MRI fetal lung volumes and percent liver herniation predict pulmonary morbidity in congenital diaphragmatic hernia (CDH). J Pediatr Surg, 49(5), 688-693. doi:10.1016/j.jpedsurg.2014.02.048 |
| 522 | Zandbaaf, S., Khanmohammadi Khorrami, M. R., Bagheri Garmarudi, A., & Hossein Rashidi, B. (2020). Diagnosis of pregnancy based classification of embryo culture medium samples by infrared spectrometry and chemometrics. Infrared Physics and Technology, 104. doi:10.1016/j.infrared.2019.103069 |
| 523 | Zhang, Y., Ma, J. K., Wei, H., Li, X. W., Li, L. Q., & Yu, J. L. (2016). Predictive scores for mortality in full-term infants with necrotizing enterocolitis: experience of a tertiary hospital in Southwest China. World J Pediatr, 12(2), 202-208. doi:10.1007/s12519-015-0063-x |
| 524 | Zhao, Z. D., Zhang, Y., Comert, Z. E., & Deng, Y. J. (2019). Computer-Aided Diagnosis System of Fetal Hypoxia Incorporating Recurrence Plot With Convolutional Neural Network. Frontiers in Physiology, 10, 14. doi:10.3389/fphys.2019.00255 |
| 525 | Zhong, Q. Y., Mittal, L. P., Nathan, M. D., Brown, K. M., Knudson González, D., Cai, T., . . . Williams, M. A. (2019). Use of natural language processing in electronic medical records to identify pregnant women with suicidal behavior: towards a solution to the complex classification problem. Eur J Epidemiol, 34(2), 153-162. doi:10.1007/s10654-018-0470-0 |
| 526 | Caballero-Ruiz E, Garcia-Saez G, Rigla M, Villaplana M, Pons B, Hernando ME. Automatic classification of glycaemia measurements to enhance data interpretation in an expert system for gestational diabetes. Expert Systems with Applications 2016;63:386-96. doi: https://doi.org/10.1016/j.eswa.2016.07.019. |
| 527 | De Carli MM, Baccarelli AA, Trevisi L, et al. Epigenome-wide cross-tissue predictive modeling and comparison of cord blood and placental methylation in a birth cohort. Epigenomics 2017;9:231-40. doi: https://doi.org/10.2217/epi-2016-0109. |
| 528 | Gentillon H, Stefanczyk L, Strzelecki M, Respondek-Liberska M. Texture analysis of the developing human brain using customization of a knowledge-based system. F1000Research 2017;6. doi: https://doi.org/10.12688/f1000research.10401.1. |
| 529 | González-Recio O, Forni S. Genome-wide prediction of discrete traits using bayesian regressions and machine learning. Genetics, selection, evolution : GSE 2011;43:7. doi: https://doi.org/10.1186/1297-9686-43-7. |
| 530 | Khan SR, Mohan H, Liu Y, et al. The discovery of novel predictive biomarkers and early-stage pathophysiology for the transition from gestational diabetes to type 2 diabetes. Diabetologia 2019;62:687-703. doi: https://doi.org/10.1007/s00125-018-4800-2. |
| 531 | Lin HC, Su CT, Wang PC. An application of artificial immune recognition system for prediction of diabetes following gestational diabetes. Journal of Medical Systems 2011;35:283-89. doi: https://doi.org/10.1007/s10916-009-9364-8. |
| 532 | Manna C, Nanni L, Lumini A, Pappalardo S. Artificial intelligence techniques for embryo and oocyte classification. Reproductive Biomedicine Online 2013;26:42-49. doi: https://doi.org/10.1016/j.rbmo.2012.09.015. |
| 533 | Maraci MA, Bridge CP, Napolitano R, Papageorghiou A, Noble JA. A framework for analysis of linear ultrasound videos to detect fetal presentation and heartbeat. Medical Image Analysis 2017;37:22-36. doi: https://doi.org/10.1016/j.media.2017.01.003. |
| 534 | Naimi AI, Platt RW, Larkin JC. Machine learning for fetal growth prediction. Epidemiology 2018;29:290-98. doi: https://doi.org/10.1097/ede.0000000000000788. |
| 535 | Orlandi S, Reyes Garcia CA, Bandini A, Donzelli G, Manfredi C. Application of pattern recognition techniques to the classification of full-term and preterm infant cry. J Voice 2016;30:656-63. doi: https://doi.org/10.1016/j.jvoice.2015.08.007. |
| 536 | Papageorghiou AT, Kemp B, Stones W, et al. Ultrasound-based gestational-age estimation in late pregnancy. Ultrasound Obstet Gynecol 2016;48:719-26. doi: https://doi.org/10.1002/uog.15894. |
| 537 | Rittenhouse KJ, Vwalika B, Keil A, et al. Improving preterm newborn identification in low-resource settings with machine learning. PLoS ONE 2019;14. doi: https://doi.org/10.1371/journal.pone.0198919. |
| 538 | Rodriguez LM, Fushman DD. Automatic classification of structured product labels for pregnancy risk drug categories, a machine learning approach. AMIA Annu Symp Proc 2015;2015:1093-102. doi, PMID: https://www.ncbi.nlm.nih.gov/pubmed/26958248. |
| 539 | Sarker A, Chandrashekar P, Magge A, Cai H, Klein A, Gonzalez G. Discovering cohorts of pregnant women from social media for safety surveillance and analysis. Journal of medical Internet research 2017;19:e361. doi: https://doi.org/10.2196/jmir.8164. |
| 540 | Sridar P, Kumar A, Quinton A, Nanan R, Kim J, Krishnakumar R. Decision fusion-based fetal ultrasound image plane classification using convolutional neural networks. Ultrasound in Medicine and Biology 2019;45:1259-73. doi: https://doi.org/10.1016/j.ultrasmedbio.2018.11.016. |
| 541 | Tylee DS, Kikinis Z, Quinn TP, et al. Machine-learning classification of 22q11.2 deletion syndrome: A diffusion tensor imaging study. Neuroimage Clin 2017;15:832-42. doi: https://doi.org/10.1016/j.nicl.2017.04.029. |
| 542 | Volk M, Maver A, Lovrečić L, Juvan P, Peterlin B. Expression signature as a biomarker for prenatal diagnosis of trisomy 21. PLoS ONE 2013;8. doi: https://doi.org/10.1371/journal.pone.0074184. |
| 543 | Yilmaz E, Kilikcier C. Determination of fetal state from cardiotocogram using ls-svm with particle swarm optimization and binary decision tree. Comput Math Methods Med 2013;2013:487179. doi: https://doi.org/10.1155/2013/487179. |
| 544 | Akhtar, F., Li, J., Pei, Y., Imran, A., Rajput, A., Azeem, M., & Wang, Q. (2019). Diagnosis and prediction of Large-for-Gestational-Age fetus using the stacked generalization method. Applied Sciences (Switzerland), 9(20). doi:10.3390/app9204317 |
| 545 | Alonso-Betanzos, A., Mosqueira-Rey, E., Moret-Bonillo, V., & Baldonedo del Río, B. (1999). Applying statistical, uncertainty-based and connectionist approaches to the prediction of fetal outcome: a comparative study. Artif Intell Med, 17(1), 37-57. doi:10.1016/s0933-3657(99)00013-5 |
| 546 | Ayachi, A., Bouchahda, R., Derouich, S., Mkaouer, L., Kehila, M., Abouda, H., . . . Mourali, M. (2018). Accuracy of preoperative real-time dynamic transvaginal ultrasound sliding sign in prediction of pelvic adhesions in women with previous abdominopelvic surgery: prospective, multicenter, double-blind study. Ultrasound in Obstetrics & Gynecology, 51(2), 253-258. doi:10.1002/uog.17465 |
| 547 | Broeze, K. A., Opmeer, B. C., Coppus, S. F., Van Geloven, N., Den Hartog, J. E., Land, J. A., . . . Mol, B. W. (2012). Integration of patient characteristics and the results of Chlamydia antibody testing and hysterosalpingography in the diagnosis of tubal pathology: an individual patient data meta-analysis. Hum Reprod, 27(10), 2979-2990. doi:10.1093/humrep/des281 |
| 548 | Chiogna, M., Spiegelhalter, D. J., Franklin, R. C., & Bull, K. (1996). An empirical comparison of expert-derived and data-derived classification trees. Stat Med, 15(2), 157-169. doi:10.1002/(sici)1097-0258(19960130)15:2<157::Aid-sim149>3.0.Co;2-5 |
| 549 | Mantini, D., Alleva, G., & Comani, S. (2005). A method for the automatic reconstruction of fetal cardiac signals from magnetocardiographic recordings. Phys Med Biol, 50(20), 4763-4781. doi:10.1088/0031-9155/50/20/002 |
| 550 | Ahmed F, Shams MMB, Shill PC, Rahman M. Classification on bdhs data analysis: Hybrid approach for predicting pregnancy termination. Year. doi: https://doi.org/10.1109/ECACE.2019.8679302. |
| 551 | Moreira MWL, Rodrigues JJPC, Al-Muhtadi J, Korotaev VV, de Albuquerque VHC. Neuro-fuzzy model for hellp syndrome prediction in mobile cloud computing environments. Concurrency Computation 2018. doi: https://doi.org/10.1002/cpe.4651. |
| 552 | Akhavan, S., Lak, P., Rahimi-Sharbaf, F., Mohammadi, S. R., & Shirazi, M. (2017). Admission Test and Pregnancy Outcome. Iran J Med Sci, 42(4), 362-368. |
| 553 | Alsayyari, A. (2019). Fetal cardiotocography monitoring using Legendre neural networks. Biomed Tech (Berl), 64(6), 669-675. doi:10.1515/bmt-2018-0074 |
| 554 | Comani, S., Srinivasan, V., Alleva, G., & Romani, G. L. (2007). Entropy-based automated classification of independent components separated from fMCG. Phys Med Biol, 52(5), N87-97. doi:10.1088/0031-9155/52/5/n02 |
| 555 | Dietz, H. P., & Kirby, A. (2010). Modelling the likelihood of levator avulsion in a urogynaecological population. Aust N Z J Obstet Gynaecol, 50(3), 268-272. doi:10.1111/j.1479-828X.2010.01157.x |
| 556 | Du, Q. Y., Wang, E. Y., Huang, Y., Guo, X. Y., Xiong, Y. J., Yu, Y. P., . . . Sun, Y. P. (2016). Blastocoele expansion degree predicts live birth after single blastocyst transfer for fresh and vitrified/warmed single blastocyst transfer cycles. Fertil Steril, 105(4), 910-919.e911. doi:10.1016/j.fertnstert.2015.12.014 |
| 557 | Elson, J., Tailor, A., Banerjee, S., Salim, R., Hillaby, K., & Jurkovic, D. (2004). Expectant management of tubal ectopic pregnancy: prediction of successful outcome using decision tree analysis. Ultrasound Obstet Gynecol, 23(6), 552-556. doi:10.1002/uog.1061 |
| 558 | Elson, J., Tailor, A., Salim, R., Hillaby, K., Dew, T., & Jurkovic, D. (2005). Expectant management of miscarriage--prediction of outcome using ultrasound and novel biochemical markers. Hum Reprod, 20(8), 2330-2333. doi:10.1093/humrep/dei038 |
| 559 | Fruscalzo, A., Londero, A. P., Calcagno, A., Cipriani, I., Bertozzi, S., Marchesoni, D., & Driul, L. (2015). Building a Prediction Model for Vacuum-Assisted Operative Vaginal Delivery Risk. Gynecol Obstet Invest, 80(4), 246-252. doi:10.1159/000381544 |
| 560 | Gasse, C., Boutin, A., Demers, S., Chaillet, N., & Bujold, E. (2019). Body mass index and the risk of hypertensive disorders of pregnancy: the great obstetrical syndromes (GOS) study. J Matern Fetal Neonatal Med, 32(7), 1063-1068. doi:10.1080/14767058.2017.1399117 |
| 561 | Guzmán-Huerta, M. E., Muro-Barragán, S. A., Acevedo-Gallegos, S., Velázquez-Torres, B., Gallardo-Gaona, J. M., Ramírez-Calvo, J. A., . . . Aguinaga-Rios, M. (2013). Amniotic band sequence: Prenatal diagnosis, phenotype descriptions, and a proposal of a new classification based on morphologic findings. Revista de Investigacion Clinica, 65(4), 300-306. |
| 562 | Kondo, M., Nagao, Y., Mahbub, M. H., Tanabe, T., & Tanizawa, Y. (2018). Factors predicting early postpartum glucose intolerance in Japanese women with gestational diabetes mellitus: decision-curve analysis. Diabet Med, 35(8), 1111-1117. doi:10.1111/dme.13657 |
| 563 | Leão Bde, F., Guazzelli, A., & Mendonça, E. A. (1994). HYCONES II: a tool to build hybrid connectionist expert systems. Proc Annu Symp Comput Appl Med Care, 747-751. |
| 564 | Lee, T. J., Rolnik, D. L., Menezes, M. A., McLennan, A. C., & da Silva Costa, F. (2018). Cell-free fetal DNA testing in singleton IVF conceptions. Hum Reprod, 33(4), 572-578. doi:10.1093/humrep/dey033 |
| 565 | Lim, S., & Kaiser, M. (2015). Developmental time windows for axon growth influence neuronal network topology. Biol Cybern, 109(2), 275-286. doi:10.1007/s00422-014-0641-3 |
| 566 | Liu, L., Hu, J., Wang, N., Liu, Y., Wei, X., Gao, M., . . . Wen, D. (2020). A novel association of CCDC80 with gestational diabetes mellitus in pregnant women: a propensity score analysis from a case-control study. BMC Pregnancy Childbirth, 20(1), 53. doi:10.1186/s12884-020-2743-3 |
| 567 | Manuck, T. A., Stoddard, G. J., Fry, R. C., Esplin, M. S., & Varner, M. W. (2016). Nonresponse to 17-alpha hydroxyprogesterone caproate for recurrent spontaneous preterm birth prevention: clinical prediction and generation of a risk scoring system. Am J Obstet Gynecol, 215(5), 622.e621-622.e628. doi:10.1016/j.ajog.2016.07.013 |
| 568 | Marvin, N., Bower, M., & Rowe, J. E. (1999). An evolutionary approach to constructing prognostic models. Artif Intell Med, 15(2), 155-165. doi:10.1016/s0933-3657(98)00050-5 |
| 569 | McPherson, K. C., Beggs, A. D., Sultan, A. H., & Thakar, R. (2014). Can the risk of obstetric anal sphincter injuries (OASIs) be predicted using a risk-scoring system? BMC Res Notes, 7, 471. doi:10.1186/1756-0500-7-471 |
| 570 | Milewska, A. J., Jankowska, D., Więsak, T., Acacio, B., & Milewski, R. (2017). The application of multinomial logistic regression models for the assessment of parameters of oocytes and embryos quality in predicting pregnancy and miscarriage. Studies in Logic, Grammar and Rhetoric, 51(1), 7-18. |
| 571 | Millischer, A. E., Salomon, L. J., Santulli, P., Borghese, B., Dousset, B., & Chapron, C. (2015). Fusion imaging for evaluation of deep infiltrating endometriosis: feasibility and preliminary results. Ultrasound in Obstetrics & Gynecology, 46(1), 109-117. doi:10.1002/uog.14712 |
| 572 | Monteith, C., McSweeney, L., Breatnach, C. R., Doherty, A., Shirren, L., Tully, E. C., . . . Kent, E. (2017). Non-invasive cardiac output monitoring (NICOM(®)) can predict the evolution of uteroplacental disease-Results of the prospective HANDLE study. Eur J Obstet Gynecol Reprod Biol, 216, 116-124. doi:10.1016/j.ejogrb.2017.07.018 |
| 573 | Murphy, M., Butler, M., Coughlan, B., Brennan, D., O'Herlihy, C., & Robson, M. (2015). Elevated amniotic fluid lactate predicts labor disorders and cesarean delivery in nulliparous women at term. Am J Obstet Gynecol, 213(5), 673.e671-678. doi:10.1016/j.ajog.2015.06.035 |
| 574 | Naito, K., Udagawa, J., & Otani, H. (2010). Multidimensional standard curve for the development process of human fetuses. Stat Med, 29(21), 2235-2245. doi:10.1002/sim.3952 |
| 575 | Oztas, E., Ozler, S., Ersoy, A. O., Iskender, C. T., Sucak, A., Ergin, M., . . . Danisman, N. (2016). Increased levels of serum clusterin is associated with intrauterine growth restriction and adverse pregnancy outcomes in preeclampsia. J Perinat Med, 44(3), 269-275. doi:10.1515/jpm-2015-0120 |
| 576 | Papadimitriou, S., Gatzounas, D., Papadopoulos, V., Tzigounis, V., & Bezerianos, A. (1997). Denoising of the fetal heart rate signal with non-linear filtering of the wavelet transform maxima. Int J Med Inform, 44(3), 177-192. doi:10.1016/s1386-5056(97)00019-1 |
| 577 | Papadimitriou, S., & Bezerianos, A. (1999). Nonlinear analysis of the performance and reliability of wavelet singularity detection based denoising for Doppler ultrasound fetal heart rate signals. Int J Med Inform, 53(1), 43-60. doi:10.1016/s1386-5056(98)00102-6 |
| 578 | Papanna, R., Mann, L. K., Baschat, A. A., Bebbington, M. W., Khalek, N., Johnson, A., . . . Moise, K. J., Jr. (2015). Cervical length in prediction of preterm birth after laser surgery for twin-twin transfusion syndrome. Ultrasound Obstet Gynecol, 45(2), 175-182. doi:10.1002/uog.14696 |
| 579 | Pare, E., Parry, S., McElrath, T. F., Pucci, D., Newton, A., & Lim, K. H. (2014). Clinical Risk Factors for Preeclampsia in the 21st Century. Obstetrics and Gynecology, 124(4), 763-770. doi:10.1097/aog.0000000000000451 |
| 580 | Parra-Saavedra, M., Crovetto, F., Triunfo, S., Savchev, S., Parra, G., Sanz, M., . . . Figueras, F. (2013). Added value of umbilical vein flow as a predictor of perinatal outcome in term small-for-gestational-age fetuses. Ultrasound Obstet Gynecol, 42(2), 189-195. doi:10.1002/uog.12380 |
| 581 | Payne, J. L., Osborne, L. M., Cox, O., Kelly, J., Meilman, S., Jones, I., . . . Kaminsky, Z. A. (2020). DNA methylation biomarkers prospectively predict both antenatal and postpartum depression. Psychiatry Res, 285, 112711. doi:10.1016/j.psychres.2019.112711 |
| 582 | Reed, N. E., Gini, M., Johnson, P. E., & Moller, J. H. (1997). Diagnosing congenital heart defects using the Fallot computational model. Artif Intell Med, 10(1), 25-40. doi:10.1016/s0933-3657(97)00382-5 |
| 583 | Ren, P., Yao, S., Li, J., Valdes-Sosa, P. A., & Kendrick, K. M. (2015). Improved prediction of preterm delivery using empirical mode decomposition analysis of uterine electromyography signals. PLoS One, 10(7). doi:10.1371/journal.pone.0132116 |
| 584 | Roberts, L. A., Ling, H. Z., Poon, L. C., Nicolaides, K. H., & Kametas, N. A. (2018). Maternal hemodynamics, fetal biometry and Doppler indices in pregnancies followed up for suspected fetal growth restriction. Ultrasound Obstet Gynecol, 52(4), 507-514. doi:10.1002/uog.19067 |
| 585 | Semenova, O., Carra, G., Lightbody, G., Boylan, G., Dempsey, E., & Temko, A. (2018). Heart Rate Variability during Periods of Low Blood Pressure as a Predictor of Short-Term Outcome in Preterms. Conf Proc IEEE Eng Med Biol Soc, 2018, 5614-5517. doi:10.1109/embc.2018.8513600 |
| 586 | Simon, L., Frondas-Chauty, A., Senterre, T., Flamant, C., Darmaun, D., & Rozé, J. C. (2014). Determinants of body composition in preterm infants at the time of hospital discharge. Am J Clin Nutr, 100(1), 98-104. doi:10.3945/ajcn.113.080945 |
| 587 | Sinclair, M., Baumgartner, C. F., Matthew, J., Bai, W., Martinez, J. C., Li, Y., . . . Rueckert, D. (2018). Human-level Performance On Automatic Head Biometrics In Fetal Ultrasound Using Fully Convolutional Neural Networks. Conf Proc IEEE Eng Med Biol Soc, 2018, 714-717. doi:10.1109/embc.2018.8512278 |
| 588 | Siristatidis, C., Pouliakis, A., Chrelias, C., & Kassanos, D. (2011). Artificial intelligence in IVF: a need. Syst Biol Reprod Med, 57(4), 179-185. doi:10.3109/19396368.2011.558607 |
| 589 | Smith, G. C., Seaman, S. R., Wood, A. M., Royston, P., & White, I. R. (2014). Correcting for optimistic prediction in small data sets. Am J Epidemiol, 180(3), 318-324. doi:10.1093/aje/kwu140 |
| 590 | Sur, S. D., Jayaprakasan, K., Jones, N. W., Clewes, J., Winter, B., Cash, N., . . . Raine-Fenning, N. J. (2010). A novel technique for the semi-automated measurement of embryo volume: an intraobserver reliability study. Ultrasound Med Biol, 36(5), 719-725. doi:10.1016/j.ultrasmedbio.2010.03.006 |
| 591 | Torgersen, S. (1979). The determination of twin zygosity by means of a mailed questionnaire. Acta Genet Med Gemellol (Roma), 28(3), 225-236. doi:10.1017/s0001566000009077 |
| 592 | Van Ravenswaaij, R., Tesselaar‐Van der Goot, M., de Wolf, S., van Leeuwen‐Spruijt, M., Visser, G., & Schielen, P. (2011). First‐trimester serum PAPP‐A and fβ‐hCG concentrations and other maternal characteristics to establish logistic regression‐based predictive rules for adverse pregnancy outcome. Prenat Diagn, 31(1), 50-57. |
| 593 | Velzel, J., Schuit, E., Vlemmix, F., Molkenboer, J. F. M., Van der Post, J. A. M., Mol, B. W., & Kok, M. (2018). Development and internal validation of a clinical prediction model for external cephalic version. Eur J Obstet Gynecol Reprod Biol, 228, 137-142. doi:10.1016/j.ejogrb.2018.06.019 |
| 594 | Wang, Y., Zhu, Q., Lin, F., Xie, L., Li, J., & Wang, X. (2019). Development and internal validation of a Nomogram for preoperative prediction of surgical treatment effect on cesarean section diverticulum. BMC Womens Health, 19(1), 136. doi:10.1186/s12905-019-0817-z |
| 595 | Webster, L. M., Bramham, K., Seed, P. T., Homsy, M., Widdows, K., Webb, A. J., . . . Chappell, L. C. (2019). Impact of ethnicity on adverse perinatal outcome in women with chronic hypertension: a cohort study. Ultrasound Obstet Gynecol, 54(1), 72-78. doi:10.1002/uog.20132 |
| 596 | Weiss, R. J., Bates, S. V., Song, Y., Zhang, Y., Herzberg, E. M., Chen, Y. C., . . . Ou, Y. (2019). Mining multi-site clinical data to develop machine learning MRI biomarkers: application to neonatal hypoxic ischemic encephalopathy. J Transl Med, 17(1), 385. doi:10.1186/s12967-019-2119-5 |
| 597 | Ye, K. J., Dai, J., Liu, L. Y., & Peng, M. J. (2018). Network‑based gene function inference method to predict optimal gene functions associated with fetal growth restriction. Mol Med Rep, 18(3), 3003-3010. doi:10.3892/mmr.2018.9232 |
| 598 | Zhang, K., Ma, H., Zhao, Y., Zan, H., & Zhuang, L. (2018). The Comparative Experimental Study of Multilabel Classification for Diagnosis Assistant Based on Chinese Obstetric EMRs. J Healthc Eng, 2018, 7273451. doi:10.1155/2018/7273451 |
| 599 | Zhu, B., Dunson, D. B., & Ashley-Koch, A. E. (2012). Adverse subpopulation regression for multivariate outcomes with high-dimensional predictors. Stat Med, 31(29), 4102-4113. doi:10.1002/sim.5520 |
| 600 | Dey A, Hay K, Afroz B, et al. Understanding intersections of social determinants of maternal healthcare utilization in uttar pradesh, india. PLoS One 2018;13:e0204810. doi: https://doi.org/10.1371/journal.pone.0204810. |
| 601 | Alansary, A., Oktay, O., Li, Y., Folgoc, L. L., Hou, B., Vaillant, G., . . . Rueckert, D. (2019). Evaluating reinforcement learning agents for anatomical landmark detection. Med Image Anal, 53, 156-164. doi:10.1016/j.media.2019.02.007 |
| 602 | Altin, C., Yilmaz, M., Ozsoy, H. M., Gezmis, E., Balci, S., Tekindal, M. A., . . . Muderrisoglu, H. (2018). Assessment of epicardial fat and carotid intima media thickness in gestational hypertension. J Obstet Gynaecol Res, 44(6), 1072-1079. doi:10.1111/jog.13631 |
| 603 | Alves, J. A. G., de Sousa, P. C. P., Moura, S., Kane, S. C., & Costa, F. D. (2014). First-trimester maternal ophthalmic artery Doppler analysis for prediction of pre-eclampsia. Ultrasound in Obstetrics & Gynecology, 44(4), 411-418. doi:10.1002/uog.13338 |
| 604 | Amabebe, E., Reynolds, S., He, X., Wood, R., Stern, V., & Anumba, D. O. C. (2019). Infection/inflammation-associated preterm delivery within 14 days of presentation with symptoms of preterm labour: A multivariate predictive model. PLoS One, 14(9), e0222455. doi:10.1371/journal.pone.0222455 |
| 605 | Arfi, A., Bendifallah, S., Mathieu D'argent, E., Poupon, C., Ballester, M., Cohen, J., & Darai, E. (2019). Nomogram predicting the likelihood of live-birth rate after surgery for deep infiltrating endometriosis without bowel involvement in women who wish to conceive: A retrospective study. Eur J Obstet Gynecol Reprod Biol, 235, 81-87. doi:10.1016/j.ejogrb.2019.02.007 |
| 606 | Beksaç, M. S., Başaran, F., Eskiizmirliler, S., Erkmen, A. M., & Yörükan, S. (1996). A computerized diagnostic system for the interpretation of umbilical artery blood flow velocity waveforms. Eur J Obstet Gynecol Reprod Biol, 64(1), 37-42. doi:10.1016/0301-2115(95)02256-2 |
| 607 | Beksaç, M. S., Egemen, A., Izzetoglu, K., Ergün, G., & Erkmen, A. M. (1996). An automated intelligent diagnostic system for the interpretation of umbilical artery Doppler velocimetry. Eur J Radiol, 23(2), 162-167. doi:10.1016/0720-048x(96)01067-4 |
| 608 | Beksaç, M. S., Durak, B., Ozkan, O., Cakar, A. N., Balci, S., Karakaş, U., & Laleli, Y. (1995). An artificial intelligent diagnostic system with neural networks to determine genetical disorders and fetal health by using maternal serum markers. Eur J Obstet Gynecol Reprod Biol, 59(2), 131-136. doi:10.1016/0028-2243(94)02034-c |
| 609 | Braaten, O. (1996). Artificial intelligence in pediatrics: important clinical signs in newborn syndromes. Comput Biomed Res, 29(3), 153-161. doi:10.1006/cbmr.1996.0013 |
| 610 | Forkert, N. D., Schmidt-Richberg, A., Fiehler, J., Illies, T., Möller, D., Handels, H., & Säring, D. (2011). Fuzzy-based vascular structure enhancement in Time-of-Flight MRA images for improved segmentation. Methods Inf Med, 50(1), 74-83. doi:10.3414/me10-02-0003 |
| 611 | Raef, B., Maleki, M., & Ferdousi, R. (2019). Computational prediction of implantation outcome after embryo transfer. Health Informatics J, 1460458219892138. doi:10.1177/1460458219892138 |
| 612 | Spilka, J., Frecon, J., Leonarduzzi, R., Pustelnik, N., Abry, P., & Doret, M. (2015). Intrapartum fetal heart rate classification from trajectory in Sparse SVM feature space. Conf Proc IEEE Eng Med Biol Soc, 2015, 2335-2338. doi:10.1109/embc.2015.7318861 |
| 613 | Uyar, A., Bener, A., Ciray, H., & Bahceci, M. (2009). A frequency based encoding technique for transformation of categorical variables in mixed IVF dataset. Conf Proc IEEE Eng Med Biol Soc, 2009, 6214-6217. doi:10.1109/iembs.2009.5334548 |
| 614 | Shiyu Sara Huang CIL, Liping Zhou, Yu Sun. An intelligent mobile system to predict blood sugar level for gestational diabetes patients using machine learning. Journal of Computers 2018;13:1227. doi, PMID. |
| 615 | Weber KA, Yang W, Carmichael SL, Padula AM, Shaw GM. A machine learning approach to investigate potential risk factors for gastroschisis in california. Birth Defects Research 2019;111:212-21. doi: https://doi.org/10.1002/bdr2.1441. |
| 616 | Avni, F. E., Garel, C., Cassart, M., D'Haene, N., Hall, M., & Riccabona, M. (2012). Imaging and classification of congenital cystic renal diseases. AJR Am J Roentgenol, 198(5), 1004-1013. doi:10.2214/ajr.11.8083 |
| 617 | Cao, L., Graauw, M., Yan, K., Winkel, L., & Verbeek, F. J. (2016). Hierarchical classification strategy for Phenotype extraction from epidermal growth factor receptor endocytosis screening. BMC Bioinformatics, 17(1), 196. doi:10.1186/s12859-016-1053-2 |
| 618 | Diab, M. O., Marque, C., & Khalil, M. A. (2005). Unsupervised classification in uterine electromyography signal: toward the detection of preterm birth. Conf Proc IEEE Eng Med Biol Soc, 2005, 5660-5663. doi:10.1109/iembs.2005.1615770 |
| 619 | Jurisica, I., Mylopoulos, J., Glasgow, J., Shapiro, H., & Casper, R. F. (1998). Case-based reasoning in IVF: prediction and knowledge mining. Artif Intell Med, 12(1), 1-24. doi:10.1016/s0933-3657(97)00037-7 |
| 620 | Looney, P., Stevenson, G. N., Nicolaides, K. H., Plasencia, W., Molloholli, M., Natsis, S., & Collins, S. L. (2018). Fully automated, real-time 3D ultrasound segmentation to estimate first trimester placental volume using deep learning. JCI Insight, 3(11). doi:10.1172/jci.insight.120178 |
| 621 | Lovell, D. R., Rosario, B., Niranjan, M., Prager, R. W., Dalton, K. J., Derom, R., & Chalmers, J. (1997). Design, construction and evaluation of systems to predict risk in obstetrics. Int J Med Inform, 46(3), 159-173. doi:10.1016/s1386-5056(97)00068-3 |
| 622 | Grzymala-Busse JW, Woolery LK. Improving prediction of preterm birth using a new classification scheme and rule induction. Proceedings / the Annual Symposium on Computer Application [sic] in Medical Care Symposium on Computer Applications in Medical Care 1994:730-34. doi, PMID. |
| 623 | Guidi G, Adembri G, Vannuccini S, Iadanza E. Predictability of some pregnancy outcomes based on svm and dichotomous regression techniques. Lecture Notes in Computer Science (including subseries Lecture Notes in Artificial Intelligence and Lecture Notes in Bioinformatics). 2014;8868:163-66 |
| 624 | Gupta L, Sisodia RS, Pallavi V, Firtion C, Ramachandran G. Segmentation of 2d fetal ultrasound images by exploiting context information using conditional random fields. Conf Proc IEEE Eng Med Biol Soc 2011;2011:7219-22. doi: https://doi.org/10.1109/iembs.2011.6091824. |
| 625 | Patil SN, Wali UV, Swamy MK. Selection of single potential embryo to improve the success rate of implantation in ivf procedure using machine learning techniques. 2019 International Conference on Communication and Signal Processing (ICCSP) Year:0881-86. doi: https://doi.org/10.1109/ICCSP.2019.8697982. |
| 626 | Bao, J., Liu, J., Qu, Y., & Mu, D. L. (2019). [Predictive value of umbilical arterial cord pH on complications during hospitalization in neonates after cesarean section]. Beijing Da Xue Xue Bao Yi Xue Ban, 51(1), 159-164. doi:10.19723/j.issn.1671-167X.2019.01.027 |
| 627 | Boo, H., Park, Y.-S., Choi, W., & Hoh, J.-K. (2015). ISP-13-5 Prediction of low birth weight infants with nonlinear dynamic indices in the third trimester of pregnancy: A comparison of support vector machines and multiple logistic regression models (Group 13 Perinatology 3, IS Poster, International Session). 日本産科婦人科學會雜誌, 67(2), 1037. |
| 628 | Branger, B., Dochez, V., Gervier, S., & Winer, N. (2018). [Cesarean after labor induction: Risk factors and prediction score]. Gynecol Obstet Fertil Senol, 46(5), 458-465. doi:10.1016/j.gofs.2018.03.008 |
| 629 | Bruno, V., Biasiotti, M., D'Orazio, M., Pietropolli, A., Abundo, P., Ticconi, C., . . . Rosato, N. (2019). Artificial Intelligence (AI) based-method applied in recurrent pregnancy loss (RPL) patients diagnostic work-up and classification: a potential innovation in common clinical practice. Human Reproduction, 34, 55-56. |
| 630 | Caballero Sanz, S., Nozaleda Pastor, G., & Garcia-Tizon Larroca, S. (2018). First-Trimester Biochemical Screening For Low Birth Weight: Clinical Effectiveness of Low Pregnancy-Associated Plasma Protein-A and High Thyroid-Stimulating Hormone. Clin Lab, 64(9), 1501-1508. doi:10.7754/Clin.Lab.2018.180336 |
| 631 | Chéles, D. S., Molin, E. A. D., Rocha, J. C., & Nogueira, M. F. G. (2020). Mining of variables from embryo morphokinetics, blastocyst's morphology and patient parameters: an approach to predict the live birth in the assisted reproduction service. JBRA Assist Reprod. doi:10.5935/1518-0557.20200014 |
| 632 | Chen, Z., Liang, K., Liu, J., & Liang, W. (2008). Diagnosis of abortion with power Doppler ultrasound combining Logistic regression model and receiver operating characteristics curve during the early pregnancy. Chinese Journal of Postgraduates of Medicine, 31(24), 10-13. |
| 633 | Chen, C., Yan, Y., He, Q., Gao, X., Xiang, S., Sha, T., . . . Li, L. (2018). [Risk factors for delayed breastfeeding initiation based on decision tree model and logistic regression model]. Zhong Nan Da Xue Xue Bao Yi Xue Ban, 43(3), 306-312. doi:10.11817/j.issn.1672-7347.2018.03.012 |
| 634 | Chiarelli, P. A., Hauptman, J. S., & Browd, S. R. (2018). Machine Learning and the Prediction of Hydrocephalus: Can Quantitative Image Analysis Assist the Clinician? JAMA Pediatr, 172(2), 116-118. doi:10.1001/jamapediatrics.2017.4450 |
| 635 | Czabański, R., Jezewski, J., Wróbel, J., Sikora, J., & Jezewski, M. (2013). Application of fuzzy inference systems for classification of fetal heart rate tracings in relation to neonatal outcome. Ginekol Pol, 84(1), 38-43. doi:10.17772/gp/1538 |
| 636 | Dithy, M. D., & Krishna Priya, V. (2019). Anemia screening in pregnant women by using vect neighbour classification algorithm. Journal of Advanced Research in Dynamical and Control Systems, 11(4), 1894-1905. |
| 637 | EBRAHIMZADEH, F., ZAYERI, F., VAHABI, N., AZARBAR, A., BAKHTIYAR, K., & HOSSEINI, A. F. (2015). Comparison of neural networks, decision trees, discriminant analysis and logistic regression for predicting unwanted pregnancy of multiparous women in Khorramabad. |
| 638 | Etikan, I., & Caglar, M. K. (2005). Prediction methods for babies' birth weight using linear and nonlinear regression analysis. Technol Health Care, 13(2), 131-135. |
| 639 | Forest, J. C., Massé, J., Bujold, E., Lafond, J., Charland, M., Rousseau, F., & Giguère, Y. (2012). PP054. Predicting preeclampsia at late mid-term pregnancy before occurrence of clinical symptoms: Clinical utility of biomarkers and clinical parameters in a low-risk population. Pregnancy Hypertens, 2(3), 271. doi:10.1016/j.preghy.2012.04.165 |
| 640 | Forest, J. C., Massé, J., Bujold, E., Rousseau, F., Charland, M., Thériault, S., . . . Giguère, Y. (2012). OS090. Performance of candidate clinical and biochemical markers in screening early in pregnancy to detect women at high risk to develop preeclampsia. Pregnancy Hypertens, 2(3), 227. doi:10.1016/j.preghy.2012.04.091 |
| 641 | GONG, X.-y., CAI, X., & SHI, M. (2006). Logistic regression analysis of influential factors on the pregnancy outcome of *in vitro* fertilization and embryo transfer. Journal of xinjiang medical university, 29(2), 109. |
| 642 | Gratacos, E., Cruz-Martinez, R., Moreno-Alvarez, O., Hernandez-Andrade, E., Castañon, M., Done, E., . . . Deprest, J. (2010). Contribution of intrapulmonary artery Doppler to improve prediction of survival in fetuses with congenital diaphragmatic hernia treated with fetal endoscopic tracheal occlusion. Ultrasound in Obstetrics and Gynecology, 35(5), 572-577. doi:10.1002/uog.7593 |
| 643 | Guo, Y., Yang, J., Cai, J., Peng, L., & Yan, W. (2013). Multivariable prediction model and clinical outcome impact of ovarian hyper-response during controlled ovarian stimulation. Medical Journal of Wuhan University, 34(5), 751-754+761. |
| 644 | He, Y., Xia, R., Chen, X., Ye, D., Tang, Y., Li, P., . . . Chen, S. (2013). [Estimation of ovarian response using multiple predictors of ovarian reserve in women undergoing *in vitro* fertilization-embryo transfer]. Nan Fang Yi Ke Da Xue Xue Bao, 33(2), 216-220. |
| 645 | He, L. F., Zhao, Y., & Wang, Z. P. (2015). [Prediction model of fetal meconium-stained amniotic fluid in re-pregnant women with intrahepatic cholestasis of pregnancy]. Zhejiang Da Xue Xue Bao Yi Xue Ban, 44(3), 264-268. |
| 646 | Heredia-Olivera, K., & Munares-García, O. (2016). [Maternal factors associated with low birth weight]. Rev Med Inst Mex Seguro Soc, 54(5), 562-567. |
| 647 | Iftikhar, P., Kuijpers, M. V., Khayyat, A., Iftikhar, A., & DeGouvia De Sa, M. (2020). Artificial Intelligence: A New Paradigm in Obstetrics and Gynecology Research and Clinical Practice. Cureus, 12(2), e7124. doi:10.7759/cureus.7124 |
| 648 | Jeschke, U., Karamouti, M., Toth, B., Haufe, T., Scholz, C., Kuhn, C., & Friese, K. B* 07,-B* 08,-B* 52,-B* 4001 and HLA class II alleles: HLA-DRB1* 15,-DQB1* 0501/2,-DRB3* 03. Impact of pregnancy outcome in first pregnancy after referral was assessed using logistic regression adjusting for the number of previous miscarriages. Results: HY-restricting HLA class II significantly impact future pregnancies. |
| 649 | Leão, B. D., Reátegui, E. B., Guazzelli, A., & Mendonça, E. A. (1995). Hybrid systems: a promising solution for better decision support tools. Medinfo, 8 Pt 1, 823-827. |
| 650 | Leão, B. D., Reátegui, E. B., Guazzelli, A., & Mendonça, E. A. (1996). Hycones: a hybrid approach to designing decision support systems. MD Comput, 13(2), 160-164. |
| 651 | Liao, Y., Liu, X. H., Tan, J., He, G. L., Yang, H. M., & Chen, M. (2018). [Development of a Predictive Model for Adverse Outcomes of Preeclampsia]. Sichuan Da Xue Xue Bao Yi Xue Ban, 49(5), 797-802. |
| 652 | Lin, F., & Sun, X. (2018). Logistic Regression and ROC Curve for the Diagnostic Value of Progesterone, β-HCG and Serum Sugar Chain Protein 125 in Pregnancy Outcome in Patients with Early Threatened Abortion. Journal of Medical Research, 47(1), 152-155. |
| 653 | Lu, Y., You, Q., & Li, X. (2016). [Automatical Assessment of Fetal Status Based on Fuzzy Theory and Euclidean Distance]. Sheng Wu Yi Xue Gong Cheng Xue Za Zhi, 33(3), 436-441. |
| 654 | Marini, A. (2007). Early prediction of poor outcome in extremely low birth weight infants by classification tree analysis. Journal of Pediatrics, 150(6). doi:10.1016/j.jpeds.2007.02.022 |
| 655 | Mateen, B. A., David, A. L., & Denaxas, S. (2020). Electronic Health Records to Predict Gestational Diabetes Risk. Trends Pharmacol Sci, 41(5), 301-304. doi:10.1016/j.tips.2020.03.003 |
| 656 | Meng-yao, X., Shan-shan, L., Shao-nong, D., & Hong, Y. (2019). Multilevel Logistic regression model analysis between pregnancy history of women and small-for-gestational age of infants in Shaanxi Province/陕西省孕龄妇女既往生育史与小于胎龄儿的多水平 Logistic 回归分析. Xi'an jiao tong da xue xue bao. Yi xue ban(5), 815. |
| 657 | Muscatello, A., Di Nicola, M., Accurti, V., Mastrocola, N., Franchi, V., Colagrande, I., . . . Carta, G. (2014). Sonoelastography as method for preliminary evaluation of uterine cervix to predict success of induction of labor. Fetal Diagn Ther, 35(1), 57-61. doi:10.1159/000355084 |
| 658 | Patumanond, J., Tawichasri, C., & Khunpradit, S. (2012). Clinical risk score to recognize macrosomia at the time of delivery. Clin Exp Obstet Gynecol, 39(2), 195-199. |
| 659 | Prema, N. S., & Pushpalatha, M. P. (2019). An ensemble model for the prediction of gestational diabetes mellitus (GDM). Indian Journal of Public Health Research and Development, 10(9), 44-48. doi:10.5958/0976-5506.2019.02399.4 |
| 660 | Qiu, Y., Wen, Y., Li, G., Tao, Z., Yan, X., Zang, N., . . . Huang, Q. (2018). [Maternal neutrophil-to-lymphocyte ratio as a prognostic biomarker for placental inflammatory response in late pregnancy]. Nan Fang Yi Ke Da Xue Xue Bao, 38(9), 1131-1134. doi:10.12122/j.issn.1673-4254.2018.09.18 |
| 661 | Rezaei, M., Fakhri, N., Rajati, F., & Shahsavari, S. (2019). Comparison of gestational diabetes prediction with artificial neural network and decision tree models. Tehran University Medical Journal, 77(6), 359-367. |
| 662 | Rezaei, M., Fakhri, N., Shahsavari, S., & Rajati, F. (2020). Comparison of gestational diabetes prediction between logistic regression, discriminant analysis, decision tree and artificial neural network models. Iranian Journal of Epidemiology, 15(4), 362-371. |
| 663 | Saadati, N., Ahmadzadeh, B., Najafian, M., & Toghiyani, M. (2018). Estimation of fetal weight by fundal height measurement using regression and decision tree models in Ahwaz Imam Khomeini hospital in winter 2014. Iranian Journal of Obstetrics, Gynecology and Infertility, 20(11), 29-38. doi:10.22038/IJOGI.2018.10225 |
| 664 | SADAT, H. S., KAZEMNEJAD, A., LUCAS, C., BADIEI, K., KAVEHEI, B., & RAHNAVARD, Z. (2004). COMPARING PREDICTIONS OF TYPE OF PREGNANCY USING ARTIFICIAL NEURAL NETWORKS AND MULTINOMIAL LOGISTIC REGRESSION. |
| 665 | Seufert, R., Woernle, F., & Casper, F. (2000). [Computer-assisted cardiotocogram analysis--from descriptive to perinatal expert system]. Zentralbl Gynakol, 122(6), 328-333. |
| 666 | Steffann, J., Feyereisen, E., Kerbrat, V., Romana, S., & Frydman, N. (2005). [Prenatal and preimplantation genetic diagnosis: decision tree, new practices?]. Med Sci (Paris), 21(11), 987-992. doi:10.1051/medsci/20052111987 |
| 667 | Tan, Z., Wang, W., Zong, R., Pan, J., & Yang, H. (2019). [Classification of heart sound signals in congenital heart disease based on convolutional neural network]. Sheng Wu Yi Xue Gong Cheng Xue Za Zhi, 36(5), 728-736. doi:10.7507/1001-5515.201806031 |
| 668 | Tylcz, J. B., Muszynski, C., Dauchet, J., Istrate, D., & Marque, C. (2020). An Automatic Method for the Segmentation and Classification of Imminent Labor Contraction from Electrohysterograms. Ieee Transactions on Biomedical Engineering, 67(4), 1133-1141. doi:10.1109/TBME.2019.2930618 |
| 669 | Wald, M., Sparks, A. E. T., Van Voorhis, B. J., Syrop, C. H., & Niederberger, C. S. (2007). Computational models for prediction of intrauterine insemination outcomes. Journal of the Turkish German Gynecology Association, 8(3), 302-307. |
| 670 | Wang, W., Xu, W., Zheng, Y. J., & Zhou, B. S. (2007). [Study on a back propogation neural network-based predictive model for prevalence of birth defect]. Zhonghua Liu Xing Bing Xue Za Zhi, 28(5), 507-509. |
| 671 | Wang, R. J., Pan, W., Jin, L., Li, Y. H., Geng, Y. D., Gao, C., . . . Liao, S. J. (2019). Artificial intelligence in reproductive medicine. Reproduction, 158(4), R139-R154. doi:10.1530/rep-18-0523 |
| 672 | Wang, S., Pathak, J., & Zhang, Y. (2019). Using Electronic Health Records and Machine Learning to Predict Postpartum Depression. Stud Health Technol Inform, 264, 888-892. doi:10.3233/shti190351 |
| 673 | Wischnik, A., Lehmann, K. J., Labeit, D., Werner, T., Gerlach-Schmidt, H., Hiltmann, W. D., & Melchert, F. (1993). [A knowledge-based system for the interpretation of pelvimetric findings]. Z Geburtshilfe Perinatol, 197(6), 266-274. |
| 674 | Wu, J., Yang, T., Lin, J., Luo, H., Li, D., Wang, T., & Zheng, C. (2005). [Estimation of fetal weight on the basis of neural network]. Sheng Wu Yi Xue Gong Cheng Xue Za Zhi, 22(5), 922-925, 929. |
| 675 | Xiong, W., & Luo, H. (2017). [Convention Ultrasound and Contrast-enhanced Ultrasound Imaging in the Diagnosis of Placenta Implantation]. Sichuan Da Xue Xue Bao Yi Xue Ban, 48(2), 253-256. |
| 676 | Xu, X., Tan, H., Zhou, S., He, Y., Shen, L., Liu, Y., . . . Li, X. (2014). [Study on the application of Back-Propagation Artificial Neural Network used the model in predicting preterm birth]. Zhonghua Liu Xing Bing Xue Za Zhi, 35(9), 1028-1031. |
| 677 | Yi, W. J., Park, K. S., & Paick, J. S. (1998). Morphological classification of sperm heads using artificial neural networks. Stud Health Technol Inform, 52 Pt 2, 1071-1074. |
| 678 | Yoffe, L., Polsky, A., Gilam, A., Raff, C., Mecacci, F., Ognibene, A., . . . Hod, M. (2019). Early diagnosis of gestational diabetes mellitus using circulating microRNAs. Eur J Endocrinol, 181(5), 565-577. doi:10.1530/eje-19-0206 |
| 679 | Zhou, L. B., Zheng, L., Luo, J. Y., Du, Q. Y., Fang, J. Q., & Sun, Z. Q. (2008). [Risk prediction model of perinatal congenital heart disease]. Zhonghua Liu Xing Bing Xue Za Zhi, 29(12), 1251-1254. |
| 680 | Francis F, Bedeeuzzaman M, Fathima T. Preterm birth prediction using electrohysterography with local binary patterns. Year:319-24. doi, PMID. |

^a^ Sorted from the studies with most complete to incomplete criteria

b This table described 680 studies after human screening by title and abstract. For complete records (*n*=2093), we provided the list of the studies in Table S11

# Table S5. Description of eligible studies.

| Study Author (Publication Year) | Outcome | MeSH Term | Study Design | Data Source Author (Publication Year) | Data Source Design | Country | Primary care | Hospital | Other | From | To | DV | M |
| --- | --- | --- | --- | --- | --- | --- | --- | --- | --- | --- | --- | --- | --- |
|  |  |  |  |  |  |  |  |  |  |  |  |  |  |
| Abbas SA et al. (2018) | Cesarean section | Cesarean section | Cross-sectional | Abbas SA et al. (2018) | Cross-sectional | Pakistan | 0 | 2 | 0 |  |  | D | No |
| Agopian AJ et al. (2012) | Spina bifida or anencephaly | Trisomy | Nested case-control | Yoon PW et al. (2001) | Nested Case-Control | United States |  |  |  | 1997 | 2005 | D | Yes |
| Alberola-Rubio J et al. (2017) | Late term labor | Obstetric labor | Retrospective | Alavifard S et al. (2019) | Retrospective | Canada | 0 | 1 | 0 | 2016 | 2016 | D | No |
| Allouche M et al. (2011) | Early preterm delivery | Premature birth | Retrospective | Allouche M et al. (2011) | Retrospective | France | 0 | 2 | 0 | 2004 | 2008 | DV | No |
| Almeida ST et al. (2017) | Complication of severe preeclampsia | Pregnancy-induced hypertension | Retrospective | Almeida ST et al. (2017) | Retrospective | Brazil | 0 | 1 | 0 | 2014 | 2014 | DV | Yes |
| Al-Rubaie ZT et al. (2020) | Preeclampsia | Pregnancy-induced hypertension | Retrospective | Al-Rubaie ZT et al. (2020) | Retrospective | Australia | 0 | 3 | 0 | 2011 | 2014 | DV | Yes |
| Amini P et al. (2017) | Preterm delivery | Premature birth | Cross-sectional | Amini P et al. (2017) | Cross-Sectional | Iran | 0 | 4 | 0 | 2015 | 2015 | D | No |
| Artzi NS et al. (2020) | Gestational diabetes | Gestational diabetes | Retrospective | Artzi NS et al. (2020) | Retrospective | Israel |  |  |  | 2010 | 2018 | DV | Yes |
| Balani J et al. (2018) | Gestational diabetes | Gestational diabetes | Retrospective | Balani J et al. (2014) | Prospective | United Kingdom | 0 | 1 | 0 | 2010 | 2011 | D | No |
| Bastek JA et al. (2012) | Preterm delivery | Premature birth | Retrospective |  | Prospective | United States | 0 | 1 | 0 | 2008 | 2010 | D | No |
| Benalcazar-Parra C et al. (2019) | Successful induction of labor | Induced labor | Prospective | Benalcazar-Parra C et al. (2019) | Prospective | Spain | 0 | 1 | 0 |  |  | D | No |
| Benhalima K et al. (2020) | Gestational diabetes | Gestational diabetes | Retrospective | Benhalima K et al. (2014) | Prospective | Belgium | 0 | 6 | 0 | 2014 | 2018 | D | Yes |
| Berntorp K et al. (2015) | Large for gestational age | Fetal development | Retrospective | Anderberg E et al. (2010) | Prospective | Sweden | 1 | 4 | 0 | 2003 | 2005 | D | No |
| Blank C et al. (2019) | Ongoing pregnancy | Fertilization *in vitro* | Retrospective | Blank C et al. (2019) | Retrospective | Belgium | 0 | 1 | 0 | 2015 | 2017 | D | Yes |
| Borup R et al. (2016) | Ongoing pregnancy | Fertilization *in vitro* | Nested case-control | Feuerstein P et al. (2012) | Nested case-control | Denmark | 0 | 1 | 0 | 2009 | 2010 | DV | Yes |
| Broekmans FJ et al. (2014) | Ovarian response to stimulation | Fertilization *in vitro* | Retrospective | Devroey P et al (2009); Nyboe Andersen A et al. (2011) | Prospective | United States, Canada, Spain, The United Kingdoms, Belgium, Czech Republic, Finland, France, Norway, Sweden, Denmark and The Netherlands |  |  |  | 2006 | 2008 | DV | No |
| Carlsson FM et al. (2020) | Vaginal birth after cesarean | Obstetric labor | Retrospective | Carlsson FM et al. (2020) | Retrospective | Sweden |  |  |  | 1998 | 2013 | D | No |
| Casikar I et al. (2013) | Succesful expectant management of miscarriage | Others | Prospective | Casikar I et al. (2013) | Prospective | Australia | 0 | 1 | 0 | 2006 | 2009 | DV | No |
| Cerqueira FR et al. (2014) | Premature newborn death | Premature birth | Retrospective | Catley C et al. (2006) | Retrospective | Canada |  |  |  | 1999 | 2002 | D | No |
| Chandrasekaran S et al. (2016) | Large for gestational age | Fetal development | Retrospective | Chandrasekaran S et al. (2016) | Retrospective | United States, Canada, Spain, The UK, Belgium, Czech Republic, Finland, France, Norway, Sweden, Denmark and The Netherlands | 0 | 1 | 0 | 2008 | 2011 | D | Yes |
| Chen L and Hao Y (2017) | Preterm delivery | Premature birth | Retrospective | Alexandersson A et al. (2015) | Prospective | Iceland | 1 | 2 | 0 | 2008 | 2010 | D | No |
| Chen L et al. (2018) | Stress urinary incontinence | Others | Retrospective | Chen L et al. (2018) | Cross-Sectional | China | 0 | 2 | 0 | 2016 | 2017 | DV | Yes |
| Chen L et al. (2019) | Preterm delivery | Premature birth | Retrospective | Alexandersson A et al. (2015) | Retrospective | Iceland | 1 | 1 | 0 | 2008 | 2010 | D | No |
| Ciobanu A et al. (2019) | Small for gestational age | Small for gestational age infant | Retrospective | Ciobanu A et al. (2019) | Retrospective | United Kingdom | 0 | 1 | 0 | 2014 | 2018 | D | Yes |
| Comert Z et al. (2018) | Acidotic blood pH of umbilical artery | Fetal distress | Retrospective | Chudácek V et al. (2014) | Prospective | Czech | 0 | 1 | 0 | 2010 | 2012 | D | Yes |
| Coppede F et al. (2010) | Down syndrome | Trisomy | Nested case-control | Coppede F et al. (2007); Coppede F et al. (2009) | Nested Case-Control | Italy | 0 | 1 | 1 |  |  | D | Yes |
| Cortet M et al. (2015) | Postpartum hemorrhage | Others | Retrospective | Deneux-Tharaux C et al. (2010) | Retrospective | France | 0 | 106 | 0 | 2004 | 2006 | D | Yes |
| Crovetto F et al. (2015) | Early Preeclampsia | Pregnancy-induced hypertension | Nested case-control | Crovetto F et al. (2015) | Nested Case-Control | Spain | 0 | 2 | 0 |  |  | D | Yes |
| de Oliveira RV et al. (2012) | Preterm delivery | Premature birth | Prospective | de Oliveira RV et al. (2012) | Prospective | Brazil | 0 | 1 | 0 | 2005 | 2010 | D | No |
| de Wilde MA et al. (2014) | Gestational diabetes | Gestational diabetes | Prospective | de Wilde MA et al. (2014) | Prospective | Netherlands | 0 | 4 | 0 | 2008 | 2012 | D | Yes |
| Despotovic D et al. (2018) | Preterm delivery | Premature birth | Nested case-control | Fele-Zorz G et al. (2008) | Nested case-control | Slovenia | 0 | 1 | 0 | 1997 | 2006 | D | No |
| Eggebo TM et al. (2015) | Vaginal delivery | Obstetric labor | Prospective | Eggebo TM et al. (2015) | Prospective | Norway, United Kingdom | 0 | 2 | 0 | 2012 | 2013 | D | Yes |
| Elaveyni U et al. (2011) | Preterm delivery | Premature birth | Retrospective | Elaveyni U et al. (2011) | Retrospective | India | 0 | 1 | 0 | 2007 | 2009 | D | No |
| Fagerberg MC et al. (2015) | Vaginal birth after cesarean | Obstetric labor | Retrospective | Fagerberg MC et al. (2015) | Retrospective | Sweden |  |  |  | 1992 | 2011 | DV | No |
| Fergus P et al. (2013) | Preterm delivery | Premature birth | Nested case-control | Fele-Zorz G et al. (2008) | Nested case-control | Slovenia | 0 | 1 | 0 | 1997 | 2006 | D | No |
| Fergus P et al. (2016) | Preterm delivery | Premature birth | Nested case-control | Fele-Zorz G et al. (2008) | Nested case-control | Slovenia | 0 | 1 | 0 | 1997 | 2006 | D | No |
| Fergus P et al. (2017) | Cesarean section | Cesarean section | Retrospective | Chudácek V et al. (2014) | Retrospective | Czech | 0 | 1 | 0 | 2010 | 2012 | D | No |
| Fergus P et al. (2018) | Preterm delivery | Premature birth | Nested case-control | Wang X et al. (2002) | Nested case-control | United States | 1 | 0 | 0 | 1998 | 2000 | D | No |
| Figueras F et al. (2015) | Small for gestational age | Small for gestational age infant | Prospective | Figueras F et al. (2015) | Prospective | Spain | 0 | 1 | 0 | 2008 | 2012 | D | Yes |
| Fiset S et al. (2019) | Preterm delivery | Premature birth | Retrospective | Fiset S et al. (2019) | Retrospective | Canada | 0 | 1 | 0 | 2016 | 2017 | D | No |
| Gao C et al. (2019) | Extremely preterm delivery | Premature birth | Retrospective | Gao C et al. (2019) | Retrospective | United States | 1 | 0 | 0 | 2005 | 2017 | DV | Yes |
| Garces MF et al. (2015) | Preeclampsia | Pregnancy-induced hypertension | Nested case-control | Garces MF et al. (2015) | Nested Case-Control | Colombia | 0 | 1 | 0 | 2012 | 2014 | D | No |
| Georgulas G, Karvelis P, Spilka J et al. (2017) | Acidotic blood pH of umbilical artery | Fetal distress | Cross-sectional | Chudácek V et al. (2014) | Prospective | Czech Republic | 0 | 1 | 0 | 2010 | 2012 | D | No |
| Guo Z et al. (2020) | Preeclampsia | Pregnancy-induced hypertension | Nested case-control | Guo Z et al. (2020) | Nested Case-Control | China | 0 | 3 | 0 | 2013 | 2017 | DV | No |
| Hamdi MA et al. (2019) | Preterm delivery | Premature birth | Nested case-control | Fele-Zorz G et al. (2008) | Nested Case-Control | Slovenia | 0 | 1 | 0 | 1997 | 2006 | D | No |
| Harper LM et al. (2016) | Successful glyburide therapy in gestational diabetes | Gestational diabetes | Retrospective | Harper LM et al. (2016) | Prospective | United Kingdom | 0 | 1 | 0 | 2007 | 2013 | D | No |
| Hernandez-Gonzalez J et al. (2018) | Ongoing pregnancy | Fertilization *in vitro* | Nested case-control | Hernandez-Gonzalez J et al. (2018) | Nested case-control | Spain | 0 | 1 | 0 | 2013 | 2015 | D | No |
| Isakov O et al. (2019) | Successful external cephalic version | Obstetric labor | Retrospective | Isakov O et al. (2019) | Retrospective | Israel | 0 | 1 | 0 | 2016 | 2018 | D | Yes |
| Isono W et al. (2011) | Successful induction of labor | Induced labor | Retrospective | Isono W et al. (2011) | Retrospective | Japan | 0 | 1 | 0 | 2002 | 2009 | D | Yes |
| Jhee JH et al. (2019) | Late preeclampsia | Pregnancy-induced hypertension | Retrospective | Jhee JH et al. (2019) | Retrospective | South Korea | 0 | 1 | 0 | 2005 | 2017 | D | No |
| Kang J et al. (2020) | Massive transfusion during cesarean section of a pregnant woman with placenta previa | Cesarean section | Retrospective | Kang J et al. (2020) | Retrospective | South Korea | 0 | 2 | 0 | 2011 | 2018 | DV | Yes |
| Kawakita T et al. (2019) | Severe postpartum hemorrhage | Others | Retrospective | Kawakita T et al. (2019) | Retrospective | United States | 0 | 1 | 0 | 2012 | 2017 | D | No |
| Khan N et al. (2019) | Large for gestational age | Fetal development | Retrospective | Akolekar R et al. (2019); Frick AP et al. (2016); Bakalis S et al. (2015); Fadigas C et al. (2015); Ciobanu A et al. (2019); Ciobanu A et al. (2019); Ciobanu A et al. (2019); Ciobanu A et al. (2019); Akolekar R et al. (2019) | Prospective | United Kingdom | 0 | 2 | 0 | 2006 | 2018 | D | Yes |
| Koivu A et al. (2020) | Early stillbirth | Stillbirth | Retrospective | Koivu A et al. (2020) | Retrospective | United States |  |  |  | 2013 | 2016 | DV | Yes |
| Kok M et al. (2011) | Successful external cephalic version | Obstetric labor | Retrospective | Kok M et al. (2008) | Retrospective | Netherlands | 0 | 7 | 0 | 2004 | 2006 | D | Yes |
| Kuhle S et al. (2018) | Small for gestational age | Small for gestational age infant | Cross-sectional | Krupa N et al. (2011) | Cross-Sectional | Malaysia | 0 | 1 | 0 |  |  | D | No |
| Kumar SN et al. (2020) | Low birth weight | Low birth weight infant | Case-control | Kumar SN et al. (2020) | Case-Control | India | 1 | 0 | 0 |  |  | DV | No |
| Lafalla O et al. (2019) | Placenta dysfunction-related diseases | Pregnancy-induced hypertension | Case-control | Lafalla O et al. (2019) | Case-Control | Spain | 0 | 1 | 0 | 2012 | 2013 | D | No |
| Lee JSE et al. (2018) | Epidural re-siting | Cesarean section | Retrospective | Lee JSE et al. (2018); Sng BL et al. (2018) | Retrospective | Singapore | 0 | 1 | 0 | 2012 | 2015 | DV | Yes |
| Lee KS et al. (2019) | Preterm delivery | Premature birth | Retrospective | Lee KS et al. (2019) | Retrospective | South Korea | 0 | 1 | 0 | 2014 | 2018 | D | No |
| Leonarduzzi R et al. (2015) | Acidotic blood pH of umbilical artery | Fetal distress | Nested case-control | Doret M et al. (2011) | Nested case-control | French | 0 | 1 | 0 | 2000 | 2010 | D | Yes |
| Li H et al. (2017) | Congenital heart diseases | Congenital heart defects | Case-control | Li H et al. (2017) | Case-Control | China | 0 | 1 | 0 | 2013 | 2014 | D | Yes |
| Liu B et al. (2019) | Pregnancy | Others | Retrospective | Liu B et al. (2019) | Retrospective |  |  |  |  |  |  | D | No |
| Macones GA et al. (2001) | Vaginal birth after cesarean | Obstetric labor | Retrospective | MacDowell M et al. (2001) | Retrospective | United States | 0 | 1 | 0 | 1994 | 1995 | D | No |
| Mardy AH et al. (2016) | Vaginal birth after cesarean | Obstetric labor | Retrospective | Landon MB et al. (2004) | Prospective | United States | 0 | 19 | 0 | 1999 | 2002 | D | Yes |
| Maroufizadeh S et al. (2018) | Cesarean section | Cesarean section | Cross-sectional | Maroufizadeh S et al. (2018) | Cross-Sectional | Iran | 0 | 4 | 0 | 2015 | 2015 | D | Yes |
| Mas-Cabo J et al. (2019) | Preterm delivery | Premature birth | Retrospective | Fele-Zorz G et al. (2008) | Prospective | Slovenia | 0 | 1 | 0 | 1997 | 2006 | D | No |
| McCowan LM et al. (2013) | Small for gestational age | Small for gestational age infant | Retrospective | Kenny LC et al. (2014) | Prospective | New Zealand, Australia, United Kingdom, Ireland |  |  |  | 2004 | 2011 | D | No |
| McCowan LM et al. (2017) | Hypertensive small for gestational age | Small for gestational age infant | Retrospective | Kenny LC et al. (2014) | Prospective | New Zealand, Australia, United Kingdom, Ireland |  |  |  | 2004 | 2011 | D | Yes |
| Mehta-Lee SS et al. (2017) | Preterm delivery | Premature birth | Retrospective | Mehta-Lee SS et al. (2017) | Retrospective | United States |  |  |  | 2004 | 2009 | D | No |
| Meijerink AM et al. (2016) | Live birth | Fertilization *in vitro* | Retrospective | Meijerink AM et al. (2016) | Retrospective | Netherlands | 0 | 2 | 0 | 2007 | 2015 | DV | Yes |
| Meister MR et al. (2016) | Perineal laceration | Obstetric labor | Nested case-control | Meister MR et al. (2016) | Nested Case-Control | United States | 0 | 1 | 0 | 2004 | 2008 | D | No |
| Mello G et al. (2001) | Pregnancy-induced hypertension | Pregnancy-induced hypertension | Retrospective | Mello G et al. (2001) |  | Italy | 0 | 1 | 0 |  |  | D | No |
| Menon R et al. (2014) | Preterm delivery | Premature birth | Prospective | Menon R et al. (2014) | Prospective | United States | 0 | 1 | 0 | 2003 | 2006 | D | No |
| Metz TD et al. (2013) | Vaginal birth after cesarean | Obstetric labor | Retrospective | Metz TD et al. (2013) | Retrospective | United States | 0 | 15 | 0 | 2000 | 2008 | DV | Yes |
| Milewski R et al. (2013) | Biochemical pregnancy | Fertilization *in vitro* | Retrospective | Metz TD et al. (2013) | Retrospective | United States | 0 | 15 | 0 | 2000 | 2008 | D | Yes |
| Milewski R et al. (2017) | Ongoing pregnancy | Fertilization *in vitro* | Retrospective | Milewski R et al. (2013) | Retrospective | United States | 1 | 0 | 0 |  |  | D | Yes |
| Mirroshandel SA et al. (2016) | Ongoing pregnancy | Fertilization *in vitro* | Retrospective | Mirroshandel SA et al. (2016) | Retrospective | Iran | 0 | 1 | 0 | 2012 | 2014 | D | No |
| Morales DA et al. (2008) | Ongoing pregnancy | Fertilization *in vitro* | Retrospective | Morales DA et al. (2008) | Retrospective | Spain | 1 | 0 | 0 | 2003 | 2005 | D | No |
| Murtoniemi K et al. (2018) | Preeclampsia | Pregnancy-induced hypertension | Retrospective | Girchenko P et al. (2016) | Retrospective | Finland | 10 | 0 | 0 | 2005 | 2009 | D | No |
| Myers JE et al. (2013) | Preterm preeclampsia | Pregnancy-induced hypertension | Retrospective | Kenny LC et al. (2014) | Prospective | New Zealand, Australia, United Kingdom, Ireland |  |  |  | 2004 | 2011 | D | Yes |
| Oates J et al. (2013) | Miscarriage | Others | Prospective | Oates J et al. (2013) | Retrospective | Australia | 0 | 1 | 0 | 2006 | 2009 | D | No |
| Paydar K et al. (2017) | Ongoing pregnancy | Fertilization *in vitro* | Retrospective | Paydar K et al. (2017) | Retrospective | Iran | 1 | 1 | 0 | 1982 | 2014 | D | No |
| Payne BA et al. (2015) | Perinatal death in pregnancy-induced hypertension | Pregnancy-induced hypertension | Retrospective | B.A. Payne et al. (2014) | Prospective | Fiji, Uganda, South Africa, Brazil, Pakistan | 1 | 6 | 0 | 2008 | 2012 | D | Yes |
| Petrozzielo A et al. (2018) | Acidotic blood pH of umbilical artery | Fetal distress | Retrospective | Petrozzielo A et al. (2018); Chudácek V et al. (2014) | Prospective | United Kingdom, Czech Republic | 0 | 2 | 0 | 1993 | 2012 | D | No |
| Petrozzielo A et al. (2019) | Acidotic blood pH of umbilical artery | Fetal distress | Retrospective | Petrozzielo A et al. (2019); SPaM’17 dataset (2017); Chudácek V et al. (2014) | Prospective | United Kingdom, France, Czech Republic | 0 | 5 | 0 | 1993 | 2012 | D | No |
| Pettersson G et al. (2010) | Live birth | Fertilization *in vitro* | Retrospective | Andersen N et al. (2006) | Prospective | Belgium, France, Finland, Czech Republic, Poland, Denmark, Sweden, Israel, Slovenia and Spain | 37 | 0 | 0 | 2004 | 2004 | D | No |
| Pettersson G et al. (2017) | Traction force in vacuum extraction | Obstetric labor | Retrospective | Pettersson G et al. (2017) | Retrospective | Sweden | 0 | 1 | 0 | 2012 | 2015 | D | Yes |
| Qiu H et al. (2017) | Gestational diabetes | Gestational diabetes | Nested case-control | Qiu H et al. (2017) | Nested case-control | China | 0 | 1 | 0 | 2013 | 2016 | D | No |
| Qiu K et al. (2019) | Live birth | Fertilization *in vitro* | Retrospective | Qiu K et al. (2019) | Retrospective | China | 0 | 1 | 0 | 2014 | 2018 | D | Yes |
| Ramanah R et al. (2018) | Acidotic blood pH of umbilical artery | Fetal distress | Retrospective | Ramanah R et al. (2018) | Retrospective | France | 0 | 1 | 0 | 2012 | 2015 | D | No |
| Reid S et al. (2015) | Pouch of Douglas obliteration | Others | Cross-sectional | Reid S et al. (2015) | Cross-Sectional | Australia | 0 | 8 | 0 | 2009 | 2013 | D | Yes |
| Rinaudo P et al. (2012) | Ongoing pregnancy | Fertilization *in vitro* | Prospective | Rinaudo P et al. (2012) | Prospective | United States | 1 | 0 | 0 |  |  | D | Yes |
| Ryu A et al. (2019) | Low birth weight | Low birth weight infant | Prospective | Ryu A et al. (2019) | Prospective | South Korea | 0 | 1 | 0 | 2015 | 2016 | D | Yes |
| Sadi-Ahmed N et al. (2017) | Preterm delivery | Premature birth | Nested case-control | Fele-Zorz G et al. (2008) | Nested case-control | Slovenia | 0 | 1 | 0 | 1997 | 2006 | D | No |
| Saleem S et al. (2019) | Cesarean section | Cesarean section | Retrospective | Chudácek V et al. (2014) | Retrospective | Czech | 0 | 1 | 0 | 2010 | 2012 | D | No |
| Sananes N et al. (2013) | Preterm delivery | Premature birth | Prospective | Sananes N et al. (2013) | Prospective | France | 0 | 1 | 0 | 2000 | 2012 | DV | Yes |
| Sandstrom A et al. (2019) | Preeclampsia | Pregnancy-induced hypertension | Retrospective | Sandstrom A et al. (2019) | Retrospective | Sweden |  |  |  | 2008 | 2013 | D | Yes |
| Scheinhardt MO et al. (2018) | Ovarian response to stimulation | Fertilization *in vitro* | Retrospective | Lerman T et al. (2017) | 0 | Germany, Norway | 5 | 0 | 0 | 2012 | 2013 | V | Yes |
| Shahbakhti M et al. (2019) | Preterm delivery | Premature birth | Retrospective | Fele-Zorz G et al. (2008) | Prospective | Slovenia | 0 | 1 | 0 | 1997 | 2006 | D | No |
| Shi W et al. (2013) | Ongoing pregnancy | Fertilization *in vitro* | Retrospective | Shi W et al. (2013) | Retrospective | China | 1 | 0 | 0 | 2008 | 2013 | D | Yes |
| Signorini MG et al. (2020) | Intrauterine growth restriction | Fetal development | Case-control | Signorini MG et al. (2020) | Case-Control | Italy | 0 | 1 | 0 |  |  | D | Yes |
| Sims CJ et al. (2000) | Cesarean section | Cesarean section | Retrospective | Sims CJ et al. (2000) | Retrospective | United States | 0 | 1 | 0 | 1995 | 1997 | D | No |
| Sovio U et al. (2018) | Cesarean section | Cesarean section | Retrospective | Sovio U et al. (2015) | Prospective | United Kingdom | 0 | 1 | 0 | 2008 | 2012 | D | No |
| Spilka J et al. (2015) | Acidotic blood pH of umbilical artery | Fetal distress | Nested case-control | Doret M et al. (2011) | Nested case-control | French | 0 | 1 | 0 | 2000 | 2010 | D | Yes |
| Spilka J et al. (2017) | Acidotic blood pH of umbilical artery | Fetal distress | Nested case-control | Doret M et al. (2011) | Nested case-control | French | 0 | 1 | 0 | 2000 | 2010 | D | No |
| Stamatopoulos N et al. (2015) | Miscarriage | Others | Retrospective | Stamatopoulos N et al. (2015) | Retrospective | Australia | 0 | 1 | 0 | 2006 | 2013 | DV | Yes |
| Stott D et al. (2017) | Response to oral labetalol for pregnancy-induced hypertension | Pregnancy-induced hypertension | Prospective | Stott D et al. (2017) | Prospective | United Kingdom | 0 | 1 | 0 | 2013 | 2013 | D | Yes |
| Stroux L et al. (2017) | Intrauterine growth restriction | Fetal development | Cross-sectional | Stroux L et al. (2017) | Cross-Sectional | United Kingdom | 0 | 1 | 0 | 1990 | 2011 | D | No |
| Sufriyana H et al. (2020) | Preeclampsia | Pregnancy-induced hypertension | Nested case-control | Sufriyana H et al. (2020) | Nested Case-Control | Indonesia |  |  |  | 2015 | 2016 | DV | Yes |
| Tessmer-Tuck JA et al. (2014) | Vaginal birth after cesarean | Obstetric labor | Retrospective | Tessmer-Tuck JA et al. (2014) | Retrospective | United States | 0 | 1 | 0 | 2000 | 2010 | DV | Yes |
| Theriault S et al. (2016) | Gestational diabetes | Gestational diabetes | Nested case-control | Theriault S et al. (2016) | Nested Case-Control | Canada | 0 | 1 | 0 | 2005 | 2010 | D | Yes |
| Timmerman E et al. (2010) | Chromosomal anomalies | Trisomy | Cross-sectional | Timmerman E et al. (2010) | Cross-Sectional | Netherlands | 0 | 1 | 0 | 1996 | 2008 | D | Yes |
| Tran D et al. (2019) | Ongoing pregnancy | Fertilization *in vitro* | Retrospective | Tran D et al. (2019) | Retrospective | Australia, Ireland, Denmark, United Kingdom | 8 | 0 | 0 | 2014 | 2018 | D | Yes |
| Troisi J et al. (2018) | Central nervous system anomalies | Central nervous system vascular malformations | Prospective | Troisi J et al. (2018) | Prospective | Italy | 0 | 3 | 0 | 2013 | 2017 | D | Yes |
| Tsur A et al. (2019) | Shoulder dystocia | Obstetric labor | Case-control | Tsur A et al. (2019) | Case-Control | Israel, United States | 0 | 2 | 0 | 2003 | 2018 | DV | Yes |
| Uyar A et al. (2010) | Successful implantation (definition not reported) | Fertilization *in vitro* | Retrospective | Uyar A et al. (2010) | Retrospective | Turkey | 0 | 1 | 0 | 2007 | 2008 | D | Yes |
| Uyar A et al. (2015) | Ongoing pregnancy | Fertilization *in vitro* | Retrospective | Uyar A et al. (2015) | Retrospective | Turkey | 0 | 1 | 0 | 2007 | 2008 | D | No |
| Valensise H et al. (2006) | Fetal distress | Fetal distress | Cross-sectional | Valensise H et al. (2006) | Cross-Sectional | Italy | 0 | 1 | 0 | 1999 | 2003 | D | No |
| van Baaren GJ et al. (2015) | Preterm delivery | Premature birth | Retrospective | van Baaren GJ et al. (2014) | Prospective | Netherlands | 10 | 0 | 0 | 2009 | 2012 | D | Yes |
| van Calster B et al. (2009) | Pregnancy of unknown location | Others | Retrospective | van Calster B et al. (2009) | Retrospective | United Kingdom | 0 | 1 | 0 | 2001 | 2004 | DV | Yes |
| van der Ham DP et al. (2014) | Neonatal sepsis | Infectious pregnancy complication | Retrospective | van der Ham DP, van der Heyden JL, Opmeer BC, et al. (2012); van der Ham DP, Vijgen SM, Nijhuis JG, et al (2012) | Prospective | Netherlands | 0 | 60 | 0 | 2007 | 2011 | D | Yes |
| van der Tuuk K et al. (2015) | Cesarean section | Cesarean section | Retrospective | Koopmans CM et al. (2009) | Prospective | Netherlands | 0 | 38 | 0 | 2005 | 2008 | D | Yes |
| Verhoeven CJ et al. (2016) | Failure of vacuum-assisted vaginal delivery | Obstetric labor | Case-control | Verhoeven CJ et al. (2016) | Case-Control | Netherlands | 0 | 2 | 0 | 1997 | 2011 | D | Yes |
| VerMilyea M et al. (2020) | Ongoing pregnancy | Fertilization *in vitro* | Retrospective | VerMilyea M et al. (2020) | Retrospective | United States, Australia and New Zealand | 11 | 0 | 0 | 2011 | 2018 | D | No |
| Vieira MC et al. (2017) | Uncomplicated pregnancy | Others | Retrospective | Poston L et al. (2015) | Prospective | United Kingdom | 0 | 8 | 0 | 2009 | 2014 | D | Yes |
| Visentin S et al. (2017) | Late gestational hypertension | Pregnancy-induced hypertension | Prospective | Visentin S et al. (2017) | Prospective | Italy | 0 | 1 | 0 | 2012 | 2014 | D | Yes |
| Vogiatzi P et al. (2019) | Live birth | Fertilization *in vitro* | Retrospective | Vogiatzi P et al. (2019) | Retrospective | Greece | 0 | 1 | 0 | 2010 | 2017 | D | No |
| Wald M et al. (2005) | Ongoing pregnancy | Fertilization *in vitro* | Retrospective | Wald M et al. (2005) | Retrospective |  |  |  |  |  |  | D | No |
| Wang C et al. (2016) | Gestational diabetes | Gestational diabetes | Retrospective | Zhu WW et al. (2017) | Retrospective | China | 0 | 15 | 0 | 2013 | 2013 | D | Yes |
| Wang L et al. (2016) | Adverse maternal and neonatal outcomes | Others | Retrospective | Wang L et al. (2016) | Retrospective | Japan | 0 | 1 | 0 | 2004 | 2010 | D | No |
| Weber A et al. (2018) | Preterm delivery | Premature birth | Case-control | Weber A et al. (2018) | Case-Control | United States |  |  |  | 2007 | 2011 | D | Yes |
| Xing YP et al. (2019) | Vaginal birth after cesarean | Obstetric labor | Retrospective | Xing YP et al. (2019) | Retrospective | China | 0 | 1 | 0 | 2012 | 2016 | D | Yes |
| Xu H et al. (2019) | Ongoing pregnancy | Fertilization *in vitro* | Prospective | Xu H et al. (2019) | Prospective | China | 0 | 1 | 0 | 2015 | 2016 | D | No |
| Xu H et al. (2020) | Ectopic pregnancy | Others | Prospective | Xu H et al. (2019) | Prospective | China | 0 | 1 | 0 | 2015 | 2016 | D | No |
| Xu L et al. (2013) | Acidotic blood pH of umbilical artery | Fetal distress | Prospective | Xu H et al. (2019) | Prospective | China | 0 | 1 | 0 | 2015 | 2016 | D | No |
| Yang H et al. (2015) | Gestational diabetes | Gestational diabetes | Retrospective | Yang H et al. (2015) | Retrospective | China | 0 | 1 | 0 | 2009 | 2010 | D | Yes |
| Yang T et al. (2019) | Placenta accreta | Others | Retrospective | Yang T et al. (2019) | Retrospective | China | 0 | 1 | 0 | 2013 | 2017 | D | No |
| Yu CH et al. (2018) | High-quality blastocyst | Embryo implantation | Retrospective | Yu CH et al. (2018) | Retrospective | China | 0 | 1 | 0 | 2013 | 2016 | D | Yes |
| Zhao RF et al. (2019) | Vaginal delivery | Obstetric labor | Retrospective | Zhao RF et al. (2019) | Retrospective | China | 0 | 2 | 0 | 2014 | 2018 | DV | No |
| Zheng T et al. (2019) | Gestational diabetes | Gestational diabetes | Retrospective | Zheng T et al. (2019) | Retrospective | China | 0 | 1 | 0 | 2015 | 2017 | D | Yes |
| Zwertbroek EF et al. (2017) | Severe disease of late preterm pregnancy-induced hypertension | Pregnancy-induced hypertension | Retrospective | Broekhuijsen K et al. (2016) | Prospective | Netherlands | 0 | 51 | 0 | 2009 | 2013 | D | Yes |

DV, development and/or validation; M, meta-analysis.

# Table S6. Risk of bias assessment.

| Study Author (Publication Year) | Development Risk of Bias | | | | | Validation Risk of Bias | | | | |
| --- | --- | --- | --- | --- | --- | --- | --- | --- | --- | --- |
|  | Participants | Predictors | Outcome | Analysis | ROB | Participants | Predictors | Outcome | Analysis | ROB |
|  |  |  |  |  |  |  |  |  |  |  |
| Abbas SA et al. (2018) | - | - | ? | - | - |  |  |  |  |  |
| Agopian AJ et al. (2012) | + | + | + | - | - |  |  |  |  |  |
| Alberola-Rubio J et al. (2017) | + | + | + | - | - |  |  |  |  |  |
| Allouche M et al. (2011) | + | + | + | - | - | + | + | + | - | - |
| Almeida ST et al. (2017) | + | ? | + | - | - | + | ? | + | ? | ? |
| Al-Rubaie ZT et al. (2020) | + | - | - | - | - | + | + | + | - | - |
| Amini P et al. (2017) | - | + | - | - | - |  |  |  |  |  |
| Artzi NS et al. (2020) | + | + | + | + | + | + | + | + | + | + |
| Balani J et al. (2018) | + | + | ? | - | - |  |  |  |  |  |
| Bastek JA et al. (2012) | + | - | - | - | - |  |  |  |  |  |
| Benalcazar-Parra C et al. (2019) | + | + | + | - | - |  |  |  |  |  |
| Benhalima K et al. (2020) | - | - | + | - | - |  |  |  |  |  |
| Berntorp K et al. (2015) | - | + | + | - | - |  |  |  |  |  |
| Blank C et al. (2019) | + | + | + | + | + |  |  |  |  |  |
| Borup R et al. (2016) | + | + | + | + | + | + | + | + | + | + |
| Broekmans FJ et al. (2014) | + | + | - | - | - | + | + | - | - | - |
| Carlsson FM et al. (2020) | + | + | + | - | - |  |  |  |  |  |
| Casikar I et al. (2013) | + | - | - | - | - | + | + | + | - | - |
| Cerqueira FR et al. (2014) | + | + | + | + | + |  |  |  |  |  |
| Chandrasekaran S et al. (2016) | + | + | + | - | - |  |  |  |  |  |
| Chen L and Hao Y (2017) | + | ? | ? | - | - |  |  |  |  |  |
| Chen L et al. (2018) | - | + | + | - | - | - | + | + | - | - |
| Chen L et al. (2019) | + | + | + | - | - |  |  |  |  |  |
| Ciobanu A et al. (2019) | + | + | + | - | - |  |  |  |  |  |
| Comert Z et al. (2018) | + | + | + | + | + |  |  |  |  |  |
| Coppede F et al. (2010) | + | + | + | - | - |  |  |  |  |  |
| Cortet M et al. (2015) | + | - | - | - | - |  |  |  |  |  |
| Crovetto F et al. (2015) | + | + | + | - | - |  |  |  |  |  |
| de Oliveira RV et al. (2012) | + | + | + | - | - |  |  |  |  |  |
| de Wilde MA et al. (2014) | + | + | + | - | - |  |  |  |  |  |
| Despotovic D et al. (2018) | + | + | + | + | + |  |  |  |  |  |
| Eggebo TM et al. (2015) | - | + | + | - | - |  |  |  |  |  |
| Elaveyni U et al. (2011) | + | + | + | - | - |  |  |  |  |  |
| Fagerberg MC et al. (2015) | + | + | - | - | - | + | + | + | - | - |
| Fergus P et al. (2013) | + | + | + | + | + |  |  |  |  |  |
| Fergus P et al. (2016) | + | + | + | + | + |  |  |  |  |  |
| Fergus P et al. (2017) | + | + | + | + | + |  |  |  |  |  |
| Fergus P et al. (2018) | + | + | + | + | + |  |  |  |  |  |
| Figueras F et al. (2015) | + | - | - | - | - |  |  |  |  |  |
| Fiset S et al. (2019) | + | + | + | - | - |  |  |  |  |  |
| Gao C et al. (2019) | + | + | + | - | - | + | + | + | - | - |
| Garces MF et al. (2015) | + | + | - | - | - |  |  |  |  |  |
| Georgulas G, Karvelis P, Spilka J et al. (2017) | + | ? | ? | + | ? |  |  |  |  |  |
| Guo Z et al. (2020) | + | + | - | - | - | + | + | + | - | - |
| Hamdi MA et al. (2019) | + | + | + | - | - |  |  |  |  |  |
| Harper LM et al. (2016) | + | - | - | - | - |  |  |  |  |  |
| Hernandez-Gonzalez J et al. (2018) | + | + | + | - | - |  |  |  |  |  |
| Isakov O et al. (2019) | + | + | + | - | - |  |  |  |  |  |
| Isono W et al. (2011) | + | ? | + | - | - |  |  |  |  |  |
| Jhee JH et al. (2019) | + | + | + | - | - |  |  |  |  |  |
| Kang J et al. (2020) | + | ? | ? | - | - | + | ? | ? | - | - |
| Kawakita T et al. (2019) | + | + | - | - | - |  |  |  |  |  |
| Khan N et al. (2019) | + | + | - | - | - |  |  |  |  |  |
| Koivu A et al. (2020) | + | + | + | - | - | + | + | + | - | - |
| Kok M et al. (2011) | + | + | ? | - | - |  |  |  |  |  |
| Kuhle S et al. (2018) | + | + | + | - | - |  |  |  |  |  |
| Kumar SN et al. (2020) | - | + | + | - | - | - | + | + | - | - |
| Lafalla O et al. (2019) | - | - | - | - | - |  |  |  |  |  |
| Lee JSE et al. (2018) | + | + | + | - | - | + | + | + | - | - |
| Lee KS et al. (2019) | + | + | + | - | - |  |  |  |  |  |
| Leonarduzzi R et al. (2015) | + | + | + | + | + |  |  |  |  |  |
| Li H et al. (2017) | - | - | - | - | - |  |  |  |  |  |
| Liu B et al. (2019) | + | ? | ? | - | - |  |  |  |  |  |
| Macones GA et al. (2001) | - | + | + | - | - |  |  |  |  |  |
| Mardy AH et al. (2016) | + | - | - | - | - |  |  |  |  |  |
| Maroufizadeh S et al. (2018) | + | - | - | - | - |  |  |  |  |  |
| Mas-Cabo J et al. (2019) | + | + | + | - | - |  |  |  |  |  |
| McCowan LM et al. (2013) | + | + | + | - | - |  |  |  |  |  |
| McCowan LM et al. (2017) | + | + | + | - | - |  |  |  |  |  |
| Mehta-Lee SS et al. (2017) | - | - | - | - | - |  |  |  |  |  |
| Meijerink AM et al. (2016) | + | + | + | + | + | + | + | + | + | + |
| Meister MR et al. (2016) | + | + | + | + | + |  |  |  |  |  |
| Mello G et al. (2001) | + | + | + | - | - |  |  |  |  |  |
| Menon R et al. (2014) | + | + | + | + | + |  |  |  |  |  |
| Metz TD et al. (2013) | + | - | - | - | - | + | - | - | + | - |
| Milewski R et al. (2013) | + | + | + | - | - |  |  |  |  |  |
| Milewski R et al. (2017) | ? | + | + | - | - |  |  |  |  |  |
| Mirroshandel SA et al. (2016) | + | + | + | + | + |  |  |  |  |  |
| Morales DA et al. (2008) | + | + | + | - | - |  |  |  |  |  |
| Murtoniemi K et al. (2018) | + | + | + | - | - |  |  |  |  |  |
| Myers JE et al. (2013) | + | + | + | - | - |  |  |  |  |  |
| Oates J et al. (2013) | + | + | + | - | - |  |  |  |  |  |
| Paydar K et al. (2017) | ? | + | ? | - | - |  |  |  |  |  |
| Payne BA et al. (2015) | + | + | + | - | - |  |  |  |  |  |
| Petrozzielo A et al. (2018) | + | + | + | - | - |  |  |  |  |  |
| Petrozzielo A et al. (2019) | + | + | + | - | - |  |  |  |  |  |
| Pettersson G et al. (2010) | + | + | + | - | - |  |  |  |  |  |
| Pettersson G et al. (2017) | + | - | - | - | - |  |  |  |  |  |
| Qiu H et al. (2017) | + | + | + | - | - |  |  |  |  |  |
| Qiu K et al. (2019) | + | + | + | - | - |  |  |  |  |  |
| Ramanah R et al. (2018) | + | + | + | + | + |  |  |  |  |  |
| Reid S et al. (2015) | + | + | + | - | - |  |  |  |  |  |
| Rinaudo P et al. (2012) | ? | + | + | - | - |  |  |  |  |  |
| Ryu A et al. (2019) | - | + | + | - | - |  |  |  |  |  |
| Sadi-Ahmed N et al. (2017) | + | + | + | + | + |  |  |  |  |  |
| Saleem S et al. (2019) | + | + | + | + | + |  |  |  |  |  |
| Sananes N et al. (2013) | + | - | - | - | - | + | - | - | - | - |
| Sandstrom A et al. (2019) | + | + | + | - | - |  |  |  |  |  |
| Scheinhardt MO et al. (2018) |  |  |  |  |  | - | + | + | - | - |
| Shahbakhti M et al. (2019) | + | + | + | - | - |  |  |  |  |  |
| Shi W et al. (2013) | + | + | + | - | - |  |  |  |  |  |
| Signorini MG et al. (2020) | - | + | - | - | - |  |  |  |  |  |
| Sims CJ et al. (2000) | + | + | + | - | - |  |  |  |  |  |
| Sovio U et al. (2018) | - | + | + | - | - |  |  |  |  |  |
| Spilka J et al. (2015) | + | + | + | + | + |  |  |  |  |  |
| Spilka J et al. (2017) | + | + | + | - | - |  |  |  |  |  |
| Stamatopoulos N et al. (2015) | - | - | - | - | - | - | - | - | - | - |
| Stott D et al. (2017) | + | - | - | - | - |  |  |  |  |  |
| Stroux L et al. (2017) | ? | + | + | - | - |  |  |  |  |  |
| Sufriyana H et al. (2020) | + | + | + | + | + | + | + | + | + | + |
| Tessmer-Tuck JA et al. (2014) | + | + | + | - | - | + | + | + | - | - |
| Theriault S et al. (2016) | + | + | + | - | - |  |  |  |  |  |
| Timmerman E et al. (2010) | + | - | - | - | - |  |  |  |  |  |
| Tran D et al. (2019) | + | + | + | - | - |  |  |  |  |  |
| Troisi J et al. (2018) | + | + | + | + | + |  |  |  |  |  |
| Tsur A et al. (2019) | - | + | + | - | - | + | + | + | - | - |
| Uyar A et al. (2010) | ? | - | ? | - | - |  |  |  |  |  |
| Uyar A et al. (2015) | + | - | + | - | - |  |  |  |  |  |
| Valensise H et al. (2006) | + | + | + | - | - |  |  |  |  |  |
| van Baaren GJ et al. (2015) | + | + | + | - | - |  |  |  |  |  |
| van Calster B et al. (2009) | + | + | + | - | - | + | + | + | - | - |
| van der Ham DP et al. (2014) | + | + | + | - | - |  |  |  |  |  |
| van der Tuuk K et al. (2015) | + | - | - | - | - |  |  |  |  |  |
| Verhoeven CJ et al. (2016) | - | + | + | - | - |  |  |  |  |  |
| VerMilyea M et al. (2020) | + | + | + | - | - |  |  |  |  |  |
| Vieira MC et al. (2017) | + | - | - | - | - |  |  |  |  |  |
| Visentin S et al. (2017) | + | + | + | - | - |  |  |  |  |  |
| Vogiatzi P et al. (2019) | + | + | + | - | - |  |  |  |  |  |
| Wald M et al. (2005) | - | ? | ? | - | - |  |  |  |  |  |
| Wang C et al. (2016) | + | + | + | - | - |  |  |  |  |  |
| Wang L et al. (2016) | + | + | + | - | - |  |  |  |  |  |
| Weber A et al. (2018) | - | + | + | + | - |  |  |  |  |  |
| Xing YP et al. (2019) | - | - | - | - | - |  |  |  |  |  |
| Xu H et al. (2019) | + | + | + | - | - |  |  |  |  |  |
| Xu H et al. (2020) | + | + | + | + | + |  |  |  |  |  |
| Xu L et al. (2013) | + | + | + | + | + |  |  |  |  |  |
| Yang H et al. (2015) | - | + | + | - | - |  |  |  |  |  |
| Yang T et al. (2019) | + | + | + | - | - |  |  |  |  |  |
| Yu CH et al. (2018) | + | - | - | - | - |  |  |  |  |  |
| Zhao RF et al. (2019) | + | + | + | - | - | + | + | + | - | - |
| Zheng T et al. (2019) | + | + | + | + | + |  |  |  |  |  |
| Zwertbroek EF et al. (2017) | + | - | - | - | - |  |  |  |  |  |

+, low risk of bias; -, high risk of bias; ?, unclear risk of bias. ROB, risk of bias (overall).

# Table S7. Signaling questions.

| Domain # | Term | Signaling question |
| --- | --- | --- |
|  |  |  |
| Participant Q1.1 | Study Design | Were appropriate data sources used, e.g. cohort, RCT or nested case-control study data? |
| Participant Q1.2 | Selection Criteria | Were all inclusions and exclusions of participants appropriate? |
| Predictors Q2.1 | Predictor Assessment | Were predictors defined and assessed in a similar way for all participants? |
| Predictors Q2.2 | Predictor Blinding | Were predictor assessments made without knowledge of outcome data? |
| Predictors Q2.3 | Predictor Timing | Are all predictors available at the time the model is intended to be used? |
| Outcome Q3.1 | Outcome Definition | Was the outcome determined appropriately? |
| Outcome Q3.2 | Outcome Standard | Was a pre-specified or standard outcome definition used? |
| Outcome Q3.3 | Outcome Leakage | Were predictors excluded from the outcome definition? |
| Outcome Q3.4 | Outcome Assessment | Was the outcome defined and determined in a similar way for all participants? |
| Outcome Q3.5 | Outcome Blinding | Was the outcome determined without knowledge of predictor information? |
| Outcome Q3.6 | Time Interval | Was the time interval between predictor assessment and outcome determination appropriate? |
| Analysis Q4.1 | Events per Variable | Were there a reasonable number  of participants with the outcome? |
| Analysis Q4.2 | Data Scale Handling | Were continuous and categorical predictors handled appropriately? |
| Analysis Q4.3 | Post-Enrollment Selection | Were all enrolled participants included in the analysis? |
| Analysis Q4.4 | Missing Data Handling | Were participants with missing data handled appropriately? |
| Analysis Q4.5 | Predictor Selection | Was selection of predictors based on univariable analysis avoided? |
| Analysis Q4.6 | Data Complexities Handling | Were complexities in the data (e.g. censoring, competing risks, sampling of controls) accounted for appropriately? |
| Analysis Q4.7 | Calibration And Discrimination | Were relevant model performance measures evaluated appropriately? |
| Analysis.8 | Internal/External Validation | Were model overfitting and optimism in model performance accounted for? |
| Analysis.9 | Predictor Weights | Do predictors and their assigned weights in the final model correspond to the results from multivariable analysis? |

# Table S8. Answer for each signaling question.

| Study Author (Publication Year) | Development Risk of Bias | | | | | | | | | | | | | | | | | | | | | | | | | | | | Validation Risk of Bias | | | | | | | | |
| --- | --- | --- | --- | --- | --- | --- | --- | --- | --- | --- | --- | --- | --- | --- | --- | --- | --- | --- | --- | --- | --- | --- | --- | --- | --- | --- | --- | --- | --- | --- | --- | --- | --- | --- | --- | --- | --- |
|  | Q.1.1 | Q.1.2 | Q.2.1 | Q.2.2 | Q.2.3 | Q.3.1 | Q.3.2 | Q.3.3 | Q.3.4 | Q.3.5 | Q.3.6 | Q.4.1 | Q.4.2 | Q.4.3 | Q.4.4 | Q.4.5 | Q.4.6 | Q.4.7 | Q.4.8 | Q.4.9 | Q.1.1 | Q.1.2 | Q.2.1 | Q.2.2 | Q.2.3 | Q.3.1 | Q.3.2 | Q.3.3 | Q.3.4 | Q.3.5 | Q.3.6 | Q.4.1 | Q.4.2 | Q.4.3 | Q.4.4 | Q.4.6 | Q.4.7 |
|  |  |  |  |  |  |  |  |  |  |  |  |  |  |  |  |  |  |  |  |  |  |  |  |  |  |  |  |  |  |  |  |  |  |  |  |  |  |
| Abbas SA et al. (2018) | N | NI | PN | NI | NI | NI | NI | NI | NI | NI | NI | N | NI | N | NI | NI | NI | Y | Y | NI |  |  |  |  |  |  |  |  |  |  |  |  |  |  |  |  |  |
| Agopian AJ et al. (2012) | Y | PY | Y | PY | Y | Y | PY | Y | PY | Y | Y | Y | N | Y | PN | Y | Y | N | Y | PN |  |  |  |  |  |  |  |  |  |  |  |  |  |  |  |  |  |
| Alberola-Rubio J et al. (2017) | Y | Y | Y | Y | Y | Y | Y | Y | Y | Y | Y | N | Y | Y | Y | Y | Y | N | Y | Y |  |  |  |  |  |  |  |  |  |  |  |  |  |  |  |  |  |
| Allouche M et al. (2011) | Y | Y | Y | Y | PY | Y | PY | PY | PY | Y | Y | N | PY | Y | Y | Y | PY | Y | Y | Y | Y | Y | Y | Y | PY | Y | PY | PY | PY | Y | Y | N | PY | Y | Y | PY | Y |
| Almeida ST et al. (2017) | Y | Y | Y | Y | NI | Y | PY | PY | PY | Y | PY | Y | Y | Y | PY | N | NI | Y | N | Y | Y | Y | Y | Y | NI | Y | PY | PY | PY | Y | PY | N | Y | Y | PY | NI | Y |
| Al-Rubaie ZT et al. (2020) | Y | Y | PY | PN | Y | PY | Y | PN | PY | PN | PY | N | PY | Y | Y | Y | PY | Y | Y | Y | Y | Y | PY | PN | Y | PY | Y | PN | PY | PN | PY | N | PY | Y | Y | PY | Y |
| Amini P et al. (2017) | N | NI | Y | PY | Y | Y | Y | PN | Y | Y | Y | N | Y | PY | Y | Y | N | Y | N | Y |  |  |  |  |  |  |  |  |  |  |  |  |  |  |  |  |  |
| Artzi NS et al. (2020) | PY | PY | PY | PY | Y | Y | Y | PY | Y | PY | Y | PY | Y | PY | PY | Y | PY | Y | Y | Y | PY | PY | PY | PY | Y | Y | Y | PY | Y | PY | Y | Y | Y | PY | PY | PY | Y |
| Balani J et al. (2018) | Y | Y | Y | Y | PY | Y | PY | PY | PY | Y | NI | N | PY | Y | Y | Y | NI | N | N | Y |  |  |  |  |  |  |  |  |  |  |  |  |  |  |  |  |  |
| Bastek JA et al. (2012) | Y | PY | PY | PN | PY | Y | Y | PN | PY | PY | Y | N | PY | Y | Y | Y | PY | Y | N | N |  |  |  |  |  |  |  |  |  |  |  |  |  |  |  |  |  |
| Benalcazar-Parra C et al. (2019) | Y | Y | Y | Y | Y | Y | Y | PY | PY | PY | PY | N | Y | Y | PN | Y | PN | Y | N | N |  |  |  |  |  |  |  |  |  |  |  |  |  |  |  |  |  |
| Benhalima K et al. (2020) | Y | PN | Y | PN | Y | Y | Y | Y | Y | Y | Y | Y | PY | Y | PY | Y | PN | Y | N | N |  |  |  |  |  |  |  |  |  |  |  |  |  |  |  |  |  |
| Berntorp K et al. (2015) | Y | PN | Y | PY | Y | Y | Y | Y | PY | PY | Y | Y | PY | Y | Y | Y | N | Y | PN | N |  |  |  |  |  |  |  |  |  |  |  |  |  |  |  |  |  |
| Blank C et al. (2019) | Y | PY | Y | Y | Y | Y | Y | Y | Y | Y | PY | PY | PY | Y | Y | Y | Y | Y | Y | Y |  |  |  |  |  |  |  |  |  |  |  |  |  |  |  |  |  |
| Borup R et al. (2016) | Y | Y | Y | Y | Y | Y | Y | Y | Y | Y | PY | PY | Y | Y | Y | Y | Y | Y | Y | Y | Y | Y | Y | Y | Y | Y | Y | Y | Y | Y | PY | PY | Y | Y | Y | Y | Y |
| Broekmans FJ et al. (2014) | Y | Y | Y | Y | Y | Y | Y | PN | Y | Y | Y | N | Y | Y | Y | Y | N | Y | Y | Y | Y | Y | Y | Y | Y | Y | Y | PN | Y | Y | Y | N | Y | Y | Y | N | Y |
| Carlsson FM et al. (2020) | Y | Y | Y | Y | Y | PY | PY | Y | Y | Y | Y | N | PY | Y | N | Y | N | Y | PN | Y |  |  |  |  |  |  |  |  |  |  |  |  |  |  |  |  |  |
| Casikar I et al. (2013) | Y | Y | PY | N | Y | PY | PY | PN | Y | N | Y | Y | Y | Y | Y | Y | N | Y | PN | Y | Y | PY | PY | N | Y | PY | PY | PN | Y | N | Y | Y | Y | Y | Y | N | Y |
| Cerqueira FR et al. (2014) | Y | Y | PY | Y | Y | Y | Y | Y | Y | Y | PY | PY | Y | Y | Y | Y | PY | Y | Y | Y |  |  |  |  |  |  |  |  |  |  |  |  |  |  |  |  |  |
| Chandrasekaran S et al. (2016) | Y | Y | Y | Y | Y | Y | Y | Y | Y | Y | Y | N | Y | Y | Y | Y | N | Y | N | Y |  |  |  |  |  |  |  |  |  |  |  |  |  |  |  |  |  |
| Chen L and Hao Y (2017) | Y | Y | NI | Y | Y | NI | NI | NI | NI | Y | Y | Y | Y | Y | Y | Y | Y | Y | Y | N |  |  |  |  |  |  |  |  |  |  |  |  |  |  |  |  |  |
| Chen L et al. (2018) | PN | Y | Y | Y | Y | PY | Y | Y | Y | Y | Y | N | Y | N | PY | Y | Y | Y | Y | Y | PN | Y | Y | Y | Y | PY | Y | Y | Y | Y | Y | N | Y | N | PY | Y | Y |
| Chen L et al. (2019) | Y | Y | Y | Y | Y | Y | Y | Y | Y | Y | Y | Y | Y | Y | Y | Y | Y | N | N | NI |  |  |  |  |  |  |  |  |  |  |  |  |  |  |  |  |  |
| Ciobanu A et al. (2019) | PY | Y | Y | Y | Y | Y | Y | Y | Y | Y | Y | Y | Y | Y | PY | Y | Y | N | N | PY |  |  |  |  |  |  |  |  |  |  |  |  |  |  |  |  |  |
| Comert Z et al. (2018) | Y | Y | Y | Y | Y | Y | Y | Y | Y | Y | Y | PY | Y | Y | PY | Y | Y | PY | Y | PY |  |  |  |  |  |  |  |  |  |  |  |  |  |  |  |  |  |
| Coppede F et al. (2010) | PY | Y | Y | Y | Y | PY | PY | Y | Y | Y | Y | PN | Y | PY | PY | Y | Y | N | PY | PY |  |  |  |  |  |  |  |  |  |  |  |  |  |  |  |  |  |
| Cortet M et al. (2015) | PY | Y | PN | PY | Y | Y | PY | PY | PY | PN | Y | Y | Y | PY | PY | N | PN | Y | Y | PY |  |  |  |  |  |  |  |  |  |  |  |  |  |  |  |  |  |
| Crovetto F et al. (2015) | Y | PY | PY | Y | Y | Y | Y | Y | Y | Y | Y | N | Y | Y | N | Y | PY | N | N | PY |  |  |  |  |  |  |  |  |  |  |  |  |  |  |  |  |  |
| de Oliveira RV et al. (2012) | Y | Y | Y | Y | Y | Y | Y | PY | Y | Y | Y | N | PY | Y | Y | Y | N | Y | N | Y |  |  |  |  |  |  |  |  |  |  |  |  |  |  |  |  |  |
| de Wilde MA et al. (2014) | Y | Y | Y | Y | Y | Y | Y | Y | Y | Y | Y | N | Y | Y | Y | N | PY | Y | Y | Y |  |  |  |  |  |  |  |  |  |  |  |  |  |  |  |  |  |
| Despotovic D et al. (2018) | Y | PY | Y | Y | Y | Y | Y | Y | Y | Y | Y | Y | Y | PY | PY | Y | PY | Y | Y | Y |  |  |  |  |  |  |  |  |  |  |  |  |  |  |  |  |  |
| Eggebo TM et al. (2015) | Y | PN | Y | Y | Y | Y | Y | Y | Y | Y | Y | N | Y | Y | Y | Y | N | Y | PN | Y |  |  |  |  |  |  |  |  |  |  |  |  |  |  |  |  |  |
| Elaveyni U et al. (2011) | Y | Y | PY | PY | Y | Y | Y | PY | Y | PY | Y | N | Y | Y | Y | Y | N | Y | N | N |  |  |  |  |  |  |  |  |  |  |  |  |  |  |  |  |  |
| Fagerberg MC et al. (2015) | Y | Y | PY | Y | Y | Y | Y | PN | Y | Y | Y | Y | Y | Y | PN | Y | Y | Y | Y | Y | Y | Y | PY | Y | Y | Y | Y | PN | Y | Y | Y | Y | Y | Y | PN | Y | Y |
| Fergus P et al. (2013) | Y | PY | Y | Y | Y | Y | Y | Y | Y | Y | Y | Y | Y | PY | PY | Y | PY | Y | Y | Y |  |  |  |  |  |  |  |  |  |  |  |  |  |  |  |  |  |
| Fergus P et al. (2016) | Y | PY | Y | Y | Y | Y | Y | Y | Y | Y | Y | Y | Y | PY | PY | Y | PY | Y | Y | Y |  |  |  |  |  |  |  |  |  |  |  |  |  |  |  |  |  |
| Fergus P et al. (2017) | Y | Y | Y | Y | Y | Y | Y | Y | PY | PY | Y | Y | Y | Y | Y | Y | PY | Y | Y | Y |  |  |  |  |  |  |  |  |  |  |  |  |  |  |  |  |  |
| Fergus P et al. (2018) | Y | Y | Y | Y | Y | Y | Y | Y | Y | Y | Y | Y | Y | Y | Y | Y | Y | Y | PY | PY |  |  |  |  |  |  |  |  |  |  |  |  |  |  |  |  |  |
| Figueras F et al. (2015) | Y | Y | Y | PN | Y | Y | Y | PN | Y | PN | Y | N | PY | Y | Y | Y | N | Y | Y | Y |  |  |  |  |  |  |  |  |  |  |  |  |  |  |  |  |  |
| Fiset S et al. (2019) | Y | Y | Y | Y | Y | Y | Y | Y | Y | Y | PY | PY | Y | Y | Y | N | Y | PY | N | Y |  |  |  |  |  |  |  |  |  |  |  |  |  |  |  |  |  |
| Gao C et al. (2019) | Y | PY | Y | Y | Y | Y | Y | PY | Y | Y | Y | N | PN | Y | Y | Y | PY | Y | Y | Y | Y | PY | Y | Y | Y | Y | Y | PY | Y | Y | Y | N | PN | Y | Y | PY | Y |
| Garces MF et al. (2015) | Y | Y | Y | Y | Y | Y | Y | PN | Y | Y | Y | N | Y | Y | NI | Y | N | Y | Y | Y |  |  |  |  |  |  |  |  |  |  |  |  |  |  |  |  |  |
| Georgulas G, Karvelis P, Spilka J et al. (2017) | Y | Y | NI | Y | Y | NI | NI | NI | NI | Y | Y | Y | Y | Y | Y | Y | Y | Y | Y | Y |  |  |  |  |  |  |  |  |  |  |  |  |  |  |  |  |  |
| Guo Z et al. (2020) | Y | Y | Y | Y | Y | Y | Y | PN | PY | Y | Y | N | Y | Y | Y | Y | N | N | Y | Y | Y | Y | Y | Y | Y | Y | Y | PY | PY | Y | Y | N | Y | Y | Y | N | N |
| Hamdi MA et al. (2019) | Y | PY | Y | PY | Y | Y | Y | Y | Y | PY | Y | Y | Y | Y | PY | Y | PY | N | PY | PY |  |  |  |  |  |  |  |  |  |  |  |  |  |  |  |  |  |
| Harper LM et al. (2016) | Y | Y | Y | PN | Y | Y | Y | PN | PY | PY | Y | N | Y | Y | Y | Y | N | Y | Y | Y |  |  |  |  |  |  |  |  |  |  |  |  |  |  |  |  |  |
| Hernandez-Gonzalez J et al. (2018) | Y | PY | Y | Y | Y | Y | Y | Y | Y | Y | PY | N | N | N | Y | Y | Y | Y | Y | Y |  |  |  |  |  |  |  |  |  |  |  |  |  |  |  |  |  |
| Isakov O et al. (2019) | Y | Y | PY | Y | PY | Y | PY | Y | PY | Y | Y | N | Y | Y | Y | Y | PY | Y | Y | Y |  |  |  |  |  |  |  |  |  |  |  |  |  |  |  |  |  |
| Isono W et al. (2011) | Y | Y | NI | PY | Y | PY | PY | Y | PY | Y | Y | N | Y | PY | NI | Y | PY | Y | Y | Y |  |  |  |  |  |  |  |  |  |  |  |  |  |  |  |  |  |
| Jhee JH et al. (2019) | Y | Y | Y | Y | Y | Y | Y | Y | Y | Y | Y | N | Y | Y | Y | Y | Y | PN | N | Y |  |  |  |  |  |  |  |  |  |  |  |  |  |  |  |  |  |
| Kang J et al. (2020) | Y | PY | PY | NI | Y | NI | NI | NI | NI | NI | Y | N | Y | Y | Y | Y | PN | Y | Y | Y | Y | PY | PY | NI | Y | NI | NI | NI | NI | NI | Y | N | Y | Y | Y | PN | Y |
| Kawakita T et al. (2019) | Y | Y | Y | Y | Y | Y | Y | PY | PY | PN | Y | N | Y | Y | Y | Y | N | Y | Y | Y |  |  |  |  |  |  |  |  |  |  |  |  |  |  |  |  |  |
| Khan N et al. (2019) | Y | Y | Y | Y | Y | PY | PY | PN | PY | Y | Y | PY | Y | Y | PY | Y | Y | Y | N | N |  |  |  |  |  |  |  |  |  |  |  |  |  |  |  |  |  |
| Koivu A et al. (2020) | Y | Y | Y | Y | Y | Y | PY | PY | PY | Y | Y | Y | Y | Y | Y | Y | N | Y | Y | Y | Y | Y | Y | Y | Y | Y | PY | PY | PY | Y | Y | Y | Y | Y | Y | N | Y |
| Kok M et al. (2011) | PY | Y | Y | Y | Y | Y | Y | PY | PY | NI | Y | N | Y | Y | Y | Y | N | Y | Y | Y |  |  |  |  |  |  |  |  |  |  |  |  |  |  |  |  |  |
| Kuhle S et al. (2018) | Y | Y | Y | Y | Y | Y | Y | Y | Y | Y | Y | NI | Y | PY | PY | Y | PY | N | Y | Y |  |  |  |  |  |  |  |  |  |  |  |  |  |  |  |  |  |
| Kumar SN et al. (2020) | N | Y | PY | Y | Y | Y | Y | PY | PY | Y | Y | N | Y | Y | Y | Y | N | Y | Y | Y | N | NI | PY | Y | Y | Y | Y | PY | PY | Y | Y | N | Y | Y | Y | N | Y |
| Lafalla O et al. (2019) | N | N | PY | PN | Y | Y | Y | PN | PY | PN | Y | N | N | Y | PN | Y | N | Y | Y | Y |  |  |  |  |  |  |  |  |  |  |  |  |  |  |  |  |  |
| Lee JSE et al. (2018) | Y | PY | Y | PY | Y | Y | Y | PY | Y | PY | Y | N | Y | Y | Y | Y | N | Y | Y | Y | Y | PY | Y | PY | Y | Y | Y | PY | Y | PY | Y | N | Y | Y | Y | N | Y |
| Lee KS et al. (2019) | Y | PY | Y | Y | Y | Y | Y | PY | PY | Y | Y | N | Y | Y | Y | Y | N | N | N | Y |  |  |  |  |  |  |  |  |  |  |  |  |  |  |  |  |  |
| Leonarduzzi R et al. (2015) | PY | Y | Y | Y | PY | Y | Y | Y | Y | Y | Y | PY | Y | PY | Y | Y | PY | PY | Y | Y |  |  |  |  |  |  |  |  |  |  |  |  |  |  |  |  |  |
| Li H et al. (2017) | N | Y | Y | PN | Y | Y | Y | Y | Y | PN | Y | N | Y | Y | Y | Y | N | Y | N | N |  |  |  |  |  |  |  |  |  |  |  |  |  |  |  |  |  |
| Liu B et al. (2019) | Y | Y | NI | Y | Y | NI | NI | NI | NI | Y | Y | PY | PY | Y | PY | Y | Y | Y | N | N |  |  |  |  |  |  |  |  |  |  |  |  |  |  |  |  |  |
| Macones GA et al. (2001) | N | Y | Y | Y | Y | Y | Y | Y | Y | Y | Y | N | Y | Y | Y | N | N | N | N | Y |  |  |  |  |  |  |  |  |  |  |  |  |  |  |  |  |  |
| Mardy AH et al. (2016) | Y | Y | PY | PN | Y | Y | Y | PY | PY | PN | Y | Y | Y | Y | Y | Y | N | Y | Y | N |  |  |  |  |  |  |  |  |  |  |  |  |  |  |  |  |  |
| Maroufizadeh S et al. (2018) | Y | Y | PY | PN | Y | PY | PY | PY | PY | PN | Y | Y | Y | Y | Y | Y | Y | Y | N | Y |  |  |  |  |  |  |  |  |  |  |  |  |  |  |  |  |  |
| Mas-Cabo J et al. (2019) | Y | PY | PY | Y | Y | PY | PY | PY | PY | Y | Y | Y | Y | Y | Y | Y | PY | Y | Y | N |  |  |  |  |  |  |  |  |  |  |  |  |  |  |  |  |  |
| McCowan LM et al. (2013) | Y | Y | Y | Y | Y | Y | Y | Y | Y | Y | Y | Y | Y | Y | Y | Y | Y | Y | N | Y |  |  |  |  |  |  |  |  |  |  |  |  |  |  |  |  |  |
| McCowan LM et al. (2017) | Y | Y | Y | Y | Y | Y | Y | Y | Y | Y | Y | Y | PN | Y | Y | Y | Y | Y | N | Y |  |  |  |  |  |  |  |  |  |  |  |  |  |  |  |  |  |
| Mehta-Lee SS et al. (2017) | Y | PN | PY | PN | Y | PY | PY | PN | PY | PN | Y | Y | NI | Y | N | Y | PN | Y | Y | PY |  |  |  |  |  |  |  |  |  |  |  |  |  |  |  |  |  |
| Meijerink AM et al. (2016) | Y | Y | Y | Y | Y | Y | Y | Y | Y | Y | Y | Y | Y | Y | Y | Y | Y | Y | Y | Y | Y | Y | Y | Y | Y | Y | Y | Y | Y | Y | Y | Y | Y | Y | Y | Y | Y |
| Meister MR et al. (2016) | Y | Y | Y | Y | Y | Y | Y | Y | Y | Y | Y | Y | Y | Y | PY | Y | Y | Y | Y | Y |  |  |  |  |  |  |  |  |  |  |  |  |  |  |  |  |  |
| Mello G et al. (2001) | Y | Y | Y | Y | Y | Y | Y | Y | Y | Y | Y | N | Y | Y | Y | Y | Y | Y | N | Y |  |  |  |  |  |  |  |  |  |  |  |  |  |  |  |  |  |
| Menon R et al. (2014) | Y | Y | Y | Y | Y | Y | Y | Y | Y | Y | Y | Y | Y | Y | Y | Y | PY | Y | Y | Y |  |  |  |  |  |  |  |  |  |  |  |  |  |  |  |  |  |
| Metz TD et al. (2013) | Y | Y | PY | PN | Y | PY | PY | PN | PY | PN | Y | Y | Y | Y | PN | Y | Y | Y | Y | Y | Y | PY | PY | PN | Y | PY | PY | PN | PY | PN | Y | Y | Y | Y | PN | PY | Y |
| Milewski R et al. (2013) | Y | Y | PY | Y | Y | PY | PY | PY | PY | Y | Y | NI | Y | Y | Y | Y | N | Y | N | Y |  |  |  |  |  |  |  |  |  |  |  |  |  |  |  |  |  |
| Milewski R et al. (2017) | Y | NI | Y | Y | Y | Y | Y | Y | Y | Y | Y | Y | PN | Y | Y | Y | N | Y | N | Y |  |  |  |  |  |  |  |  |  |  |  |  |  |  |  |  |  |
| Mirroshandel SA et al. (2016) | Y | Y | Y | Y | Y | Y | Y | Y | Y | Y | Y | Y | PY | PY | Y | Y | PY | Y | Y | Y |  |  |  |  |  |  |  |  |  |  |  |  |  |  |  |  |  |
| Morales DA et al. (2008) | Y | PY | Y | Y | Y | Y | Y | Y | Y | Y | Y | N | PN | Y | Y | Y | N | Y | Y | Y |  |  |  |  |  |  |  |  |  |  |  |  |  |  |  |  |  |
| Murtoniemi K et al. (2018) | Y | Y | PY | Y | Y | Y | Y | Y | Y | Y | Y | N | Y | Y | N | Y | N | Y | Y | Y |  |  |  |  |  |  |  |  |  |  |  |  |  |  |  |  |  |
| Myers JE et al. (2013) | Y | PY | Y | Y | Y | Y | Y | Y | Y | Y | Y | N | PN | Y | PN | Y | N | Y | Y | Y |  |  |  |  |  |  |  |  |  |  |  |  |  |  |  |  |  |
| Oates J et al. (2013) | Y | Y | PY | Y | Y | Y | Y | Y | Y | Y | Y | N | Y | Y | Y | Y | N | Y | Y | Y |  |  |  |  |  |  |  |  |  |  |  |  |  |  |  |  |  |
| Paydar K et al. (2017) | Y | NI | PY | Y | Y | NI | NI | Y | PY | Y | Y | N | PY | Y | PN | Y | N | N | Y | Y |  |  |  |  |  |  |  |  |  |  |  |  |  |  |  |  |  |
| Payne BA et al. (2015) | Y | Y | PY | Y | Y | PY | PY | Y | PY | Y | Y | N | Y | Y | Y | Y | N | Y | Y | N |  |  |  |  |  |  |  |  |  |  |  |  |  |  |  |  |  |
| Petrozzielo A et al. (2018) | Y | PY | PY | Y | Y | PY | PY | Y | Y | Y | Y | Y | Y | Y | Y | Y | PY | Y | Y | N |  |  |  |  |  |  |  |  |  |  |  |  |  |  |  |  |  |
| Petrozzielo A et al. (2019) | Y | Y | PY | Y | Y | PY | PY | Y | PY | Y | Y | Y | Y | Y | Y | Y | Y | Y | Y | N |  |  |  |  |  |  |  |  |  |  |  |  |  |  |  |  |  |
| Pettersson G et al. (2010) | Y | Y | Y | Y | Y | Y | Y | Y | Y | Y | Y | Y | PN | Y | PN | Y | Y | Y | Y | N |  |  |  |  |  |  |  |  |  |  |  |  |  |  |  |  |  |
| Pettersson G et al. (2017) | Y | Y | PY | N | Y | PY | PY | N | PY | N | Y | N | Y | Y | Y | Y | Y | Y | Y | N |  |  |  |  |  |  |  |  |  |  |  |  |  |  |  |  |  |
| Qiu H et al. (2017) | Y | Y | PY | Y | Y | Y | Y | Y | Y | Y | Y | Y | N | Y | Y | Y | PN | N | Y | NI |  |  |  |  |  |  |  |  |  |  |  |  |  |  |  |  |  |
| Qiu K et al. (2019) | Y | Y | PY | Y | Y | Y | Y | Y | PY | Y | Y | Y | Y | Y | PN | Y | Y | Y | Y | N |  |  |  |  |  |  |  |  |  |  |  |  |  |  |  |  |  |
| Ramanah R et al. (2018) | Y | Y | Y | Y | Y | Y | Y | Y | Y | Y | Y | Y | Y | Y | PY | Y | Y | Y | Y | PY |  |  |  |  |  |  |  |  |  |  |  |  |  |  |  |  |  |
| Reid S et al. (2015) | Y | Y | Y | Y | Y | Y | Y | Y | Y | Y | Y | N | Y | Y | Y | Y | N | Y | Y | N |  |  |  |  |  |  |  |  |  |  |  |  |  |  |  |  |  |
| Rinaudo P et al. (2012) | Y | NI | Y | Y | Y | Y | Y | Y | Y | Y | Y | N | Y | Y | Y | Y | N | Y | Y | N |  |  |  |  |  |  |  |  |  |  |  |  |  |  |  |  |  |
| Ryu A et al. (2019) | Y | N | PY | Y | Y | Y | Y | Y | PY | Y | Y | N | Y | Y | Y | Y | N | Y | Y | Y |  |  |  |  |  |  |  |  |  |  |  |  |  |  |  |  |  |
| Sadi-Ahmed N et al. (2017) | Y | PY | Y | Y | Y | Y | Y | Y | Y | Y | Y | Y | Y | PY | PY | Y | PY | Y | Y | Y |  |  |  |  |  |  |  |  |  |  |  |  |  |  |  |  |  |
| Saleem S et al. (2019) | Y | Y | Y | Y | Y | Y | Y | Y | PY | PY | Y | Y | Y | Y | Y | Y | PY | Y | Y | Y |  |  |  |  |  |  |  |  |  |  |  |  |  |  |  |  |  |
| Sananes N et al. (2013) | Y | Y | Y | PN | Y | PY | PY | N | PY | PN | Y | Y | PY | Y | PN | Y | N | Y | Y | Y | Y | Y | Y | PN | Y | PY | PY | Y | PY | PN | Y | N | PY | Y | PN | N | N |
| Sandstrom A et al. (2019) | Y | Y | PY | Y | Y | PY | PY | Y | PY | Y | Y | Y | Y | Y | Y | Y | N | Y | Y | N |  |  |  |  |  |  |  |  |  |  |  |  |  |  |  |  |  |
| Scheinhardt MO et al. (2018) |  |  |  |  |  |  |  |  |  |  |  |  |  |  |  |  |  |  |  |  | PN | Y | PY | Y | Y | PY | PY | Y | PY | Y | Y | N | Y | Y | Y | Y | Y |
| Shahbakhti M et al. (2019) | Y | Y | Y | Y | Y | Y | Y | Y | Y | Y | Y | Y | Y | Y | Y | Y | N | N | Y | Y |  |  |  |  |  |  |  |  |  |  |  |  |  |  |  |  |  |
| Shi W et al. (2013) | Y | Y | PY | Y | Y | Y | Y | Y | PY | Y | Y | Y | PY | Y | Y | Y | Y | Y | N | Y |  |  |  |  |  |  |  |  |  |  |  |  |  |  |  |  |  |
| Signorini MG et al. (2020) | N | Y | PY | Y | Y | PN | PN | Y | PY | Y | Y | Y | Y | Y | PY | Y | Y | Y | N | Y |  |  |  |  |  |  |  |  |  |  |  |  |  |  |  |  |  |
| Sims CJ et al. (2000) | Y | Y | PY | PY | Y | PY | PY | Y | PY | PY | Y | PY | PY | Y | PN | Y | Y | Y | N | Y |  |  |  |  |  |  |  |  |  |  |  |  |  |  |  |  |  |
| Sovio U et al. (2018) | Y | PN | PY | Y | Y | PY | PY | Y | PY | Y | Y | PY | PY | Y | Y | Y | PN | Y | Y | Y |  |  |  |  |  |  |  |  |  |  |  |  |  |  |  |  |  |
| Spilka J et al. (2015) | PY | Y | Y | Y | Y | Y | Y | Y | Y | Y | Y | PY | Y | PY | Y | Y | PY | PY | Y | Y |  |  |  |  |  |  |  |  |  |  |  |  |  |  |  |  |  |
| Spilka J et al. (2017) | PY | Y | Y | Y | Y | Y | Y | Y | Y | Y | Y | N | Y | PY | Y | Y | PY | PY | Y | Y |  |  |  |  |  |  |  |  |  |  |  |  |  |  |  |  |  |
| Stamatopoulos N et al. (2015) | Y | PN | Y | PN | Y | PY | PY | N | PY | PN | Y | N | Y | Y | Y | Y | PN | Y | Y | Y | Y | PN | Y | PN | Y | PY | PY | N | PY | PN | Y | NI | NI | NI | NI | PN | Y |
| Stott D et al. (2017) | Y | Y | PY | PN | Y | PY | PY | N | PY | PN | Y | N | Y | Y | Y | Y | Y | Y | Y | N |  |  |  |  |  |  |  |  |  |  |  |  |  |  |  |  |  |
| Stroux L et al. (2017) | Y | NI | Y | Y | Y | Y | Y | Y | Y | Y | Y | Y | Y | Y | Y | Y | NI | Y | Y | Y |  |  |  |  |  |  |  |  |  |  |  |  |  |  |  |  |  |
| Sufriyana H et al. (2020) | Y | Y | PY | Y | Y | Y | Y | Y | PY | Y | Y | Y | Y | Y | Y | Y | Y | Y | Y | Y | Y | Y | PY | Y | Y | Y | Y | Y | PY | Y | Y | Y | Y | Y | Y | Y | Y |
| Tessmer-Tuck JA et al. (2014) | Y | Y | PY | PY | Y | PY | PY | Y | PY | PY | Y | N | Y | Y | N | Y | Y | Y | Y | Y | Y | Y | Y | Y | PY | PY | PY | Y | PY | PY | Y | N | Y | Y | N | Y | Y |
| Theriault S et al. (2016) | Y | Y | PY | Y | Y | Y | Y | Y | Y | Y | Y | Y | PY | Y | PN | Y | Y | Y | Y | Y |  |  |  |  |  |  |  |  |  |  |  |  |  |  |  |  |  |
| Timmerman E et al. (2010) | Y | PY | PY | PN | Y | PY | PY | N | PY | PN | Y | Y | Y | Y | PY | Y | PY | Y | PN | Y |  |  |  |  |  |  |  |  |  |  |  |  |  |  |  |  |  |
| Tran D et al. (2019) | Y | PY | PY | Y | Y | PY | PY | Y | PY | Y | Y | Y | Y | Y | Y | Y | PY | Y | Y | Y |  |  |  |  |  |  |  |  |  |  |  |  |  |  |  |  |  |
| Troisi J et al. (2018) | Y | Y | Y | Y | Y | PY | Y | Y | PY | PY | Y | Y | Y | Y | Y | Y | Y | Y | Y | Y |  |  |  |  |  |  |  |  |  |  |  |  |  |  |  |  |  |
| Tsur A et al. (2019) | N | Y | PY | Y | Y | PY | PY | Y | PY | Y | Y | N | Y | Y | Y | Y | Y | Y | Y | Y | Y | PY | PY | Y | Y | PY | PY | Y | PY | Y | Y | N | Y | Y | Y | PY | Y |
| Uyar A et al. (2010) | PY | NI | PN | NI | Y | NI | NI | NI | NI | NI | NI | Y | Y | NI | NI | Y | NI | PN | Y | NI |  |  |  |  |  |  |  |  |  |  |  |  |  |  |  |  |  |
| Uyar A et al. (2015) | PY | PY | PN | Y | Y | Y | Y | Y | Y | Y | Y | Y | Y | N | N | Y | Y | PN | Y | Y |  |  |  |  |  |  |  |  |  |  |  |  |  |  |  |  |  |
| Valensise H et al. (2006) | Y | Y | Y | Y | Y | PY | PY | Y | PY | Y | Y | N | Y | Y | Y | Y | Y | Y | N | N |  |  |  |  |  |  |  |  |  |  |  |  |  |  |  |  |  |
| van Baaren GJ et al. (2015) | Y | Y | Y | Y | Y | Y | Y | Y | Y | Y | Y | N | Y | Y | Y | Y | Y | Y | Y | Y |  |  |  |  |  |  |  |  |  |  |  |  |  |  |  |  |  |
| van Calster B et al. (2009) | Y | PY | Y | Y | Y | Y | Y | Y | PY | Y | Y | N | Y | Y | Y | Y | PY | Y | Y | N | Y | PY | Y | Y | Y | Y | Y | Y | PY | Y | Y | N | Y | Y | Y | PY | Y |
| van der Ham DP et al. (2014) | Y | Y | Y | Y | Y | Y | Y | Y | Y | Y | Y | N | N | Y | Y | Y | Y | Y | Y | Y |  |  |  |  |  |  |  |  |  |  |  |  |  |  |  |  |  |
| van der Tuuk K et al. (2015) | Y | Y | PY | PN | Y | PY | PY | N | PY | PN | Y | N | Y | Y | Y | Y | Y | Y | Y | N |  |  |  |  |  |  |  |  |  |  |  |  |  |  |  |  |  |
| Verhoeven CJ et al. (2016) | N | Y | Y | Y | Y | Y | Y | Y | Y | Y | Y | Y | PN | Y | PN | Y | Y | Y | Y | Y |  |  |  |  |  |  |  |  |  |  |  |  |  |  |  |  |  |
| VerMilyea M et al. (2020) | Y | Y | Y | Y | Y | Y | Y | Y | Y | Y | Y | PY | Y | Y | Y | Y | Y | Y | PN | Y |  |  |  |  |  |  |  |  |  |  |  |  |  |  |  |  |  |
| Vieira MC et al. (2017) | Y | Y | Y | PN | Y | Y | Y | N | Y | PN | Y | Y | Y | Y | PN | Y | Y | Y | Y | N |  |  |  |  |  |  |  |  |  |  |  |  |  |  |  |  |  |
| Visentin S et al. (2017) | Y | Y | Y | Y | Y | Y | Y | Y | Y | Y | Y | N | Y | Y | NI | Y | Y | Y | Y | N |  |  |  |  |  |  |  |  |  |  |  |  |  |  |  |  |  |
| Vogiatzi P et al. (2019) | Y | Y | PY | Y | Y | PY | PY | Y | PY | Y | Y | N | Y | Y | Y | Y | Y | Y | Y | Y |  |  |  |  |  |  |  |  |  |  |  |  |  |  |  |  |  |
| Wald M et al. (2005) | PY | PN | NI | Y | Y | NI | NI | Y | NI | Y | Y | N | Y | Y | Y | Y | PN | N | Y | Y |  |  |  |  |  |  |  |  |  |  |  |  |  |  |  |  |  |
| Wang C et al. (2016) | Y | Y | Y | Y | Y | Y | Y | Y | Y | Y | Y | Y | Y | Y | Y | Y | Y | Y | Y | Y |  |  |  |  |  |  |  |  |  |  |  |  |  |  |  |  |  |
| Wang L et al. (2016) | Y | Y | Y | Y | Y | Y | Y | Y | Y | Y | Y | N | Y | Y | Y | NI | Y | Y | N | Y |  |  |  |  |  |  |  |  |  |  |  |  |  |  |  |  |  |
| Weber A et al. (2018) | N | Y | PY | Y | Y | PY | PY | Y | PY | Y | Y | Y | Y | Y | Y | Y | Y | Y | Y | Y |  |  |  |  |  |  |  |  |  |  |  |  |  |  |  |  |  |
| Xing YP et al. (2019) | Y | PN | PY | PN | Y | PY | PY | N | PY | PN | Y | N | Y | Y | N | Y | PN | Y | Y | N |  |  |  |  |  |  |  |  |  |  |  |  |  |  |  |  |  |
| Xu H et al. (2019) | Y | Y | Y | Y | Y | Y | Y | Y | Y | Y | Y | Y | Y | Y | Y | Y | Y | Y | Y | Y |  |  |  |  |  |  |  |  |  |  |  |  |  |  |  |  |  |
| Xu H et al. (2020) | Y | Y | Y | Y | Y | Y | Y | Y | Y | Y | Y | Y | Y | Y | Y | Y | Y | Y | Y | PY |  |  |  |  |  |  |  |  |  |  |  |  |  |  |  |  |  |
| Xu L et al. (2013) | Y | Y | PY | Y | Y | Y | Y | Y | Y | Y | Y | Y | Y | PY | PY | Y | Y | Y | Y | PY |  |  |  |  |  |  |  |  |  |  |  |  |  |  |  |  |  |
| Yang H et al. (2015) | Y | PN | Y | Y | Y | Y | Y | Y | Y | Y | Y | Y | Y | Y | NI | Y | PN | Y | Y | N |  |  |  |  |  |  |  |  |  |  |  |  |  |  |  |  |  |
| Yang T et al. (2019) | Y | Y | PY | Y | Y | PY | PY | Y | PY | Y | Y | Y | PN | Y | N | Y | Y | Y | Y | PY |  |  |  |  |  |  |  |  |  |  |  |  |  |  |  |  |  |
| Yu CH et al. (2018) | Y | Y | PY | PN | Y | PY | PY | N | PY | PN | Y | Y | Y | Y | Y | Y | Y | Y | PY | Y |  |  |  |  |  |  |  |  |  |  |  |  |  |  |  |  |  |
| Zhao RF et al. (2019) | Y | Y | Y | Y | Y | Y | Y | Y | Y | Y | Y | Y | Y | Y | Y | Y | Y | N | Y | Y | Y | Y | Y | Y | Y | Y | Y | Y | Y | Y | Y | Y | Y | Y | Y | Y | N |
| Zheng T et al. (2019) | Y | PY | Y | Y | Y | Y | Y | Y | Y | Y | Y | Y | Y | Y | Y | Y | PY | Y | Y | Y |  |  |  |  |  |  |  |  |  |  |  |  |  |  |  |  |  |
| Zwertbroek EF et al. (2017) | Y | Y | PY | PN | Y | PY | PY | N | PY | PN | Y | N | Y | Y | PY | Y | Y | Y | Y | N |  |  |  |  |  |  |  |  |  |  |  |  |  |  |  |  |  |

Y, yes; PY, probably yes; PN, probably no; N, no; NI, no information.

# Table S9. Predictive performance and sample size.

| Study Author (Publication Year) | Type | LR algorithm | | | Non-LR algorithm | | | | Sample size | | AUROC Source |
| --- | --- | --- | --- | --- | --- | --- | --- | --- | --- | --- | --- |
|  |  | AUROC | LB | UB | Algorithm | AUROC | LB | UB | Events | Total |  |
|  |  |  |  |  |  |  |  |  |  |  |  |
| Abbas SA et al. (2018) | Non-LR |  |  |  | Random Forests | 0.941 |  |  | 55 | 122 | Inferred |
| Agopian AJ et al. (2012) | Both | 0.56 | 0.53 | 0.6 | Decision Tree | 0.58 |  |  | 1239 | 9733 | Reported |
| Alberola-Rubio J et al. (2017) | Non-LR |  |  |  | Support Vector Machine | 0.93 |  |  | 24 | 62 | Reported |
| Allouche M et al. (2011) | LR | 0.72 | 0.67 | 0.78 |  |  |  |  | 197 | 906 | Reported |
| Almeida ST et al. (2017) | LR | 0.72 | 0.67 | 0.77 |  |  |  |  | 55 | 325 | Reported |
| Al-Rubaie ZT et al. (2020) | LR | 0.72 | 0.63 | 0.81 |  |  |  |  | 293 | 12395 | Reported |
| Amini P et al. (2017) | Both | 0.69 |  |  | Decision Tree | 0.6 |  |  | 244 | 4415 | Reported |
| Artzi NS et al. (2020) | Both | 0.692 | 0.686 | 0.699 | Gradient Boosting | 0.875 | 0.868 | 0.885 | 1984 | 82678 | Reported |
| Balani J et al. (2018) | Non-LR |  |  |  | Random Forests | 0.649 |  |  | 55 | 227 | Inferred |
| Bastek JA et al. (2012) | LR | 0.73 |  |  |  |  |  |  | 204 | 483 | Reported |
| Benalcazar-Parra C et al. (2019) | Non-LR |  |  |  | Artificial Neural Network | 0.87 |  |  | 33 | 115 | Reported |
| Benhalima K et al. (2020) | LR | 0.716 | 0.678 | 0.754 |  |  |  |  | 231 | 1843 | Reported |
| Berntorp K et al. (2015) | LR | 0.69 | 0.66 | 0.72 |  |  |  |  | 277 | 5487 | Reported |
| Blank C et al. (2019) | Both | 0.66 | 0.61 | 0.71 | Random Forests | 0.74 | 0.71 | 0.77 | 253 | 1052 | Reported |
| Borup R et al. (2016) | Non-LR |  |  |  | Support Vector Machine | 0.7 | 0.59 | 0.81 | 12 | 27 | Reported |
| Broekmans FJ et al. (2014) | LR | 0.82 |  |  |  |  |  |  | 232 | 747 | Reported |
| Carlsson FM et al. (2020) | LR | 0.67 |  |  |  |  |  |  | 13688 | 19343 | Reported |
| Casikar I et al. (2013) | LR | 0.803 |  |  |  |  |  |  | 97 | 126 | Reported |
| Cerqueira FR et al. (2014) | LR | 0.84 |  |  |  |  |  |  | 39 | 293 | Reported |
| Chandrasekaran S et al. (2016) | LR | 0.73 | 0.66 | 0.79 |  |  |  |  | 79 | 669 | Reported |
| Chen L and Hao Y (2017) | Non-LR |  |  |  | Artificial Neural Network | 0.88 |  |  | 50 | 100 | Reported |
| Chen L et al. (2018) | LR | 0.746 | 0.667 | 0.825 |  |  |  |  | 122 | 340 | Reported |
| Chen L et al. (2019) | Non-LR |  |  |  | Deep Neural Network | 0.9 |  |  | 50 | 100 | Reported |
| Ciobanu A et al. (2019) | LR | 0.898 | 0.892 | 0.904 |  |  |  |  | 2024 | 19209 | Reported |
| Comert Z et al. (2018) | Non-LR |  |  |  | Support Vector Machine | 0.65 | 0.64 | 0.66 | 113 | 552 | Reported |
| Coppede F et al. (2010) | Non-LR |  |  |  | Artificial Neural Network | 0.913 | 0.885 | 0.94 | 29 | 61 | Reported |
| Cortet M et al. (2015) | LR | 0.64 | 0.62 | 0.65 |  |  |  |  | 1361 | 6387 | Reported |
| Crovetto F et al. (2015) | LR | 0.978 | 0.966 | 0.99 |  |  |  |  | 57 | 9216 | Reported |
| de Oliveira RV et al. (2012) | LR | 0.905 |  |  |  |  |  |  | 23 | 70 | Reported |
| de Wilde MA et al. (2014) | LR | 0.83 | 0.77 | 0.89 |  |  |  |  | 41 | 189 | Reported |
| Despotovic D et al. (2018) | Non-LR |  |  |  | Random Forests | 0.964 |  |  | 19 | 162 | Reported |
| Eggebo TM et al. (2015) | LR | 0.853 | 0.678 | 1 |  |  |  |  | 20 | 61 | Reported |
| Elaveyni U et al. (2011) | Non-LR |  |  |  | Artificial Neural Network |  |  |  | 6 | 50 |  |
| Fagerberg MC et al. (2015) | LR | 0.74 | 0.73 | 0.74 |  |  |  |  | 18485 | 24736 | Reported |
| Fergus P et al. (2013) | Both | 0.94 |  |  | Decision Tree | 0.93 |  |  | 38 | 300 | Reported |
| Fergus P et al. (2016) | Non-LR |  |  |  | Artificial Neural Network | 0.94 |  |  | 38 | 300 | Reported |
| Fergus P et al. (2017) | Non-LR |  |  |  | Deep Neural Network | 0.8711 |  |  | 46 | 552 | Reported |
| Fergus P et al. (2018) | Non-LR |  |  |  | Deep Neural Network | 0.9825 |  |  | 632 | 1527 | Reported |
| Figueras F et al. (2015) | Non-LR |  |  |  | Decision Tree | 0.828 | 0.751 | 0.886 | 134 | 509 | Reported |
| Fiset S et al. (2019) | Non-LR |  |  |  | Artificial Neural Network | 0.75 | 0.67 | 0.82 | 61 | 98 | Reported |
| Gao C et al. (2019) | Non-LR |  |  |  | Deep Neural Network | 0.827 | 0.813 | 0.84 | 134 | 509 | Reported |
| Garces MF et al. (2015) | Non-LR |  |  |  | Decision Tree | 0.73 |  |  | 16 | 53 | Reported |
| Georgulas G, Karvelis P, Spilka J et al. (2017) | Non-LR |  |  |  | Support Vector Machine | 0.731 |  |  | 44 | 552 | Inferred |
| Guo Z et al. (2020) | LR | 0.804 | 0.71 | 0.899 |  |  |  |  | 23 | 115 | Reported |
| Hamdi MA et al. (2019) | Non-LR |  |  |  | Artificial Neural Network | 0.945 |  |  | 38 | 300 | Inferred |
| Harper LM et al. (2016) | LR | 0.86 |  |  |  |  |  |  | 63 | 220 | Reported |
| Hernandez-Gonzalez J et al. (2018) | Non-LR |  |  |  | Naïve Bayes |  |  |  | 45 | 253 |  |
| Isakov O et al. (2019) | Both | 967 |  |  | Decision Tree | 0.933 | 0.863 | 1 | 162 | 250 | Reported |
| Isono W et al. (2011) | LR | 0.734 | 0.731 | 0.737 |  |  |  |  | 129 | 392 | Reported |
| Jhee JH et al. (2019) | Non-LR |  |  |  | Gradient Boosting | 0.924 |  |  | 474 | 10532 | Reported |
| Kang J et al. (2020) | LR | 0.922 | 0.89 | 0.95 |  |  |  |  | 21 | 287 | Reported |
| Kawakita T et al. (2019) | LR | 0.77 | 0.71 | 0.84 |  |  |  |  | 76 | 6301 | Reported |
| Khan N et al. (2019) | LR | 0.892 | 0.884 | 0.9 |  |  |  |  | 4229 | 45847 | Reported |
| Koivu A et al. (2020) | Both | 0.73 | 0.71 | 0.74 | Gradient Boosting | 0.75 | 0.74 | 0.77 | 768 | 1196568 | Reported |
| Kok M et al. (2011) | LR | 0.71 | 0.66 | 0.77 |  |  |  |  | 122 | 310 | Reported |
| Kuhle S et al. (2018) | Both | 0.771 |  |  | Artificial Neural Network | 0.722 |  |  | 1245 | 30705 | Reported |
| Kumar SN et al. (2020) | Both | 0.85 |  |  | Support Vector Machine | 0.81 |  |  | 55 | 175 | Reported |
| Lafalla O et al. (2019) | LR | 0.833 |  |  |  |  |  |  | 222 | 373 | Reported |
| Lee JSE et al. (2018) | LR | 0.89 | 0.858 | 0.923 |  |  |  |  | 86 | 10170 | Reported |
| Lee KS et al. (2019) | Both | 0.918 |  |  | Artificial Neural Network | 0.9115 |  |  | 43 | 596 | Reported |
| Leonarduzzi R et al. (2015) | Non-LR |  |  |  | Support Vector Machine | 0.755 | 0.746 | 0.764 | 37 | 1288 | Reported |
| Li H et al. (2017) | Non-LR |  |  |  | Artificial Neural Network | 0.87 | 0.75 | 0.98 | 123 | 366 | Reported |
| Liu B et al. (2019) | Both | 0.63 |  |  | Deep Neural Network | 0.67 |  |  |  | 65276 | Reported |
| Macones GA et al. (2001) | Both | 0.77 |  |  | Artificial Neural Network | 0.53 |  |  | 100 | 400 | Reported |
| Mardy AH et al. (2016) | LR | 0.78 | 0.74 | 0.82 |  |  |  |  | 303 | 1295 | Reported |
| Maroufizadeh S et al. (2018) | Both | 0.75 | 0.71 | 0.79 | Artificial Neural Network | 0.8 | 0.76 | 0.84 | 591 | 2120 | Reported |
| Mas-Cabo J et al. (2019) | Non-LR |  |  |  | Artificial Neural Network | 0.911 | 0.862 | 0.96 | 38 | 300 | Reported |
| McCowan LM et al. (2013) | LR | 0.69 |  |  |  |  |  |  | 465 | 633 | Reported |
| McCowan LM et al. (2017) | LR | 0.84 | 0.78 | 0.89 |  |  |  |  | 465 | 633 | Reported |
| Mehta-Lee SS et al. (2017) | LR | 0.647 |  |  |  |  |  |  | 47654 | 192110 | Reported |
| Meijerink AM et al. (2016) | LR | 0.67 | 0.62 | 0.72 |  |  |  |  | 337 | 1559 | Reported |
| Meister MR et al. (2016) | LR | 0.72 |  |  |  |  |  |  | 3382 | 5543 | Reported |
| Mello G et al. (2001) | Non-LR |  |  |  | Artificial Neural Network | 0.952 |  |  | 76 | 303 | Reported |
| Menon R et al. (2014) | LR | 0.89 |  |  |  |  |  |  |  | 40 | Reported |
| Metz TD et al. (2013) | LR | 0.7 | 0.67 | 0.74 |  |  |  |  | 938 | 1170 | Reported |
| Milewski R et al. (2013) | Non-LR |  |  |  | Artificial Neural Network | 0.703 | 0.676 | 0.73 |  |  | Reported |
| Milewski R et al. (2017) | Non-LR |  |  |  | Artificial Neural Network | 0.71 | 0.59 | 0.84 |  |  | Reported |
| Mirroshandel SA et al. (2016) | Non-LR |  |  |  | Random Forests | 0.982 |  |  | 90 | 329 | Reported |
| Morales DA et al. (2008) | Non-LR |  |  |  | Naïve Bayes | 0.594 |  |  | 43 | 61 | Inferred |
| Murtoniemi K et al. (2018) | LR | 0.66 |  |  |  |  |  |  | 34 | 257 | Reported |
| Myers JE et al. (2013) | LR | 0.85 | 0.79 | 0.92 |  |  |  |  | 47 | 235 | Reported |
| Oates J et al. (2013) | LR | 0.78 |  |  |  |  |  |  | 65 | 481 | Reported |
| Paydar K et al. (2017) | Non-LR |  |  |  | Artificial Neural Network | 0.97 |  |  | 46 | 149 | Reported |
| Payne BA et al. (2015) | LR | 0.75 | 0.71 | 0.8 |  |  |  |  | 110 | 1688 | Reported |
| Petrozzielo A et al. (2018) | Non-LR |  |  |  | Deep Neural Network | 0.68 |  |  | 1470 | 35429 | Reported |
| Petrozzielo A et al. (2019) | Non-LR |  |  |  | Deep Neural Network | 0.76 |  |  | 180 | 4429 | Reported |
| Pettersson G et al. (2010) | LR | 0.65 |  |  |  |  |  |  | 1106 | 1462 | Reported |
| Pettersson G et al. (2017) | LR | 0.86 | 0.8 | 0.91 |  |  |  |  | 70 | 271 | Reported |
| Qiu H et al. (2017) | Non-LR |  |  |  | Ensemble | 0.847 |  |  | 613 | 4378 | Reported |
| Qiu K et al. (2019) | Non-LR |  |  |  | Gradient Boosting | 0.74 | 0.72 | 0.76 | 2797 | 7188 | Reported |
| Ramanah R et al. (2018) | LR | 0.75 |  |  |  |  |  |  | 373 | 1913 | Reported |
| Reid S et al. (2015) | LR | 0.96 | 0.89 | 1 |  |  |  |  | 47 | 189 | Reported |
| Rinaudo P et al. (2012) | LR | 0.87 | 0.74 | 1 |  |  |  |  | 54 | 108 | Reported |
| Ryu A et al. (2019) | LR | 0.902 | 0.817 | 0.986 |  |  |  |  | 65 | 140 | Reported |
| Sadi-Ahmed N et al. (2017) | Non-LR |  |  |  | Support Vector Machine | 0.96 |  |  | 15 | 30 | Reported |
| Saleem S et al. (2019) | Non-LR |  |  |  | Gradient Boosting | 0.98 |  |  | 46 | 552 | Reported |
| Sananes N et al. (2013) | LR | 0.693 | 0.655 | 0.731 |  |  |  |  | 1188 | 31834 | Reported |
| Sandstrom A et al. (2019) | LR | 0.67 | 0.66 | 0.68 |  |  |  |  | 2773 | 62562 | Reported |
| Scheinhardt MO et al. (2018) | LR | 0.8992 | 0.824 | 0.975 |  |  |  |  | 30 | 211 | Reported |
| Shahbakhti M et al. (2019) | Non-LR |  |  |  | Support Vector Machine | 0.991 |  |  | 38 | 300 | Inferred |
| Shi W et al. (2013) | LR | 0.6934 | 0.671 | 0.716 |  |  |  |  | 1133 | 2313 | Reported |
| Signorini MG et al. (2020) | Non-LR |  |  |  | Random Forests | 0.974 | 0.946 | 1 | 60 | 120 | Reported |
| Sims CJ et al. (2000) | Both | 0.93 |  |  | Decision Tree | 0.93 |  |  | 3735 | 22157 | Reported |
| Sovio U et al. (2018) | LR | 0.71 |  |  |  |  |  |  | 569 | 22157 | Reported |
| Spilka J et al. (2015) | Non-LR |  |  |  | Support Vector Machine | 0.771 | 0.766 | 0.777 | 37 | 1288 | Reported |
| Spilka J et al. (2017) | Non-LR |  |  |  | Support Vector Machine | 0.77 |  |  | 37 | 1288 | Reported |
| Stamatopoulos N et al. (2015) | LR | 0.816 | 0.733 | 0.898 |  |  |  |  | 66 | 862 | Reported |
| Stott D et al. (2017) | LR | 0.96 | 0.86 | 0.99 |  |  |  |  | 37 | 50 | Reported |
| Stroux L et al. (2017) | LR | 0.76 |  |  |  |  |  |  | 1163 | 2326 | Reported |
| Sufriyana H et al. (2020) | Non-LR |  |  |  | Random Forests | 0.88 | 0.88 | 0.89 | 145 | 1322 | Reported |
| Tessmer-Tuck JA et al. (2014) | LR | 0.723 | 0.68 | 0.767 |  |  |  |  | 143 | 599 | Reported |
| Theriault S et al. (2016) | LR | 0.895 | 0.858 | 0.933 |  |  |  |  | 264 | 792 | Reported |
| Timmerman E et al. (2010) | LR | 0.79 | 0.74 | 0.83 |  |  |  |  | 239 | 445 | Reported |
| Tran D et al. (2019) | Non-LR |  |  |  | Deep Neural Network | 0.93 | 0.92 | 0.94 | 694 | 8836 | Reported |
| Troisi J et al. (2018) | Non-LR |  |  |  | Ensemble | 0.996 | 0.988 | 1 | 70 | 168 | Reported |
| Tsur A et al. (2019) | LR | 0.793 | 0.752 | 0.834 |  |  |  |  | 31 | 2584 | Reported |
| Uyar A et al. (2010) | Non-LR |  |  |  | Naïve Bayes | 0.739 | 0.703 | 0.775 | 2183 | 2453 | Reported |
| Uyar A et al. (2015) | Non-LR |  |  |  | Naïve Bayes | 0.754 |  |  | 270 | 2453 | Reported |
| Valensise H et al. (2006) | Non-LR |  |  |  | Artificial Neural Network | 0.62 | 0.52 | 0.71 | 42 | 84 | Reported |
| van Baaren GJ et al. (2015) | LR | 0.95 | 0.92 | 0.97 |  |  |  |  | 73 | 600 | Reported |
| van Calster B et al. (2009) | LR | 0.989 | 0.978 | 0.995 |  |  |  |  | 396 | 856 | Reported |
| van der Ham DP et al. (2014) | LR | 0.71 | 0.61 | 0.82 |  |  |  |  | 33 | 970 | Reported |
| van der Tuuk K et al. (2015) | LR | 0.8 | 0.75 | 0.85 |  |  |  |  | 126 | 756 | Reported |
| Verhoeven CJ et al. (2016) | LR | 0.83 | 0.8 | 0.87 |  |  |  |  | 309 | 927 | Reported |
| VerMilyea M et al. (2020) | Non-LR |  |  |  | Deep Neural Network | 0.66 |  |  | 97 | 193 | Reported |
| Vieira MC et al. (2017) | LR | 0.68 | 0.65 | 0.71 |  |  |  |  | 505 | 1409 | Reported |
| Visentin S et al. (2017) | LR | 0.8107 | 0.758 | 0.863 |  |  |  |  | 73 | 1381 | Reported |
| Vogiatzi P et al. (2019) | Non-LR |  |  |  | Artificial Neural Network | 0.671 |  |  | 92 | 426 | Reported |
| Wald M et al. (2005) | Both | 0.575 |  |  | Artificial Neural Network | 0.783 |  |  |  | 113 | Reported |
| Wang C et al. (2016) | LR | 0.717 | 0.648 | 0.77 |  |  |  |  | 1062 | 5265 | Reported |
| Wang L et al. (2016) | LR | 0.976 |  |  |  |  |  |  |  | 61 | Reported |
| Weber A et al. (2018) | LR | 0.67 | 0.67 | 0.68 |  |  |  |  | 1130 | 54084 | Reported |
| Xing YP et al. (2019) | LR | 0.849 | 0.78 | 0.89 |  |  |  |  | 52 | 406 | Reported |
| Xu H et al. (2019) | LR | 0.923 |  |  |  |  |  |  | 1389 | 1650 | Reported |
| Xu H et al. (2020) | LR | 0.942 |  |  |  |  |  |  | 78 | 1703 | Reported |
| Xu L et al. (2013) | Non-LR |  |  |  | Random Forests | 0.726 |  |  | 255 | 1214 | Inferred |
| Yang H et al. (2015) | LR | 0.72 | 0.68 | 0.76 |  |  |  |  | 302 | 612 | Reported |
| Yang T et al. (2019) | LR | 0.927 |  |  |  |  |  |  | 290 | 5581 | Reported |
| Yu CH et al. (2018) | LR | 0.79 | 0.766 | 0.813 |  |  |  |  | 521 | 1564 | Reported |
| Zhao RF et al. (2019) | LR | 0.744 |  |  |  |  |  |  | 383 | 6799 | Reported |
| Zheng T et al. (2019) | LR | 0.766 | 0.731 | 0.801 |  |  |  |  | 612 | 4771 | Reported |
| Zwertbroek EF et al. (2017) | LR | 0.76 | 0.731 | 0.807 |  |  |  |  | 115 | 519 | Reported |

LR, logistic regression; AUROC, the area under curve of receiver operating characteristics; LB, lower bound of 95% confidence interval; UB, upper bound of 95% confidence interval.

# Table S10. R code for meta-analysis.

| ---  title: "Comparison of multivariable logistic regression and other machine learning algorithms for prognostic prediction studies in pregnancy care: systematic review and meta-analysis"  output: html_notebook  ---  **# Install and load packages**  ```{r Install packages}  install.packages("tidyverse")  install.packages("metafor")  ```  ```{r Load packages}  library(tidyverse)  library(metafor)  ```  **# Load meta-analysis data**  Please download the dataset here: https://drive.google.com/file/d/1WTLAvp7WTSYZn0rB7ZvQ6w8VfgKbn9uK/view?usp=sharing  ```{r Load meta_analysis.csv}  meta_analysis=read_csv('meta_analysis.csv')  ```  ```{r View meta_analysis dataframe}  meta_analysis  ```  **# Conduct random-effects modeling (REM)**  ```{r Run multiple REM by outcome and algorithm}  meta_analysis_results=  **# Summarize data by outcome and algorithm**  meta_analysis %>%  select(c(1:5,10)) %>%  group_by(Outcome,Algorithm) %>%  summarise(k=n()) %>%  left_join(  group_by(.,Outcome) %>%  summarise(n=sum(k)),  by='Outcome'  ) %>%  arrange(desc(n),desc(k)) %>%  **# For each pair of outcome and algorithm:**    **## Exclude studies as source of heterogeneity**  lapply(  X=seq(nrow(.)),  Y=.,  Z=meta_analysis %>%  filter(  !(Study.y %in% c(  'Theriault S et al. (2016)', # Because of outliers among LR in an outcome  'Bastek JA et al. (2012)', # Because of different outcome definition  'Menon R et al. (2014)', # Because of different outcome definition  'van Baaren GJ et al. (2015)' # Because of different outcome definition  )) &  !(Study.x %in% c(  'Fiset S et al. (2019)' # Because of outliers among the same non-LR in an outcome  ))  ),  FUN=function(X,Y,Z){    **## Conduct REM**  K=Z %>%  filter(Outcome==Y$Outcome[X] & Algorithm==Y$Algorithm[X]) %>%  rma(  data=.,  yi=dlogit_AUROC,  sei=sd(dlogit_AUROC)/sqrt(nrow(.)),  weights=sqrt(`Sample size.y`),  weighted=TRUE  ) %>%  .[c(1,3,6,7,9,13,25,22)] %>%  unlist()    **## Retrieve/calculate information of SE, k, variance, tau-square, and 95% PI**  K=c(K,(K['se']*sqrt(K['k']))^2) %>% setNames(c(names(K),'sd2'))    L=2.2622*sqrt(K['tau2']+K['sd2'])  K=c(K,K['b']-L,K['b']+L) %>% setNames(c(names(K),'pi.lb','pi.ub'))    K=c(Y$Outcome[X],Y$Algorithm[X],K) %>%  matrix(nrow=1,byrow=TRUE,dimnames=list(NULL,c('outcome','algorithm',names(K)))) %>%  data.frame(stringsAsFactors=FALSE) %>%  mutate_at(colnames(.)[c(-1,-2)],as.numeric)  K  }  ) %>%  do.call(rbind,.) %>%  **# Join with the ROB information**  left_join(  meta_analysis %>%  select(Outcome,Algorithm,Study.x,ROB.x) %>%  unique() %>%  group_by(Outcome,Algorithm) %>%  summarise(  Low=sum(ifelse(ROB.x=='+',1,0)),  High=sum(ifelse(ROB.x=='-',1,0)),  Unclear=sum(ifelse(ROB.x=='?',1,0))  ) %>%  ungroup() %>%  mutate(ROB=ifelse(High>0,'High',ifelse(Unclear>0,'Unclear','Low'))) %>%  select(-Low,-High,-Unclear) %>%  setNames(str_to_lower(names(.))),  by=c('outcome','algorithm')  ) %>%  **# Rename the algorithm for simplicity**  mutate(  algorithm=  ifelse(algorithm=='Decision Tree','DT vs.LR',  ifelse(algorithm=='Random Forests','RF vs.LR',  ifelse(algorithm=='Gradient Boosting','GB vs.LR',  ifelse(algorithm=='Naïve Bayes','NB vs.LR',  ifelse(algorithm=='Support Vector Machine','SVM vs.LR',  ifelse(algorithm=='Artificial Neural Network','ANN vs.LR',  ifelse(algorithm=='Deep Neural Network','DNN vs.LR',  ifelse(algorithm=='Ensemble','Ens. vs.LR',NA))))))))  )  ```  ```{r View meta-analysis results}  meta_analysis_results  ``` |
| --- |

# Table S11. Unduplicated and refined records of studies.

| # | Full Citation |
| --- | --- |
|  |  |
|  | Abbas R, Hussain AJ, Al-Jumeily D, Baker T, Khattak A. Classification of foetal distress and hypoxia using machine learning approaches. 2018;10956 LNAI:767-76 |
|  | Abbas SA, Riaz R, Kazmi SZH, Rizvi SS, Kwon SJ. Cause analysis of caesarian sections and application of machine learning methods for classification of birth data. IEEE Access 2018;6:67555-61. doi: https://doi.org/10.1109/ACCESS.2018.2879115. |
|  | Abbas Z, Saad A, Ayache M, Fakih C. Applications of logistic regression and artificial neural network for icsi prediction. International Arab Journal of Information Technology 2019;16:557-64. doi, PMID: https://www.ncbi.nlm.nih.gov/pubmed/WOS:000475506000009. |
|  | Abbasi H, Unsworth CP, McKenzie AC, Gunn AJ, Bennet L. Using type-2 fuzzy logic systems for spike detection in the hypoxic ischemic eeg of the preterm fetal sheep. Conf Proc IEEE Eng Med Biol Soc 2014;2014:938-41. doi: https://doi.org/10.1109/embc.2014.6943746. |
|  | Abdollahi F, Zarghami M, Sazlina SG, Zain AM, Mohammad AJ, Lye MS. Prediction of incidence and bio-psycho-socio-cultural risk factors of post-partum depression immediately after birth in an iranian population. Arch Med Sci 2016;12:1043-51. doi: https://doi.org/10.5114/aoms.2016.58642. |
|  | Abdollahi M, Omani Samani R, Hemat M, et al. Factors that influence the occurrence of multiple pregnancies after intracytoplasmic injection cycles with two or three fresh embryo transfers. Int J Fertil Steril 2017;11:191-96. doi: https://doi.org/10.22074/ijfs.2017.4718. |
|  | Abell SK, Shorakae S, Harrison CL, et al. The association between dysregulated adipocytokines in early pregnancy and development of gestational diabetes. Diabetes Metab Res Rev 2017;33. doi: https://doi.org/10.1002/dmrr.2926. |
|  | Abella R, Satriano A, Frigiola A, et al. Adrenomedullin alterations related to cardiopulmonary bypass in infants with low cardiac output syndrome. J Matern Fetal Neonatal Med 2012;25:2756-61. doi: https://doi.org/10.3109/14767058.2012.718393. |
|  | Abiodun O. Use of emergency contraception in nigeria: An exploration of related factors among sexually active female university students. Sex Reprod Healthc 2016;7:14-20. doi: https://doi.org/10.1016/j.srhc.2015.10.004. |
|  | Abuelghar WM, Ellaithy MI, Swidan KH, Allam IS, Haggag HM. Prediction of spontaneous preterm birth: Salivary progesterone assay and transvaginal cervical length assessment after 24 weeks of gestation, another critical window of opportunity. J Matern Fetal Neonatal Med 2019;32:3847-58. doi: https://doi.org/10.1080/14767058.2018.1482872. |
|  | Abusief ME, Missmer SA, Ginsburg ES, Weeks JC, Partridge AH. Relationship between reproductive history, anthropometrics, lifestyle factors, and the likelihood of persistent chemotherapy-related amenorrhea in women with premenopausal breast cancer. Fertil Steril 2012;97:154-9. doi: https://doi.org/10.1016/j.fertnstert.2011.10.005. |
|  | Acharya UR, Sudarshan VK, Rong SQ, et al. Automated detection of premature delivery using empirical mode and wavelet packet decomposition techniques with uterine electromyogram signals. Comput Biol Med 2017;85:33-42. doi: https://doi.org/10.1016/j.compbiomed.2017.04.013. |
|  | Adam S, Rheeder P. Selective screening strategies for gestational diabetes: A prospective cohort observational study. J Diabetes Res 2017;2017:2849346. doi: https://doi.org/10.1155/2017/2849346. |
|  | Adamson G, Karamlou T, Moore P, Natal-Hernandez L, Tabbutt S, Peyvandi S. Coarctation index predicts recurrent aortic arch obstruction following surgical repair of coarctation of the aorta in infants. Pediatr Cardiol 2017;38:1241-46. doi: https://doi.org/10.1007/s00246-017-1651-4. |
|  | Adedimeji AA, Hoover DR, Shi Q, Cohen MH, Gard T, Anastos K. Differences in the nonuse of any contraception and use of specific contraceptive methods in hiv positive and hiv negative rwandan women. AIDS Res Treat 2012;2012:367604. doi: https://doi.org/10.1155/2012/367604. |
|  | Adeyinka DA, Olakunde BO, Muhajarine N. Evidence of health inequity in child survival: Spatial and bayesian network analyses of stillbirth rates in 194 countries. Sci Rep 2019;9:19755. doi: https://doi.org/10.1038/s41598-019-56326-w. |
|  | Adhikari EH, Yule CS, Roberts SW, et al. Factors associated with postpartum loss to follow-up and detectable viremia after delivery among pregnant women living with hiv. AIDS Patient Care STDS 2019;33:14-20. doi: https://doi.org/10.1089/apc.2018.0117. |
|  | Admon LK, Winkelman TNA, Zivin K, Terplan M, Mhyre JM, Dalton VK. Racial and ethnic disparities in the incidence of severe maternal morbidity in the united states, 2012-2015. Obstet Gynecol 2018;132:1158-66. doi: https://doi.org/10.1097/aog.0000000000002937. |
|  | Adrouche-Amrani L, Green RS, Gluck KM, Lin J. Failure of a repeat course of cyclooxygenase inhibitor to close a pda is a risk factor for developing chronic lung disease in elbw infants. BMC Pediatr 2012;12:10. doi: https://doi.org/10.1186/1471-2431-12-10. |
|  | Agarwal R, Harding V, Short D, et al. Uterine artery pulsatility index: A predictor of methotrexate resistance in gestational trophoblastic neoplasia. Br J Cancer 2012;106:1089-94. doi: https://doi.org/10.1038/bjc.2012.65. |
|  | Aghaeepour N, Lehallier B, Baca Q, et al. A proteomic clock of human pregnancy. Am J Obstet Gynecol 2018;218:347.e1-47.e14. doi: https://doi.org/10.1016/j.ajog.2017.12.208. |
|  | Aghamirzaie D, Batra D, Heath LS, Schneider A, Grene R, Collakova E. Transcriptome-wide functional characterization reveals novel relationships among differentially expressed transcripts in developing soybean embryos. BMC Genomics 2015;16:928. doi: https://doi.org/10.1186/s12864-015-2108-x. |
|  | Agopian AJ, Lupo PJ, Tinker SC, Canfield MA, Mitchell LE, Natl Birth Defects Prevention S. Working towards a risk prediction model for neural tube defects. Birth Defects Research Part a-Clinical and Molecular Teratology 2012;94:141-46. doi: https://doi.org/10.1002/bdra.22883. |
|  | Ahamed MF, Verma P, Lee S, et al. Predictors of successful closure of patent ductus arteriosus with indomethacin. J Perinatol 2015;35:729-34. doi: https://doi.org/10.1038/jp.2015.33. |
|  | Ahlström A, Westin C, Wikland M, Hardarson T. Prediction of live birth in frozen-thawed single blastocyst transfer cycles by pre-freeze and post-thaw morphology. Hum Reprod 2013;28:1199-209. doi: https://doi.org/10.1093/humrep/det054. |
|  | Ahmadzia HK, Phillips JM, James AH, Rice MM, Amdur RL. Predicting peripartum blood transfusion in women undergoing cesarean delivery: A risk prediction model. PLoS One 2018;13:e0208417. doi: https://doi.org/10.1371/journal.pone.0208417. |
|  | Ahmed F, Shams MMB, Shill PC, Rahman M. Classification on bdhs data analysis: Hybrid approach for predicting pregnancy termination. Year. doi: https://doi.org/10.1109/ECACE.2019.8679302. |
|  | Ahn J, Baek SY, Kim K, Cho YS. Predictive factors for hearing outcomes after canaloplasty in patients with congenital aural atresia. Otol Neurotol 2017;38:1140-44. doi: https://doi.org/10.1097/mao.0000000000001504. |
|  | Aizenberg I, Myasnikova E, Samsonova M, Reinitz J. Temporal classification of drosophila segmentation gene expression patterns by the multi-valued neural recognition method. Math Biosci 2002;176:145-59. doi: https://doi.org/10.1016/s0025-5564(01)00104-3. |
|  | Akbarian M, Paydar K, Kalhori SRN, Sheikhtaheri A. Designing an artificial neural network for prediction of pregnancy outcomes in women with systemic lupus erythematosus in iran. Tehran University Medical Journal 2015;73:251-59. doi, PMID. |
|  | Akbulut A, Ertugrul E, Topcu V. Fetal health status prediction based on maternal clinical history using machine learning techniques. Comput Methods Programs Biomed 2018;163:87-100. doi: https://doi.org/10.1016/j.cmpb.2018.06.010. |
|  | Akhavan S, Lak P, Rahimi-Sharbaf F, Mohammadi SR, Shirazi M. Admission test and pregnancy outcome. Iran J Med Sci 2017;42:362-68. doi, PMID: https://www.ncbi.nlm.nih.gov/pubmed/28761202. |
|  | Akhtar F, Li J, Pei Y, et al. Diagnosis and prediction of large-for-gestational-age fetus using the stacked generalization method. Applied Sciences (Switzerland) 2019;9. doi: https://doi.org/10.3390/app9204317. |
|  | Akinmboni TO, Davis NL, Falck AJ, Bearer CF, Mooney SM. Excipient exposure in very low birth weight preterm neonates. J Perinatol 2018;38:169-74. doi: https://doi.org/10.1038/jp.2017.165. |
|  | Akintayo AA, Olagbuji BN, Aderoba AK, Akadiri O, Olofinbiyi BA, Bakare B. Emergency peripartum hysterectomy: A multicenter study of incidence, indications and outcomes in southwestern nigeria. Matern Child Health J 2016;20:1230-6. doi: https://doi.org/10.1007/s10995-016-1924-1. |
|  | Akolekar R, Ciobanu A, Zingler E, Syngelaki A, Nicolaides KH. Routine assessment of cerebroplacental ratio at 35-37 weeks' gestation in the prediction of adverse perinatal outcome. Am J Obstet Gynecol 2019;221:65.e1-65.e18. doi: https://doi.org/10.1016/j.ajog.2019.03.002. |
|  | Akolekar R, Machuca M, Mendes M, Paschos V, Nicolaides KH. Prediction of stillbirth from placental growth factor at 11-13 weeks. Ultrasound Obstet Gynecol 2016;48:618-23. doi: https://doi.org/10.1002/uog.17288. |
|  | Akolekar R, Syngelaki A, Gallo DM, Poon LC, Nicolaides KH. Umbilical and fetal middle cerebral artery doppler at 35-37 weeks' gestation in the prediction of adverse perinatal outcome. Ultrasound Obstet Gynecol 2015;46:82-92. doi: https://doi.org/10.1002/uog.14842. |
|  | Akolekar R, Tokunaka M, Ortega N, Syngelaki A, Nicolaides KH. Prediction of stillbirth from maternal factors, fetal biometry and uterine artery doppler at 19-24 weeks. Ultrasound Obstet Gynecol 2016;48:624-30. doi: https://doi.org/10.1002/uog.17295. |
|  | Alam TM, Khan MMA, Iqbal MA, Wahab A, Mushtaq M. Cervical cancer prediction through different screening methods using data mining. International Journal of Advanced Computer Science and Applications 2019;10:388-96. doi, PMID. |
|  | Alansary A, Oktay O, Li Y, et al. Evaluating reinforcement learning agents for anatomical landmark detection. Med Image Anal 2019;53:156-64. doi: https://doi.org/10.1016/j.media.2019.02.007. |
|  | Alavifard S, Meier K, Shulman Y, Tomlinson G, D'Souza R. Derivation and validation of a model predicting the likelihood of vaginal birth following labour induction. BMC Pregnancy Childbirth 2019;19:130. doi: https://doi.org/10.1186/s12884-019-2232-8. |
|  | Alberola-Rubio J, Garcia-Casado J, Prats-Boluda G, et al. Prediction of labor onset type: Spontaneous vs induced; role of electrohysterography? Comput Methods Programs Biomed 2017;144:127-33. doi: https://doi.org/10.1016/j.cmpb.2017.03.018. |
|  | Alemu T, Umeta M. Prevalence and predictors of "small size" babies in ethiopia: In-depth analysis of the ethiopian demographic and health survey, 2011. Ethiop J Health Sci 2016;26:243-50. doi: https://doi.org/10.4314/ejhs.v26i3.7. |
|  | Alexander PMA, DiOrio M, Andren K, et al. Accurate prediction of congenital heart surgical length of stay incorporating a procedure-based categorical variable. Pediatr Crit Care Med 2018;19:949-56. doi: https://doi.org/10.1097/pcc.0000000000001668. |
|  | Alharbi A, Alghahtani M. Using genetic algorithm and elm neural networks for feature extraction and classification of type 2-diabetes mellitus. Applied Artificial Intelligence 2019;33:311-28. doi: https://doi.org/10.1080/08839514.2018.1560545. |
|  | Ali U, Goldenberg N, Foreman C, et al. Association between cyanosis, transfusion, and thrombotic complications in neonates and children undergoing cardiac surgery. J Cardiothorac Vasc Anesth 2020;34:349-55. doi: https://doi.org/10.1053/j.jvca.2019.07.123. |
|  | Aljohani N, Al Serehi A, Ahmed AM, et al. Factors associated with diabetes mellitus prediction among pregnant arab subjects with gestational diabetes. Int J Clin Exp Pathol 2015;8:8512-5. doi, PMID: https://www.ncbi.nlm.nih.gov/pubmed/26339426. |
|  | Alkharfy TM, Ba-Abbad R, Hadi A, Sobaih BH, AlFaleh KM. Total parenteral nutrition-associated cholestasis and risk factors in preterm infants. Saudi J Gastroenterol 2014;20:293-6. doi: https://doi.org/10.4103/1319-3767.141688. |
|  | Allen KL, Byrne SM, Kusel MM, Hart PH, Whitehouse AJ. Maternal vitamin d levels during pregnancy and offspring eating disorder risk in adolescence. Int J Eat Disord 2013;46:669-76. doi: https://doi.org/10.1002/eat.22147. |
|  | Allen KL, Byrne SM, Oddy WH, Schmidt U, Crosby RD. Risk factors for binge eating and purging eating disorders: Differences based on age of onset. Int J Eat Disord 2014;47:802-12. doi: https://doi.org/10.1002/eat.22299. |
|  | Allen R, Aquilina J. Prospective observational study to determine the accuracy of first-trimester serum biomarkers and uterine artery dopplers in combination with maternal characteristics and arteriography for the prediction of women at risk of preeclampsia and other adverse pregnancy outcomes. J Matern Fetal Neonatal Med 2018;31:2789-806. doi: https://doi.org/10.1080/14767058.2017.1355903. |
|  | Allouche M, Huissoud C, Guyard-Boileau B, Rouzier R, Parant O. Development and validation of nomograms for predicting preterm delivery. Am J Obstet Gynecol 2011;204:242.e1-8. doi: https://doi.org/10.1016/j.ajog.2010.09.030. |
|  | Almeida ST, Katz L, Coutinho I, Amorim MMR. Validation of fullpiers model for prediction of adverse outcomes among women with severe pre-eclampsia. Int J Gynaecol Obstet 2017;138:142-47. doi: https://doi.org/10.1002/ijgo.12197. |
|  | Alonso-Betanzos A, Mosqueira-Rey E, Moret-Bonillo V, Baldonedo del Río B. Applying statistical, uncertainty-based and connectionist approaches to the prediction of fetal outcome: A comparative study. Artif Intell Med 1999;17:37-57. doi: https://doi.org/10.1016/s0933-3657(99)00013-5. |
|  | Al-Rubaie ZTA, Hudson HM, Jenkins G, et al. Prediction of pre-eclampsia in nulliparous women using routinely collected maternal characteristics: A model development and validation study. BMC Pregnancy Childbirth 2020;20:23. doi: https://doi.org/10.1186/s12884-019-2712-x. |
|  | Alsaied T, Sleeper LA, Masci M, et al. Maldistribution of pulmonary blood flow in patients after the fontan operation is associated with worse exercise capacity. J Cardiovasc Magn Reson 2018;20:85. doi: https://doi.org/10.1186/s12968-018-0505-4. |
|  | Alsayegh E, Barrett J, Melamed N. Optimal timing of antenatal corticosteroids in women with bleeding placenta previa or low-lying placenta(). J Matern Fetal Neonatal Med 2019;32:1971-77. doi: https://doi.org/10.1080/14767058.2017.1422713. |
|  | Alsayyari A. Fetal cardiotocography monitoring using legendre neural networks. Biomed Tech (Berl) 2019;64:669-75. doi: https://doi.org/10.1515/bmt-2018-0074. |
|  | Altin C, Yilmaz M, Ozsoy HM, et al. Assessment of epicardial fat and carotid intima media thickness in gestational hypertension. J Obstet Gynaecol Res 2018;44:1072-79. doi: https://doi.org/10.1111/jog.13631. |
|  | Altmäe S, Koel M, Võsa U, et al. Meta-signature of human endometrial receptivity: A meta-analysis and validation study of transcriptomic biomarkers. Sci Rep 2017;7:10077. doi: https://doi.org/10.1038/s41598-017-10098-3. |
|  | Altraigey A, Ellaithy M, Barakat E, Majeed A. Cervical length should be measured for women with placenta previa: Cohort study. J Matern Fetal Neonatal Med 2019:1-281. doi: https://doi.org/10.1080/14767058.2019.1659239. |
|  | Alushi B, Biasco L, Orzan F, et al. Patent foramen ovale treatment strategy: An italian large prospective study. J Cardiovasc Med (Hagerstown) 2014;15:761-8. doi: https://doi.org/10.2459/jcm.0000000000000138. |
|  | Alves JAG, de Sousa PCP, Moura S, Kane SC, Costa FD. First-trimester maternal ophthalmic artery doppler analysis for prediction of pre-eclampsia. Ultrasound in Obstetrics & Gynecology 2014;44:411-18. doi: https://doi.org/10.1002/uog.13338. |
|  | Aly SA, Zurakowski D, Glass P, Skurow-Todd K, Jonas RA, Donofrio MT. Cerebral tissue oxygenation index and lactate at 24 hours postoperative predict survival and neurodevelopmental outcome after neonatal cardiac surgery. Congenit Heart Dis 2017;12:188-95. doi: https://doi.org/10.1111/chd.12426. |
|  | Alzola I, Murua E, Rodríguez J, Burgos J, Maiz N. Can the progression angle before labor help to predict cesarean section? Fetal Diagn Ther 2020;47:284-91. doi: https://doi.org/10.1159/000503387. |
|  | Amabebe E, Reynolds S, He X, Wood R, Stern V, Anumba DOC. Infection/inflammation-associated preterm delivery within 14 days of presentation with symptoms of preterm labour: A multivariate predictive model. PLoS One 2019;14:e0222455. doi: https://doi.org/10.1371/journal.pone.0222455. |
|  | Amar E, Michon L, Gnansia E. Hypospadias and environment: A registry-based case-control study. Revue d'Épidémiologie et de Santé Publique 2018;66:S333-S34. doi: https://doi.org/https://doi.org/10.1016/j.respe.2018.05.257. |
|  | Ambalavanan N, Carlo WA. Comparison of the prediction of extremely low birth weight neonatal mortality by regression analysis and by neural networks. Early Hum Dev 2001;65:123-37. doi: https://doi.org/10.1016/s0378-3782(01)00228-6. |
|  | Ambalavanan N, Carlo WA, Bobashev G, et al. Prediction of death for extremely low birth weight neonates. Pediatrics 2005;116:1367-73. doi: https://doi.org/10.1542/peds.2004-2099. |
|  | Ambalavanan N, Carlo WA, Tyson JE, et al. Outcome trajectories in extremely preterm infants. Pediatrics 2012;130:e115-25. doi: https://doi.org/10.1542/peds.2011-3693. |
|  | Ambalavanan N, Nelson KG, Alexander G, Johnson SE, Biasini F, Carlo WA. Prediction of neurologic morbidity in extremely low birth weight infants. J Perinatol 2000;20:496-503. doi: https://doi.org/10.1038/sj.jp.7200419. |
|  | Ambalavanan N, Walsh M, Bobashev G, et al. Intercenter differences in bronchopulmonary dysplasia or death among very low birth weight infants. Pediatrics 2011;127:e106-16. doi: https://doi.org/10.1542/peds.2010-0648. |
|  | Ameyaw EK, Dickson KS. Skilled birth attendance in sierra leone, niger, and mali: Analysis of demographic and health surveys. BMC Public Health 2020;20:164. doi: https://doi.org/10.1186/s12889-020-8258-z. |
|  | Amialchuk A, Gerhardinger L. Contraceptive use and pregnancies in adolescents' romantic relationships: Role of relationship activities and parental attitudes and communication. J Dev Behav Pediatr 2015;36:86-97. doi: https://doi.org/10.1097/dbp.0000000000000125. |
|  | Amini P, Maroufizadeh S, Samani RO, Hamidi O, Sepidarkish M. Prevalence and determinants of preterm birth in tehran, iran: A comparison between logistic regression and decision tree methods. Osong Public Health Res Perspect 2017;8:195-200. doi: https://doi.org/10.24171/j.phrp.2017.8.3.06. |
|  | Anand V, Schneeberger D, Piedimonte G. A probabilistic model for prediction of bronchopulmonary dysplasia in pre-term and term babies admitted to the nicu. American Journal of Respiratory and Critical Care Medicine 2015;191. doi, PMID. |
|  | Andersen LG, Holst C, Michaelsen KF, Baker JL, Sørensen TI. Weight and weight gain during early infancy predict childhood obesity: A case-cohort study. Int J Obes (Lond) 2012;36:1306-11. doi: https://doi.org/10.1038/ijo.2012.134. |
|  | Anderson CK, Medlin E, Ferriss AF, et al. Association between gelatin-thrombin matrix use and abscesses in women undergoing pelvic surgery. Obstet Gynecol 2014;124:589-95. doi: https://doi.org/10.1097/aog.0000000000000406. |
|  | Anilkumar A, Kappanayil M, Thampi MV, Nampoothiri S, Sundaram KR, Vasudevan DM. Variation in prevalence of chromosome 22q11 deletion in subtypes of conotruncal defect in 254 children. Acta Paediatr 2011;100:e97-100. doi: https://doi.org/10.1111/j.1651-2227.2011.02271.x. |
|  | Annessi E, Del Giovane C, Magnani L, et al. A modified prediction model for vbac, in a european population. J Matern Fetal Neonatal Med 2016;29:435-9. doi: https://doi.org/10.3109/14767058.2014.1002767. |
|  | Ansari MS, Gulia A, Srivastava A, Kapoor R. Risk factors for progression to end-stage renal disease in children with posterior urethral valves. J Pediatr Urol 2010;6:261-4. doi: https://doi.org/10.1016/j.jpurol.2009.09.001. |
|  | Anuwutnavin S, Satou G, Chang RK, DeVore GR, Abuel A, Sklansky M. Prenatal sonographic predictors of neonatal coarctation of the aorta. J Ultrasound Med 2016;35:2353-64. doi: https://doi.org/10.7863/ultra.15.06049. |
|  | Aolin Wang JMS, Roy Gerona , Thomas Lin , Rachel Morello-Frosch , Marina Sirota , . The pregnancy chemisome in relation to birth outcomes and consumer product use: Suspect screening of industrial chemicals. ISES-ISEE 2018 Joint Annual Meeting Year. doi, PMID. |
|  | Araki S, Shima M, Yamamoto K. Spatiotemporal land use random forest model for estimating metropolitan no2 exposure in japan. Science of the Total Environment 2018;634:1269-77. doi: https://doi.org/10.1016/j.scitotenv.2018.03.324. |
|  | Arav-Boger R, Boger YS, Foster CB, Boger Z. The use of artificial neural networks in prediction of congenital cmv outcome from sequence data. Bioinform Biol Insights 2008;2:281-9. doi: https://doi.org/10.4137/bbi.s764. |
|  | Arbel H, Basu S, Fisher WW, et al. Exploiting regulatory heterogeneity to systematically identify enhancers with high accuracy. Proc Natl Acad Sci U S A 2019;116:900-08. doi: https://doi.org/10.1073/pnas.1808833115. |
|  | Arce DY, Bellavia A, Cantonwine DE, et al. Average and time-specific maternal prenatal inflammatory biomarkers and the risk of labor epidural associated fever. PLoS One 2019;14:e0222958. doi: https://doi.org/10.1371/journal.pone.0222958. |
|  | Arena VC, Sussman NB, Mazumdar S, Yu S, Macina OT. The utility of structure-activity relationship (sar) models for prediction and covariate selection in developmental toxicity: Comparative analysis of logistic regression and decision tree models. SAR QSAR Environ Res 2004;15:1-18. doi: https://doi.org/10.1080/1062936032000169633. |
|  | Arfi A, Bendifallah S, Mathieu D'argent E, et al. Nomogram predicting the likelihood of live-birth rate after surgery for deep infiltrating endometriosis without bowel involvement in women who wish to conceive: A retrospective study. Eur J Obstet Gynecol Reprod Biol 2019;235:81-87. doi: https://doi.org/10.1016/j.ejogrb.2019.02.007. |
|  | Arlen AM, Kirsch AJ, Leong T, Broecker BH, Smith EA, Elmore JM. Further analysis of the glans-urethral meatus-shaft (gms) hypospadias score: Correlation with postoperative complications. J Pediatr Urol 2015;11:71.e1-5. doi: https://doi.org/10.1016/j.jpurol.2014.11.015. |
|  | Armstrong R, Scott JG, Whitehouse AJO, Copland DA, McMahon KL, Arnott W. Late talkers and later language outcomes: Predicting the different language trajectories. Int J Speech Lang Pathol 2017;19:237-50. doi: https://doi.org/10.1080/17549507.2017.1296191. |
|  | Armstrong R, Symons M, Scott JG, et al. Predicting language difficulties in middle childhood from early developmental milestones: A comparison of traditional regression and machine learning techniques. Journal of Speech, Language, and Hearing Research 2018;61:1926-44. doi: https://doi.org/10.1044/2018_JSLHR-L-17-0210. |
|  | Arnaoutakis GJ, Zhao Y, George TJ, Sciortino CM, McCarthy PM, Conte JV. Surgical repair of ventricular septal defect after myocardial infarction: Outcomes from the society of thoracic surgeons national database. Ann Thorac Surg 2012;94:436-43; discussion 43-4. doi: https://doi.org/10.1016/j.athoracsur.2012.04.020. |
|  | Arnold SE, Xie SX, Leung YY, et al. Plasma biomarkers of depressive symptoms in older adults. Translational psychiatry 2012;2:e65. doi, PMID. |
|  | Artzi NS, Shilo S, Hadar E, et al. Prediction of gestational diabetes based on nationwide electronic health records. Nat Med 2020;26:71-76. doi: https://doi.org/10.1038/s41591-019-0724-8. |
|  | Asadi H, Kok HK, Looby S, Brennan P, O'Hare A, Thornton J. Outcomes and complications after endovascular treatment of brain arteriovenous malformations: A prognostication attempt using artificial intelligence. World Neurosurg 2016;96:562-69.e1. doi: https://doi.org/10.1016/j.wneu.2016.09.086. |
|  | Asano E, Ebara T, Yamada-Namikawa C, et al. Genotyping analysis for the 46 c/t polymorphism of coagulation factor xii and the involvement of factor xii activity in patients with recurrent pregnancy loss. PLoS One 2014;9:e114452. doi: https://doi.org/10.1371/journal.pone.0114452. |
|  | Ashrafi M, Arabipoor A, Hemat M, Salman-Yazdi R. The impact of the localisation of endometriosis lesions on ovarian reserve and assisted reproduction techniques outcomes. J Obstet Gynaecol 2019;39:91-97. doi: https://doi.org/10.1080/01443615.2018.1465898. |
|  | Ashtarinezhad A, Panahyab A, Shaterzadeh-Oskouei S, Khoshniat H, Mohamadzadehasl B, Shirazi FH. Teratogenic study of phenobarbital and levamisole on mouse fetus liver tissue using biospectroscopy. J Pharm Biomed Anal 2016;128:174-83. doi: https://doi.org/10.1016/j.jpba.2016.05.015. |
|  | Assawapalanggool S, Kasatpibal N, Sirichotiyakul S, Arora R, Suntornlimsiri W. A prognostic scoring tool for cesarean organ/space surgical site infections: Derivation and internal validation. Surg Infect (Larchmt) 2017;18:694-701. doi: https://doi.org/10.1089/sur.2016.264. |
|  | Assibey-Mensah V, Glantz JC, Hopke PK, et al. Ambient wintertime particulate air pollution and hypertensive disorders of pregnancy in monroe county, new york. Environ Res 2019;168:25-31. doi: https://doi.org/10.1016/j.envres.2018.09.003. |
|  | Assibey-Mensah V, Glantz JC, Hopke PK, et al. Wintertime wood smoke, traffic particle pollution, and preeclampsia. Hypertension 2020;75:851-58. doi: https://doi.org/10.1161/hypertensionaha.119.13139. |
|  | Atasever M, Kalem MN, Hatırnaz Ş, Hatırnaz E, Kalem Z, Kalaylıoğlu Z. Factors affecting clinical pregnancy rates after iui for the treatment of unexplained infertility and mild male subfertility. J Turk Ger Gynecol Assoc 2016;17:134-8. doi: https://doi.org/10.5152/jtgga.2016.16056. |
|  | Attallah O, Sharkas MA, Gadelkarim H. Fetal brain abnormality classification from mri images of different gestational age. Brain Sci 2019;9. doi: https://doi.org/10.3390/brainsci9090231. |
|  | Attallah O, Sharkas MA, Gadelkarim H. Deep learning techniques for automatic detection of embryonic neurodevelopmental disorders. Diagnostics 2020;10:23. doi: https://doi.org/10.3390/diagnostics10010027. |
|  | Attar MA, Dechert RE, Schumacher RE, Sarkar S. Do prenatal steroids improve the survival of late preterm infants with complex congenital heart defects? J Neonatal Perinatal Med 2014;7:107-11. doi: https://doi.org/10.3233/npm-1474813. |
|  | Atzmon Y, Shoshan-Karchovsky E, Michaeli M, et al. Obesity results with smaller oocyte in in vitro fertilization/intracytoplasmic sperm injection cycles-a prospective study. J Assist Reprod Genet 2017;34:1145-51. doi: https://doi.org/10.1007/s10815-017-0975-6. |
|  | Aulinas A, Biagetti B, Vinagre I, et al. [gestational diabetes mellitus and maternal ethnicity: High prevalence of fetal macrosomia in non-caucasian women]. Med Clin (Barc) 2013;141:240-5. doi: https://doi.org/10.1016/j.medcli.2012.05.034. |
|  | Aung MT, Yu Y, Ferguson KK, et al. Prediction and associations of preterm birth and its subtypes with eicosanoid enzymatic pathways and inflammatory markers. Sci Rep 2019;9:17049. doi: https://doi.org/10.1038/s41598-019-53448-z. |
|  | Aupont JE, Akolekar R, Illian A, Neonakis S, Nicolaides KH. Prediction of stillbirth from placental growth factor at 19-24 weeks. Ultrasound Obstet Gynecol 2016;48:631-35. doi: https://doi.org/10.1002/uog.17229. |
|  | Austdal M, Tangerås LH, Skråstad RB, et al. First trimester urine and serum metabolomics for prediction of preeclampsia and gestational hypertension: A prospective screening study. Int J Mol Sci 2015;16:21520-38. doi: https://doi.org/10.3390/ijms160921520. |
|  | Avadhani SA, Martin-Doyle W, Shaikh AY, Pape LA. Predictors of ascending aortic dilation in bicuspid aortic valve disease: A five-year prospective study. Am J Med 2015;128:647-52. doi: https://doi.org/10.1016/j.amjmed.2014.12.027. |
|  | Avni FE, Garel C, Cassart M, D'Haene N, Hall M, Riccabona M. Imaging and classification of congenital cystic renal diseases. AJR Am J Roentgenol 2012;198:1004-13. doi: https://doi.org/10.2214/ajr.11.8083. |
|  | Ayachi A, Bouchahda R, Derouich S, et al. Accuracy of preoperative real-time dynamic transvaginal ultrasound sliding sign in prediction of pelvic adhesions in women with previous abdominopelvic surgery: Prospective, multicenter, double-blind study. Ultrasound in Obstetrics & Gynecology 2018;51:253-58. doi: https://doi.org/10.1002/uog.17465. |
|  | Aydin Y, Hassa H, Oge T, Tokgoz VY. Factors predictive of clinical pregnancy in the first intrauterine insemination cycle of 306 couples with favourable female patient characteristics. Hum Fertil (Camb) 2013;16:286-90. doi: https://doi.org/10.3109/14647273.2013.841328. |
|  | Azantee YW, Murad ZA, Roszaman R, Hayati MY, Norsina MA. Associated factors affecting the successful pregnancy rate of intrauterine insemination at international islamic university malaysia (iium) fertility centre. Med J Malaysia 2011;66:195-8. doi, PMID: https://www.ncbi.nlm.nih.gov/pubmed/22111439. |
|  | Baba Y, Ohkuchi A, Usui R, Suzuki H, Kuwata T, Matsubara S. Calculating probability of requiring allogeneic blood transfusion using three preoperative risk factors on cesarean section for placenta previa. Arch Gynecol Obstet 2015;291:281-5. doi: https://doi.org/10.1007/s00404-014-3451-x. |
|  | Baba Y, Takahashi H, Ohkuchi A, Usui R, Matsubara S. Which type of placenta previa requires blood transfusion more frequently? A new concept of indiscernible edge total previa. J Obstet Gynaecol Res 2016;42:1502-08. doi: https://doi.org/10.1111/jog.13097. |
|  | Babtain FA. Impact of a family history of epilepsy on the diagnosis of epilepsy in southern saudi arabia. Seizure 2013;22:542-7. doi: https://doi.org/10.1016/j.seizure.2013.04.002. |
|  | Babu-Narayan SV, Diller GP, Gheta RR, et al. Clinical outcomes of surgical pulmonary valve replacement after repair of tetralogy of fallot and potential prognostic value of preoperative cardiopulmonary exercise testing. Circulation 2014;129:18-27. doi: https://doi.org/10.1161/circulationaha.113.001485. |
|  | Badhiwala JH, Farrokhyar F, Alhazzani W, et al. Surgical outcomes and natural history of intramedullary spinal cord cavernous malformations: A single-center series and meta-analysis of individual patient data: Clinic article. J Neurosurg Spine 2014;21:662-76. doi: https://doi.org/10.3171/2014.6.Spine13949. |
|  | Baer RJ, McLemore MR, Adler N, et al. Pre-pregnancy or first-trimester risk scoring to identify women at high risk of preterm birth. Eur J Obstet Gynecol Reprod Biol 2018;231:235-40. doi: https://doi.org/10.1016/j.ejogrb.2018.11.004. |
|  | Baggen VJM, Venema E, Živná R, et al. Development and validation of a risk prediction model in patients with adult congenital heart disease. Int J Cardiol 2019;276:87-92. doi: https://doi.org/10.1016/j.ijcard.2018.08.059. |
|  | Bahado-Singh RO, Sonek J, McKenna D, et al. Artificial intelligence and amniotic fluid multiomics analysis: The prediction of perinatal outcome in asymptomatic short cervix. Ultrasound Obstet Gynecol 2018. doi: https://doi.org/10.1002/uog.20168. |
|  | Bahado-Singh RO, Vishweswaraiah S, Aydas B, et al. Precision cardiovascular medicine: Artificial intelligence and epigenetics for the pathogenesis and prediction of coarctation in neonates. J Matern Fetal Neonatal Med 2020:1-8. doi: https://doi.org/10.1080/14767058.2020.1722995. |
|  | Bahado-Singh RO, Yilmaz A, Bisgin H, et al. Artificial intelligence and the analysis of multi-platform metabolomics data for the detection of intrauterine growth restriction. PLoS One 2019;14:e0214121. doi: https://doi.org/10.1371/journal.pone.0214121. |
|  | Bakalis S, Akolekar R, Gallo DM, Poon LC, Nicolaides KH. Umbilical and fetal middle cerebral artery doppler at 30-34 weeks' gestation in the prediction of adverse perinatal outcome. Ultrasound Obstet Gynecol 2015;45:409-20. doi: https://doi.org/10.1002/uog.14822. |
|  | Bakalis S, Gallo DM, Mendez O, Poon LC, Nicolaides KH. Prediction of small-for-gestational-age neonates: Screening by maternal biochemical markers at 30-34 weeks. Ultrasound Obstet Gynecol 2015;46:208-15. doi: https://doi.org/10.1002/uog.14861. |
|  | Bakalis S, Peeva G, Gonzalez R, Poon LC, Nicolaides KH. Prediction of small-for-gestational-age neonates: Screening by biophysical and biochemical markers at 30-34 weeks. Ultrasound Obstet Gynecol 2015;46:446-51. doi: https://doi.org/10.1002/uog.14863. |
|  | Bakalis S, Silva M, Akolekar R, Poon LC, Nicolaides KH. Prediction of small-for-gestational-age neonates: Screening by fetal biometry at 30-34 weeks. Ultrasound Obstet Gynecol 2015;45:551-8. doi: https://doi.org/10.1002/uog.14771. |
|  | Bakalis S, Stoilov B, Akolekar R, Poon LC, Nicolaides KH. Prediction of small-for-gestational-age neonates: Screening by uterine artery doppler and mean arterial pressure at 30-34 weeks. Ultrasound Obstet Gynecol 2015;45:707-14. doi: https://doi.org/10.1002/uog.14777. |
|  | Bakkes T, Sammali F, Kuijsters NPM, et al. Machine learning for classification of uterine activity outside pregnancy. Conf Proc IEEE Eng Med Biol Soc 2019;2019:2161-64. doi: https://doi.org/10.1109/embc.2019.8857374. |
|  | Balani J, Hyer SL, Shehata H, Mohareb F. Visceral fat mass as a novel risk factor for predicting gestational diabetes in obese pregnant women. Obstet Med 2018;11:121-25. doi: https://doi.org/10.1177/1753495x17754149. |
|  | Ball G, Aljabar P, Arichi T, et al. Machine-learning to characterise neonatal functional connectivity in the preterm brain. Neuroimage 2016;124:267-75. doi: https://doi.org/10.1016/j.neuroimage.2015.08.055. |
|  | Ballantyne M, Stevens B, Guttmann A, Willan AR, Rosenbaum P. Maternal and infant predictors of attendance at neonatal follow-up programmes. Child Care Health Dev 2014;40:250-8. doi: https://doi.org/10.1111/cch.12015. |
|  | Ballard AR, Mallett LH, Pruszynski JE, Cantey JB. Chorioamnionitis and subsequent bronchopulmonary dysplasia in very-low-birth weight infants: A 25-year cohort. J Perinatol 2016;36:1045-48. doi: https://doi.org/10.1038/jp.2016.138. |
|  | Ballot DE, Potterton J, Chirwa T, Hilburn N, Cooper PA. Developmental outcome of very low birth weight infants in a developing country. BMC Pediatr 2012;12:11. doi: https://doi.org/10.1186/1471-2431-12-11. |
|  | Bao J, Liu J, Qu Y, Mu DL. [predictive value of umbilical arterial cord ph on complications during hospitalization in neonates after cesarean section]. Beijing Da Xue Xue Bao Yi Xue Ban 2019;51:159-64. doi: https://doi.org/10.19723/j.issn.1671-167X.2019.01.027. |
|  | Barberet J, Bruno C, Valot E, et al. Can novel early non-invasive biomarkers of embryo quality be identified with time-lapse imaging to predict live birth? Hum Reprod 2019;34:1439-49. doi: https://doi.org/10.1093/humrep/dez085. |
|  | Bardin R, Aviram A, Meizner I, et al. Association of fetal biparietal diameter with mode of delivery and perinatal outcome. Ultrasound Obstet Gynecol 2016;47:217-23. doi: https://doi.org/10.1002/uog.14837. |
|  | Barnhart KT, Casanova B, Sammel MD, Timbers K, Chung K, Kulp JL. Prediction of location of a symptomatic early gestation based solely on clinical presentation. Obstetrics and Gynecology 2008;112:1319-26. doi: https://doi.org/10.1097/AOG.0b013e31818eddcf. |
|  | Barrett ES, Sathyanarayana S, Janssen S, et al. Environmental health attitudes and behaviors: Findings from a large pregnancy cohort study. Eur J Obstet Gynecol Reprod Biol 2014;176:119-25. doi: https://doi.org/10.1016/j.ejogrb.2014.02.029. |
|  | Barros EP, Schiffer JM, Vorobieva A, Dou J, Baker D, Amaro RE. Improving the efficiency of ligand-binding protein design with molecular dynamics simulations. J Chem Theory Comput 2019;15:5703-15. doi: https://doi.org/10.1021/acs.jctc.9b00483. |
|  | Bastek JA, Sammel MD, Srinivas SK, et al. Clinical prediction rules for preterm birth in patients presenting with preterm labor. Obstet Gynecol 2012;119:1119-28. doi: https://doi.org/10.1097/AOG.0b013e31825503e5. |
|  | Bateman BT, Franklin JM, Bykov K, et al. Persistent opioid use following cesarean delivery: Patterns and predictors among opioid-naïve women. Am J Obstet Gynecol 2016;215:353.e1-53.e18. doi: https://doi.org/10.1016/j.ajog.2016.03.016. |
|  | Bathen TF, Lohaugen GCC, Brubakk AM, Gribbestad IS, Axelson DE, Skranes J. Combining clinical assessment scores and in vivo mr spectroscopy neurometabolites in very low birth weight adolescents. Artificial Intelligence in Medicine 2009;47:135-46. doi: https://doi.org/10.1016/j.artmed.2009.04.001. |
|  | Bathen TF, Sjöbakk TE, Skranes J, et al. Cerebral metabolite differences in adolescents with low birth weight: Assessment with in vivo proton mr spectroscopy. Pediatr Radiol 2006;36:802-9. doi: https://doi.org/10.1007/s00247-006-0159-5. |
|  | Batinelli L, Serafini A, Nante N, Petraglia F, Severi FM, Messina G. Induction of labour: Clinical predictive factors for success and failure. J Obstet Gynaecol 2018;38:352-58. doi: https://doi.org/10.1080/01443615.2017.1361388. |
|  | Baugh N, Harris DE, Aboueissa AM, Sarton C, Lichter E. The impact of maternal obesity and excessive gestational weight gain on maternal and infant outcomes in maine: Analysis of pregnancy risk assessment monitoring system results from 2000 to 2010. J Pregnancy 2016;2016:5871313. doi: https://doi.org/10.1155/2016/5871313. |
|  | Baumgartner CF, Kamnitsas K, Matthew J, et al. Sononet: Real-time detection and localisation of fetal standard scan planes in freehand ultrasound. IEEE Trans Med Imaging 2017;36:2204-15. doi: https://doi.org/10.1109/tmi.2017.2712367. |
|  | Baye Mulu G, Gebremichael B, Wondwossen Desta K, Adimasu Kebede M, Asmare Aynalem Y, Bimirew Getahun M. Determinants of low birth weight among newborns delivered in public hospitals in addis ababa, ethiopia: Case-control study. Pediatric Health Med Ther 2020;11:119-26. doi: https://doi.org/10.2147/phmt.S246008. |
|  | Baykal N, Reggia JA, Yalabik N, Erkmen A, Beksac MS. Interpretation of doppler blood flow velocity waveforms using neural networks. Proc Annu Symp Comput Appl Med Care 1994:865-9. doi, PMID: https://www.ncbi.nlm.nih.gov/pubmed/7950048. |
|  | Bean Jaworski JL, Flynn T, Burnham N, et al. Rates of autism and potential risk factors in children with congenital heart defects. Congenit Heart Dis 2017;12:421-29. doi: https://doi.org/10.1111/chd.12461. |
|  | Becker JH, Krikhaar A, Schuit E, et al. The added predictive value of biphasic events in st analysis of the fetal electrocardiogram for intrapartum fetal monitoring. Acta Obstet Gynecol Scand 2015;94:175-82. doi: https://doi.org/10.1111/aogs.12548. |
|  | Becker JH, Kuipers LJ, Schuit E, et al. Predictive value of the baseline t-qrs ratio of the fetal electrocardiogram in intrapartum fetal monitoring: A prospective cohort study. Acta Obstet Gynecol Scand 2012;91:189-97. doi: https://doi.org/10.1111/j.1600-0412.2011.01320.x. |
|  | Beg KA, Haq A, Amanullah M, et al. Distinctive hemodynamics in the immediate postoperative period of patients with a longer cardiac intensive care stay post-tetralogy of fallot repair. Congenit Heart Dis 2015;10:346-53. doi: https://doi.org/10.1111/chd.12259. |
|  | Beksaç MS, Başaran F, Eskiizmirliler S, Erkmen AM, Yörükan S. A computerized diagnostic system for the interpretation of umbilical artery blood flow velocity waveforms. Eur J Obstet Gynecol Reprod Biol 1996;64:37-42. doi: https://doi.org/10.1016/0301-2115(95)02256-2. |
|  | Beksaç MS, Durak B, Ozkan O, et al. An artificial intelligent diagnostic system with neural networks to determine genetical disorders and fetal health by using maternal serum markers. Eur J Obstet Gynecol Reprod Biol 1995;59:131-6. doi: https://doi.org/10.1016/0028-2243(94)02034-c. |
|  | Beksaç MS, Egemen A, Izzetoglu K, Ergün G, Erkmen AM. An automated intelligent diagnostic system for the interpretation of umbilical artery doppler velocimetry. Eur J Radiol 1996;23:162-7. doi: https://doi.org/10.1016/0720-048x(96)01067-4. |
|  | Beksac MS, Tanacan A, Bacak HO, Leblebicioglu K. Computerized prediction system for the route of delivery (vaginal birth versus cesarean section). J Perinat Med 2018;46:881-84. doi: https://doi.org/10.1515/jpm-2018-0022. |
|  | Belfort MA, White GL, Vermeulen FM. Association of fetal cranial shape with shoulder dystocia. Ultrasound Obstet Gynecol 2012;39:304-9. doi: https://doi.org/10.1002/uog.9066. |
|  | Bellido-González M, Díaz-López M, López-Criado S, Maldonado-Lozano J. Cognitive functioning and academic achievement in children aged 6-8 years, born at term after intrauterine growth restriction and fetal cerebral redistribution. J Pediatr Psychol 2017;42:345-54. doi: https://doi.org/10.1093/jpepsy/jsw060. |
|  | Benalcazar-Parra C, Ye-Lin YY, Garcia-Casado J, et al. Prediction of labor induction success from the uterine electrohysterogram. Journal of Sensors 2019;2019:12. doi: https://doi.org/10.1155/2019/6916251. |
|  | Bender WR, Koelper NC, Sammel MD, Durnwald C. Validation of a breastfeeding history questionnaire for the risk of in-hospital formula supplementation among multiparous women. J Hum Lact 2019;35:665-71. doi: https://doi.org/10.1177/0890334419868157. |
|  | Bengtson MB, Haugen M, Brantsæter AL, Aamodt G, Vatn MH. Intake of dairy protein during pregnancy in ibd and risk of sga in a norwegian population-based mother and child cohort. BMC Gastroenterol 2020;20:28. doi: https://doi.org/10.1186/s12876-020-1182-y. |
|  | Benhalima K, Van Crombrugge P, Moyson C, et al. Estimating the risk of gestational diabetes mellitus based on the 2013 who criteria: A prediction model based on clinical and biochemical variables in early pregnancy. Acta Diabetol 2020. doi: https://doi.org/10.1007/s00592-019-01469-5. |
|  | Ben-Haroush A, Farhi J, Zahalka Y, Sapir O, Meizner I, Fisch B. Small antral follicle count (2-5 mm) and ovarian volume for prediction of pregnancy in in vitro fertilization cycles. Gynecol Endocrinol 2011;27:748-52. doi: https://doi.org/10.3109/09513590.2010.526668. |
|  | Benjamin SJ, Daniel AB, Kamath A, Ramkumar V. Anthropometric measurements as predictors of cephalopelvic disproportion: Can the diagnostic accuracy be improved? Acta Obstet Gynecol Scand 2012;91:122-7. doi: https://doi.org/10.1111/j.1600-0412.2011.01267.x. |
|  | Benke K, Ágg B, Mátyás G, et al. Gene polymorphisms as risk factors for predicting the cardiovascular manifestations in marfan syndrome. Role of folic acid metabolism enzyme gene polymorphisms in marfan syndrome. Thromb Haemost 2015;114:748-56. doi: https://doi.org/10.1160/th15-02-0096. |
|  | Bennett AE, Kearney JM. Factors associated with maternal wellbeing at four months post-partum in ireland. Nutrients 2018;10. doi: https://doi.org/10.3390/nu10050609. |
|  | Bennett WL, Chang HY, Levine DM, et al. Utilization of primary and obstetric care after medically complicated pregnancies: An analysis of medical claims data. J Gen Intern Med 2014;29:636-45. doi: https://doi.org/10.1007/s11606-013-2744-2. |
|  | Bentsen MH, Markestad T, Øymar K, Halvorsen T. Lung function at term in extremely preterm-born infants: A regional prospective cohort study. BMJ Open 2017;7:e016868. doi: https://doi.org/10.1136/bmjopen-2017-016868. |
|  | Berger E, Wu A, Smulian EA, et al. Universal versus risk factor-targeted early inpatient postpartum depression screening. J Matern Fetal Neonatal Med 2015;28:739-44. doi: https://doi.org/10.3109/14767058.2014.932764. |
|  | Berger RP, Pak BJ, Kolesnikova MD, et al. Derivation and validation of a serum biomarker panel to identify infants with acute intracranial hemorrhage. JAMA Pediatr 2017;171:e170429. doi: https://doi.org/10.1001/jamapediatrics.2017.0429. |
|  | Berker B, Şükür YE, Kahraman K, et al. Absence of rapid and linear progressive motile spermatozoa "grade a" in semen specimens: Does it change intrauterine insemination outcomes? Urology 2012;80:1262-6. doi: https://doi.org/10.1016/j.urology.2012.07.004. |
|  | Berkhout DJC, Klaassen P, Niemarkt HJ, et al. Risk factors for necrotizing enterocolitis: A prospective multicenter case-control study. Neonatology 2018;114:277-84. doi: https://doi.org/10.1159/000489677. |
|  | Berlan E, Mizraji K, Bonny AE. Twelve-month discontinuation of etonogestrel implant in an outpatient pediatric setting. Contraception 2016;94:81-6. doi: https://doi.org/10.1016/j.contraception.2016.02.030. |
|  | Bernard L, Giles A, Fabiano S, et al. Predictors of obstetric fistula repair outcomes in lubango, angola. J Obstet Gynaecol Can 2019;41:1726-33. doi: https://doi.org/10.1016/j.jogc.2019.01.025. |
|  | Berntorp K, Anderberg E, Claesson R, Ignell C, Källén K. The relative importance of maternal body mass index and glucose levels for prediction of large-for-gestational-age births. BMC Pregnancy Childbirth 2015;15:280. doi: https://doi.org/10.1186/s12884-015-0722-x. |
|  | Bertelsen CA. Complete mesocolic excision an assessment of feasibility and outcome. Dan Med J 2017;64. doi, PMID: https://www.ncbi.nlm.nih.gov/pubmed/28157065. |
|  | Bertozzi S, Londero AP, Salvador S, et al. Influence of the couple on hypertensive disorders during pregnancy: A retrospective cohort study. Pregnancy Hypertens 2011;1:156-63. doi: https://doi.org/10.1016/j.preghy.2011.01.005. |
|  | Besson P, Bernasconi N, Colliot O, Evans A, Bernasconi A. Surface-based texture and morphological analysis detects subtle cortical dysplasia. Med Image Comput Comput Assist Interv 2008;11:645-52. doi: https://doi.org/10.1007/978-3-540-85988-8_77. |
|  | Beta J, Issat T, Nowicka MA, Jakimiuk AJ. Risk factors for cesarean section after using the foley catheter for labor induction. Ginekol Pol 2013;84:359-62. doi: https://doi.org/10.17772/gp/1589. |
|  | Betts KS, Kisely S, Alati R. Predicting common maternal postpartum complications: Leveraging health administrative data and machine learning. Bjog 2019;126:702-09. doi: https://doi.org/10.1111/1471-0528.15607. |
|  | Betts KS, Salom CL, Williams GM, Najman JM, Alati R. Associations between self-reported symptoms of prenatal maternal infection and post-traumatic stress disorder in offspring: Evidence from a prospective birth cohort study. J Affect Disord 2015;175:241-7. doi: https://doi.org/10.1016/j.jad.2015.01.011. |
|  | Betts KS, Williams GM, Najman JM, Alati R. Maternal depressive, anxious, and stress symptoms during pregnancy predict internalizing problems in adolescence. Depress Anxiety 2014;31:9-18. doi: https://doi.org/10.1002/da.22210. |
|  | Betts KS, Williams GM, Najman JM, Alati R. The relationship between maternal depressive, anxious, and stress symptoms during pregnancy and adult offspring behavioral and emotional problems. Depress Anxiety 2015;32:82-90. doi: https://doi.org/10.1002/da.22272. |
|  | Beurskens LW, de Jonge R, Schoonderwaldt EM, Tibboel D, Steegers-Theunissen RP. Biomarkers of the one-carbon pathway in association with congenital diaphragmatic hernia. Birth Defects Res A Clin Mol Teratol 2012;94:557-60. doi: https://doi.org/10.1002/bdra.23039. |
|  | Bevilacqua E, Gil MM, Nicolaides KH, et al. Performance of screening for aneuploidies by cell-free DNA analysis of maternal blood in twin pregnancies. Ultrasound Obstet Gynecol 2015;45:61-6. doi: https://doi.org/10.1002/uog.14690. |
|  | Beydoun HA, el-Amin R, McNeal M, Perry C, Archer DF. Reproductive history and postmenopausal rheumatoid arthritis among women 60 years or older: Third national health and nutrition examination survey. Menopause 2013;20:930-5. doi: https://doi.org/10.1097/GME.0b013e3182a14372. |
|  | Bharadwaj SK, Vishnu Bhat B, Vickneswaran V, Adhisivam B, Bobby Z, Habeebullah S. Oxidative stress, antioxidant status and neurodevelopmental outcome in neonates born to pre-eclamptic mothers. Indian J Pediatr 2018;85:351-57. doi: https://doi.org/10.1007/s12098-017-2560-5. |
|  | Bharatha A, Faughnan ME, Kim H, et al. Brain arteriovenous malformation multiplicity predicts the diagnosis of hereditary hemorrhagic telangiectasia: Quantitative assessment. Stroke 2012;43:72-8. doi: https://doi.org/10.1161/strokeaha.111.629865. |
|  | Bhatikar SR, DeGroff C, Mahajan RL. A classifier based on the artificial neural network approach for cardiologic auscultation in pediatrics. Artif Intell Med 2005;33:251-60. doi: https://doi.org/10.1016/j.artmed.2004.07.008. |
|  | Bhatti S, Cordina M, Penna L, Sherwood R, Dew T, Kametas NA. The effect of ethnicity on the performance of protein-creatinine ratio in the prediction of significant proteinuria in pregnancies at risk of or with established hypertension: An implementation audit and cost implications. Acta Obstet Gynecol Scand 2018;97:598-607. doi: https://doi.org/10.1111/aogs.13303. |
|  | Bhide A, Caric V, Arulkumaran S. Prediction of vaginal birth after cesarean delivery. Int J Gynaecol Obstet 2016;133:297-300. doi: https://doi.org/10.1016/j.ijgo.2015.09.031. |
[truncated: 471,736 more chars]
